# Oxyalkylation of Alkenes via Triple Radical Sorting

**DOI:** 10.1021/jacs.6c03814

**Published:** 2026-06-06

**Authors:** Lauren J. Harstad, David W. C. MacMillan

**Affiliations:** 6740 Merck Center for Catalysis at Princeton University, Princeton, New Jersey 08544, United States

## Abstract

Alkene difunctionalization enables rapid access to structurally diverse chemical space. However, oxyalkylation, where an oxygenated group and an alkyl fragment are simultaneously installed across an alkene, remains underexplored. Whereas formal Markovnikov oxyalkylation has been known for several decades, the development of a general method with reversed selectivity remains a notable challenge. Such a transformation would streamline the synthesis of pharmaceutically relevant scaffolds including hindered all-carbon quaternary centers. Herein, we report a three-component oxyalkylation of unactivated alkenes with anti-Markovnikov selectivity via a metallaphotoredox-based ‘triple radical sorting’ mechanism. This protocol couples alkenes with benzoic acids and redox-active esters in a single step and accommodates a range of substrates including complex, drug-like molecules. The reaction is readily scalable and can be adapted to a one-flask oxyalkylation/deprotection sequence to directly furnish free alcohols without intermediate purification.

## Graphical abstract

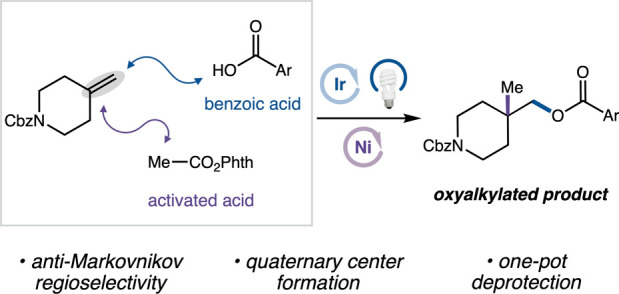

Organic synthesis is fundamental to small-molecule drug discovery, providing access to a wide range of pharmaceutical scaffolds.^1^ Yet in early discovery efforts, where speed and structural diversity are paramount, the pace of analogue construction remains a key bottleneck.^2^ Coupling strategies such as alkene functionalization offer a powerful approach for the efficient assembly of novel building blocks and complex architectures.^3,4^ This includes established two- and three-component reactions for the alkylation or oxygenation of alkenes such as hydroalkylation,^5,6^ oxyfunctionalization,^7–10^ dialkylation,^11–15^ and dihydroxylation.^16^ In contrast, alkene oxyalkylation, which involves the selective introduction of both an oxygenated group and an alkyl fragment, remains notably underdeveloped.

Classically, formal olefin oxyalkylation proceeds through a two-part sequence of alkene epoxidation followed by nucleophilic ring-opening, furnishing the Markovnikov-type product with high regioselectivity (Figure 1a).^17^ Beyond its multistep nature, this protocol relies on strong oxidizing agents such as *m*-CPBA and harsh organometallic nucleophiles, which significantly limit functional group compatibility. In recent years, select oxyalkylated motifs have been accessed in a single step, either with the same Markovnikov selectivity,^18,19^ or in rare cases with reversed selectivity for specific styrenes or intramolecular systems.^20,21^ However, *a general oxyalkylation strategy that affords the anti-Markovnikov product remains unknown.* Such a transformation would streamline access to complex C­(sp^3^)-rich scaffolds including hindered all-carbon quaternary centers, which are correlated with enhanced target binding for small molecule drugs.^22,23^ Moreover, the installed oxygenated functional group can serve as a handle for further derivatization, or may itself confer beneficial pharmacological properties such as improved aqueous solubility.^24^

**Figure 1:**
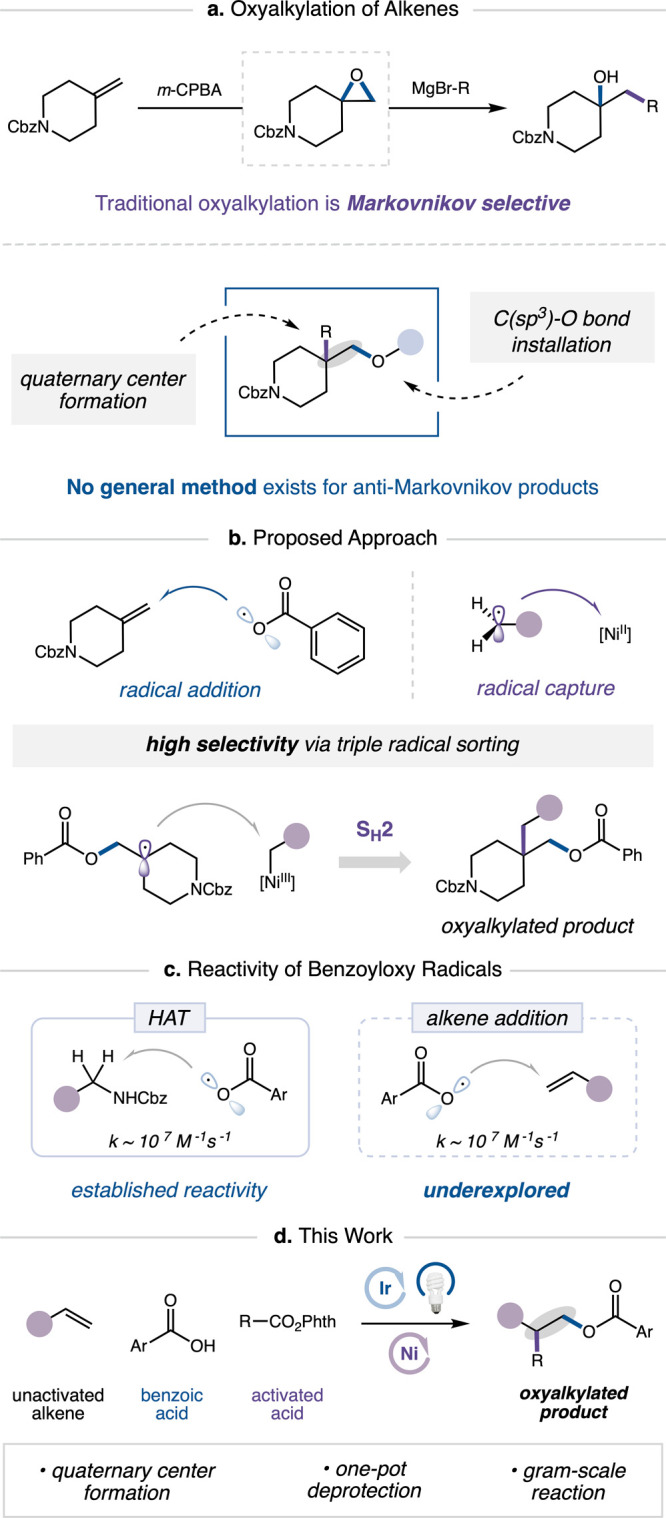
Oxyalkylation of alkenes.

In designing an approach to this challenging transformation, we envisioned a reaction in which both an oxygen-centered radical and an alkyl radical selectively engage the alkene to deliver the oxyalkylated product in a single step. We were particularly interested in exploring oxyalkylation using benzoic acids, which readily furnish benzoyloxy radicals upon oxidation of the corresponding benzoate (*E*_p/2_ = +1.40 V vs SCE in MeCN).^25^ Benzoic acids are common feedstock chemicals and, importantly, would yield protected alcohol products that can undergo facile hydrolysis to reveal free hydroxyl groups. We proposed that the electrophilic benzoyloxy radical would perform regioselective addition across a nucleophilic alkene to generate a hindered alkyl radical (Figure 1b). Subsequently, this species could be engaged in a metal-mediated C­(sp^3^)–C­(sp^3^) bond forming reaction to yield the desired oxyalkylated product.

Recently, our group has disclosed a novel mechanism for the formation of C­(sp^3^)–C­(sp^3^) bonds through a bimolecular homolytic substitution (S_H_2) pathway.^26–28^ This approach involves the capture of a primary alkyl radical by an iron- or nickel-based catalyst, followed by substitution with a more hindered alkyl radical to furnish the desired bond. The S_H_2 paradigm uniquely enables the “sorting” of two transient, differentially substituted radicals, enabling cross-coupling with high selectivity over dimerization and disproportionation pathways. This concept has subsequently been expanded to three-component couplings through generation of an electrophilic carbon or nitrogen-centered radical, which performs alkene addition to generate a hindered radical for subsequent alkylation through S_H_2.^13,29,30^ We questioned whether a “triple radical sorting” approach could be leveraged to develop a highly selective anti-Markovnikov oxyalkylation protocol via an oxygen-centered radical and two alkyl radical intermediates.

While benzoyloxy radicals are sufficiently electrophilic to add to nucleophilic alkenes,^9,31,32^ we recognized several mechanistic challenges that must be overcome to achieve a general oxyalkylation platform.^33^ Notably, benzoyloxy radicals have found widespread use for hydrogen atom transfer (HAT), capable of abstracting strong, unactivated C­(sp^3^)–H bonds as well as polarity-matched hydridic sites (Figure 1c).^34,35^ Additionally, these species can generate aryl radicals via decarboxylation^36–38^ or react with arenes to furnish diaryl esters.^39^ Thus, achieving an efficient oxyalkylation process would require outcompeting numerous deleterious but kinetically facile pathways.

Herein, we report the successful realization of a nickel-mediated three-component coupling of unactivated alkenes, benzoic acids, and alkyl redox-active esters (RAEs) to form oxyalkylated products (Figure 1d). This method enables the synthesis of a diverse scope of molecules with wide functional group tolerance, including products with congested C­(sp^3^)-rich motifs such as quaternary centers. The reaction is readily scalable and can be applied to the late-stage functionalization of complex molecules. We further demonstrate the facile deprotection of the ester product to furnish a free alcohol that can be elaborated into a diverse suite of formally difunctionalized products.

We sought to employ a redox-neutral, metallaphotoredox-based radical sorting platform where a photocatalyst would oxidatively generate the oxygen-centered radical, while the primary alkyl radical would result from reductive cleavage of a redox-active ester.^40^ Once formed, the benzoyloxy radical would add across the nucleophilic alkene, and an appropriate S_H_2 catalyst would be utilized for the subsequent alkylation event. Optimized conditions with iridium-based photocatalyst **1** and nickel-based S_H_2 catalyst **2**—proposed to form from Ni­(acac)_2_ and potassium tri­(3,5-dimethyl-1-pyrazolyl)­borohydride (KTp*)—enabled the coupling of 2-methoxybenzoic acid, methyl redox-active ester (Me-RAE, **3**), and unactivated alkene **4**, affording the oxyalkylated product **5** in 75% yield (Table 1, entry 1). Key to high reaction efficiency was the use of the Tp* ligand, which has been shown to improve selectivity for methylation reactions via an S_H_2 pathway (Table 1, entry 2).^27^ Substoichiometric loading of the phosphazene base *t*-butylimino-tri­(pyrrolidino)-phosphorane (BTTP) was additionally required for optimal activity (Table 1, entry 3). This may improve yields in part by minimizing base-mediated decomposition of the RAE. We propose that phthalimide anion, generated in situ upon reduction of the RAE, acts as additional base. Control reactions confirmed the necessity of the catalysts as well as light for product formation (Table 1, entries 5–7; see the SI for additional optimization studies and proposed mechanism).

**Table 1:**
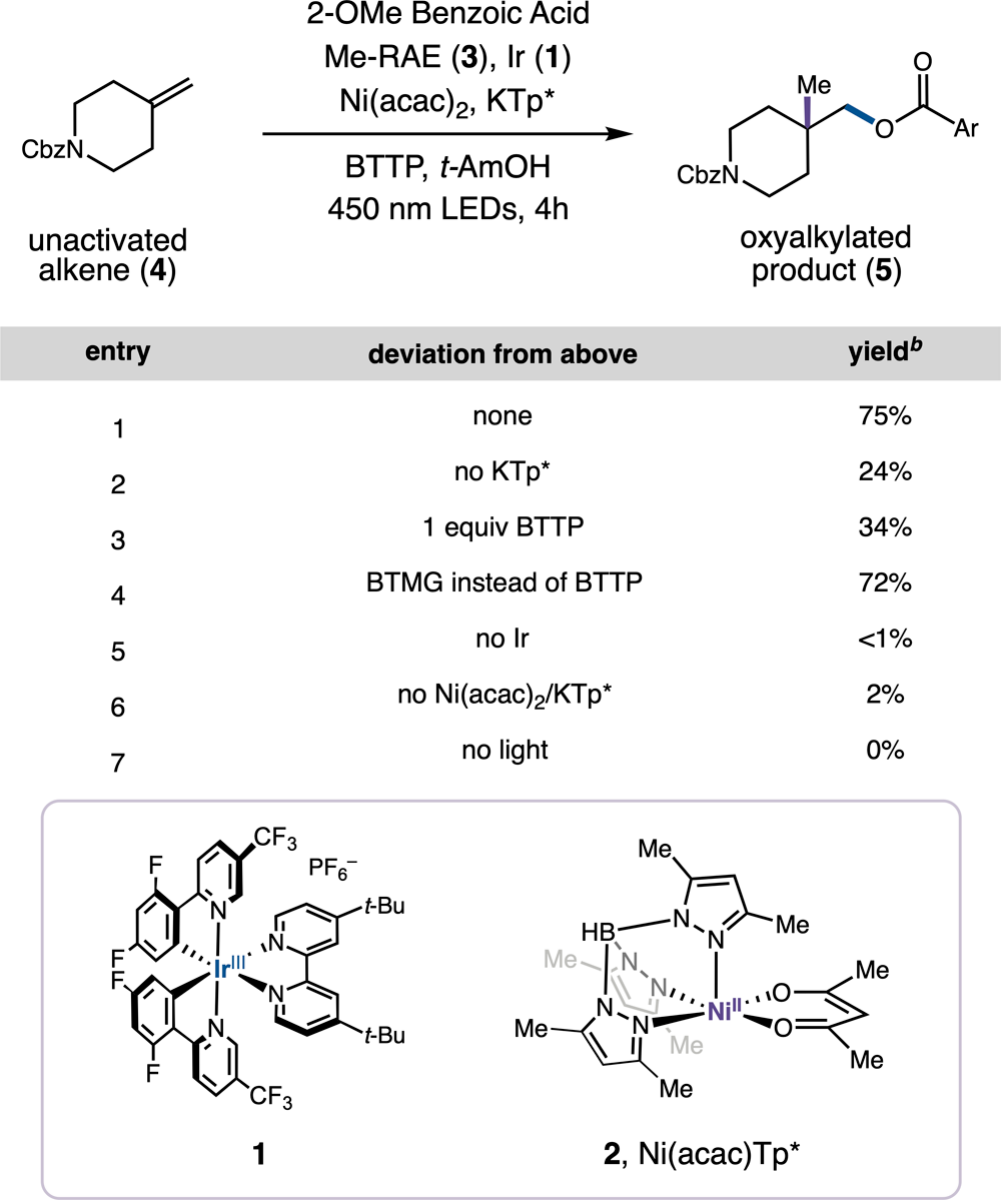
Optimization and Control Reactions[Table-fn t1fn2]

a Performed on 0.05 mmol scale with 2-methoxybenzoic acid (1 equiv), Me-RAE (**3**, 2 equiv), alkene (**4**, 2 equiv), Ni­(acac)_2_ (5 mol %), KTp* (5 mol %), Ir (**1**, 2 mol %), BTTP (0.2 equiv), *t*-AmOH (0.05 M), integrated photoreactor (450 nm, 100% intensity (3.4 W), 35 °C), 4 h, with deviations as noted. ^*b*^Yield determined by ^1^H NMR analysis. See the SI for experimental details. Ar = 2-methoxybenzene.

With optimized conditions in hand, we next sought to investigate the scope of alkenes that could be utilized in the reaction. Terminal vinyl ethers and oxazolidinones furnished oxyalkylated products in good yield, tolerating sensitive alkyl and aryl halides (Table 2, **6**–**9**, 61–76% yield). Acyclic 1,1-disubstituted alkenes bearing free alcohols, homolytically labile allylic esters, and base-sensitive epoxides reacted efficiently to form synthetically challenging all-carbon quaternary centers (**10**–**14**, 63–76% yield). (*S*)-Carvone was selectively functionalized at the electron-rich alkene to afford **15** in 60% yield. Exocyclic 1,1-disubstituted alkenes of various ring sizes were also successful (**16**–**20**, 44–93% yield), including those with easily abstractable α-amino and α-oxy C–H bonds. Notably, this method enables construction of highly elusive vicinal quaternary centers, with **19** being formed in 72% yield. Di- and trisubstituted vinyl ethers and a 1,2-disubstituted enamine reacted smoothly to deliver **21**–**24** (64–84% yield), and both cyclic and acyclic unactivated trisubstituted alkenes proved to be viable coupling partners (**25**–**29**, 53–89% yield). Finally, the oxyalkylation of alkene **4** was readily scaled 8-fold to 4 mmol scale, producing over 1 g of **5** using the standard photoreactor equipment.

**Table 2:**
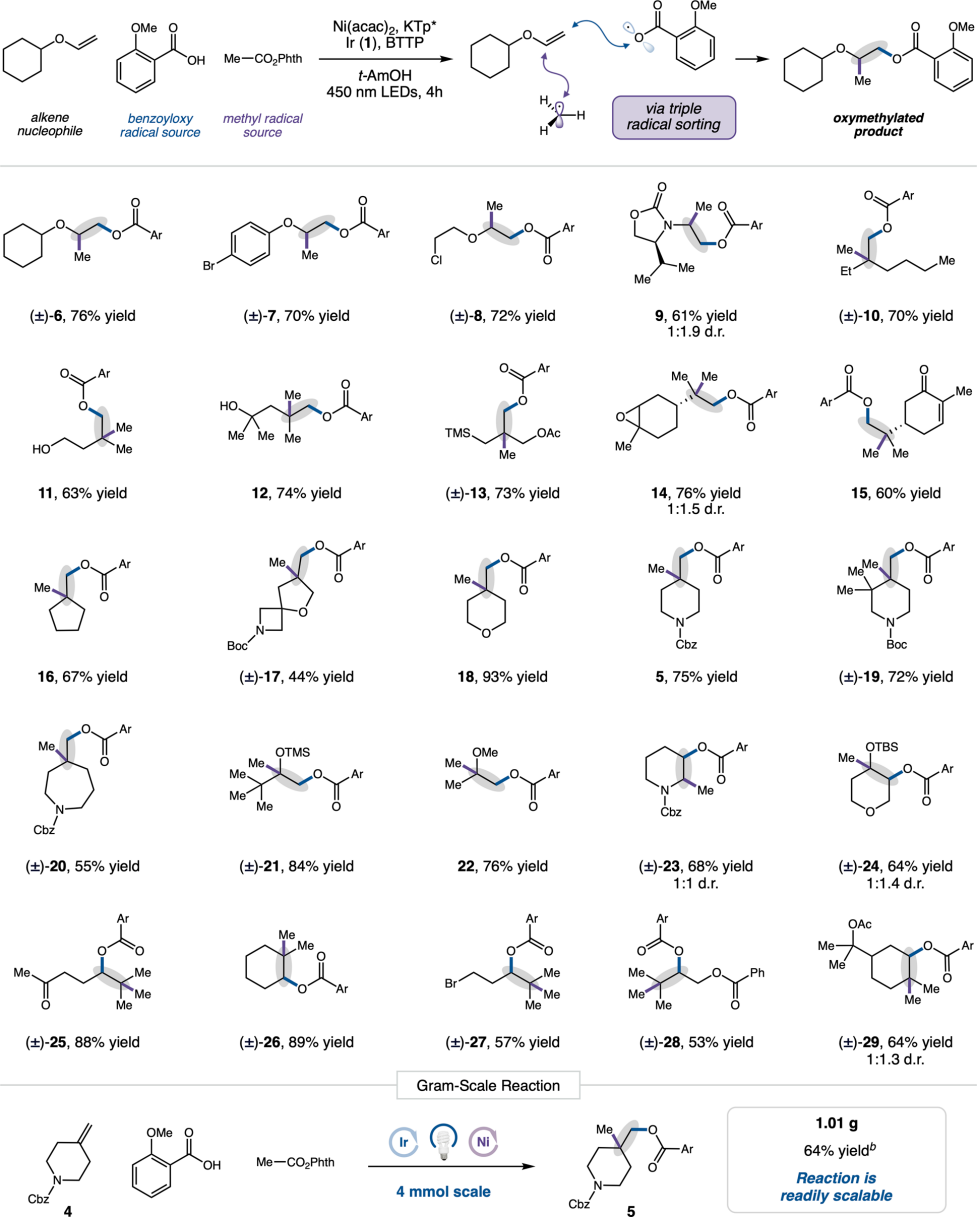
Alkene Scope^a^

a Performed on 0.5 mmol scale with 2-methoxybenzoic acid (1 equiv), Me-RAE (2 equiv), alkene (2 equiv), Ni­(acac)_2_ (5 mol %), KTp* (5 mol %), Ir (**1**, 2 mol %), BTTP (0.2 equiv), *t*-AmOH (0.05 M), integrated photoreactor (450 nm, 100% intensity (3.4 W), 35 °C), 4 h. All yields isolated. ^*b*^4 mmol scale, *t*-AmOH (0.13 M). Ar = 2-methoxybenzene, Phth = phthalimide.

We next turned our attention to the reaction scope with respect to the benzoic acid component. Gratifyingly, a range of different substitutions could be tolerated, including sensitive benzylic alcohols, semisaturated dioxane cores, and aryl halides (Table 3, **30–41**, 51–92% yield). Throughout these studies, we found that electron-rich benzoic acids generally react with high efficiency, likely in part due to a lower oxidation potential of the benzoate. Furthermore, the corresponding benzoyloxy radicals are slower at performing deleterious processes including decarboxylation, HAT, and arene addition.^41,42^ Despite differences in reactivity, both electron-neutral and weakly electron-deficient acids could be incorporated in synthetically useful yields (see the SI for additional scope and discussion).

**Table 3:**
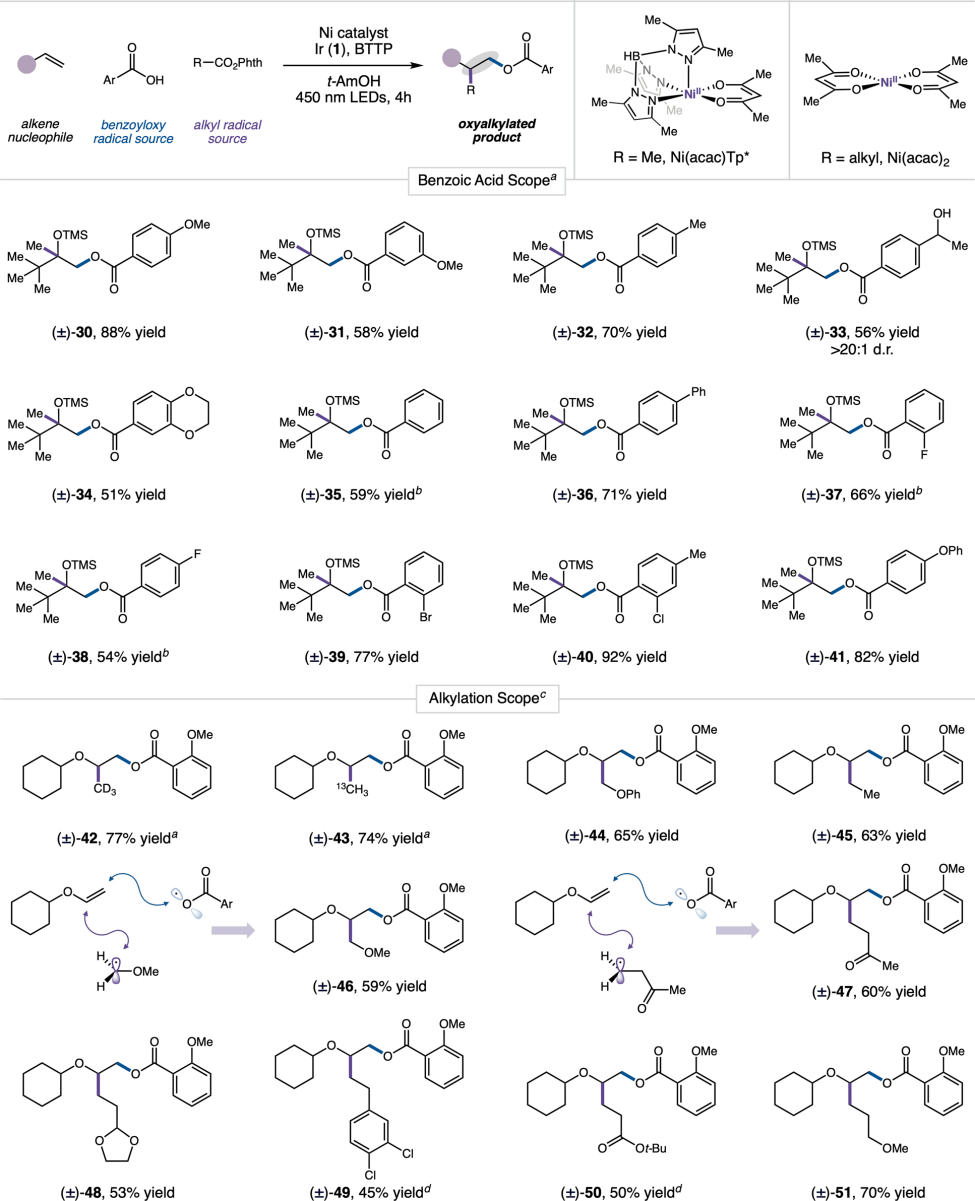
Benzoic Acid and Alkylation Scope[Table-fn t3fn1]

* Performed on 0.5 mmol scale. Isolated yield reported unless otherwise noted. ^*a*^Benzoic acid (1 equiv), Me-RAE (2 equiv), alkene (2 equiv), Ni­(acac)_2_ (5 mol %), KTp* (5 mol %), Ir (**1**, 2 mol %), BTTP (0.2 equiv), *t*-AmOH (0.05 M), integrated photoreactor (450 nm, 100% intensity (3.4 W), 35 °C), 4 h. ^*b*^3 equiv alkene. ^*c*^2-methoxybenzoic acid (1 equiv), RAE (3 equiv), alkene (3 equiv), Ni­(acac)_2_ (15 mol %), Ir (**1**, 2 mol %), BTTP (0.2 equiv), *t*-AmOH (0.05 M), integrated photoreactor (450 nm, 100% intensity (3.4 W), 35 °C), 4 h. ^*d*^Yield determined by ^1^H NMR analysis. Phth = phthalimide.

Finally, we set out to explore the scope of the alkyl coupling partner. Deuterated and ^13^C-labeled methyl groups, originating from the corresponding acetic acid–based precursor, were incorporated in good yields (**42** and **43**, 77 and 74% yield, respectively). This demonstrates the power of our method to rapidly access isotopically labeled products from abundant sources. In evaluating the formation of phenyl ether **44**, we found that exclusion of the Tp* ligand was necessary for efficient reactivity. This finding may be attributed to decreased steric bulk at the metal center compared to the scorpionate catalyst, resulting in a more efficient S_H_2 reaction for longer alkyl chains. Upon further optimization (see the SI for details), **44** was formed in 65% yield. These alternate conditions proved compatible with a range of alkyl functionalities, including ethers, ketones, esters, acetals, and aryl groups (**45**–**51**, 45–70% yield).

To probe the late-stage oxyalkylation of complex alkenes, several industrially relevant substrates were utilized for oxyalkylation (Table 4). Functionalization of pharmaceutical-based scaffolds, including celecoxib and ataluren cores, delivered **52** and **55** in 51 and 42% yield, respectively. Etonogestrel and (+)-nootkatone were exclusively functionalized at the nucleophilic alkene with the remaining π-systems left intact (**53** and **54**, 36 and 65% yield, respectively). Additionally, pharmaceutically active benzoic acids, including meglitinide, aspirin, and lumacaftor, were transformed into diverse oxyalkylated analogues (**56**, **57**, and **60**, 34–61% yield). A methylated analogue of the polyketide orsellinic acid was functionalized in 69% yield (**58**), and a protected form of diflunisal was utilized to give sterically congested **59** in 53% yield.

**Table 4:**
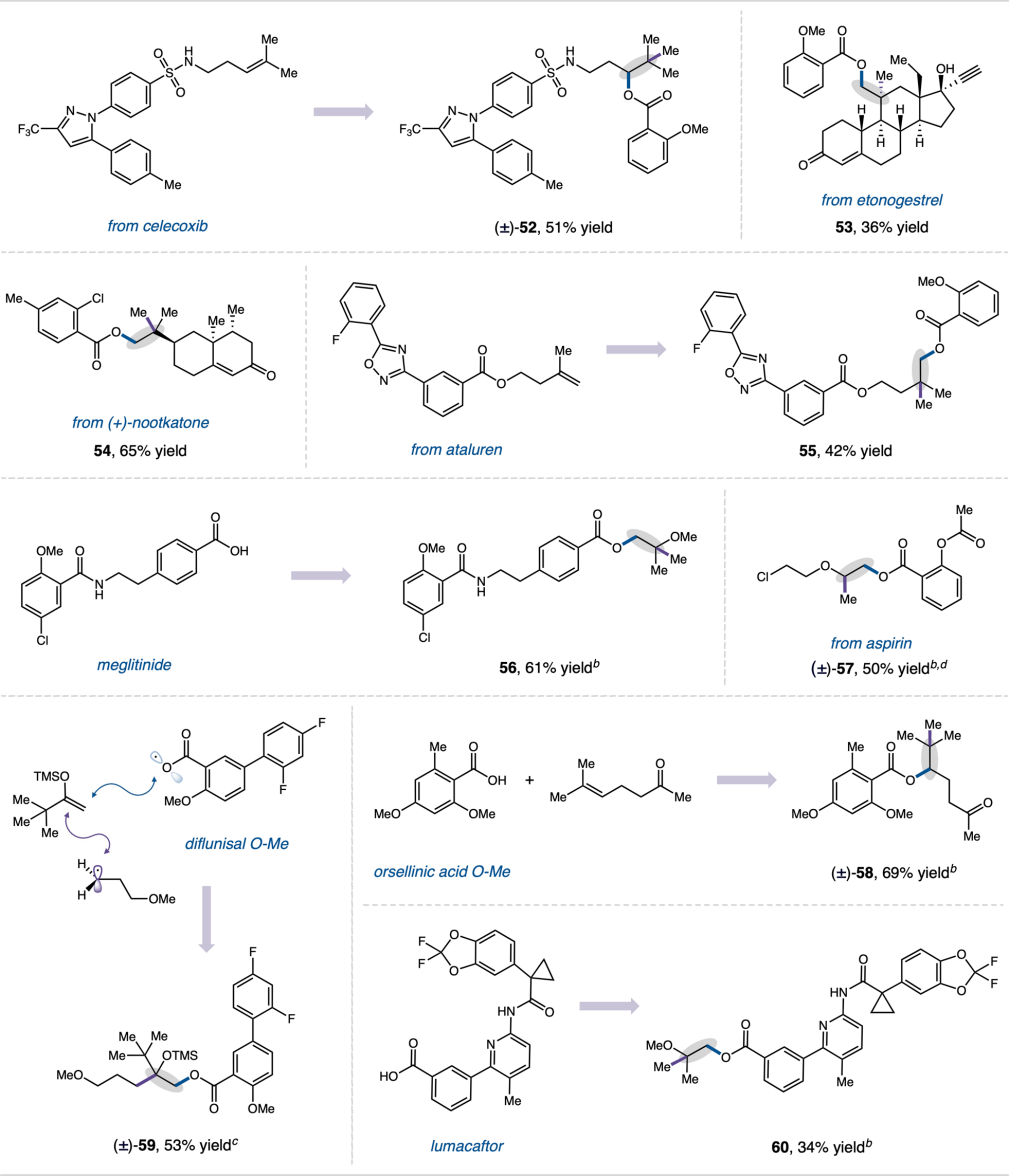
Derivatives of Pharmaceuticals and Natural Products^a^

a Performed on 0.5 mmol scale. Isolated yield reported unless otherwise noted. Benzoic acid (1 equiv), Me-RAE (2 equiv), alkene (2 equiv), Ni­(acac)_2_ (5 mol %), KTp* (5 mol %), Ir (**1**, 2 mol %), BTTP (0.2 equiv), *t*-AmOH (0.05 M), integrated photoreactor (450 nm, 100% intensity (3.4 W), 35 °C), 4 h. ^*b*^3 equiv alkene. ^*c*^Benzoic acid (1 equiv), RAE (3 equiv), alkene (3 equiv), Ni­(acac)_2_ (15 mol %), Ir (**1**, 2 mol %), BTTP (0.2 equiv), *t*-AmOH (0.05 M), integrated photoreactor (450 nm, 100% intensity (3.4 W), 35 °C), 4 h. ^*d*^Yield determined by ^1^H NMR analysis.

To further demonstrate the utility of this method, we developed conditions for a one-flask oxyalkylation/deprotection sequence, as outlined in Table 5. This transformation highlights the ability of benzoic acids to serve as a hydroxyl radical surrogate, furnishing alcohol **61** in a single vessel without workup or isolation of the intermediate ester. The resulting alcohol provides a handle for further diversification, including radical deoxygenative functionalizations recently disclosed by our laboratory. These methods rely on the activation of alcohols by benzoxazolium salts (or “NHC” reagents, **62**), generating an adduct (**63**) that can be oxidatively activated to produce an alkyl radical.^43,44^ Utilizing this technology in conjunction with the initial oxyalkylation/deprotection sequence provided rapid access to a suite of formally difunctionalized products via deoxygenative methylation, arylation, phosphonylation, and sulfination (**64**–**67**, 51–84% yield).^43,45–47^

**Table 5:**
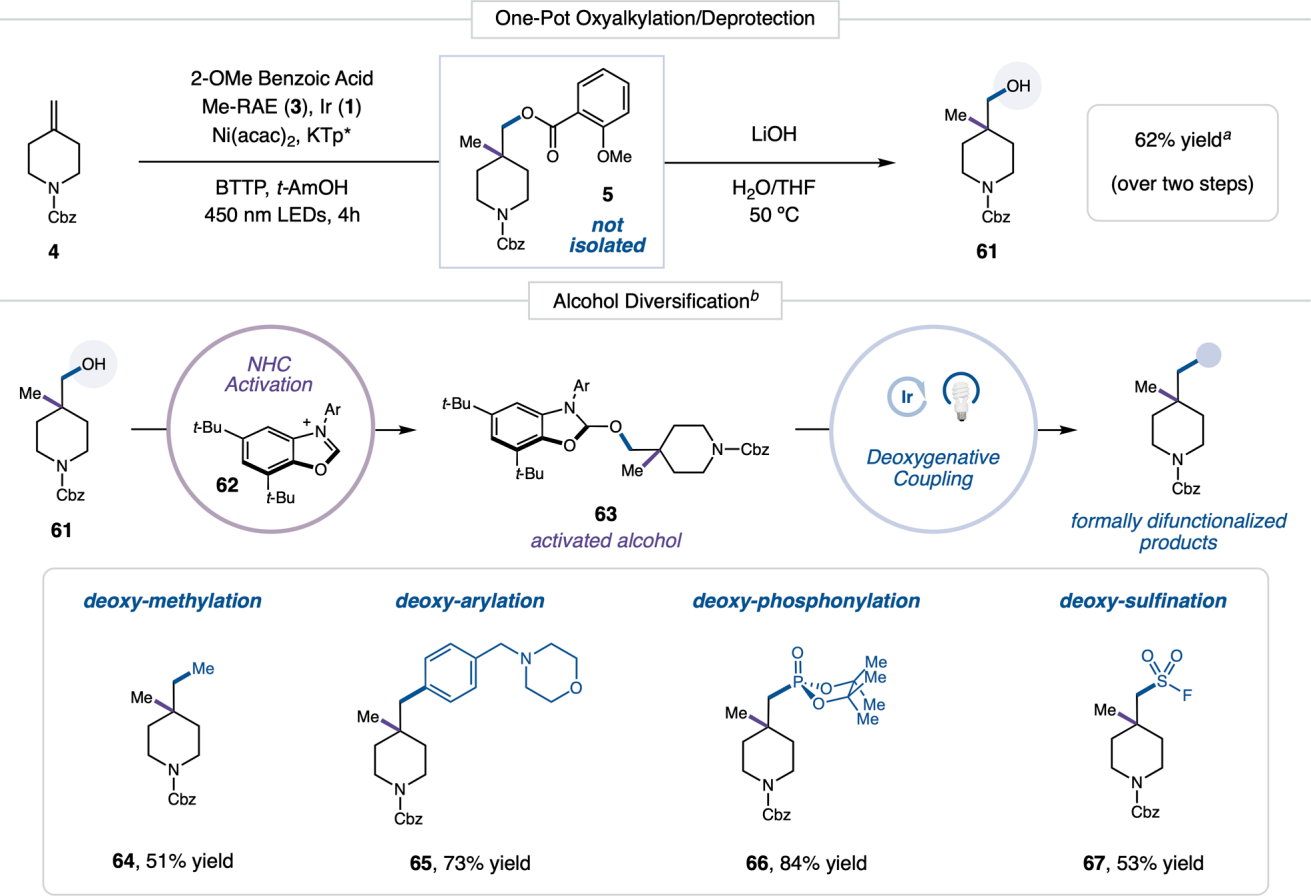
One-Pot Deprotection and Alcohol Diversification[Table-fn t5fn1]

* Performed on 0.5 mmol scale. Isolated yield reported. ^*a*^2-Methoxybenzoic acid (1 equiv), Me-RAE (2 equiv), alkene (2 equiv), Ni­(acac)_2_ (5 mol %), KTp* (5 mol %), Ir (**1**, 2 mol %), BTTP (0.2 equiv), *t*-AmOH (0.05 M), integrated photoreactor (450 nm, 100% intensity (3.4 W), 35 °C), 4 h, then 1:1 aq LiOH/THF, 50 °C. ^*b*^See the SI for experimental details.

In conclusion, we have developed a general alkene difunctionalization method for the anti-Markovnikov installation of a C­(sp^3^)–O and C­(sp^3^)–C­(sp^3^) bond in a single step. This transformation employs a broad range of alkenes, benzoic acids, and primary alkyl coupling partners, including complex pharmaceuticals and natural products. A one-flask procedure was developed to directly deliver the alcohol product via oxyalkylation/hydrolysis, and this species was successfully harnessed for downstream derivatization. We anticipate that this methodology will find broad utility in both scaffold construction and late-stage functionalization of industrially relevant molecules.

## Supplementary Material



## Notes

Additional experimental details, optimization studies, reaction scope, mechanistic studies, characterization, and spectra (PDF)
