# Supplementary material for: Oxyalkylation of Alkenes via Triple Radical Sorting
Source: J Am Chem Soc. 2026 Jun 6;148(23):23435–42. doi: 10.1021/jacs.6c03814 (PMC13281523; doi:10.1021/jacs.6c03814)
Supplement: Supplementary file 1 [file ja6c03814_si_001.pdf]

Supplementary Information

Oxyalkylation of Alkenes via Triple Radical Sorting

Lauren J. Harstad and David W. C. MacMillan†

Merck Center for Catalysis at Princeton University, Princeton, New Jersey 08544, USA

†Corresponding author. Email: dmacmill@princeton.edu

Table of Contents

1. **General Information ………………………………………………………………… 3**
2. **Proposed Mechanism ……………………………………………………………….. 6**
3. **Synthesis and Characterization of Starting Materials ………………………………. 7**
4. **Select Reaction Optimization and Control Experiments …………………………... 22**
5. **Extended Scope ……………………………………………………………………….. 37**
6. **Mechanistic Experiments……………………………………………………………... 42**
7. **General Procedures for Oxyalkylation ……………….…………………………….... 55**
8. **Additional Procedures: 4 mmol Scale Reaction and One-Pot Oxyalkylation/ Hydrolysis ……………………………………………………………………………... 58**
9. **Experimental Data ……………………………….……………………………………. 61**
10. **NMR Spectra ……………………………………….…………………………………... 134**
11. **References ……………………………………………………………………………. 224**

1. General Information

All commercial reagents were used without additional purification unless otherwise noted. $[Ir(dF(CF_3)ppy)_2(dtbbpy)]PF_6$ was prepared according to a known literature procedure,[1-2] but is also commercially available (CAS: 870987-63-6). Key reagents were purchased from commercial suppliers: nickel(II) acetylacetonate ($Ni(acac)_2$, CAS: 3264-82-2), potassium tri(3,5-dimethyl-1-pyrazolyl)borohydride (KTp*, CAS: 17567-17-8), (1,3-dioxoisoindolin-2-yl)acetate (Me-RAE, CAS: 17720-64-8), and *tert*-butylimino-tri(pyrrolidino)phosphorane (BTTP, CAS: 161118-67-8). Solvents were purified via the method reported by Grubbs unless otherwise noted.[3] Certain anhydrous solvents were purchased from commercial suppliers and used as is, including *tert*-amyl alcohol (2-methyl-2-butanol) from Sigma-Aldrich (catalog number: 721123), acetone from Thermo Scientific (catalog number 326800010), and THF from Thermo Scientific (catalog number: 348451000). Organic solvents were removed under reduced pressure on a Büchi rotary evaporator with water bath or using a GeneVac HT-4X Centrifugal Vacuum Evaporator Series II machine.

All photoreactions were performed in a PennPhD m2 integrated photoreactor fitted with a m2 450 nm LED plate. The reaction temperature was controlled with the internal fan of the machine set at 5200 rpm. No additional effort was made to regulate the reaction temperature, although this was measured to be on average 35 ºC across several reactions irradiated in different photoreactors. 100% light intensity was utilized for irradiation, which corresponds to a reported wattage of 3.4 W. Plates may vary in wattage, especially after sustained use, and plates below 2 W can provide significantly diminished yields.

Chromatographic purification of compounds was performed using an automated Teledyne ISCO CombiFlash® NextGen 300+ system with either Biotage® Sfär Silica Normal Phase Flash

Columns (60 µm) or RediSep® Silver Normal Phase Silica Gel Disposable Flash Columns (40–60 µm). Reverse-phase chromatography was performed on a Biotage IsoleraOneTM with a Biotage® Sfär C18 D - Duo (100 Å, 30 µm) column with 0.1% ammonium hydroxide buffered water and acetonitrile solutions. Preparative High Performance Liquid Chromatography (preparative HPLC) purification was performed on a Teledyne ISCO ACCQPrep® HP150 system equipped with a Waters XBridge BEH C18 OBD Prep Column (30 mm x 150 mm, 130 Å, 5 µm) with 0.1% ammonium hydroxide buffered water and acetonitrile solutions.

^{1}H and ^{13}C NMR spectra were recorded on a Bruker Avance III NMR 500 MHz instrument, a Bruker NanoBay Avance III HD NMR 400 MHz instrument, or an Ultrashield NMR 300 MHz instrument and are internally referenced to the residual proteo-solvent signals with $CDCl_3$ referenced at 7.26 ppm and 77.16 ppm, respectively, DMSO-d_6 referenced at 2.50 and 39.52 ppm, respectively, and $(CD_3)_2CO$ referenced at 2.05 ppm and 29.84 ppm. ^{19}F and ^{31}P NMR spectra were recorded on a Bruker Avance III NMR 500 MHz instrument or a Bruker NanoBay Avance III HD NMR 400 MHz instrument and are reported unreferenced. Data for ^{1}H and ^{19}F NMR are reported in the following format: chemical shift (δ ppm), multiplicity (s (singlet), b (broad signal), d (doublet), dd (doublet of doublets), ddd (doublet of doublet of doublets), t (triplet), dt (doublet of triplets), td (triplet of doublets), tt (triplet of triplets), ddt (doublet of doublet of triplets), q (quartet), p (pentet), h (hextet), m (multiplet), coupling constant (Hz), and integration. Data for ^{13}C NMR and ^{31}P NMR (proton decoupled) are reported as chemical shift, with multiplicity and coupling constants included only for observed coupling with ^{19}F or ^{31}P nuclei, as well as ^{13}C or ^{2}H (D) nuclei in the case of isotopically-enriched compounds.

Ultra-Performance liquid chromatography (uHPLC) analysis was performed with an Agilent 1290 Infinity II LC system. Infrared (IR) spectroscopy was performed with a Thermo

Nicolet 6700 FTIR spectrometer (diamond Smart Orbit ATR accessory), with spectra reported in wavenumbers (cm^{-1}). High resolution mass spectra (HRMS) were obtained at the Princeton University Mass Spectral Facility using an Agilent 6220 ESI-TOF LC/MS system.

2. Proposed Mechanism

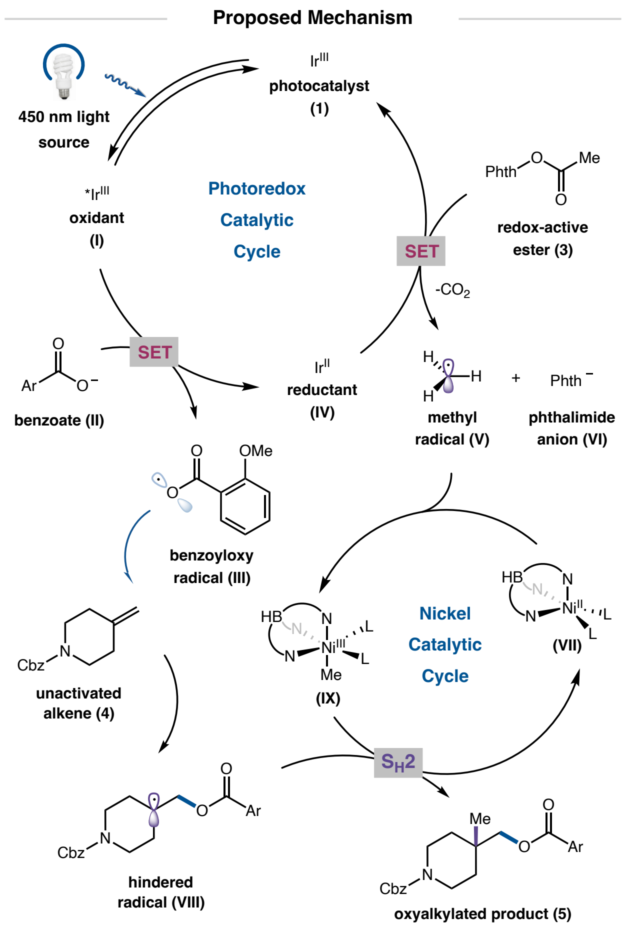

Figure S1: Proposed Mechanism for the Formation of 5

The proposed mechanism for the formation of **5** is shown in Figure S1. Upon blue light excitation, ground-state iridium photocatalyst **1** accesses a long-lived triplet excited state (**I**). This excited photocatalyst oxidizes the benzoate (**II**) to generate the corresponding benzoyloxy radical (**III**). The photocatalytic cycle is closed via reduction of Me-RAE (**3**) by the reduced photocatalyst (**IV**), furnishing methyl radical (**V**) and phthalimide anion (**VI**) upon decarboxylation. Initial electronic sorting of the two radicals occurs through polarity-matched anti-Markovnikov addition of **III** into the nucleophilic alkene (**4**) accompanied by capture of methyl radical (**V**) by a Ni(II) catalyst (**VII**). The resulting hindered radical (**VIII**) and Ni(III)–methyl complex (**IX**) undergo further steric sorting via an S_H2 reaction to deliver the oxyalkylated product (**5**).

3. Synthesis and Characterization of Starting Materials

Synthesis and Characterization of Alkene Substrates:

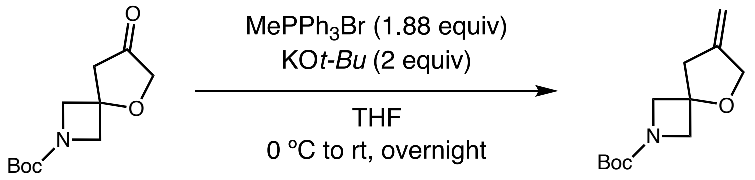

***tert*-butyl 7-methylene-5-oxa-2-azaspiro[3.4]octane-2-carboxylate (S1):** The title compound was prepared according to the following procedure. To a flame-dried 50 mL round bottom flask and stir bar under N_2 was added methyltriphenylphosphonium bromide (1.88 equiv, 3.76 mmol, 1.34 g) and 15 mL of dry THF. The mixture was cooled to 0 ºC and potassium *tert*-butoxide (2 equiv, 4 mmol, 449 mg) was added portionwise. The mixture was stirred at this temperature for 5 minutes and then allowed to warm to room temperature while stirring for 1 hour. The mixture was then cooled back to 0 ºC and *tert*-butyl 7-oxo-5-oxa-2-azaspiro[3.4]octane-2-carboxylate (1 equiv, 2 mmol, 455 mg) added as a single portion. The mixture was diluted with an additional 10 mL of dry THF and stirred at room temperature overnight. The reaction contents were transferred to a separatory funnel with 25 mL of saturated ammonium chloride. This mixture was extracted with DCM (3 x 30 mL). The combined organics were dried over Na_2SO_4 and concentrated. Purification was performed via automated flash chromatography (30 g high performance silica column, 0-25% ethyl acetate/hexanes). The solvent was removed and the product dried on high-vac to yield the title compound as a clear oil (320 mg, 1.42 mmol, 71% yield).

Note: This compound was found to decompose over several months when stored at room temperature.

^{1}H NMR (500 MHz, $CDCl_3$) δ 5.04 (t, J = 2.3 Hz, 1H), 4.95 (t, J = 2.2 Hz, 1H), 4.36 – 4.31 (m, 2H), 3.99 (dd, J = 9.2, 1.1 Hz, 2H), 3.88 (dd, J = 9.1, 1.1 Hz, 2H), 2.75 – 2.67 (m, 2H), 1.43 (s, 9H).

^{13}C NMR (126 MHz, $CDCl_3$) δ 156.50, 145.70, 106.00, 79.74, 78.47, 70.61, 61.14, 42.14, 28.48.

IR (film) v_{max} 2977, 2941, 2877, 1698, 1391, 1365, 1149, 1086, 770 cm^{-1}.

HRMS (ESI-TOF) *m/z* calculated for $C_{12}H_{19}NNaO_3^+$ ([M+Na]$^+$) 248.1257, found 248.1253.

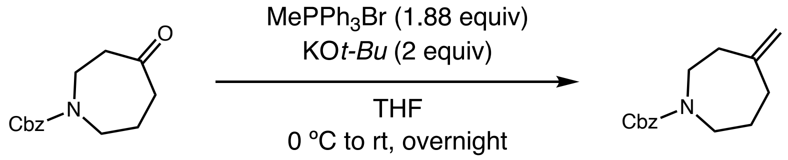

benzyl 4-methyleneazepane-1-carboxylate (S2): The title compound was prepared according to the following procedure. To a flame-dried 50 mL round bottom flask and stir bar under N_2 was added methyltriphenylphosphonium bromide (1.88 equiv, 3.76 mmol, 1.34 g) and 15 mL of dry THF. The mixture was cooled to 0 ºC and potassium *tert*-butoxide (2 equiv, 4 mmol, 449 mg) was added portionwise. The mixture was stirred at this temperature for 5 minutes and then allowed to warm to room temperature while stirring for 1 hour. The mixture was then cooled back to 0 ºC and benzyl 4-oxoazepane-1-carboxylate (1 equiv, 2 mmol, 495 mg) was added as a single portion. The mixture was diluted with an additional 10 mL of dry THF and stirred at room temperature overnight. The reaction contents were transferred to a separatory funnel with 25 mL of saturated ammonium chloride. This mixture was extracted with DCM (3 x 30 mL). The combined organics were dried over Na_2SO_4 and concentrated. Purification was performed via automated flash chromatography (50 g high performance silica column, 0-25% ethyl acetate/hexanes). The solvent was removed and the product dried on high-vac to yield the title compound as a clear oil (480 mg, 1.96 mmol, 98% yield).

^{1}H NMR (500 MHz, CDCl$_3$) (summary of rotamers) δ 7.40 – 7.27 (m, 5H), 5.14 (s, 2H), 4.79 (s, 1H), 4.74 (d, J = 36.6 Hz, 1H), 3.52 – 3.42 (m, 4H), 2.43 (dt, J = 23.6, 6.2 Hz, 2H), 2.27 – 2.21 (m, 2H), 1.79 – 1.64 (m, 2H).

^{13}C NMR (126 MHz, CDCl$_3$) (summary of rotamers) δ 155.87, 155.82, 148.46, 148.32, 137.19, 128.55, 128.54, 127.97, 127.94, 127.84, 127.80, 113.23, 113.18, 66.96, 66.94, 48.48, 47.96, 47.08, 46.37, 36.74, 36.31, 34.91, 34.83, 29.03, 28.77.

IR (film) v_{max} 2936, 2851, 1696, 1475, 1420, 1220, 1100, 895, 767, 696 cm^{-1}.

HRMS (ESI-TOF) *m/z* calculated for $C_{15}H_{20}NO_2^+$ ([M+H]$^+$) 246.1494, found 246.1493.

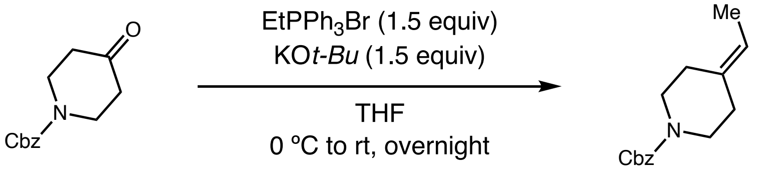

benzyl 4-ethylidenepiperidine-1-carboxylate (S3): The title compound was prepared according to the following procedure. To a flame-dried 250 mL round bottom flask and stir bar under N_2 was added ethyltriphenylphosphonium bromide (1.5 equiv, 19.29 mmol, 7.16 g) and 60 mL of dry THF. The mixture was cooled to 0 ºC and potassium *tert*-butoxide (1.5 equiv, 19.29 mmol, 2.16 g) was added portionwise. The mixture was stirred at this temperature for 5 minutes and then allowed to warm to room temperature while stirring for 1 hour. The mixture was then cooled back to 0 ºC and benzyl 4-oxopiperidine-1-carboxylate (1 equiv, 12.86 mmol, 3.00 g) added dropwise in 60 mL of dry THF. The mixture was stirred at room temperature overnight. The reaction contents were transferred to a separatory funnel with 50 mL of saturated ammonium chloride. This mixture was extracted with DCM (2 x 50 mL). The combined organics were dried over Na_2SO_4 and concentrated. Purification was performed via automated flash chromatography (100 g high

performance silica column, 0-20% ethyl acetate/hexanes). The solvent was removed and the product dried on high-vac to yield the title compound as a clear oil (2.47 g, 10.1 mmol, 79% yield).

^{1}H NMR (500 MHz, CDCl$_3$) δ 7.41 – 7.28 (m, 5H), 5.29 (q, *J* = 6.8 Hz, 1H), 5.15 (s, 2H), 3.47 (t, *J* = 5.9 Hz, 4H), 2.19 (d, *J* = 38.8 Hz, 4H), 1.60 (d, *J* = 6.7 Hz, 3H).

^{13}C NMR (126 MHz, CDCl$_3$) δ 155.41, 137.07, 135.10, 128.60, 128.06, 127.96, 118.41, 67.15, 45.93, 44.89, 35.86, 27.91, 12.77.

IR (film) v_{max} 2905, 2861, 1694, 1425, 1218, 1111, 825, 696 cm^{-1}.

HRMS (ESI-TOF) *m/z* calculated for $C_{15}H_{20}NO_2^+$ ([M+H]$^+$) 246.1489, found 246.1489.

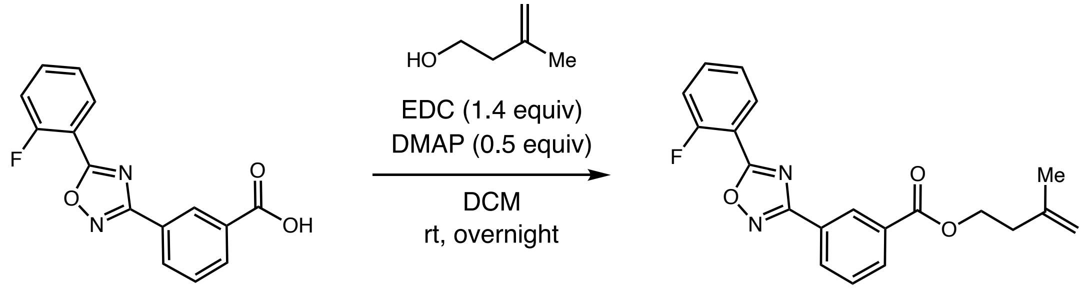

3-methylbut-3-en-1-yl 3-(5-(2-fluorophenyl)-1,2,4-oxadiazol-3-yl)benzoate (S4): The title compound was prepared according to the following procedure. Ataluren (1 equiv, 3.52 mmol, 1.00 g), 1-ethyl-3-(3-dimethylaminopropyl)carbodiimide hydrochloride (1.4 equiv, 4.93 mmol, 944 mg), 4-dimethylaminopyridine (0.5 equiv, 1.76 mmol, 215 mg), and DCM (5 mL) were added to a dry 10 mL round bottom flask with stir bar under N_2. 3-methylbut-3-en-1-ol (1.4 equiv, 4.93 mmol, 424 mg, 499 µL) was added and the reaction stirred at room temperature overnight. The crude reaction mixture was then transferred to a separatory funnel. An additional 50 mL of DCM was added and the solution washed with 50 mL of 1 M HCl. The organic layer was dried over Na_2SO_4 and concentrated. Purification was performed via automated flash chromatography (100 g high performance silica column, 0-10% ethyl acetate/hexanes). The solvent was removed and

the product dried on high-vac to yield the title compound as a white solid (1.04 g, 2.95 mmol, 84% yield).

^{1}H NMR (500 MHz, CDCl$_3$) δ 8.84 (t, *J* = 1.7 Hz, 1H), 8.37 (dt, *J* = 7.7, 1.5 Hz, 1H), 8.27 – 8.22 (m, 1H), 8.20 (dt, *J* = 7.8, 1.5 Hz, 1H), 7.66 – 7.58 (m, 2H), 7.36 (td, *J* = 7.6, 1.1 Hz, 1H), 7.33 – 7.28 (m, 1H), 4.89 – 4.83 (m, 2H), 4.49 (t, *J* = 6.9 Hz, 2H), 2.53 (t, *J* = 6.3 Hz, 2H), 1.84 (s, 3H).

^{13}C NMR (126 MHz, CDCl$_3$) δ 173.19 (d, *J* = 4.4 Hz), 168.27, 165.98, 160.98 (d, *J* = 260.8 Hz), 141.75, 134.88 (d, *J* = 8.6 Hz), 132.35, 131.87, 131.44, 131.13, 129.20, 128.91, 127.39, 124.89 (d, *J* = 3.7 Hz), 117.36 (d, *J* = 21.0 Hz), 112.91 (d, *J* = 11.4 Hz), 112.74, 63.69, 36.99, 22.72.

^{19}F NMR (376 MHz, CDCl$_3$) δ -106.53 – -110.79 (m).

IR (solid) v_{max} 2964, 2872, 1712, 1621, 1554, 1461, 1350, 1269, 1110, 897, 741 cm^{-1}.

HRMS (ESI-TOF) *m/z* calculated for $C_{20}H_{18}FN_2O_3^+$ ([M+H]$^+$) 353.1296, found 353.1301.

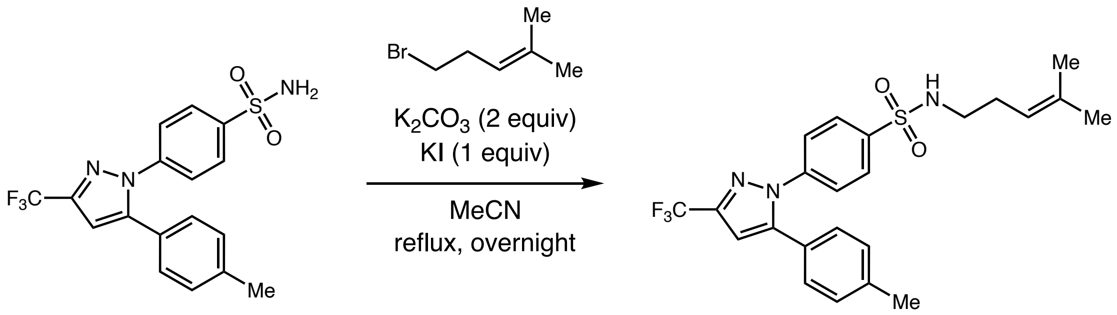

***N*-(4-methylpent-3-en-1-yl)-4-(5-(*p*-tolyl)-3-(trifluoromethyl)-1*H*-pyrazol-1-yl)benzene sulfonamide (S5):** The title compound was prepared according to a modified literature procedure as follows.[4] To a dry 25 mL flask under N_2 was added a stir bar, celecoxib (1 equiv, 0.675 mmol, 257 mg), K_2CO_3 (2 equiv, 1.35 mmol, 187 mg), potassium iodide (1 equiv, 0.675 mmol, 112 mg), and MeCN (5 mL). 5-bromo-2-methylpent-2-ene (1 equiv, 0.675 mmol, 110 mg, 90 µL) was added dropwise and the mixture stirred at reflux overnight. The acetonitrile was then removed *in vacuo* and the resulting residue dissolved in DCM (5 mL) and transferred to a separatory funnel. 25 mL

of water was added and the mixture extracted with DCM (3 x 25 mL). The combined organics were dried over Na_2SO_4 and concentrated. Purification was performed via automated flash chromatography (50 g high performance silica column, 0-30% ethyl acetate/hexanes). The solvent was removed and the product dried on high-vac to yield the title compound as a white solid (191.6 mg, 0.413 mmol, 61% yield).

^{1}H NMR (500 MHz, $CDCl_3$) δ 7.87 – 7.81 (m, 2H), 7.50 – 7.44 (m, 2H), 7.17 (d, *J* = 8.2 Hz, 2H), 7.10 (d, *J* = 8.1 Hz, 2H), 6.74 (s, 1H), 4.92 (ddt, *J* = 7.3, 5.9, 1.5 Hz, 1H), 4.46 – 4.39 (m, 1H), 2.95 (q, *J* = 6.6 Hz, 2H), 2.38 (s, 3H), 2.16 (q, *J* = 7.0 Hz, 2H), 1.68 (s, 3H), 1.58 (s, 3H).

^{13}C NMR (126 MHz, $CDCl_3$) δ 145.39, 144.25 (q, *J* = 38.6 Hz), 142.62, 139.94, 139.63, 136.25, 129.89, 128.86, 128.25, 125.83, 125.72, 121.20 (q, *J* = 269.2 Hz), 119.53, 106.40 (d, *J* = 2.0 Hz), 43.11, 28.28, 25.92, 21.47, 18.05.

^{19}F NMR (376 MHz, $CDCl_3$) δ -62.46.

IR (solid) v_{max} 3247, 2907, 2872, 1597, 1472, 1324, 1233, 1153, 1092, 971, 804, 758 cm^{-1}.

HRMS (ESI-TOF) *m/z* calculated for $C_{23}H_{25}F_3N_3O_2S^+$ ($[M+H]^+$) 464.1614, found 464.1627.

Synthesis and Characterization of Benzoic Acid Substrates:

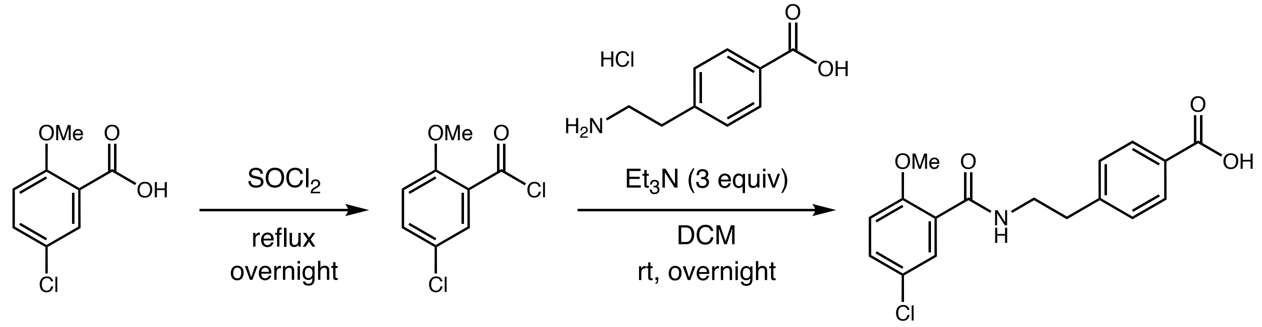

meglitinide (S6): The title compound was prepared according to the following procedure. From a modified literature procedure,[5] 5-chloro-2-methoxybenzoic acid (1 equiv, 30 mmol, 5.60 g) was added to a dry 100 mL flask. Thionyl chloride (10 equiv, 300 mmol, 35.7 g, 21.9 mL) was added dropwise and the mixture stirred at reflux overnight. The reaction was cooled to room temperature and concentrated *in vacuo*. The resulting solids were washed with cold hexanes (3 x 50 mL) and dried on high vac to yield 5-chloro-2-methoxybenzoyl chloride as a white powder (5.21 g, 25.41 mmol, 85% yield). **^{1}H NMR (500 MHz, $CDCl_3$)** δ 8.03 (d, J = 2.7 Hz, 1H), 7.53 (dd, J = 8.9, 2.7 Hz, 1H), 6.95 (d, J = 8.9 Hz, 1H), 3.92 (s, 3H).

To a dry 100 mL flask under N_2 was added 4-(2-aminoethyl)benzoic acid hydrochloride (1 equiv, 3 mmol, 605 mg). Dry DCM (40 mL) was added followed by triethylamine (3 equiv, 9 mmol, 911 mg, 1.25 mL). The mixture was stirred at room temperature for 5 minutes and then 5-chloro-2-methoxybenzoyl chloride (1.1 equiv, 3.3 mmol, 677 mg) added portionwise. The reaction was stirred at room temperature overnight then quenched via addition of 1 M HCl (40 mL) and transferred to a separatory funnel. The mixture was extracted with DCM (3 x 40 mL) and the combined organics dried over Na_2SO_4 and concentrated. Purification was performed via automated flash chromatography (100 g high performance silica column, 0-100% ethyl acetate/hexanes). The solvent was removed and the product dried on high-vac to yield the title compound as a white solid (449.8 mg, 1.35 mmol, 45% yield).

^{1}H NMR (500 MHz, CDCl$_3$) δ 8.18 (d, *J* = 2.8 Hz, 1H), 8.12 – 8.04 (m, 2H), 7.82 (t, *J* = 5.7 Hz, 1H), 7.41 – 7.34 (m, 3H), 6.87 (d, *J* = 8.8 Hz, 1H), 3.81 – 3.76 (m, 5H), 3.02 (t, *J* = 6.8 Hz, 2H).

^{13}C NMR (126 MHz, CDCl$_3$) δ 170.97, 164.17, 156.05, 145.91, 132.52, 132.16, 130.65, 129.26, 127.72, 126.97, 122.87, 112.94, 56.30, 40.78, 35.86.

IR (solid) v_{max} 3383, 3072, 2943, 2849, 1710, 1628, 1542, 1483, 1272, 1178, 1020, 730 cm^{-1}.

HRMS (ESI-TOF) *m/z* calculated for $C_{17}H_{17}ClNO_4^+$ ([M+H]$^+$) 334.0841, found 334.0843.

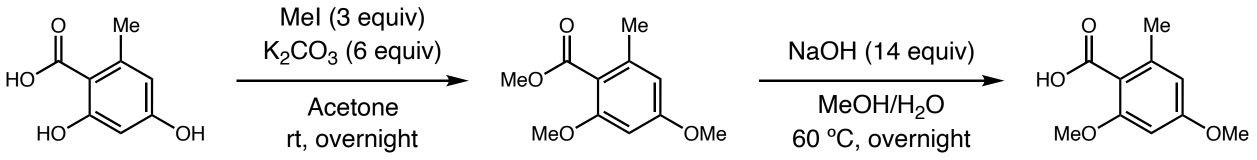

2,4-dimethoxy-6-methylbenzoic acid (orsellinic acid O-Me, S7): The title compound was prepared according to the following procedure. To a dry 50 mL flask was added orsellinic acid (1 equiv, 3 mmol, 504 mg) and K_2CO_3 (6 equiv, 18 mmol, 2.49 g). The flask was put under vacuum and the atmosphere exchanged with N_2 three times. Dry acetone (10 mL) was added and the mixture stirred for 10 minutes under N_2. Iodomethane (3 equiv, 9 mmol, 1.28 g, 560 μL) was added and the mixture stirred overnight. The reaction was quenched via slow addition of H_2O (10 mL) and the contents transferred to a separatory funnel. 1 M HCl (60 mL) was added and the mixture extracted with EtOAc (2 x 60 mL). The combined organics were washed with brine (60 mL) then dried over Na_2SO_4 and concentrated. Purification was performed via automated flash chromatography (50 g high performance silica column, 0-20% ethyl acetate/hexanes). The solvent was removed and the product dried on high-vac to yield methyl 2,4-dimethoxy-6-methylbenzoate as a clear oil (383 mg, 1.82 mmol, 61% yield). **^{1}H NMR (500 MHz, CDCl$_3$)** δ 6.32 – 6.30 (m, 2H), 3.88 (s, 3H), 3.79 (s, 3H), 3.79 (s, 3H), 2.28 (s, 3H).

According to a modified literature procedure,[6] methyl 2,4-dimethoxy-6-methylbenzoate (1 equiv, 1.82 mmol, 383 mg) was dissolved in MeOH (8 mL) in a 40 mL vial. NaOH (14 equiv, 25.5 mmol, 1.02 g) was added as a solution in H_2O (4 M, 6.4 mL) and the mixture heated to 60 ºC overnight. The reaction was cooled to room temperature and transferred to a separatory funnel. Saturated aqueous $NaHCO_3$ (40 mL) was added and the mixture extracted with EtOAc (2 x 40 mL). The aqueous layer was acidified to pH 1 with concentrated HCl and extracted with EtOAc (3 x 40 mL). The combined organics were dried over Na_2SO_4 and concentrated to yield the title compound as a white solid (315 mg, 1.61 mmol, 88% yield).

^{1}H NMR (500 MHz, CDCl$_3$) δ 10.39 (b, 1H), 6.46 (d, *J* = 2.4 Hz, 1H), 6.40 (d, *J* = 2.4 Hz, 1H), 3.97 (s, 3H), 3.85 (s, 3H), 2.60 (s, 3H).

^{13}C NMR (126 MHz, CDCl$_3$) δ 166.68, 162.58, 159.89, 145.63, 111.51, 109.80, 96.79, 56.81, 55.62, 23.36.

IR (solid) v_{max} 2973, 2930, 2848, 1681, 1600, 1304, 1163 cm^{-1}.

HRMS (ESI-TOF) *m/z* calculated for $C_{10}H_{13}O_4^+$ ($[M+H]^+$) 197.0808, found 197.0805.

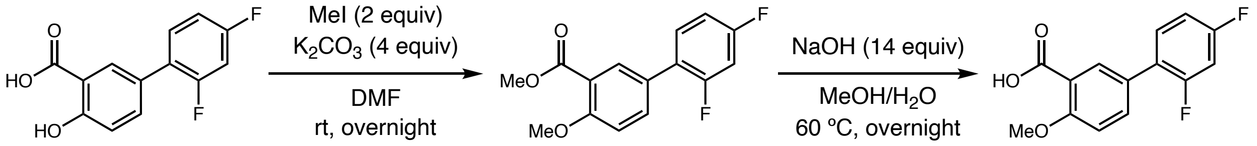

2',4'-difluoro-4-methoxy-[1,1'-biphenyl]-3-carboxylic acid (diflunisal O-Me, S8): The title compound was prepared according to the following procedure. From a modified literature procedure,[7] diflunisal (1 equiv, 4 mmol, 1.00 g) and K_2CO_3 (4 equiv, 16 mmol, 2.21 g) were added to a dry 50 mL flask. The flask was put under vacuum and the atmosphere exchanged with N_2 three times. Dry DMF (10 mL) was added and the mixture stirred for 10 minutes under N_2. Iodomethane (2 equiv, 8 mmol, 1.14 g, 498 μL) was added and the mixture stirred overnight. The reaction was

quenched via slow addition of H_2O (10 mL) and the contents transferred to a separatory funnel. 1 M HCl (60 mL) was added and the mixture extracted with EtOAc (2 x 60 mL). The combined organics were washed with brine (60 mL) then dried over Na_2SO_4 and concentrated. Purification was performed via automated flash chromatography (50 g high performance silica column, 0-30% ethyl acetate/hexanes). The solvent was removed and the product dried on high-vac to yield methyl 2',4'-difluoro-4-methoxy-[1,1'-biphenyl]-3-carboxylate as a white powder (962 mg, 3.46 mmol, 87% yield). **^{1}H NMR (300 MHz, $CDCl_3$)** δ 7.93 (dd, *J* = 2.5, 1.2 Hz, 1H), 7.62 (dt, *J* = 8.8, 2.1 Hz, 1H), 7.38 (td, *J* = 8.8, 6.5 Hz, 1H), 7.05 (d, *J* = 8.7 Hz, 1H), 6.99 – 6.85 (m, 2H), 3.95 (s, 3H), 3.91 (s, 3H).

Methyl 2',4'-difluoro-4-methoxy-[1,1'-biphenyl]-3-carboxylate (1 equiv, 3.46 mmol, 962 mg) was dissolved in MeOH (15 mL) in a 40 mL vial. NaOH (14 equiv, 48.4 mmol, 1.94 g) was added as a solution in H_2O (4 M, 12.1 mL) and the mixture heated to 60 ºC overnight. The reaction was cooled to room temperature and transferred to a separatory funnel. Saturated aqueous $NaHCO_3$ (80 mL) was added and the mixture extracted with EtOAc (2 x 80 mL). The aqueous layer was acidified to pH 1 with concentrated HCl and extracted with EtOAc (3 x 80 mL). The combined organics were dried over Na_2SO_4 and concentrated to yield the title compound as a white solid (764 mg, 2.89 mmol, 84% yield).

^{1}H NMR (500 MHz, $CDCl_3$) δ 10.69 (b, 1H), 8.34 – 8.30 (m, 1H), 7.75 (dt, *J* = 8.7, 2.1 Hz, 1H), 7.45 – 7.37 (m, 1H), 7.15 (d, *J* = 8.7 Hz, 1H), 7.01 – 6.87 (m, 2H), 4.14 (s, 3H).

^{13}C NMR (126 MHz, $CDCl_3$) δ 165.16, 162.64 (dd, *J* = 249.8, 11.9 Hz), 159.84 (dd, *J* = 250.5, 11.8 Hz), 157.65, 135.59 (d, *J* = 4.1 Hz), 134.16 (d, *J* = 2.1 Hz), 131.38 (dd, *J* = 9.5, 4.7 Hz), 129.40 (d, *J* = 1.3 Hz), 123.43 (dd, *J* = 13.5, 3.9 Hz), 117.97, 112.04, 111.87 (d, *J* = 3.8 Hz), 104.65 (dd, *J* = 26.5, 25.3 Hz), 57.08.

^{19}F NMR (376 MHz, $CDCl_3$) δ -110.49 (dt, *J* = 14.6, 7.8 Hz), -113.68 (q, *J* = 9.4 Hz).

IR (solid) v_{max} 3286, 2948, 2848, 1734, 1489, 1274, 1016, 734 cm^{-1}.

HRMS (ESI-TOF) *m/z* calculated for $C_{14}H_{11}F_2O_3^+$ ($[M+H]^+$) 265.0671, found 265.0670.

Synthesis of Alkyl Redox-Active Esters:

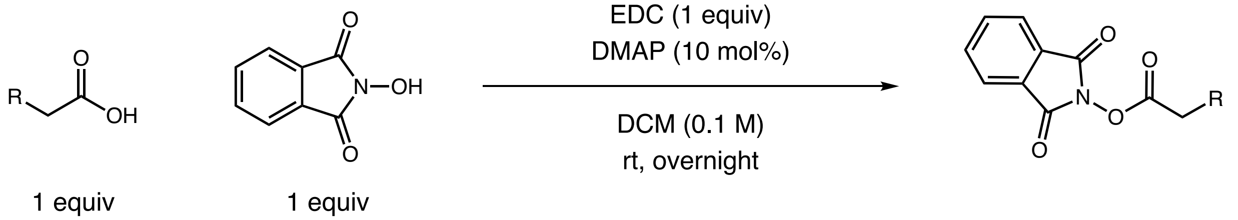

General Procedure A: Synthesis of Alkyl Redox-Active Esters (RAEs)

To a round bottom flask with stir bar under air was added N-hydroxyphthalimide (1 equiv), acid (1 equiv), 1-ethyl-3-(3-dimethylaminopropyl)carbodiimide hydrochloride (1 equiv), and 4-dimethyl aminopyridine (0.1 equiv). DCM (0.1 M) was added and the reaction stirred at room temperature overnight. The reaction mixture was then transferred to a separatory funnel and washed with an equal amount of 1 M HCl. The organic layer was dried over Na_2SO_4 and concentrated. Purification was performed via automated flash chromatography (high performance silica column, either isocratic DCM mobile phase or ethyl acetate/hexanes gradient).

Characterization of Unknown Alkyl Redox-Active Esters:

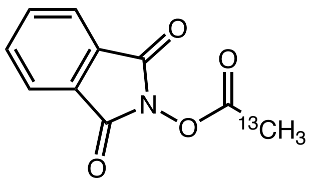

1,3-dioxoisoindolin-2-yl acetate-2-^{13}*C* (S9): The title compound was prepared according to General Procedure A with N-hydroxyphthalimide (1 equiv, 5 mmol, 816 mg), acetic-2-^{13}*C* acid (1 equiv, 5 mmol, 305 mg), 1-ethyl-3-(3-dimethyl aminopropyl)carbodiimide hydrochloride (1 equiv, 5 mmol, 959 mg), 4-dimethylaminopyridine (0.1 equiv, 0.5 mmol, 61.1 mg), and DCM (50 mL). The crude reaction mixture was then transferred to a separatory funnel and washed with 50 mL of

1 M HCl. The organic layer was dried over Na_2SO_4 and concentrated. Purification performed via automated flash chromatography (50 g high performance silica column, 100% DCM isocratic mobile phase). The solvent was removed and the product dried on high-vac to yield the title compound as a white solid (811 mg, 3.90 mmol, 78% yield).

^{1}H NMR (500 MHz, CDCl$_3$) δ 7.89 (dd, J = 5.4, 3.1 Hz, 2H), 7.79 (dd, J = 5.5, 3.1 Hz, 2H), 2.40 (d, J = 131.9 Hz, 3H).

^{13}C NMR (126 MHz, CDCl$_3$) δ 166.69 (d, J = 59.4 Hz), 162.04, 134.92, 129.05, 124.15, 17.78.

IR (solid) v_{max} 2930, 1807, 1785, 1735, 1467, 1364, 1139, 998, 878, 833, 693 cm^{-1}.

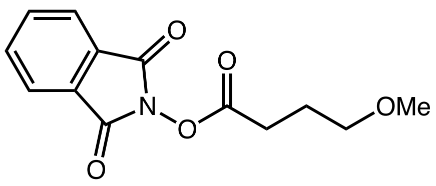

1,3-dioxoisoindolin-2-yl 4-methoxybutanoate (S10): The title compound was prepared according to General Procedure A with N-hydroxyphthalimide (1 equiv, 8.47 mmol, 1.38 g), 4-methoxybutanoic acid (1 equiv, 8.47 mmol, 1.00 g), 1-ethyl-3-(3-dimethyl aminopropyl)carbodiimide hydrochloride (1 equiv, 8.47 mmol, 1.62 g), 4-dimethylaminopyridine (0.1 equiv, 0.847 mmol, 103 mg), and DCM (85 mL). The crude reaction mixture was then transferred to a separatory funnel and washed with 85 mL of 1 M HCl. The organic layer was dried over Na_2SO_4 and concentrated. Purification performed via automated flash chromatography (50 g high performance silica column, 100% DCM isocratic mobile phase). The solvent was removed and the product dried on high-vac to yield the title compound as a white solid (1.50 g, 5.70 mmol, 67% yield).

^{1}H NMR (500 MHz, CDCl$_3$) δ 7.89 (dd, J = 5.5, 3.1 Hz, 2H), 7.79 (dd, J = 5.5, 3.1 Hz, 2H), 3.49 (t, J = 6.0 Hz, 2H), 3.37 (s, 3H), 2.78 (t, J = 7.3 Hz, 2H), 2.04 (tt, J = 7.3, 6.0 Hz, 2H).

^{13}C NMR (126 MHz, $CDCl_3$) δ 169.68, 162.09, 134.88, 129.09, 124.09, 70.85, 58.84, 27.97, 24.95.

IR (solid) v_{max} 2924, 2892, 1807, 1781, 1738, 1467, 1359, 1116, 1041, 877. 857, 690 cm^{-1}.

HRMS (ESI-TOF) *m/z* calculated for $C_{13}H_{13}NNaO_5^+$ ($[M+Na]^+$) 286.0686, found 286.0681.

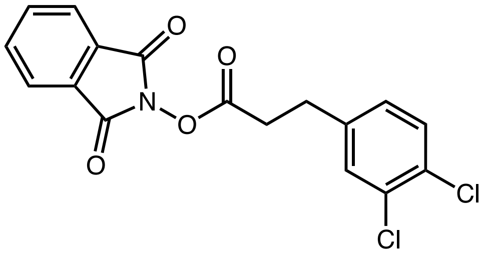

1,3-dioxoisoindolin-2-yl 3-(3,4-dichlorophenyl)propanoate (S11): The title compound was prepared according to General Procedure A with N-hydroxyphthalimide (1 equiv, 10 mmol, 1.63 g), 3-(3,4-dichlorophenyl)propanoic acid (1 equiv, 10 mmol, 2.19 g), 1-ethyl-3-(3-dimethyl aminopropyl)carbodiimide hydrochloride (1 equiv, 10 mmol, 1.92 g), 4-dimethylaminopyridine (0.1 equiv, 1.0 mmol, 122 mg), and DCM (100 mL). The crude reaction mixture was then transferred to a separatory funnel and washed with 100 mL of 1 M HCl. The organic layer was dried over Na_2SO_4 and concentrated. Purification performed via automated flash chromatography (100 g high performance silica column, 100% DCM isocratic mobile phase). The solvent was removed and the product dried on high-vac to yield the title compound as a white solid (2.83 g, 7.77 mmol, 78% yield).

^{1}H NMR (500 MHz, $CDCl_3$) δ 7.90 (dd, *J* = 5.5, 3.1 Hz, 2H), 7.80 (dd, *J* = 5.5, 3.1 Hz, 2H), 7.40 (d, *J* = 8.2 Hz, 1H), 7.37 (d, *J* = 2.1 Hz, 1H), 7.11 (dd, *J* = 8.2, 2.1 Hz, 1H), 3.06 (t, *J* = 7.2 Hz, 2H), 3.00 – 2.95 (m, 2H).

^{13}C NMR (126 MHz, $CDCl_3$) δ 168.59, 161.95, 139.40, 134.99, 132.80, 131.04, 130.81, 130.55, 129.01, 127.97, 124.20, 32.45, 29.79.

IR (solid) v_{max} 2932, 2874, 1809, 1785, 1741, 1467, 1362, 1131, 1080, 961, 864, 693 cm^{-1}.

HRMS (ESI-TOF) *m/z* calculated for $C_{17}H_{11}Cl_2NNaO_4^+$ ($[M+Na]^+$) 385.9957, found 385.9960.

4. Select Reaction Optimization and Control Experiments

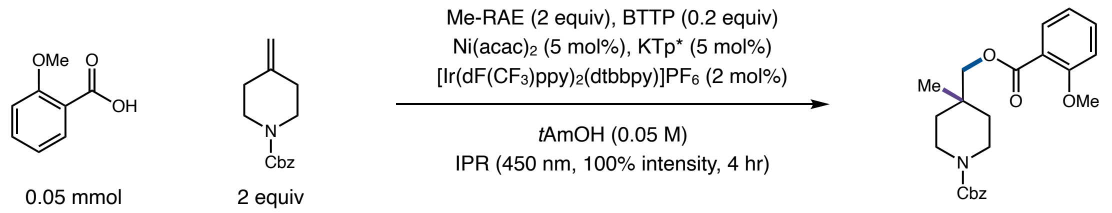

Example Procedure for Reaction Optimization (0.05 mmol scale)

Addition of catalysts via stock solution: (Ir[dF(CF_3)ppy]$_2$(dtbbpy))(PF_6) (2 mol%, 0.001 mmol, 1.1 mg) was added to a dry 8 mL vial equipped with stir bar as a stock solution in acetone (0.001 M, 1 mL stock/vial). Ni(acac)$_2$ (5 mol%, 0.0025 mmol, 0.6 mg) and KTp* (5 mol%, 0.0025 mmol, 0.8 mg) were added to the same vial as a single stock solution in acetone (0.0025 M, 1 mL stock/vial). The acetone was then removed via GeneVac (40 ºC, 1.5 mbar, 15-30 minutes) to leave the catalysts in the reaction vial.

Reaction setup: Under air, Me-RAE (2 equiv, 0.10 mmol, 20.5 mg) was added directly to the prepared vial with catalysts. 2-methoxybenzoic acid (1 equiv, 0.05 mmol, 7.6 mg) was next added as a stock solution in dry *t*-AmOH (0.05 M, 1 mL stock/vial). BTTP (0.2 equiv, 0.01 mmol, 3.1 mg, 3.1 µL) was added via microsyringe. The vial was capped and gently sparged with N_2 for 10 minutes. Alkene (2 equiv, 0.1 mmol, 23.1 mg, 20.8 µL) was added via microsyringe against the flow of N_2. The vial was sealed with parafilm and placed in a PennPhD m2 integrated photoreactor. The reaction was irradiated with 450 nm light for 4 hours (m2 450 nm LED plate, 100% light intensity, 5200 rpm fan speed, 1000 rpm stirring).

Reaction analysis: The solvent was removed via GeneVac and the crude reaction mixture dissolved in 0.5 mL of $CDCl_3$ containing a known amount of mesitylene (~0.05 mmol). The vial was sonicated for 30 seconds and a small aliquot (~15 µL) taken for analysis via ^{1}H NMR (500

mHz) with mesitylene as an internal standard. This procedure was modified as needed to evaluate different variables.

Optimization for Oxymethylation Reaction:

Table S1: Control Reactions

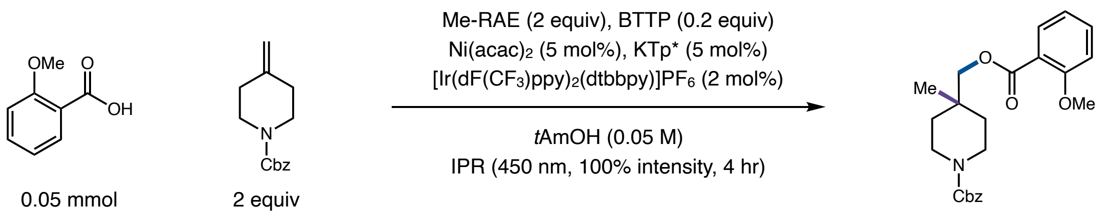

entry	deviation from above	yield
1	none	75%
2	no Ir cat	<1%
3	no $Ni(acac)_2$/KTp*	2%
4	no light	0%
5	no base	40%
6	no sparge	21%

Table S2: Evaluation of Nickel Catalysts at Variable Loadings

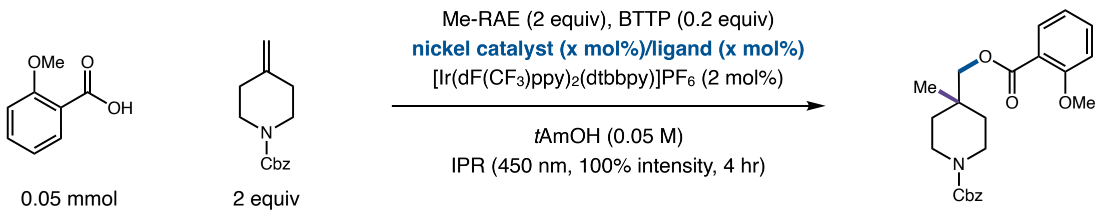

	1 mol%	5 mol%	10 mol%	15 mol%
$Ni(acac)_2$	5%	24%	43%	51%
$Ni(acac)_2$/KTp*	62%	75%	73%	73%
$Ni(acac)_2$/KTp	10%	39%	51%	47%
$Ni(TMHD)_2$	3%	17%	30%	35%

Table S3: Evaluation of Reaction Solvent

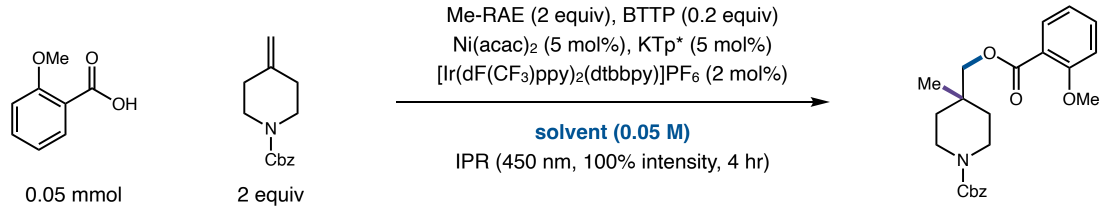

entry	solvent	yield
1	*t*AmOH	75%
2	*t*BuOH	71%
3	MeOH	trace
4	IPA	12%
5	MeCN	41%
6	EtOAc	41%
7	DMC	44%
8	DMA	trace
9	DMSO	28%
10	Acetone	48%
11	DCM	65%

Table S4: Evaluation of Reaction Cosolvents

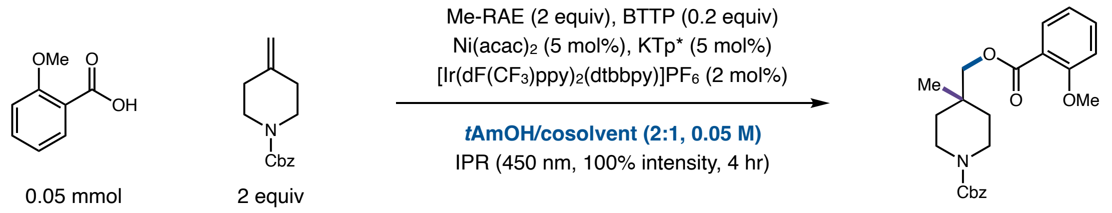

entry	cosolvent	yield
1	MeCN	60%
2	DMC	64%
3	Acetone	65%
4	EtOAc	67%

Table S5: Evaluation of Alkene and Redox-Active Ester Stoichiometry

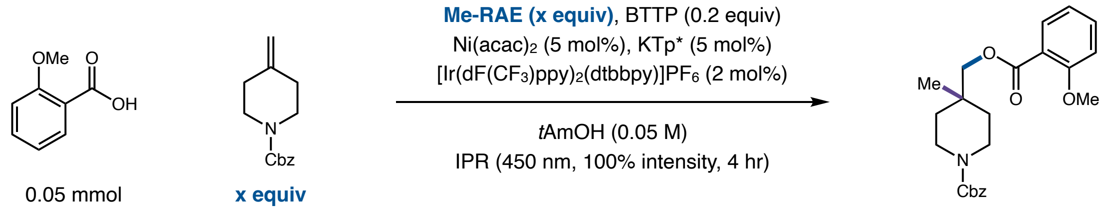

		alkene		
		1 equiv	2 equiv	3 equiv
	1 equiv	34%	45%	55%
Me-RAE	2 equiv	56%	75%	76%
	3 equiv	60%	75%	70%

Table S6: Evaluation of Organic Bases at Variable Loadings

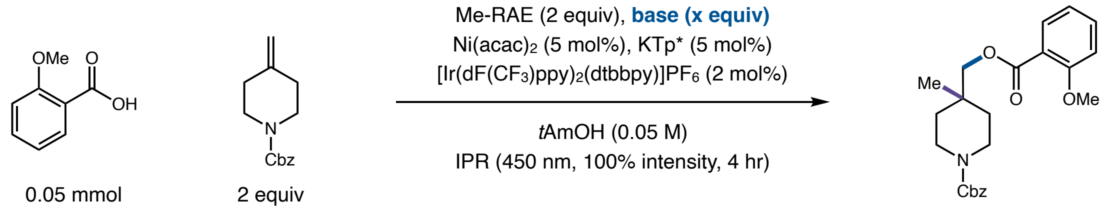

	0.2 equiv	0.5 equiv	0.75 equiv	1 equiv
BTTP	75%	64%	44%	34%
BTMG	72%	73%	65%	39%
BEMP	70%	64%	51%	36%
DBU	67%	64%	57%	44%
MTBD	66%	61%	47%	28%

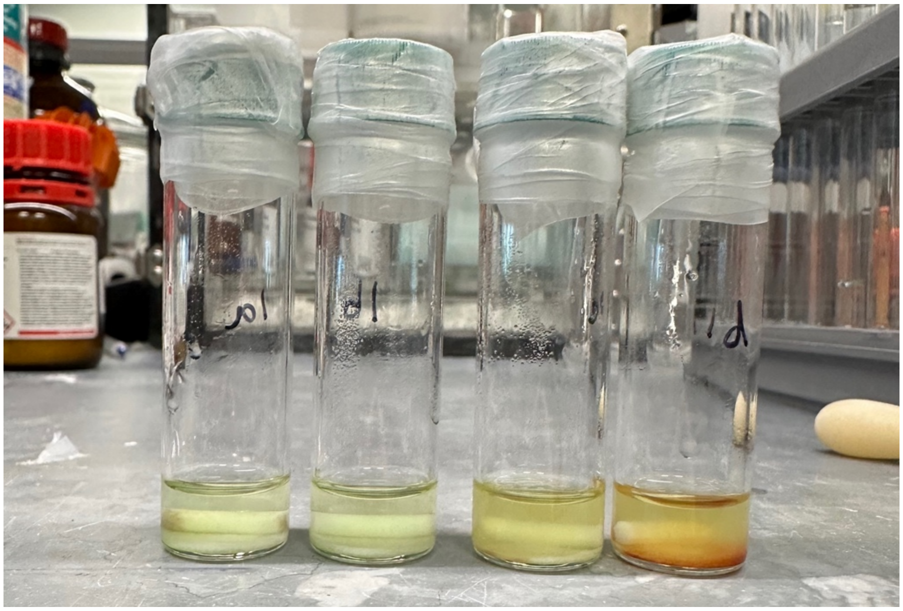

Figure S2: Pre-Irradiation Reactions after Addition of BTTP (from left to right: 0.2 equiv, 0.5 equiv, 0.75 equiv, 1 equiv BTTP)

We were interested to discover that reactions show diminished yield as BTTP loading increases (Table S6). Furthermore, we noticed that using higher equivalencies of BTTP results in a more cloudy solution with a noticeable amount of red precipitate, which forms directly after addition of the base (Figure S2). Subsequent studies combining select components from the reaction in *t*-amyl alcohol (Figure S3) allowed us to identify that base-mediated decomposition of the redox-active ester occurs with BTTP, forming N-hydroxyphthalimide anion (Figure S4, distinctive red color) that was confirmed via UPLC-MS analysis of the mixtures. This phenomenon has been observed for decomposition of similar phthalimide-based reagents by acetate bases.[8] While a small amount of BTTP is necessary to properly initiate the reaction (see control reactions, Table S1), we propose that the fragmentation of the redox-active ester provides additional base *in situ* that can deprotonate the benzoic acid before oxidation (Figure S5).

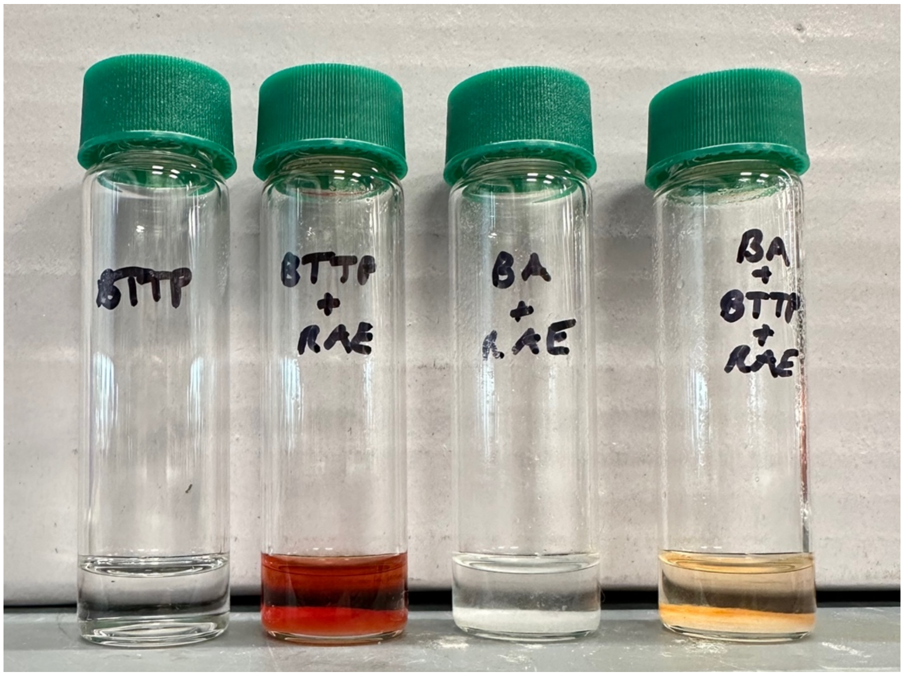

Figure S3: Identification of N-Hydroxyphthalimide Anion with Select Reaction Components (BA = 2-OMe benzoic acid, RAE = methyl redox active ester)

From left to right in Figure S3, N-hydroxyphthalimide anion was observed via UPLC-MS analysis for vial 2 (BTTP + RAE) and vial 4 (BA + BTTP + RAE) only.

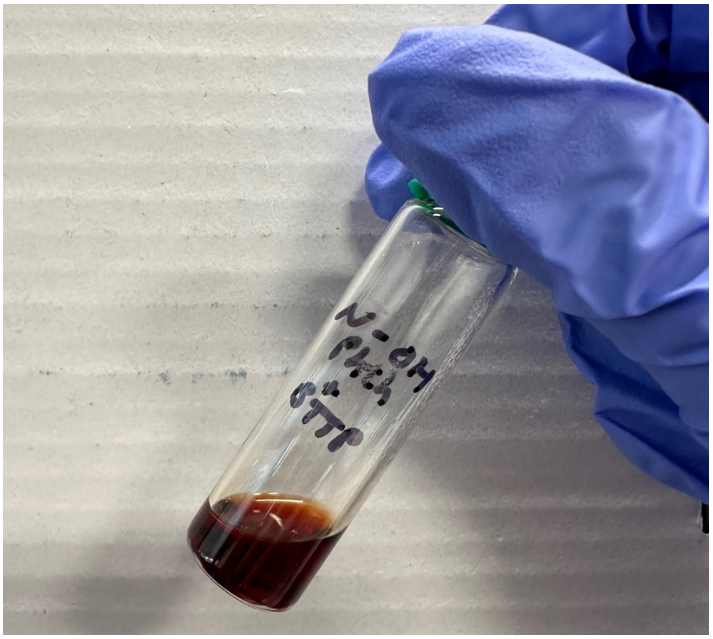

Figure S4: Deprotonation of N-Hydroxyphthalimide

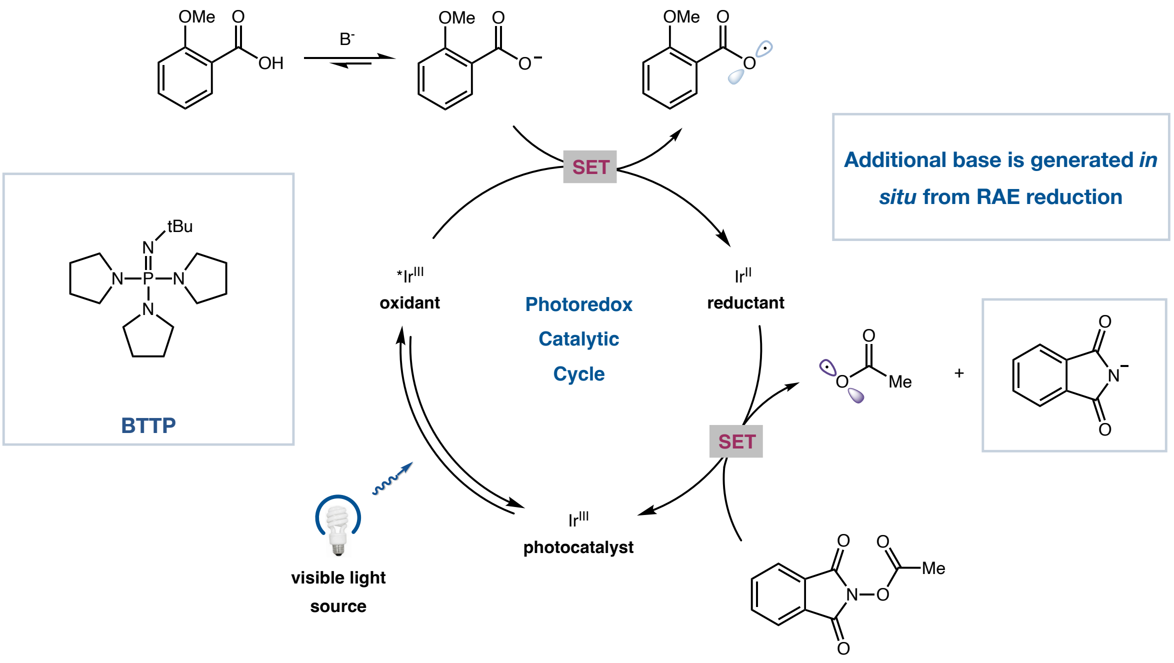

Figure S5: Proposed Mechanism for *in situ* Base Formation

Additional Optimization Performed for Alkyl Chains Beyond Methyl:

Table S7: Evaluation of Nickel Catalysts at Variable Loadings (Phenyl Ether RAE)

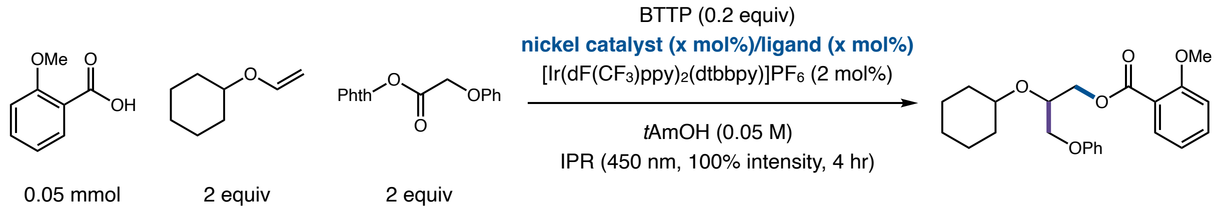

	1 mol%	5 mol%	10 mol%	15 mol%	20 mol%
**Ni(acac)$_2$**	16%	41%	49%	51%	46%
**Ni(acac)$_2$/KTp***	28%	37%	34%	34%	36%
**Ni(acac)$_2$/KTp**	18%	33%	37%	38%	46%
**Ni(TMHD)$_2$**	17%	24%	36%	38%	32%

Table S8: Evaluation of Alkene and Redox-Active Ester Stoichiometry (Phenyl Ether RAE)

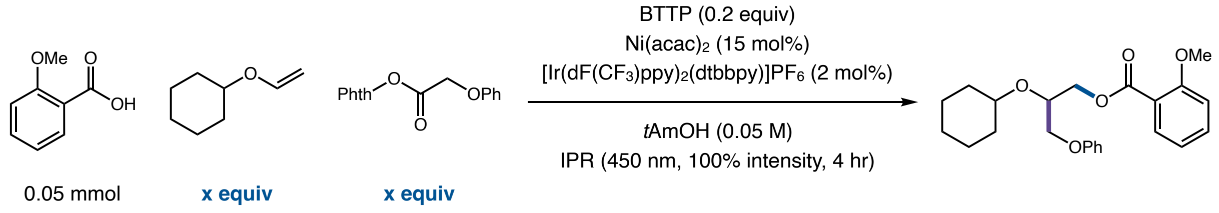

		alkene		
		**1 equiv**	**2 equiv**	**3 equiv**
**RAE**	**1 equiv**	23%	35%	38%
	**2 equiv**	36%	51%	60%
	**3 equiv**	40%	59%	65%

Table S9: Control Reactions (Phenyl Ether RAE)

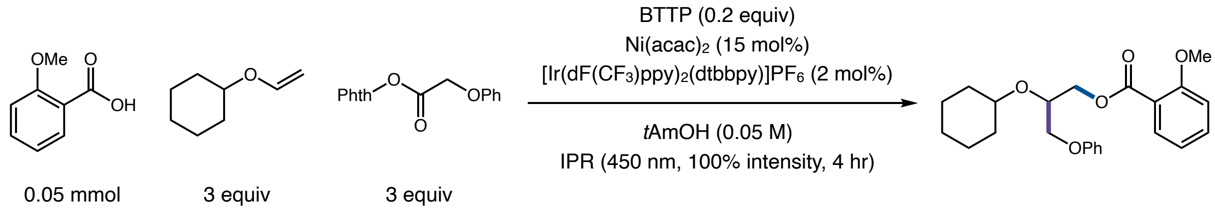

entry	deviation from above	yield
1	none	65%
2	no Ir cat	<1%
3	no $Ni(acac)_2$	27%
4	no light	0%
5	no base	12%

<u>**Additional Optimization Performed for Electron-Deficient Benzoic Acids:**</u>

Table S10: Evaluation of Photocatalysts with Me-RAE

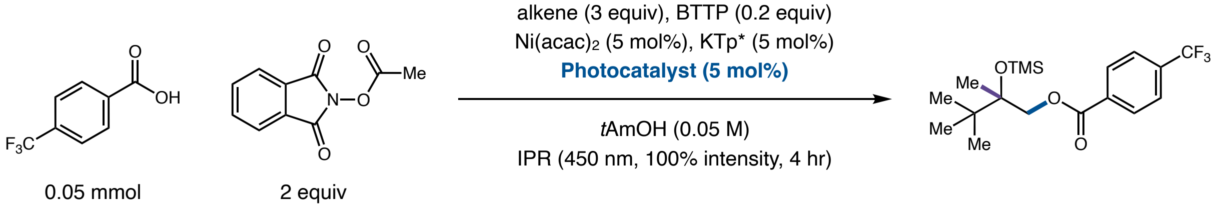

entry	Photocatalyst	yield
1	[Ir(dF(CF$_3$)ppy)$_2$(dtbbpy)]PF$_6$ (2 mol%)	6%
2	4CzIPN	0%
3	4CzIPN-CF$_3$	4%
4	4CzPN	0%
5	4CzIPN-Cl	0%

Table S11: Evaluation of Photocatalysts with Me-RAE-Cl$_4$

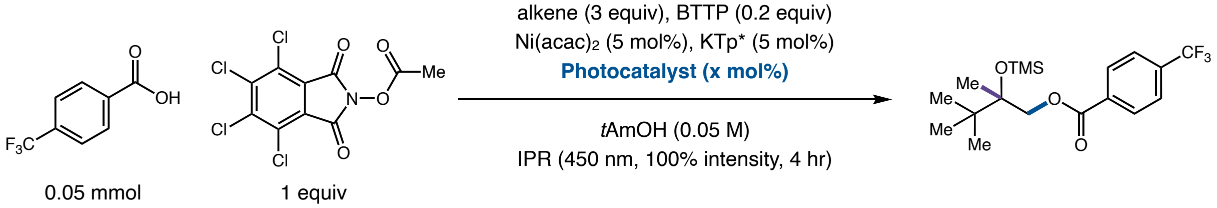

entry	Iridium Photocatalyst (2 mol%)	yield
1	[Ir(dF(CF$_3$)ppy)$_2$(dtbbpy)]PF$_6$	34%
2	[Ir(dFppy)$_2$(phen)]PF$_6$	4%
3	[Ir(dF(CF$_3$)ppy)$_2$(phen)]PF$_6$	14%
4	[Ir(dF(CF$_3$)ppy)$_2$(bpy)]PF$_6$	7%

entry	Organic Photocatalyst (5 mol%)	yield
5	4CzIPN	4%
6	4CzIPN-CF$_3$	11%
7	4CzPN	1%
8	4CzIPN-Cl	2%

*See Table S12 for photocatalyst structures

Table S12: Photocatalyst Structures and Redox Potentials

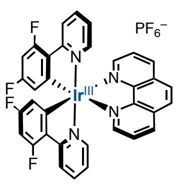

$[Ir(dFppy)_2(phen)]PF_6$

$E_{1/2}(^*PC/PC^-)$ = +1.42 V

$E_{1/2}(PC/PC^-)$ = -1.15 V

$E_{1/2}(PC^+/^*PC)$ = -1.22 V

$E_{1/2}(PC^+/PC)$ = +1.35 V

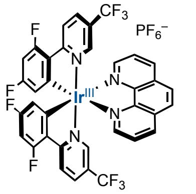

$[Ir(dF(CF_3)ppy)_2(phen)]PF_6$

$E_{1/2}(^*PC/PC^-)$ = +1.33 V

$E_{1/2}(PC/PC^-)$ = -1.22 V

$E_{1/2}(PC^+/^*PC)$ = -0.99 V

$E_{1/2}(PC^+/PC)$ = +1.56 V

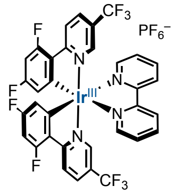

$[Ir(dF(CF_3)ppy)_2(bpy)]PF_6$

$E_{1/2}(^*PC/PC^-)$ = +1.32 V

$E_{1/2}(PC/PC^-)$ = -1.37 V

$E_{1/2}(PC^+/^*PC)$ = -1.00 V

$E_{1/2}(PC^+/PC)$ = +1.69 V

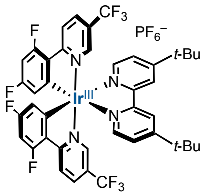

$[Ir(dF(CF_3)ppy)_2(dtbbpy)]PF_6$

$E_{1/2}(^*PC/PC^-)$ = +1.21 V

$E_{1/2}(PC/PC^-)$ = -1.37 V

$E_{1/2}(PC^+/^*PC)$ = -0.89 V

$E_{1/2}(PC^+/PC)$ = +1.69 V

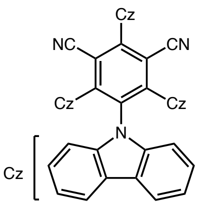

4CzIPN

$E_{1/2}(^*PC/PC^-)$ = +1.35 V

$E_{1/2}(PC/PC^-)$ = -1.21 V

$E_{1/2}(PC^+/^*PC)$ = -1.04 V

$E_{1/2}(PC^+/PC)$ = +1.52 V

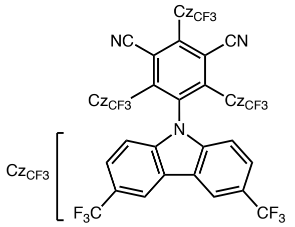

4CzIPN-CF_3

$E_{1/2}(^*PC/PC^-)$ = +1.91 V

$E_{1/2}(PC/PC^-)$ = -0.91 V

$E_{1/2}(PC^+/^*PC)$ = -1.14 V

$E_{1/2}(PC^+/PC)$ = +1.68 V

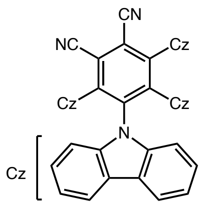

4CzPN

$E_{1/2}(^*PC/PC^-)$ = +1.39 V

$E_{1/2}(PC/PC^-)$ = -1.11 V

$E_{1/2}(PC^+/^*PC)$ = -1.14 V

$E_{1/2}(PC^+/PC)$ = +1.36 V

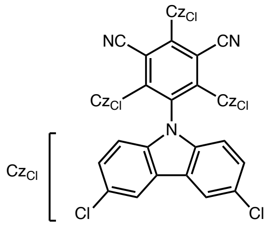

4CzIPN-Cl

$E_{1/2}(^*PC/PC^-)$ = +1.49 V

$E_{1/2}(PC/PC^-)$ = -1.10 V

$E_{1/2}(PC^+/^*PC)$ = -0.72 V

$E_{1/2}(PC^+/PC)$ = +1.87 V

Literature values for redox potentials are reported vs SCE.[9-11] Potentials are in MeCN except for as follows: potentials for 4CzPN are in THF and 4CzIPN-Cl are in DCM.

Due to the higher oxidation potentials of benzoic acids bearing electron-deficient substituents, different photocatalysts were screened to find more oxidizing conditions that could enable product formation. Crucial to this optimization was the use of a tetra-chlorinated redox active ester (Me-RAE-Cl_4), which is more easily reduced than the standard Me-RAE. The three photocatalysts with the highest reactivity of those screened in Table S11 were taken for further optimization as shown below.

Table S13: Evaluation of Photocatalyst Loading for 4CzIPN-CF_3

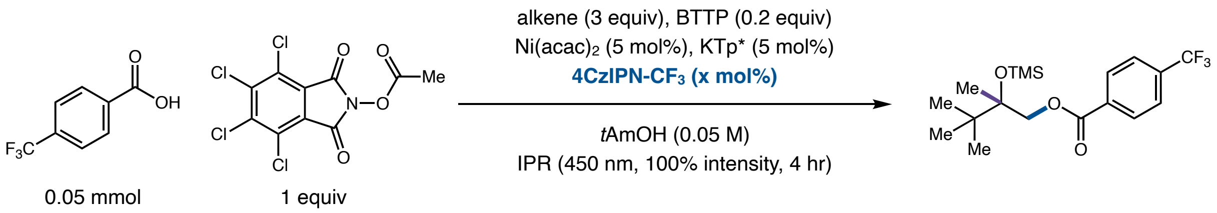

entry	Photocatalyst Loading	yield
1	2 mol%	5%
2	5 mol%	11%
3	10 mol%	20%
4	15 mol%	19%

Table S14: Evaluation of Solvents for 4CzIPN-CF_3 and [Ir(dFCF$_3$ppy)$_2$(phen)]PF$_6$

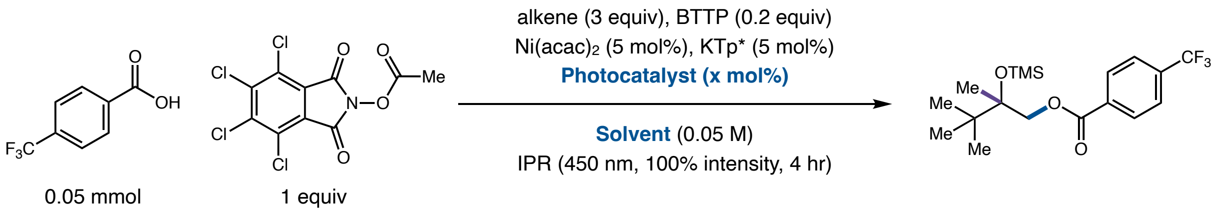

	4CzIPN-CF_3 (10 mol%)	[Ir(dF(CF_3)ppy)$_2$(phen)]PF$_6$ (2 mol%)
*t*AmOH	20%	14%
EtOAc	18%	6%
DMC	26%	6%
MeCN	8%	15%
DCM	18%	8%
Acetone	14%	21%

Table S15: Evaluation of Photocatalyst Loading for [Ir(dFCF3ppy)2(dtbbpy)]PF6

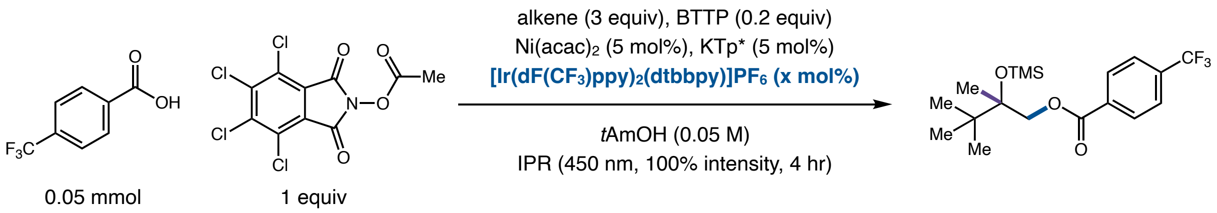

entry	Photocatalyst Loading	yield
1	1.5 mol%	34%
2	2 mol%	34%
3	2.5 mol%	38%

Table S16: Evaluation of Solvents for [Ir(dFCF3ppy)2(dtbbpy)]PF6

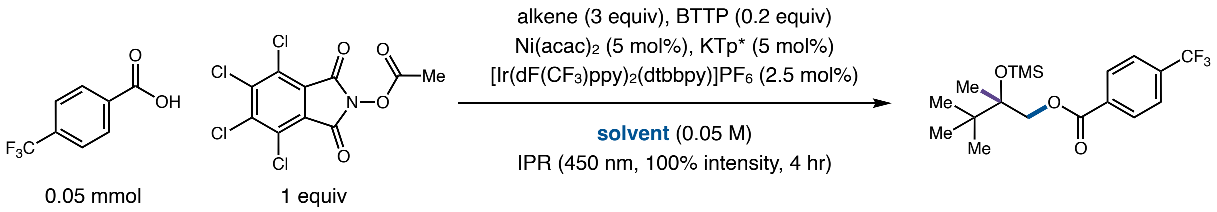

entry	Solvent	yield
1	tAmOH	38%
2	EtOAc	22%
3	DMC	16%
4	MeCN	15%
5	DCM	21%
6	Acetone	36%

Table S17: Evaluation of Me-RAE-Cl_4 Loading for $[Ir(dFCF_3ppy)_2(dtbbpy)]PF_6$

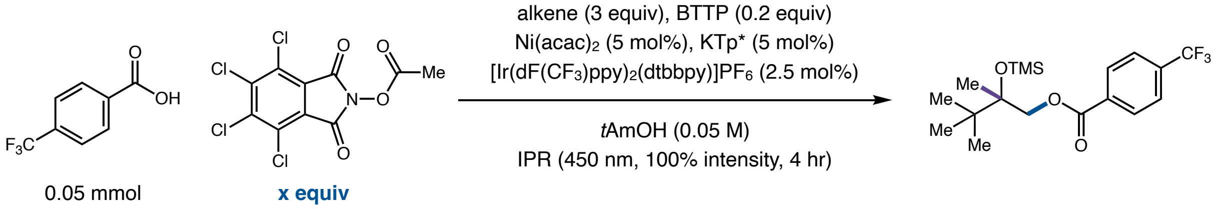

entry	Me-RAE-Cl_4 Loading	yield
1	1 equiv	38%
2	2 equiv	46%
3	3 equiv	46%

As shown in Table S18 of the subsequent section, the new conditions from Table S17 entry 2 (Conditions C below) proved compatible with a selection of highly electron-deficient acids (**S20-S24**) that were unreactive or low-yielding under the general reaction parameters (Conditions B). We were further pleased to see an increase in reactivity for weakly electron-deficient acids (**S25-S27**) as well as the incorporation of electron-deficient heteroaryl acids (**S28-S31**) in low but synthetically useful yields.

5. Extended Scope

Table S18: Additional (Hetero)Aryl Acid Scope

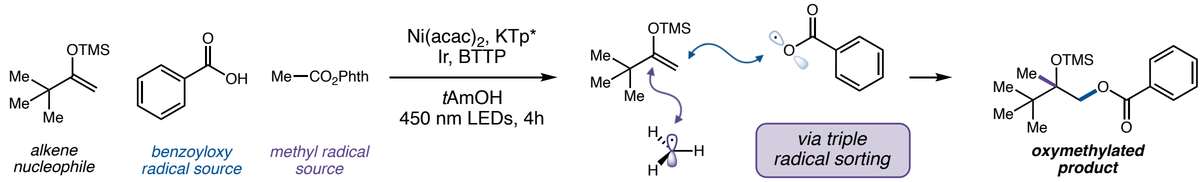

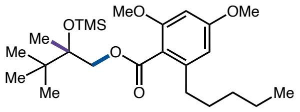

(±)-**S12**, 85% yield[a]
from olivetolic acid O-Me

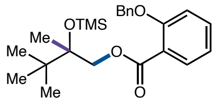

(±)-**S13**, 41% yield[a]

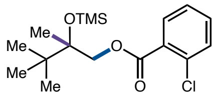

(±)-**S14**, 71% yield[a]

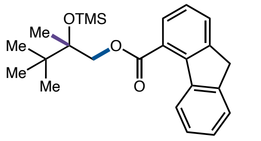

(±)-**S15**, 47% yield[a]

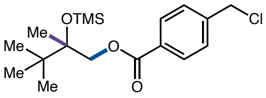

(±)-**S16**, 46% yield[b]

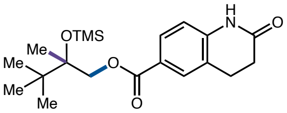

(±)-**S17**, 36% yield[b]

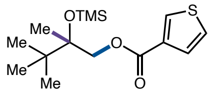

(±)-**S18**, 45% yield[b]

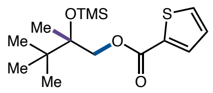

(±)-**S19**, 19% yield[b]

Electron-Deficient Acids

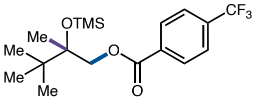

(±)-**S20**,
Conditions B: 6% yield[b]
Conditions C: 46% yield[c]

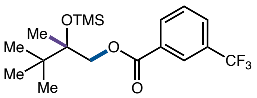

(±)-**S21**,
Conditions B: 17% yield[b]
Conditions C: 43% yield[c]

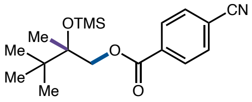

(±)-**S22**,
Conditions B: 2% yield[b]
Conditions C: 42% yield[c]

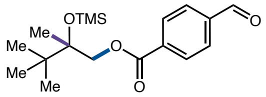

(±)-**S23**,
Conditions B: 4% yield[b]
Conditions C: 47% yield[c]

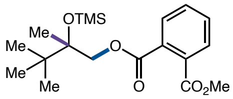

(±)-**S24**,
Conditions B: 11% yield[b]
Conditions C: 29% yield[c]

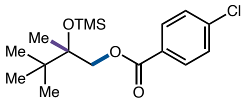

(±)-**S25**,
Conditions B: 28% yield[b]
Conditions C: 48% yield[c]

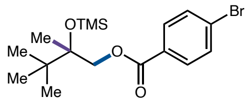

(±)-**S26**,
Conditions B: 23% yield[b]
Conditions C: 47% yield[c]

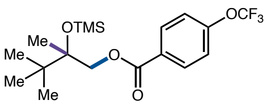

(±)-**S27**,
Conditions B: 27% yield[b]
Conditions C: 52% yield[c]

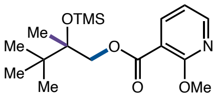

(±)-**S28**,
Conditions B: 16% yield[b]
Conditions C: 48% yield[c]

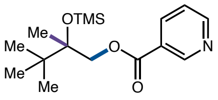

(±)-**S29**,
Conditions B: 2% yield[b]
Conditions C: 24% yield[c]

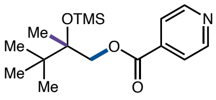

(±)-**S30**,
Conditions B: 1% yield[b]
Conditions C: 9% yield[c]

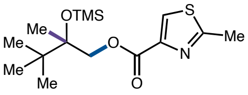

(±)-**S31**,
Conditions B: 5% yield[b]
Conditions C: 24% yield[c]

Unsuccessful Substrates

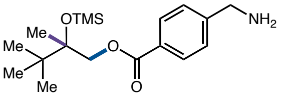

(±)-**S32**
(free amine decomposes RAE)

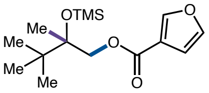

(±)-**S33**

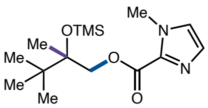

(±)-**S34**

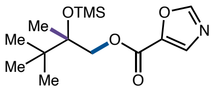

(±)-**S35**

All reactions performed on 0.05 mmol scale with yields determined via ^{1}H NMR. [a]With benzoic acid (1 equiv), Me-RAE (2 equiv), alkene (2 equiv), Ni(acac)$_2$ (5 mol%), KTp* (5 mol%), [Ir(dF(CF$_3$)ppy)$_2$(dtbbpy)]PF$_6$ (2 mol%), BTTP (0.2 equiv), *t*-AmOH (0.05 M), integrated photoreactor (450 nm, 100% intensity), 4 h. [b]With 3 equiv alkene. [c]With 3 equiv alkene, 2.5 mol% [Ir(dF(CF$_3$)ppy)$_2$(dtbbpy)]PF$_6$, and 2 equiv Me-RAE-Cl$_4$.

Table S19: Additional Alkene Scope[a]

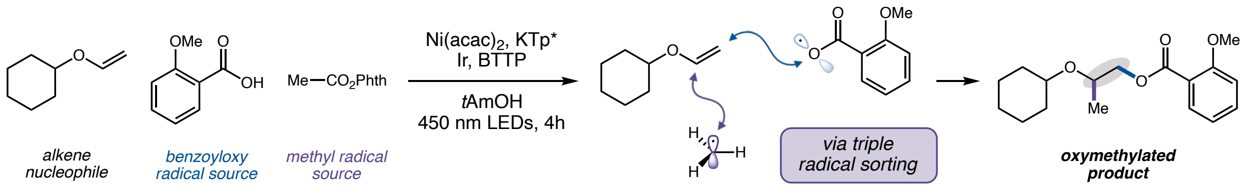

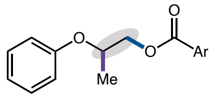

(±)-**S36**, 77% yield[b]

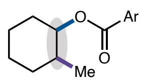

(±)-**S37**, 46% yield[b]
1:1.4 d.r.

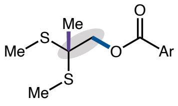

S38, 33% yield[b]

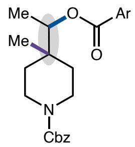

(±)-**S39**, 79% yield

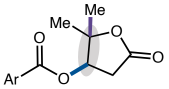

(±)-**S40**, 52% yield

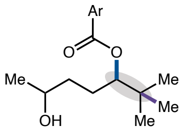

(±)-**S41**, 53% yield[c]

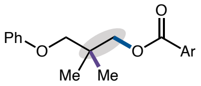

S42, 30% yield

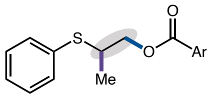

(±)-**S43**, 32% yield

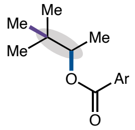

(±)-**S44**, 29% yield[b]

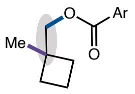

S45, 34% yield

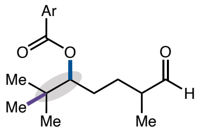

(±)-**S46**, 26% yield
1:1.4 d.r.

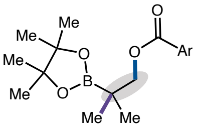

S47, 28% yield

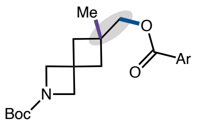

S48, 30% yield

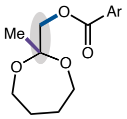

S49, 27% yield

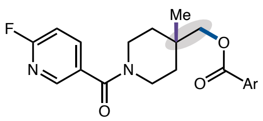

S50, 41% yield

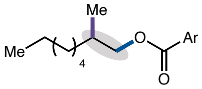

(±)-**S51,** 28% yield

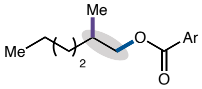

(±)-**S52**, 28% yield

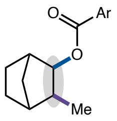

(±)-**S53**, 30% yield
1:1.8 d.r.

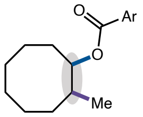

(±)-**S54**, 19% yield[c]

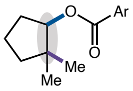

(±)-**S55**, 47% yield

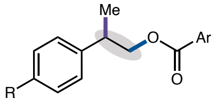

R = OMe
(±)-**S56**, 55% yield

R = Me
(±)-**S57**, 45% yield

R = H
(±)-**S58**, 41% yield

R = F
(±)-**S59**, 45% yield

R = CF_3
(±)-**S60**, 19% yield

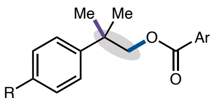

R = OMe
S61, 57% yield

R = Me
S62, 45% yield

R = H
S63, 43% yield

R = F
S64, 35% yield

R = CF_3
S65, 23% yield

[a]Performed on 0.05 mmol scale with 2-methoxybenzoic acid (1 equiv), Me-RAE (2 equiv), alkene (2 equiv), Ni(acac)$_2$ (5 mol%), KTp* (5 mol%), [Ir(dF(CF$_3$)ppy)$_2$(dtbbpy)]PF$_6$ (2 mol%), BTTP (0.2 equiv), *t*-AmOH (0.05 M), integrated photoreactor (450 nm, 100% intensity), 4 h. Yields determined via ^{1}H NMR or UPLC assay unless otherwise noted. Ar = 2-methoxybenzene. [b]0.5 mmol scale, isolated yield. [c]The d.r. of the crude reaction mixture could not be determined due to peak overlap.

Table S20: Additional Pharmaceuticals and Natural Products and Unsuccessful Substrates[a]

Complex Examples

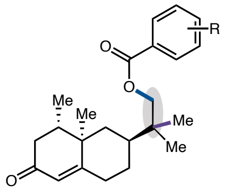

from (+)-nootkatone

2-OMe benzoic acid: **S66**, 77% yield
4-OMe benzoic acid: **S67**, 67% yield
2-Br benzoic acid: **S68**, 52% yield
4-Me benzoic acid: **S69**, 35% yield

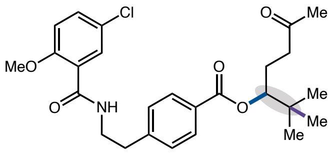

(±)-**S70**, 39% yield[b]
from meglitinide

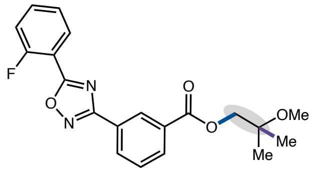

S71, 21% yield[b]
from ataluren

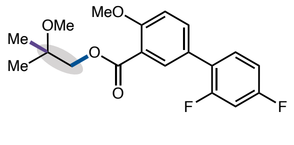

S72, 80% yield[b]
from diflunisal O-Me

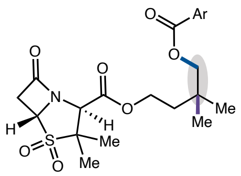

S73, 46% yield
from sulbactam core

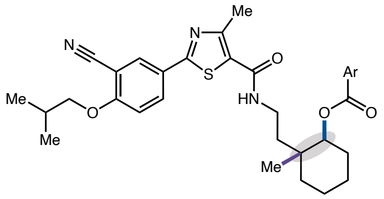

(±)-**S74**, 22% yield[c]
from febuxostat core

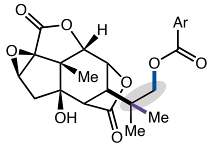

S75, 25% yield
from picrotoxinin

Unsuccessful Substrates

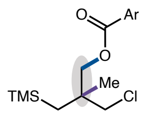

(±)-**S76**

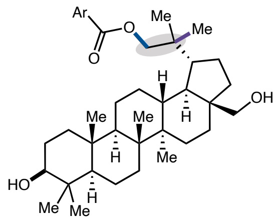

S77, *from betulin*
(alkene insoluble)

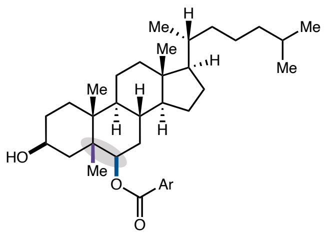

(±)-**S78**, *from cholesterol*
(alkene insoluble)

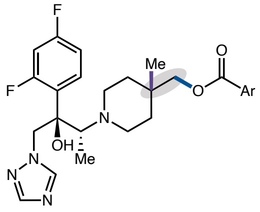

S79, *from efinaconazole*
(oxidizable amine)

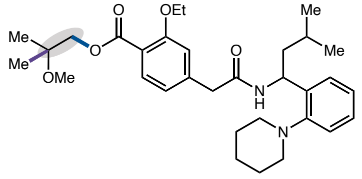

(±)-**S80**[b]
from repaglinide
(oxidizable amine)

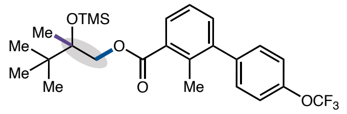

(±)-**S81**, <5% yield
from Sonidegib metabolite m48

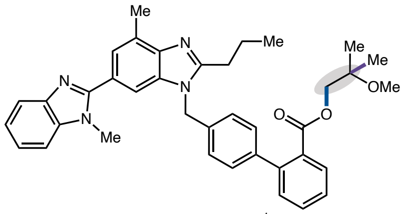

S82, trace yield[b]
from telmisartan

All reactions performed on 0.05 mmol scale with yields determined via ^{1}H NMR. [a]With benzoic acid (1 equiv), Me-RAE (2 equiv), alkene (2 equiv), Ni(acac)$_2$ (5 mol%), KTp* (5 mol%), [Ir(dF(CF$_3$)ppy)$_2$(dtbbpy)]PF$_6$ (2 mol%), BTTP (0.2 equiv), *t*-AmOH (0.05 M), integrated photoreactor (450 nm, 100% intensity), 4 h. [b]With 3 equiv alkene. [c]The d.r. of the crude reaction mixture could not be determined due to peak overlap. Ar = 2-methoxybenzene.

Table S21: Additional Alkylation Scope[a]

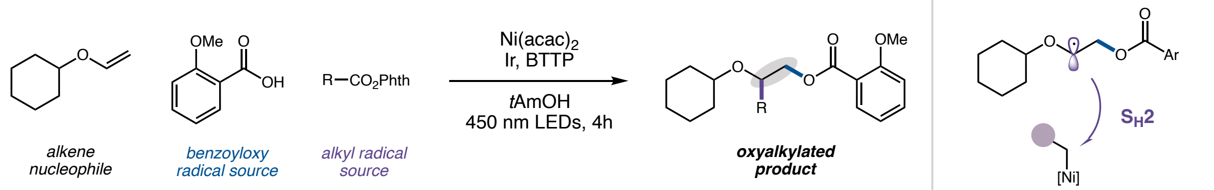

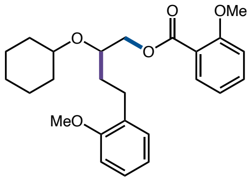

(±)-**S83**, 40% yield[b]

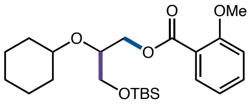

(±)-**S84**, 41% yield

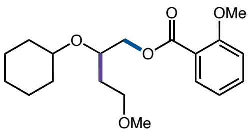

(±)-**S85**, 38% yield

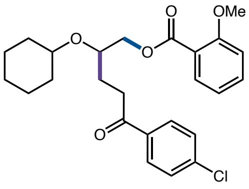

(±)-**S86**, 22% yield

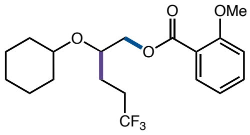

(±)-**S87**, 18% yield

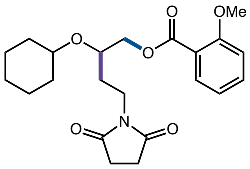

(±)-**S88**, 18% yield

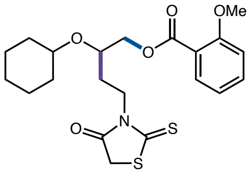

(±)-**S89**, 0% yield
(RAE fully recovered)

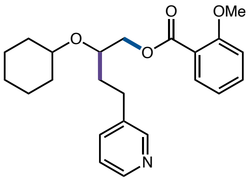

(±)-**S90**, <5% yield

[a]Performed on 0.05 mmol scale with 2-methoxybenzoic acid (1 equiv), RAE (3 equiv), alkene (3 equiv), Ni(acac)$_2$ (15 mol%), [Ir(dF(CF$_3$)ppy)$_2$(dtbbpy)]PF$_6$ (2 mol%), BTTP (0.2 equiv), *t*-AmOH (0.05 M), integrated photoreactor (450 nm, 100% intensity), 4 h. Yields determined via ^{1}H NMR unless otherwise noted. [b]0.5 mmol scale, isolated yield.

Additional benzoic acid substrates were evaluated under the reaction conditions and found to provide product. Yields for these examples were determined by ^{1}H NMR vs mesitylene or 1,3,5-trimethoxybenzene as an internal standard, and are noted as assay yields in Table S18. For electron-deficient benzoic acids, the standard reaction conditions (Conditions B) were employed along with the alternative conditions (Conditions C) from Table S17 entry 2 in section 4. While these new conditions extend the reaction scope to more challenging benzoic acids and heteroaryl acids, unsuccessful substrates including those with free amine nucleophiles (**S32**) and select heterocycles (**S33-S35**) are also shown. Additional alkenes were also evaluated and listed in Table S19. In general, diminished but synthetically useful yields were obtained for unactivated, monosubstituted alkenes (such as **S51** and **S52**) as well as strained alkenes such as norbornene (**S53**), which we

attribute to a slower addition of the oxygen-centered radical due to a reduced nucleophilicity of the alkene. Finally, additional complex examples and alkylation examples are also provided in Tables S20 and S21. Substrates that did not undergo efficient oxyalkylation are also listed in Tables S20 and S21. In some cases, this was due to insolubility of the alkene in *t*-amyl alcohol (steroid derivatives **S79** and **S80**) or an oxidizable functionality that would compete with benzoate oxidation (the tertiary amine in **S81** and **S82**).

6. Mechanistic Experiments

TEMPO Experiments:

Table S22: TEMPO Addition to Oxymethylation Reaction

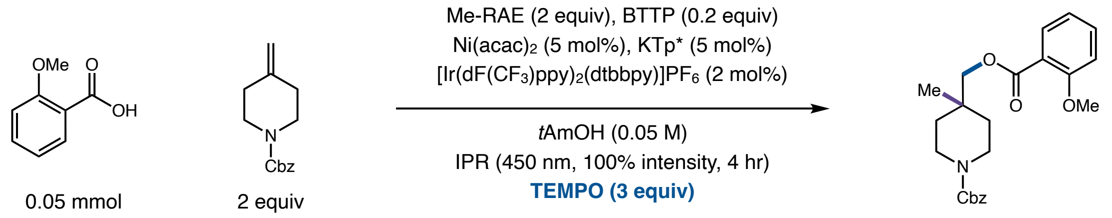

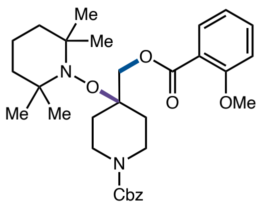

S91, $C_{31}H_{42}N_2NaO_6^+$ ($[M+Na]^+$) calc. 561.2935, found 561.2940

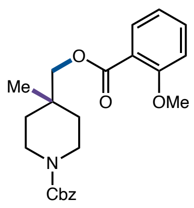

not detected

Addition of TEMPO at the start of the above reaction prevented product formation (Table S22), consistent with a radical-based pathway. Using high-resolution mass spectrometry, we identified the TEMPO adduct of the post-addition radical (**S91**), which is consistent with the benzoyloxy radical undergoing addition to the alkene; the resultant tertiary alkyl radical recombines with TEMPO before the subsequent methylation can occur to form the oxyalkylated product.

Procedure for Table S22: To a dry 40 mL vial equipped with X-shaped stir bar was added $Ni(acac)_2$ (5 mol%, 25 μmol, 6.4 mg), KTp* (5 mol%, 25 μmol, 8.4 mg), $(Ir[dF(CF_3)ppy]_2(dtbbpy))(PF_6)$ (2 mol%, 10 μmol, 11.2 mg), (1,3-dioxoisoindolin-2-yl)acetate (2 equiv, 1 mmol, 205.2 mg), 2-methoxybenzoic acid (1 equiv, 0.5 mmol, 76.1 mg), and TEMPO (3 equiv, 1.5 mmol, 234.4 mg). 10 mL of dry *t*-amyl alcohol was added followed by addition of BTTP (0.2 equiv, 0.1 mmol, 31.2 mg, 30.6 μL) via microsyringe. The vial was capped and gently sparged with N_2 for 15 minutes.

Benzyl 4-methylenepiperidine-1-carboxylate (2 equiv, 1 mmol, 231.3 mg, 208.4 µL) was added via microsyringe against the flow of N_2. The vial was sealed with parafilm and electrical tape then placed in a PennPhD m2 integrated photoreactor. The reaction was irradiated with 450 nm light for 4 hours (m2 450 nm LED plate, 100% light intensity, 5200 rpm fan speed, 1000 rpm stirring). The solvent was removed via GeneVac and the crude reaction mixture analyzed via ^{1}H NMR (500 mHz) and UPLC-MS to detect product formation. The TEMPO adduct was isolated via automated reverse-phase chromatography (25 g C18 column, 20-100% gradient of 0.1% ammonium hydroxide in water/0.1% ammonium hydroxide in acetonitrile) followed by additional purification via Preparative HPLC (XBridge BEH C18 OBD column, 40-100% gradient of 0.1% ammonium hydroxide in water/0.1% ammonium hydroxide in acetonitrile) and then characterized by HRMS (ESI-TOF).

Table S23: TEMPO Addition to Oxyalkylation Reaction

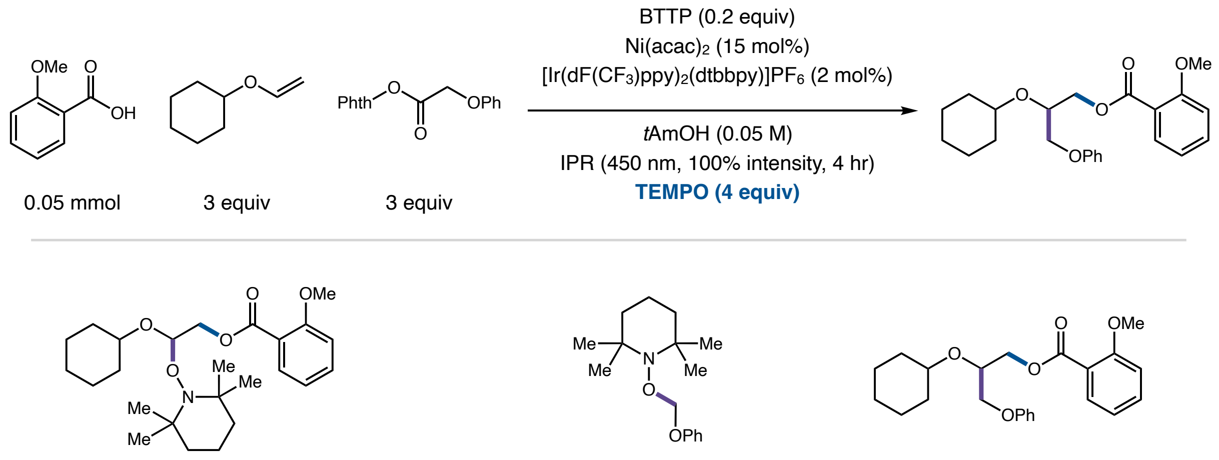

S92, $C_{25}H_{40}NO_5^+$ ([M+H]+) calc. 434.2901, found 434.2905

S93, $C_{16}H_{26}NO_2^+$ ([M+H]+) calc. 264.1958, found 264.1963

not detected

The addition of TEMPO at the beginning of this oxyalkylation reaction resulted in no product formation (Table S23). We identified TEMPO adduct **S92**, presumably arising from trapping of

the secondary radical formed from addition of a benzoyloxy radical into the alkene. We also isolated the TEMPO adduct of the primary alkyl chain (**S93**), indicating decomposition of the redox-active ester to form a primary alkyl radical.

Procedure for Table S23: To a dry 40 mL vial equipped with X-shaped stir bar was added Ni(acac)$_2$ (15 mol%, 75 μmol, 19.3 mg), (Ir[dF(CF$_3$)ppy]$_2$(dtbbpy))(PF$_6$) (2 mol%, 10 μmol, 11.2 mg), 1,3-dioxoisoindolin-2-yl 2-phenoxyacetate (3 equiv, 1.5 mmol, 445.9 mg), 2-methoxybenzoic acid (1 equiv, 0.5 mmol, 76.1 mg), and TEMPO (4 equiv, 2 mmol, 312.5 mg). 10 mL of dry *t*-amyl alcohol was added followed by addition of *tert*-butylimino-tri(pyrrolidino)phosphorane (0.2 equiv, 0.1 mmol, 31.2 mg, 30.6 μL) via microsyringe. The vial was capped and gently sparged with N_2 for 15 minutes. (Vinyloxy)cyclohexane (3 equiv, 1.5 mmol, 189.3 mg, 212.5 μL) was added via microsyringe against the flow of N_2. The vial was sealed with parafilm and electrical tape then placed in a PennPhD m2 integrated photoreactor. The reaction was irradiated with 450 nm light for 4 hours (m2 450 nm LED plate, 100% light intensity, 5200 rpm fan speed, 1000 rpm stirring). The solvent was removed via GeneVac and the crude reaction mixture analyzed via ^{1}H NMR (500 mHz) and UPLC-MS to detect product formation. The TEMPO adducts were isolated via automated reverse-phase chromatography (25 g C18 column, 20-100% gradient of 0.1% ammonium hydroxide in water/0.1% ammonium hydroxide in acetonitrile) followed by additional purification via Preparative HPLC (XBridge BEH C18 OBD column, 40-100% gradient of 0.1% ammonium hydroxide in water/0.1% ammonium hydroxide in acetonitrile) and then characterized by HRMS (ESI-TOF).

Table S24: Interruption of Reaction Progress with TEMPO

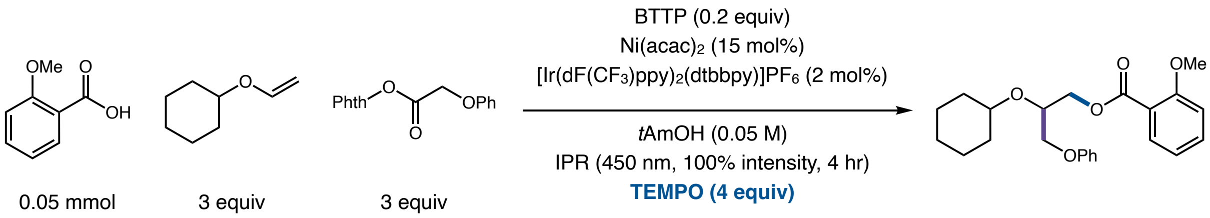

entry	TEMPO addition at	yield
1	0 min	0%
2	5 min	<5%
3	20 min	19%

We next sought to directly observe the interruption of the reaction progress by the addition of TEMPO. To accomplish this, we added a solution of TEMPO to reactions at either 5 minutes or 20 minutes, then allowed irradiation to continue until the full 4 hours were completed. In both cases, we observed suboptimal product formation, with the yield stalling out at <5% and 19%, respectively (Table S24). Furthermore, we identified both of the above TEMPO adducts (**S92** and **S93**) via UPLC-MS. This experiment provides additional support for the radical nature of the mechanism and confirms that TEMPO addition interrupts product formation even for an in-progress reaction.

Procedure for Table S24:

Addition of catalysts via stock solution: (Ir[dF(CF_3)ppy]$_2$(dtbbpy))(PF_6) (2 mol%, 0.001 mmol, 1.1 mg) was added to a dry 8 mL vial equipped with stir bar as a stock solution in acetone (0.001 M, 1 mL stock/vial). Ni(acac)$_2$ (15 mol%, 0.0075 mmol, 1.9 mg) was added to the same vial as a

stock solution in acetone (0.0075 M, 1 mL stock/vial). The acetone was then removed via GeneVac (40 ºC, 1.5 mbar, 15-30 minutes) to leave the catalysts in the reaction vial.

Reaction setup: Under air, 1,3-dioxoisoindolin-2-yl 2-phenoxyacetate (3 equiv, 0.15 mmol, 44.6 mg) was added directly to the prepared vial with catalysts. 2-methoxybenzoic acid (1 equiv, 0.05 mmol, 7.6 mg) was next added as a stock solution in dry *t*-AmOH (0.05 M, 1 mL stock/vial). BTTP (0.2 equiv, 0.01 mmol, 3.1 mg, 3.1 μL) was added via microsyringe. The vial was capped and gently sparged with N_2 for 10 minutes then kept under positive pressure of N_2 for the duration of the experiment. At the same time, TEMPO (4 equiv, 0.2 mmol, 31.3 mg) was dissolved in dry *t*-AmOH (0.4 M, 0.5 mL) and sparged with N_2 for 10 minutes then kept under positive pressure of N_2. (Vinyloxy)cyclohexane (3 equiv, 0.15 mmol, 18.9 mg, 21.3 μL) was added via microsyringe to the reaction vial, which was transferred to a PennPhD m2 integrated photoreactor. The reaction was irradiated with 450 nm light for 4 hours (m2 450 nm LED plate, 100% light intensity, 5200 rpm fan speed, 1000 rpm stirring), with the sparged TEMPO solution transferred to the vial at the appropriate time point.

Reaction analysis: The solvent was removed via GeneVac and the crude reaction mixture analyzed via ^{1}H NMR (500 mHz) and UPLC-MS with 1,3,5-trimethoxybenzene as an internal standard.

Competition Studies:

Table S25: Benzoic Acid Competition Study (2-OMe Benzoic Acid vs Benzoic Acid)

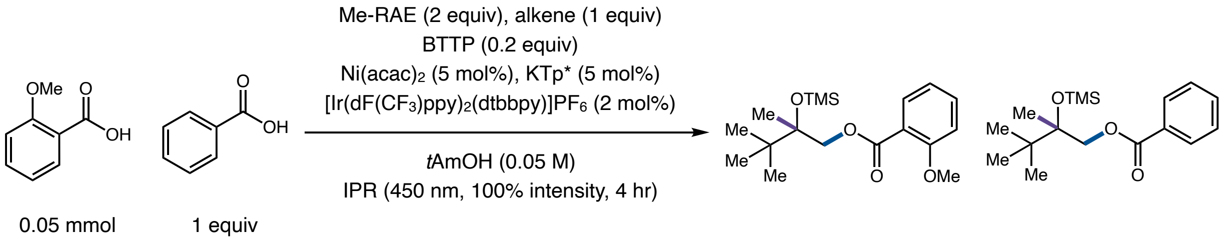

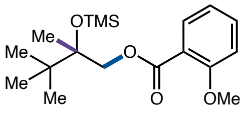

21

62% yield

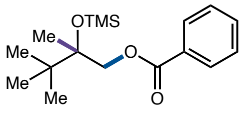

35

6% yield

In this experiment, the reaction was performed in the presence of both 2-methoxy benzoic acid and benzoic acid, which resulted in a 1:10 ratio in favor of the 2-methoxy ester product (**21**) over the unsubstituted product (**35**). This is likely due to the more facile oxidation potential of the electron-rich benzoate, allowing it to be preferentially oxidized in the presence of the electron-neutral species.

Procedure for Table S25:

Addition of catalysts via stock solution: (Ir[dF(CF_3)ppy]$_2$(dtbbpy))(PF_6) (2 mol%, 0.001 mmol, 1.1 mg) was added to a dry 8 mL vial equipped with stir bar as a stock solution in acetone (0.001 M, 1 mL stock/vial). Ni(acac)$_2$ (5 mol%, 0.0025 mmol, 0.6 mg) and KTp* (5 mol%, 0.0025 mmol, 0.8 mg) were added to the same vial as a single stock solution in acetone (0.0025 M, 1 mL stock/vial). The acetone was then removed via GeneVac (40 ºC, 1.5 mbar, 15-30 minutes) to leave the catalysts in the reaction vial.

Reaction setup: Under air, Me-RAE (2 equiv, 0.10 mmol, 20.5 mg), 2-methoxybenzoic acid (1 equiv, 0.05 mmol, 7.6 mg), and benzoic acid (1 equiv, 0.05 mmol, 6.1 mg) were added directly to the prepared vial with catalysts. Dry *t*-AmOH (0.05 M, 1 mL) was added followed by BTTP (0.2 equiv, 0.01 mmol, 3.1 mg, 3.1 μL). The vial was capped and gently sparged with N_2 for 10 minutes. ((3,3-Dimethylbut-1-en-2-yl)oxy)trimethylsilane (1 equiv, 0.05 mmol, 8.6 mg, 10.8 μL) was added against the flow of N_2. The vial was sealed with parafilm and placed in a PennPhD m2 integrated photoreactor. The reaction was irradiated with 450 nm light for 4 hours (m2 450 nm LED plate, 100% light intensity, 5200 rpm fan speed, 1000 rpm stirring).

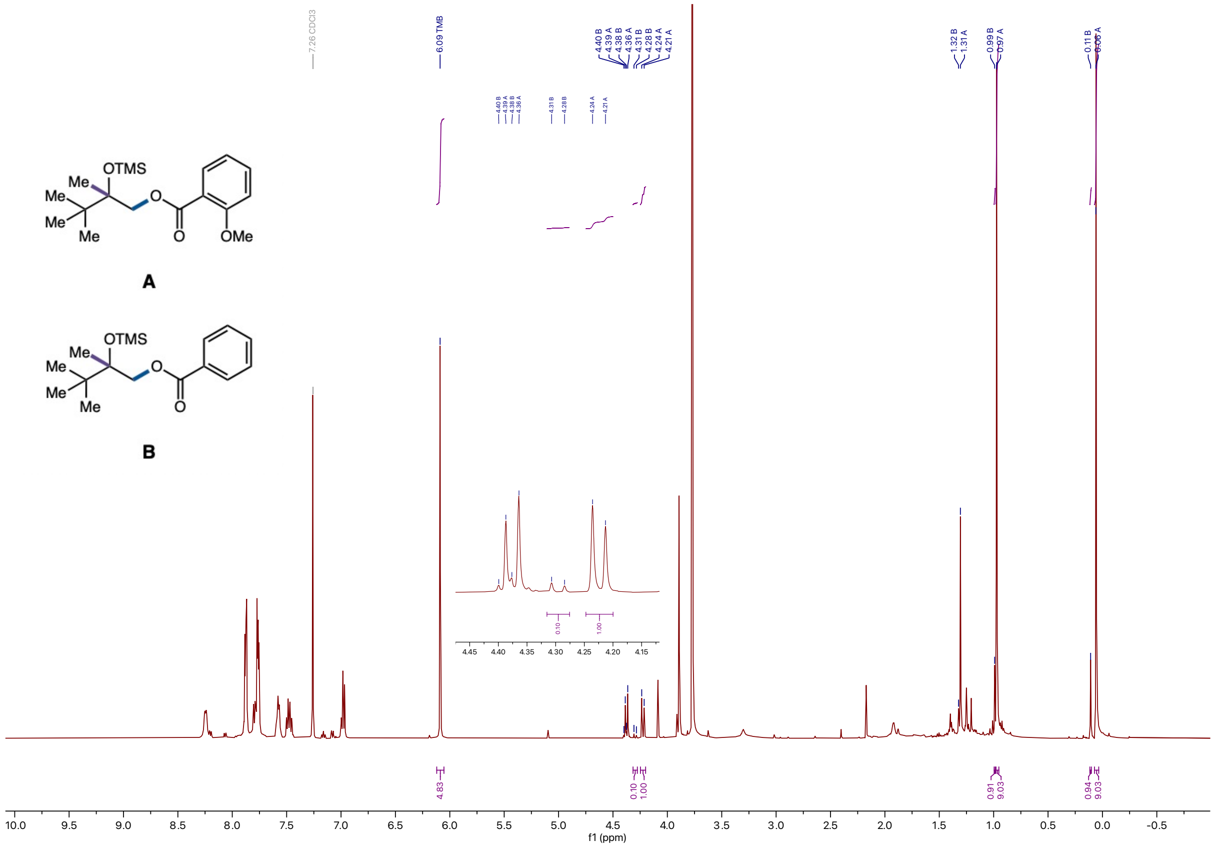

Reaction analysis: The solvent was removed via GeneVac and the crude reaction mixture dissolved in 0.5 mL of $CDCl_3$ containing 0.05 mmol of 1,3,5-trimethoxybenzene (TMB). The vial was sonicated for 30 seconds and a small aliquot (~15 μL) taken for analysis via ^{1}H NMR (500 mHz).

Peaks in the crude NMR were assigned based on analogy to the isolated products, with the NMR ratio determined from the α-oxy signal at 4.22 (d, 1H) for A (**21**) and 4.30 (d, 1H) for B (**35**).

Table S26: Alkene Competition Study (Disubstituted vs Trisubstituted)

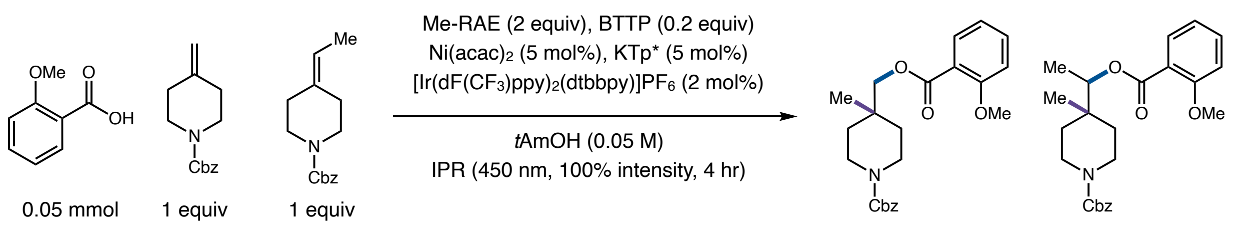

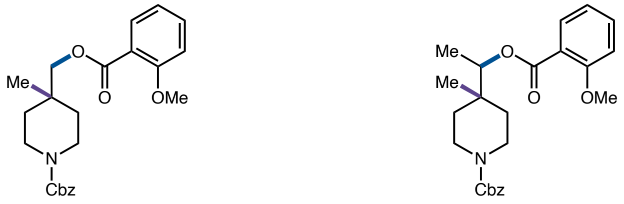

5 *32% yield*

S39 *48% yield*

We performed a second competition experiment wherein a disubstituted and trisubstituted alkene with similar structures were subjected to oxyalkylation in the same vessel. This resulted in a 1:1.5 ratio of the respective products (Table S26, **5** and **S39**). We attribute this selectivity to a more rapid addition of the electrophilic benzoyloxy radical to the trisubstituted alkene. While this substrate has more steric hindrance compared to the disubstituted analogue, trisubstituted alkenes are generally more electron-rich, and this is hypothesized to be the factor that determines selectivity based on this experiment.

Procedure for Table S26:

Addition of catalysts via stock solution: (Ir[dF(CF_3)ppy]$_2$(dtbbpy))(PF_6) (2 mol%, 0.001 mmol, 1.1 mg) was added to a dry 8 mL vial equipped with stir bar as a stock solution in acetone (0.001 M, 1 mL stock/vial). Ni(acac)$_2$ (5 mol%, 0.0025 mmol, 0.6 mg) and KTp* (5 mol%, 0.0025 mmol,

0.8 mg) were added to the same vial as a single stock solution in acetone (0.0025 M, 1 mL stock/vial). The acetone was then removed via GeneVac (40 ºC, 1.5 mbar, 15-30 minutes) to leave the catalysts in the reaction vial.

Reaction setup: Under air, Me-RAE (2 equiv, 0.10 mmol, 20.5 mg), 2-methoxybenzoic acid (1 equiv, 0.05 mmol, 7.6 mg), benzyl 4-methylenepiperidine-1-carboxylate (1 equiv, 0.05 mmol, 11.6 mg, 10.4 µL), and benzyl 4-ethylidenepiperidine-1-carboxylate (**S3**, 1 equiv, 0.05 mmol, 12.3 mg) were added directly to the prepared vial with catalysts. Dry *t*-AmOH (0.05 M, 1 mL) was added followed by BTTP (0.2 equiv, 0.01 mmol, 3.1 mg, 3.1 µL). The vial was capped and gently sparged with N_2 for 10 minutes. The vial was sealed with parafilm and placed in a PennPhD m2 integrated photoreactor. The reaction was irradiated with 450 nm light for 4 hours (m2 450 nm LED plate, 100% light intensity, 5200 rpm fan speed, 1000 rpm stirring).

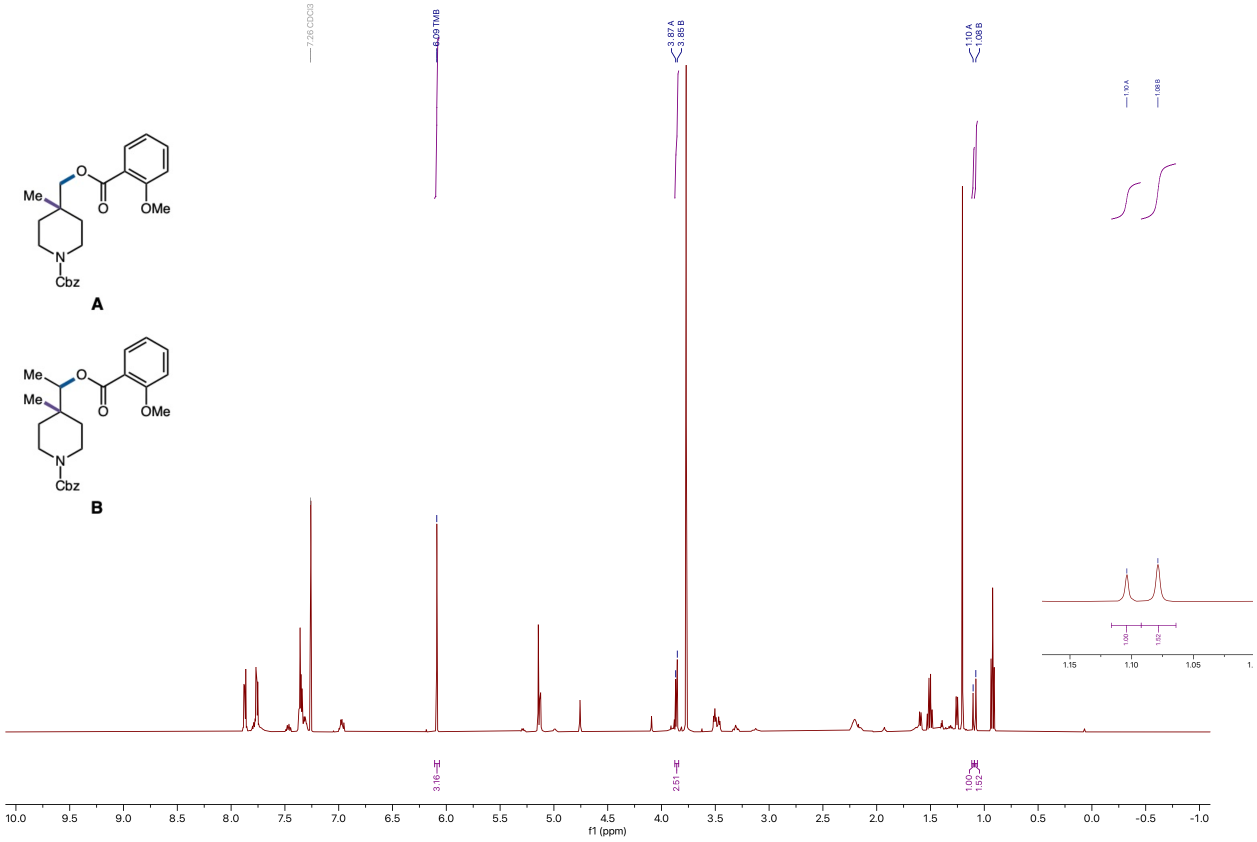

Reaction analysis: The solvent was removed via GeneVac and the crude reaction mixture dissolved in 0.5 mL of $CDCl_3$ containing 0.05 mmol of 1,3,5-trimethoxybenzene. The vial was sonicated for 30 seconds and a small aliquot (~15 μL) taken for analysis via ^1H NMR (500 mHz). Peaks in the crude NMR were assigned based on analogy to the isolated products, with the NMR ratio determined from the methyl signal at 1.10 (s, 3H) for A (**4**) and 1.08 (s, 3H) for B (**S39**).

Compound B (**S39**) was isolated and characterized to confirm the assignment from the crude NMR.

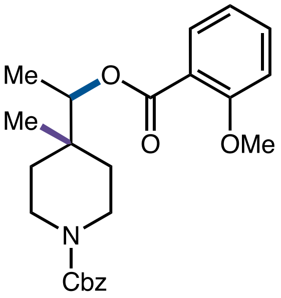

(±)-benzyl 4-(1-((2-methoxybenzoyl)oxy)ethyl)-4-methylpiperidine-1-carboxylate (S39): The title compound was prepared according to General Procedure B with $Ni(acac)_2$ (5 mol%, 25 μmol, 6.4 mg), KTp* (5 mol%, 25 μmol, 8.4 mg), $(Ir[dF(CF_3)ppy]_2(dtbbpy))(PF_6)$ (2 mol%, 10 μmol, 11.2 mg), (1,3-dioxoisoindolin-2-yl)acetate (2 equiv, 1 mmol, 205.2 mg), 2-methoxybenzoic acid (1 equiv, 0.5 mmol, 76.1 mg), benzyl 4-ethylidenepiperidine-1-carboxylate (**S3**) (2 equiv, 1 mmol, 245.3 mg), *tert*-butylimino-tri(pyrrolidino)phosphorane (0.2 equiv, 0.1 mmol, 31.2 mg, 30.6 μL), and 10 mL *t*-amyl alcohol. The crude reaction mixture was concentrated, dissolved in DMSO/MeCN, and purified by automated reverse-phase chromatography (25 g C18 column, 20-50% gradient of 0.1% ammonium hydroxide in water/0.1% ammonium hydroxide in acetonitrile). Additional purification via Preparative HPLC (XBridge BEH C18 OBD column, 20-75% gradient of 0.1% ammonium hydroxide in water/0.1% ammonium hydroxide in acetonitrile). Fractions were directly concentrated and the product dissolved in acetonitrile and filtered through a cotton

plug into a 40 mL vial. The solvent was removed and the product dried on high-vac to yield the title compound as a light yellow oil (138.8 mg, 0.337 mmol, 67% yield). Note: A small amount of product could not be separated from the alkene starting material in two columns, resulting in the lower yield compared to the 79% NMR yield reported in Table S19.

^{1}H NMR (500 MHz, CDCl$_3$) δ 7.79 (dd, *J* = 7.7, 1.9 Hz, 1H), 7.47 (ddd, *J* = 8.3, 7.4, 1.8 Hz, 1H), 7.37 – 7.28 (m, 5H), 7.00 – 6.95 (m, 2H), 5.12 (s, 2H), 4.99 (q, *J* = 6.3 Hz, 1H), 3.91 (s, 2H), 3.85 (s, 3H), 3.19 – 3.05 (m, 2H), 1.64 – 1.57 (m, 2H), 1.48 (s, 1H), 1.33 (s, 1H), 1.26 (d, *J* = 6.4 Hz, 3H), 1.08 (s, 3H).

^{13}C NMR (126 MHz, CDCl$_3$) δ 165.99, 159.36, 155.44, 137.06, 133.61, 131.78, 128.61, 128.07, 127.98, 120.55, 120.24, 112.13, 67.11, 55.89, 40.02, 39.82, 35.80, 33.27, 17.75, 14.20.

IR (film) v_{max} 2943, 2839, 1691, 1448, 1248, 1023, 726 cm^{-1}.

HRMS (ESI-TOF) *m/z* calculated for $C_{24}H_{30}NO_5^+$ ([M+H]$^+$) 412.2118, found 412.2120.

Stern-Volmer Quenching Studies:

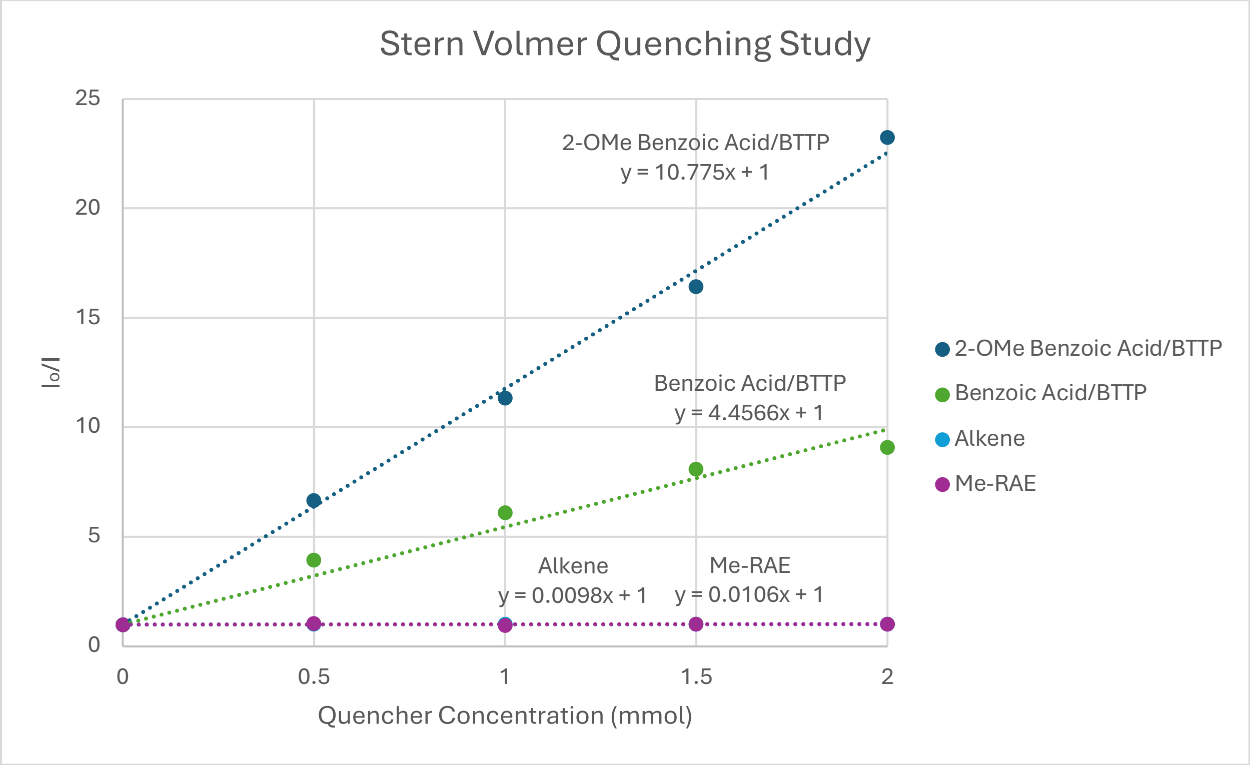

Figure S6: Stern-Volmer Plot of (Ir[dF(CF_3)ppy]$_2$(dtbbpy))(PF_6) with Various Quenchers

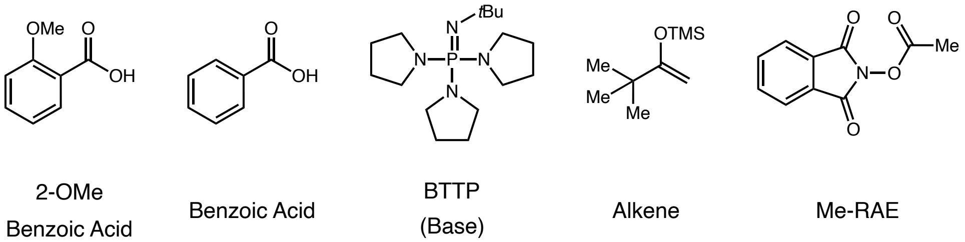

Benzoic acids were deprotonated with BTTP to form the corresponding benzoates

Figure S7: Substrates for Stern-Volmer Quenching Study

To investigate the nature of the species that quenches the excited state photocatalyst, Stern-Volmer quenching studies were carried out with two electronically different benzoates, an electron-rich alkene, and the methyl redox-active ester. To best replicate the standard reaction conditions, the benzoates were formed via stoichiometric addition of the base BTTP to the corresponding benzoic

acid. As shown in Figure S6 above, our studies reveal that the benzoates readily quench the excited state photocatalyst, with the electron rich 2-OMe benzoate showing the fastest rate of quenching, followed by the neutral benzoate. The alkene and the redox active ester were not observed to quench the photocatalyst at appreciable rates. This study supports our mechanistic proposal of the catalytic cycle beginning with benzoate oxidation.

We would like to note that these experiments were performed in EtOAc as the solvent, as the redox-active ester and photocatalyst were not fully soluble in the *t*-AmOH reaction solvent at the necessary concentrations for Stern Volmer. As shown in optimization Table S3, the oxyalkylation reaction can be performed in EtOAc, but with somewhat diminished yield compared to the optimal solvent (41% vs 75% for the optimization substrate).

Procedure and Experimental Details: 10 μM solutions of (Ir[dF(CF_3)ppy]$_2$(dtbbpy))(PF_6) in EtOAc were prepared in screw-top 10.0 mm optical glass cuvettes with 5 different concentrations of quencher, ranging from 0 mM to 2 mM in 0.5 mM increments. Samples were prepared with anhydrous, degassed solvent in a glovebox and sealed with parafilm. Benzoate-containing samples were prepared from a stock solution of the benzoic acid that was deprotonated with BTTP within the glovebox and used immediately. The samples were irradiated at 400 nm using an Agilent Cary Eclipse Fluorescence Spectrophotometer and the emission intensity was observed at 477 nm. The data were plotted in accordance with the Stern-Volmer equation:

$$\frac{I_0}{I} = k_q \tau_0 [Q] + 1$$

where I_0 is the emission intensity of the photocatalyst in the absence of quencher, I is the emission intensity in the presence of a quencher at concentration $[Q]$, k_q is the quenching rate constant, and τ_0 is the excited state lifetime of the photocatalyst.

7. General Procedures for Oxyalkylation

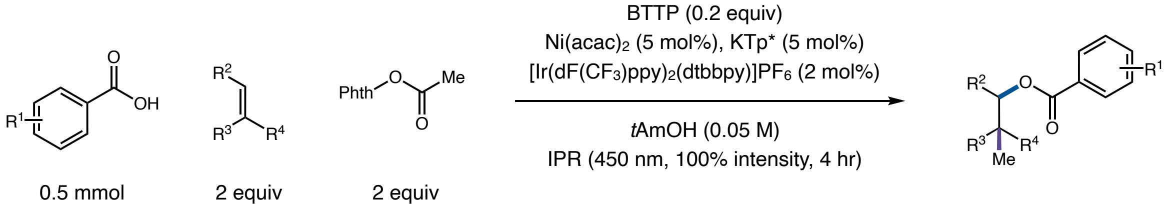

General Procedure B: Oxymethylation of Alkenes with Me-RAE (0.5 mmol scale)

To a dry 40 mL vial equipped with X-shaped stir bar was added Ni(acac)$_2$ (5 mol%, 25 µmol, 6.4 mg), KTp* (5 mol%, 25 µmol, 8.4 mg), (Ir[dF(CF$_3$)ppy]$_2$(dtbbpy))(PF$_6$) (2 mol%, 10 µmol, 11.2 mg), (1,3-dioxoisoindolin-2-yl)acetate (2 equiv, 1 mmol, 205.2 mg), benzoic acid (1 equiv, 0.5 mmol), and alkene (if solid) (2 equiv, 1 mmol). 10 mL of dry *t*-amyl alcohol was added followed by addition of *tert*-butylimino-tri(pyrrolidino)phosphorane (0.2 equiv, 0.1 mmol, 31.2 mg, 30.6 µL) via microsyringe. The vial was capped and gently sparged with N$_2$ for 15 minutes. Alkene (if liquid) (2 equiv, 1 mmol) was added via microsyringe against the flow of N$_2$. The vial was sealed with parafilm and electrical tape then placed in a PennPhD m2 integrated photoreactor. The reaction was irradiated with 450 nm light for 4 hours (m2 450 nm LED plate, 100% light intensity, 5200 rpm fan speed, 1000 rpm stirring). The solvent was removed via GeneVac and the crude reaction mixture purified via Reverse Phase Biotage. Additional purification was performed, if necessary, via preparative HPLC or automated flash chromatography.

Notes:

- Low-yielding substrate combinations can often be improved by the addition of an extra equivalent of alkene.
- Electron-deficient benzoic acids can be tested with the alternative reaction conditions shown in Table S17, particularly the use of 2.5 mol% (Ir[dF(CF$_3$)ppy]$_2$(dtbbpy))(PF$_6$), 2 equivalents of Me-RAE-Cl$_4$, and 3 equivalents of alkene.

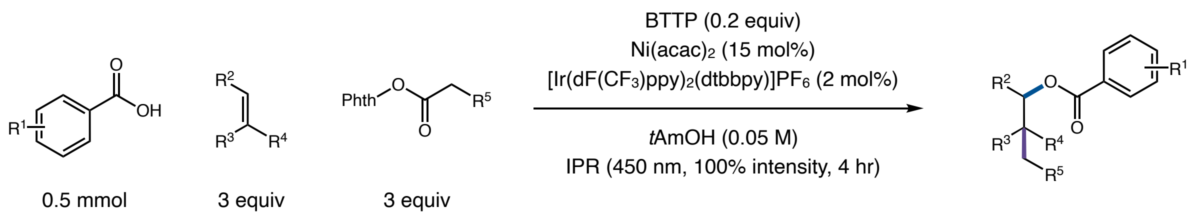

General Procedure C: Oxyalkylation of Alkenes with primary alkyl RAEs (0.5 mmol scale)

To a dry 40 mL vial equipped with X-shaped stir bar was added Ni(acac)$_2$ (15 mol%, 75 µmol, 19.3 mg), (Ir[dF(CF$_3$)ppy]$_2$(dtbbpy))(PF$_6$) (2 mol%, 10 µmol, 11.2 mg), primary alkyl redox-active ester (3 equiv, 1.5 mmol), benzoic acid (1 equiv, 0.5 mmol), and alkene (if solid) (3 equiv, 1.5 mmol). 10 mL of dry *t*-amyl alcohol was added followed by addition of *tert*-butylimino-tri(pyrrolidino)phosphorane (0.2 equiv, 0.1 mmol, 31.2 mg, 30.6 µL) via microsyringe. The vial was capped and gently sparged with N_2 for 15 minutes. Alkene (if liquid) (3 equiv, 1.5 mmol) was added via microsyringe against the flow of N_2. The vial was sealed with parafilm and electrical tape then placed in a PennPhD m2 integrated photoreactor. The reaction was irradiated with 450 nm light for 4 hours (m2 450 nm LED plate, 100% light intensity, 5200 rpm fan speed, 1000 rpm stirring). The solvent was removed via GeneVac and the crude reaction mixture purified via Reverse Phase Biotage. Additional purification was performed, if necessary, via preparative HPLC or automated flash chromatography.

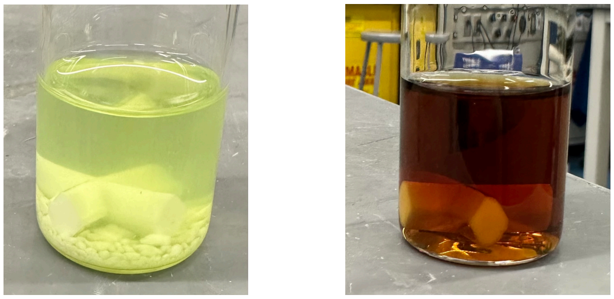

Reaction before irradiation

Reaction after irradiation

Figure S8: Example of Reaction Appearance Before and After Irradiation

As shown in Figure S8, the redox-active ester and the photocatalyst are often not fully soluble in *t*AmOH at the beginning of the reaction. However, the solution is generally homogenous after irradiation. The color of a successful reaction varies significantly but is commonly orange or brown as shown above.

8. Additional Procedures: 4 mmol Scale Reaction and One-Pot Oxyalkylation/Hydrolysis

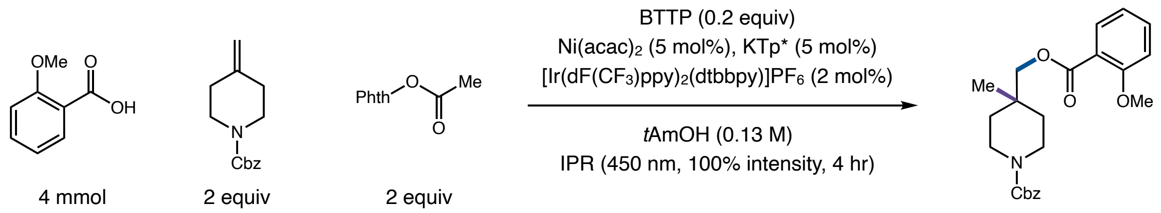

Procedure D: Oxyalkylation of benzyl 4-methylenepiperidine-1-carboxylate (4 mmol scale)

To a dry 40 mL vial equipped with X-shaped stir bar was added Ni(acac)$_2$ (5 mol%, 0.2 mmol, 51.4 mg), KTp* (5 mol%, 0.2 mmol, 67.3 mg), (Ir[dF(CF$_3$)ppy]$_2$(dtbbpy))(PF$_6$) (2 mol%, 80 µmol, 89.8 mg), (1,3-dioxoisoindolin-2-yl)acetate (2 equiv, 8 mmol, 1.64 g), and 2-methoxybenzoic acid (1 equiv, 4 mmol, 608.6 mg). 30 mL of dry *t*-amyl alcohol was added followed by addition of *tert*-butylimino-tri(pyrrolidino)phosphorane (0.2 equiv, 0.8 mmol, 249.9 mg, 244.6 µL) via microsyringe. The vial was capped and gently sparged with N$_2$ for 15 minutes. Benzyl 4-methylenepiperidine-1-carboxylate (2 equiv, 8 mmol, 1.85 g, 1.67 mL) was added via syringe against the flow of N$_2$. The vial was sealed with parafilm and electrical tape then placed in a PennPhD m2 integrated photoreactor. The reaction was irradiated with 450 nm light for 4 hours (m2 450 nm LED plate, 100% light intensity, 5200 rpm fan speed, 1000 rpm stirring). Upon completion, the reaction mixture was transferred to a 250 mL round bottom flask and the solvent removed under reduced pressure. The crude reaction mixture was dissolved in DMSO/MeCN and purified by automated reverse-phase chromatography (50 g C18 column, 20-100% gradient of 0.1% ammonium hydroxide in water/0.1% ammonium hydroxide in acetonitrile). Additional purification performed via automated flash chromatography (50 g high performance silica column, 0-35% ethyl acetate/hexanes) to yield benzyl 4-(((2-methoxybenzoyl)oxy)methyl)-4-methylpiperidine-1-carboxylate (**5**) as a yellow oil (1.01 g, 2.54 mmol, 64% yield).

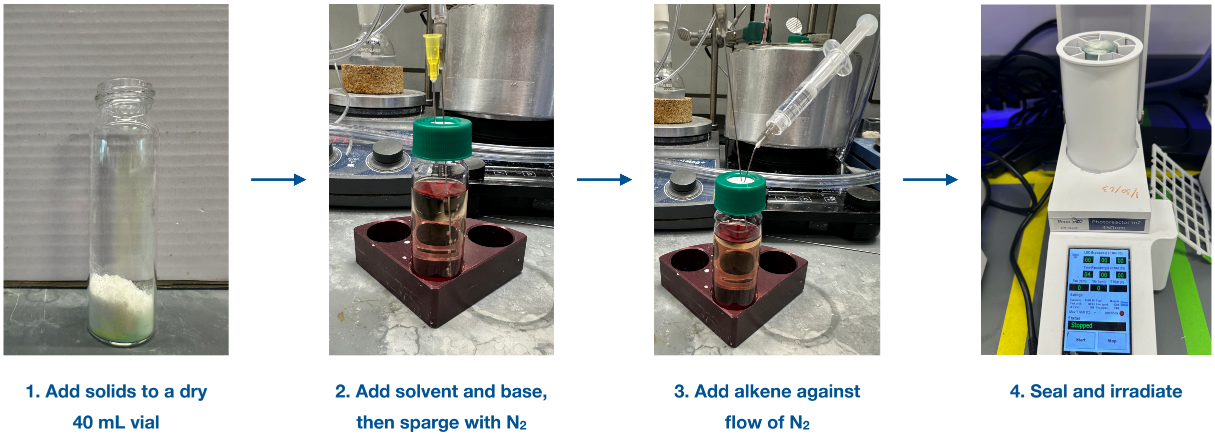

Figure S9: Visual Procedure for 4 mmol Scale Oxyalkylation

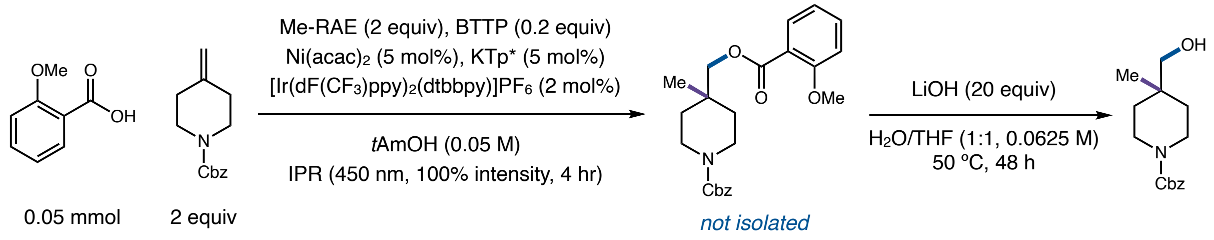

Procedure E: One-pot oxyalkylation/hydrolysis of benzyl 4-methylenepiperidine-1-carboxylate

To a dry 40 mL vial equipped with X-shaped stir bar was added Ni(acac)$_2$ (5 mol%, 25 µmol, 6.4 mg), KTp* (5 mol%, 25 µmol, 8.4 mg), (Ir[dF(CF$_3$)ppy]$_2$(dtbbpy))(PF$_6$) (2 mol%, 10 µmol, 11.2 mg), (1,3-dioxoisoindolin-2-yl)acetate (2 equiv, 1 mmol, 205.2 mg), and 2-methoxybenzoic acid (1 equiv, 0.5 mmol, 76.1 mg). 10 mL of dry *t*-amyl alcohol was added followed by addition of *tert*-butylimino-tri(pyrrolidino)phosphorane (0.2 equiv, 0.1 mmol, 31.2 mg, 30.6 µL) via microsyringe. The vial was capped and gently sparged with N$_2$ for 15 minutes. Benzyl 4-methylenepiperidine-1-carboxylate (2 equiv, 1 mmol, 231.3 mg, 208.4 µL) was added via microsyringe. The vial was sealed with parafilm and electrical tape then placed in a PennPhD m2 integrated photoreactor. The

reaction was irradiated with 450 nm light for 4 hours (m2 450 nm LED plate, 100% light intensity, 5200 rpm fan speed, 1000 rpm stirring). Following irradiation, the *tert*-amyl alcohol solvent was removed via GeneVac. In the same reaction vessel, the crude reaction mixture was dissolved in 4 mL tetrahydrofuran. Lithium hydroxide (20 equiv, 10 mmol, 240 mg) in 4 mL H_2O was added and the mixture stirred at 50 ºC for 48 hours. After cooling to room temperature, the mixture was transferred to a separatory funnel. Additional water/brine was added and the mixture extracted with ethyl acetate (3 x 100 mL). The combined organics were dried, filtered through celite, and concentrated. The resulting crude residue was purified via Preparative HPLC (XBridge BEH C18 OBD column, 20-34% gradient of 0.1% ammonium hydroxide in water/0.1% ammonium hydroxide in acetonitrile). Fractions were directly concentrated and the product dissolved in MeCN and filtered through a cotton plug into a 40 mL vial. The solvent was removed and the product dried on high-vac to yield benzyl 4-(hydroxymethyl)-4-methylpiperidine-1-carboxylate (**61**) as a pale yellow oil (82.0 mg, 0.311 mmol, 62% yield over two steps).

9. Experimental Data

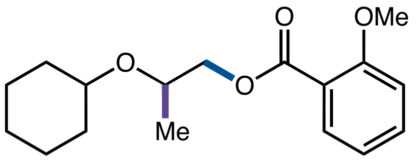

(±)-2-(cyclohexyloxy)propyl 2-methoxybenzoate (6): The title compound was prepared according to General Procedure B with Ni(acac)$_2$ (5 mol%, 25 µmol, 6.4 mg), KTp* (5 mol%, 25 µmol, 8.4 mg), (Ir[dF(CF$_3$)ppy]$_2$(dtbbpy))(PF$_6$) (2 mol%, 10 µmol, 11.2 mg), (1,3-dioxoisoindolin-2-yl)acetate (2 equiv, 1 mmol, 205.2 mg), 2-methoxybenzoic acid (1 equiv, 0.5 mmol, 76.1 mg), (vinyloxy)cyclohexane (2 equiv, 1 mmol, 126.2 mg, 141.6 µL), *tert*-butylimino-tri(pyrrolidino)phosphorane (0.2 equiv, 0.1 mmol, 31.2 mg, 30.6 µL), and 10 mL *t*-amyl alcohol. The crude reaction mixture was concentrated, dissolved in DMSO/MeCN, and purified by automated reverse-phase chromatography (25 g C18 column, 20-50% gradient of 0.1% ammonium hydroxide in water/0.1% ammonium hydroxide in acetonitrile). Fractions were directly concentrated and the product dissolved in acetonitrile and filtered through a cotton plug into a 40 mL vial. The solvent was removed and the product dried on high-vac to yield the title compound as a dark oil (110.9 mg, 0.379 mmol, 76% yield).

^{1}H NMR (500 MHz, CDCl$_3$) δ 7.82 (dd, *J* = 7.9, 1.8 Hz, 1H), 7.47 (ddd, *J* = 8.4, 7.4, 1.8 Hz, 1H), 7.00 – 6.94 (m, 2H), 4.25 (dd, *J* = 11.2, 6.3 Hz, 1H), 4.18 (dd, *J* = 11.2, 5.1 Hz, 1H), 3.92 – 3.86 (m, 4H), 3.40 (tt, *J* = 9.4, 3.8 Hz, 1H), 1.92 – 1.86 (m, 2H), 1.76 – 1.70 (m, 2H), 1.55 – 1.49 (m, 1H), 1.32 – 1.17 (m, 8H).

^{13}C NMR (126 MHz, CDCl$_3$) δ ^{13}C NMR (126 MHz, CDCl$_3$) δ 166.09, 159.46, 133.68, 131.83, 120.20, 120.15, 112.11, 76.59, 70.56, 68.31, 56.05, 33.23, 33.07, 25.88, 24.46, 18.52.

IR (film) v_{max} 2931, 2856, 1727, 1295, 1252, 1075, 755 cm^{-1}.

HRMS (ESI-TOF) *m/z* calculated for $C_{17}H_{24}NaO_4^+$ ([M+Na]$^+$) 315.1567, found 315.1567.

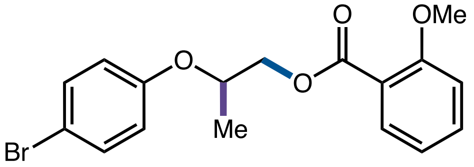

(±)-2-(4-bromophenoxy)propyl 2-methoxybenzoate (7): The title compound was prepared according to General Procedure B with Ni(acac)$_2$ (5 mol%, 25 μmol, 6.4 mg), KTp* (5 mol%, 25 μmol, 8.4 mg), (Ir[dF(CF$_3$)ppy]$_2$(dtbbpy))(PF$_6$) (2 mol%, 10 μmol, 11.2 mg), (1,3-dioxoisoindolin-2-yl)acetate (2 equiv, 1 mmol, 205.2 mg), 2-methoxybenzoic acid (1 equiv, 0.5 mmol, 76.1 mg), 1-bromo-4-(vinyloxy)benzene (2 equiv, 1 mmol, 199.0 mg, 213.6 μL), *tert*-butylimino-tri(pyrrolidino)phosphorane (0.2 equiv, 0.1 mmol, 31.2 mg, 30.6 μL), and 10 mL *t*-amyl alcohol. The crude reaction mixture was concentrated, dissolved in DMSO/MeCN, and purified by automated reverse-phase chromatography (25 g C18 column, 20-53% gradient of 0.1% ammonium hydroxide in water/0.1% ammonium hydroxide in acetonitrile). Fractions were directly concentrated and the product dissolved in acetonitrile and filtered through a cotton plug into a 40 mL vial. The solvent was removed and the product dried on high-vac to yield the title compound as a yellow oil (127.7 mg, 0.350 mmol, 70% yield).

^{1}H NMR (500 MHz, CDCl$_3$) δ 7.70 (dd, *J* = 7.6, 1.8 Hz, 1H), 7.47 (ddd, *J* = 8.3, 7.4, 1.8 Hz, 1H), 7.39 – 7.32 (m, 2H), 6.98 – 6.93 (m, 2H), 6.89 – 6.83 (m, 2H), 4.74 – 4.64 (m, 1H), 4.46 (dd, *J* = 11.5, 6.4 Hz, 1H), 4.35 (dd, *J* = 11.6, 4.5 Hz, 1H), 3.86 (s, 3H), 1.41 (d, *J* = 6.2 Hz, 3H).

^{13}C NMR (126 MHz, CDCl$_3$) δ 166.00, 159.49, 157.22, 133.93, 132.50, 131.88, 120.26, 119.70, 118.13, 113.44, 112.15, 72.55, 67.16, 56.05, 17.07.

IR (film) v_{max} 2937, 2856, 1700, 1328, 1257, 1114, 1069, 751 cm^{-1}.

HRMS (ESI-TOF) *m/z* calculated for $C_{17}H_{18}BrO_4^+$ ([M+H]$^+$) 365.0383, found 365.0386.

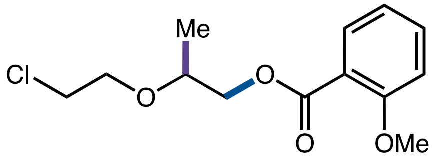

(±)-2-(2-chloroethoxy)propyl 2-methoxybenzoate (8): The title compound was prepared according to General Procedure B with Ni(acac)$_2$ (5 mol%, 25 μmol, 6.4 mg), KTp* (5 mol%, 25 μmol, 8.4 mg), (Ir[dF(CF$_3$)ppy]$_2$(dtbbpy))(PF$_6$) (2 mol%, 10 μmol, 11.2 mg), (1,3-dioxoisoindolin-2-yl)acetate (2 equiv, 1 mmol, 205.2 mg), 2-methoxybenzoic acid (1 equiv, 0.5 mmol, 76.1 mg), (2-chloroethoxy)ethene (2 equiv, 1 mmol, 106.6 mg, 101.7 μL), *tert*-butylimino-tri(pyrrolidino)phosphorane (0.2 equiv, 0.1 mmol, 31.2 mg, 30.6 μL), and 10 mL *t*-amyl alcohol. The crude reaction mixture was concentrated, dissolved in DMSO/MeCN, and purified by automated reverse-phase chromatography (25 g C18 column, 20-40% gradient of 0.1% ammonium hydroxide in water/0.1% ammonium hydroxide in acetonitrile). Fractions were directly concentrated and the product dissolved in acetonitrile and filtered through a cotton plug into a 40 mL vial. The solvent was removed and the product dried on high-vac to yield the title compound as a yellow oil (98.0 mg, 0.359 mmol, 72% yield).

^{1}H NMR (500 MHz, CDCl$_3$) δ 7.82 (dd, *J* = 7.9, 1.8 Hz, 1H), 7.47 (ddd, *J* = 8.3, 7.4, 1.8 Hz, 1H), 7.00 – 6.95 (m, 2H), 4.34 – 4.22 (m, 2H), 3.90 (s, 3H), 3.89 – 3.78 (m, 3H), 3.61 (t, *J* = 6.0 Hz, 2H), 1.28 (d, *J* = 6.5 Hz, 3H).

^{13}C NMR (126 MHz, CDCl$_3$) δ 166.09, 159.41, 133.80, 131.89, 120.27, 119.95, 112.13, 74.40, 69.73, 67.76, 56.05, 43.17, 17.41.

IR (film) v_{max} 2966, 2879, 1726, 1298, 1256, 1129, 1083, 756 cm^{-1}.

HRMS (ESI-TOF) *m/z* calculated for $C_{13}H_{17}ClNaO_4^+$ ([M+Na]$^+$) 295.0708, found 295.0705.

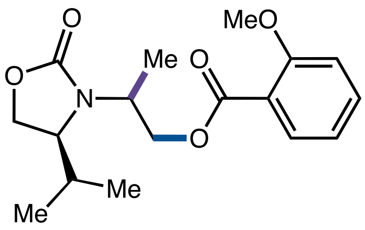

2-((*S*)-4-isopropyl-2-oxooxazolidin-3-yl)propyl 2-methoxybenzoate (9): The title compound was prepared according to General Procedure B with Ni(acac)$_2$ (5 mol%, 25 µmol, 6.4 mg), KTp* (5 mol%, 25 µmol, 8.4 mg), (Ir[dF(CF$_3$)ppy]$_2$(dtbbpy))(PF$_6$) (2 mol%, 10 µmol, 11.2 mg), (1,3-dioxoisoindolin-2-yl)acetate (2 equiv, 1 mmol, 205.2 mg), 2-methoxybenzoic acid (1 equiv, 0.5 mmol, 76.1 mg), (*S*)-4-isopropyl-3-vinyloxazolidin-2-one (2 equiv, 1 mmol, 155.2 mg, added before sparge), *tert*-butylimino-tri(pyrrolidino)phosphorane (0.2 equiv, 0.1 mmol, 31.2 mg, 30.6 µL), and 10 mL *t*-amyl alcohol. The crude reaction mixture was concentrated, dissolved in DMSO/MeCN, and purified by automated reverse-phase chromatography (25 g C18 column, 20-35% gradient of 0.1% ammonium hydroxide in water/0.1% ammonium hydroxide in acetonitrile). Fractions were directly concentrated and the product dissolved in acetonitrile and filtered through a cotton plug into a 40 mL vial. The solvent was removed and the product dried on high-vac to yield the title compound as a yellow oil (98.7 mg, 0.307 mmol, 61% yield, 1:1.9 d.r., unassigned).

^{1}H NMR (500 MHz, CDCl$_3$) (summary of diastereomers) δ 7.79 (ddd, *J* = 9.8, 7.9, 1.8 Hz, 1H), 7.50 – 7.44 (m, 1H), 7.01 – 6.94 (m, 2H), 4.60 (dd, *J* = 11.6, 8.6 Hz, 0.66H), 4.51 (dd, *J* = 11.1, 8.4 Hz, 0.34H), 4.43 – 4.37 (m, 1H), 4.20 – 4.10 (m, 1.71H), 4.10 – 4.03 (m, 1H), 3.94 – 3.90 (m, 0.31H), 3.89 (s, 3H), 3.80 – 3.74 (m, 1H), 2.11 – 2.00 (m, 1H), 1.44 (d, *J* = 7.1 Hz, 1H), 1.36 (d, *J* = 7.0 Hz, 2H), 0.90 (dd, *J* = 9.2, 6.8 Hz, 3H), 0.85 (dd, *J* = 7.0, 4.2 Hz, 3H).

^{13}C NMR (126 MHz, CDCl$_3$) (summary of diastereomers) δ 166.04, 166.01, 159.46, 159.45, 158.43, 157.77, 134.02, 133.95, 131.91, 131.88, 120.36, 120.31, 119.65, 119.48, 112.19, 112.10,

65.59, 64.66, 63.05, 62.88, 61.08, 59.34, 56.02, 55.99, 49.36, 49.26, 29.57, 29.31, 18.29, 18.09, 15.84, 14.93, 14.27, 14.01.

IR (film) v_{max} 2961, 2877, 1745, 1700, 1301, 1255, 1128, 752 cm^{-1}.

HRMS (ESI-TOF) *m/z* calculated for $C_{17}H_{24}NO_5^+$ ($[M+H]^+$) 322.1649, found 322.1651.

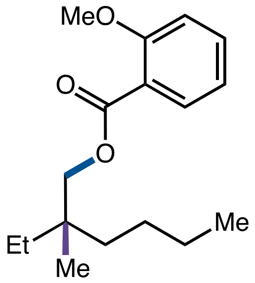

(±)-2-ethyl-2-methylhexyl 2-methoxybenzoate (10): The title compound was prepared according to General Procedure B with Ni(acac)$_2$ (5 mol%, 25 µmol, 6.4 mg), KTp* (5 mol%, 25 µmol, 8.4 mg), (Ir[dF(CF$_3$)ppy]$_2$(dtbbpy))(PF$_6$) (2 mol%, 10 µmol, 11.2 mg), (1,3-dioxoisoindolin-2-yl)acetate (2 equiv, 1 mmol, 205.2 mg), 2-methoxybenzoic acid (1 equiv, 0.5 mmol, 76.1 mg), 3-methyleneheptane (2 equiv, 1 mmol, 112.2 mg, 153.7 µL), *tert*-butylimino-tri(pyrrolidino)phosphorane (0.2 equiv, 0.1 mmol, 31.2 mg, 30.6 µL), and 10 mL *t*-amyl alcohol. The crude reaction mixture was concentrated, dissolved in DMSO/MeCN, and purified by automated reverse-phase chromatography (25 g C18 column, 20-75% gradient of 0.1% ammonium hydroxide in water/0.1% ammonium hydroxide in acetonitrile). Fractions were directly concentrated and the product dissolved in acetonitrile and filtered through a cotton plug into a 40 mL vial. The solvent was removed and the product dried on high-vac to yield the title compound as a yellow oil (97.9 mg, 0.352 mmol, 70% yield).

^{1}H NMR (500 MHz, CDCl$_3$) δ 7.80 (dd, *J* = 8.0, 1.9 Hz, 1H), 7.46 (ddd, *J* = 8.3, 7.4, 1.8 Hz, 1H), 7.00 – 6.96 (m, 2H), 4.03 (s, 2H), 3.90 (s, 3H), 1.43 – 1.37 (m, 2H), 1.35 – 1.22 (m, 6H), 0.93 (s, 3H), 0.90 (t, *J* = 7.0 Hz, 3H), 0.85 (t, *J* = 7.5 Hz, 3H).

^{13}C NMR (126 MHz, CDCl$_3$) δ 166.62, 159.39, 133.49, 131.79, 120.62, 120.20, 112.11, 70.84, 55.95, 36.37, 36.35, 29.34, 25.74, 23.76, 22.00, 14.27, 8.02.

IR (film) v_{max} 2959, 2930, 2873, 1701, 1298, 1247, 1129, 1077, 753 cm^{-1}.

HRMS (ESI-TOF) *m/z* calculated for $C_{17}H_{27}O_3^+$ ([M+H]$^+$) 279.1955, found 279.1951.

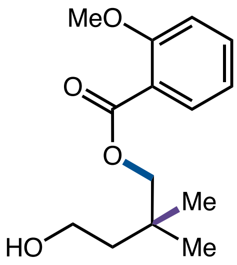

4-hydroxy-2,2-dimethylbutyl 2-methoxybenzoate (11): The title compound was prepared according to General Procedure B with Ni(acac)$_2$ (5 mol%, 25 μmol, 6.4 mg), KTp* (5 mol%, 25 μmol, 8.4 mg), (Ir[dF(CF$_3$)ppy]$_2$(dtbbpy))(PF$_6$) (2 mol%, 10 μmol, 11.2 mg), (1,3-dioxoisoindolin-2-yl)acetate (2 equiv, 1 mmol, 205.2 mg), 2-methoxybenzoic acid (1 equiv, 0.5 mmol, 76.1 mg), 3-methylbut-3-en-1-ol (2 equiv, 1 mmol, 86.1 mg, 101.3 μL), *tert*-butylimino-tri(pyrrolidino)phosphorane (0.2 equiv, 0.1 mmol, 31.2 mg, 30.6 μL), and 10 mL *t*-amyl alcohol. The crude reaction mixture was concentrated, dissolved in DMSO/MeCN, and purified by automated reverse-phase chromatography (25 g C18 column, 20-40% gradient of 0.1% ammonium hydroxide in water/0.1% ammonium hydroxide in acetonitrile). Fractions were directly concentrated and the product dissolved in acetonitrile and filtered through a cotton plug into a 40

mL vial. The solvent was removed and the product dried on high-vac to yield the title compound as a yellow oil (80.0 mg, 0.317 mmol, 63% yield).

^{1}H NMR (500 MHz, CDCl$_3$) δ 7.80 (dd, *J* = 8.0, 1.9 Hz, 1H), 7.45 (ddd, *J* = 9.2, 7.6, 1.9 Hz, 1H), 6.99 – 6.93 (m, 2H), 4.04 (s, 2H), 3.87 (s, 3H), 3.73 (t, *J* = 7.2 Hz, 2H), 2.38 (b, 1H), 1.65 (t, *J* = 7.2 Hz, 2H), 1.01 (s, 6H).

^{13}C NMR (126 MHz, CDCl$_3$) δ 166.57, 159.17, 133.64, 131.86, 120.24, 120.17, 112.05, 72.88, 59.38, 55.87, 41.99, 33.50, 24.96.

IR (film) v_{max} 3381, 2960, 2876, 1704, 1300, 1246, 1080, 1023, 754 cm^{-1}.

HRMS (ESI-TOF) *m/z* calculated for $C_{14}H_{20}NaO_4^+$ ([M+Na]$^+$) 275.1254, found 275.1256.

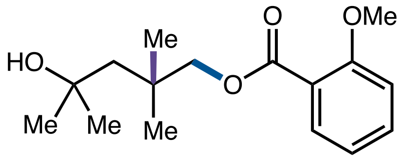

4-hydroxy-2,2,4-trimethylpentyl 2-methoxybenzoate (12): The title compound was prepared according to General Procedure B with Ni(acac)$_2$ (5 mol%, 25 µmol, 6.4 mg), KTp* (5 mol%, 25 µmol, 8.4 mg), (Ir[dF(CF$_3$)ppy]$_2$(dtbbpy))(PF$_6$) (2 mol%, 10 µmol, 11.2 mg), (1,3-dioxoisoindolin-2-yl)acetate (2 equiv, 1 mmol, 205.2 mg), 2-methoxybenzoic acid (1 equiv, 0.5 mmol, 76.1 mg), 2,4-dimethylpent-4-en-2-ol (2 equiv, 1 mmol, 114.2 mg, 136.6 µL), *tert*-butylimino-tri(pyrrolidino)phosphorane (0.2 equiv, 0.1 mmol, 31.2 mg, 30.6 µL), and 10 mL *t*-amyl alcohol. The crude reaction mixture was concentrated, dissolved in DMSO/MeCN, and purified by automated reverse-phase chromatography (25 g C18 column, 20-50% gradient of 0.1% ammonium hydroxide in water/0.1% ammonium hydroxide in acetonitrile). Fractions were directly concentrated and the product dissolved in acetonitrile and filtered through a cotton plug into a 40

mL vial. The solvent was removed and the product dried on high-vac to yield the title compound as a yellow oil (104.0 mg, 0.371 mmol, 74% yield).

^{1}H NMR (500 MHz, CDCl$_3$) δ 7.84 (dd, *J* = 7.7, 1.9 Hz, 1H), 7.48 (ddd, *J* = 8.3, 7.4, 1.8 Hz, 1H), 7.02 – 6.97 (m, 2H), 4.23 (s, 2H), 3.91 (s, 3H), 2.15 (b, 1H), 1.65 (s, 2H), 1.31 (s, 6H), 1.12 (s, 6H).

^{13}C NMR (126 MHz, CDCl$_3$) δ 166.83, 159.02, 133.68, 132.13, 120.43, 120.37, 112.08, 73.16, 71.83, 55.93, 51.39, 35.07, 32.07, 27.02.

IR (film) v_{max} 3520, 2963, 2877, 1701, 1303, 1255, 1113, 1083, 753 cm^{-1}.

HRMS (ESI-TOF) *m/z* calculated for $C_{16}H_{24}NaO_4^+$ ([M+Na]$^+$) 303.1567, found 303.1563.

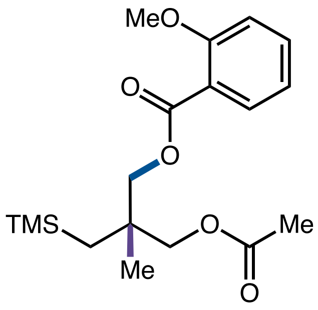

(±)-3-acetoxy-2-methyl-2-((trimethylsilyl)methyl)propyl 2-methoxybenzoate (13): The title compound was prepared according to General Procedure B with Ni(acac)$_2$ (5 mol%, 25 µmol, 6.4 mg), KTp* (5 mol%, 25 µmol, 8.4 mg), (Ir[dF(CF$_3$)ppy]$_2$(dtbbpy))(PF$_6$) (2 mol%, 10 µmol, 11.2 mg), (1,3-dioxoisoindolin-2-yl)acetate (2 equiv, 1 mmol, 205.2 mg), 2-methoxybenzoic acid (1 equiv, 0.5 mmol, 76.1 mg), 2-((trimethylsilyl)methyl)allyl acetate (2 equiv, 1 mmol, 186.3 mg, 212.5 µL), *tert*-butylimino-tri(pyrrolidino)phosphorane (0.2 equiv, 0.1 mmol, 31.2 mg, 30.6 µL), and 10 mL *t*-amyl alcohol. The crude reaction mixture was concentrated, dissolved in DMSO/MeCN, and purified by automated reverse-phase chromatography (25 g C18 column, 20-75% gradient of 0.1% ammonium hydroxide in water/0.1% ammonium hydroxide in acetonitrile).

Fractions were directly concentrated and the product dissolved in acetonitrile and filtered through a cotton plug into a 40 mL vial. The solvent was removed and the product dried on high-vac to yield the title compound as a yellow oil (129.2 mg, 0.367 mmol, 73% yield).

^{1}H NMR (500 MHz, CDCl$_3$) δ 7.80 (dd, *J* = 7.7, 1.8 Hz, 1H), 7.51 – 7.43 (m, 1H), 7.01 – 6.96 (m, 2H), 4.15 – 4.07 (m, 2H), 4.03 – 3.94 (m, 2H), 3.89 (s, 3H), 2.06 (s, 3H), 1.08 (s, 3H), 0.80 (d, *J* = 3.5 Hz, 2H), 0.08 (s, 9H).

^{13}C NMR (126 MHz, CDCl$_3$) δ 171.23, 166.50, 159.39, 133.72, 131.95, 120.26, 120.18, 112.08, 70.08, 70.01, 55.91, 37.46, 24.19, 22.02, 21.07, 0.88.

IR (film) ν_{max} 2961, 2841, 1728, 1701, 1303, 1254, 1113, 1082, 755 cm^{-1}.

HRMS (ESI-TOF) *m/z* calculated for $C_{18}H_{28}NaO_5Si^+$ ([M+Na]$^+$) 375.1598, found 375.1593.

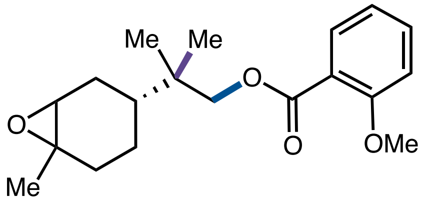

2-methyl-2-((3*R*)-6-methyl-7-oxabicyclo[4.1.0]heptan-3-yl)propyl 2-methoxybenzoate (14): The title compound was prepared according to General Procedure B with Ni(acac)$_2$ (5 mol%, 25 µmol, 6.4 mg), KTp* (5 mol%, 25 µmol, 8.4 mg), (Ir[dF(CF$_3$)ppy]$_2$(dtbbpy))(PF$_6$) (2 mol%, 10 µmol, 11.2 mg), (1,3-dioxoisoindolin-2-yl)acetate (2 equiv, 1 mmol, 205.2 mg), 2-methoxybenzoic acid (1 equiv, 0.5 mmol, 76.1 mg), (4*R*)-1-methyl-4-(prop-1-en-2-yl)-7-oxabicyclo[4.1.0]heptane (2 equiv, 1 mmol, 152.2 mg, 163.9 µL), *tert*-butylimino-tri(pyrrolidino)phosphorane (0.2 equiv, 0.1 mmol, 31.2 mg, 30.6 µL), and 10 mL *t*-amyl alcohol. The crude reaction mixture was concentrated, dissolved in DMSO/MeCN, and purified by automated reverse-phase chromatography (25 g C18 column, 20-55% gradient of 0.1% ammonium hydroxide in water/0.1%

ammonium hydroxide in acetonitrile). Fractions were directly concentrated and the product dissolved in acetonitrile and filtered through a cotton plug into a 40 mL vial. The solvent was removed and the product dried on high-vac to yield the title compound as a yellow oil (120.4 mg, 0.378 mmol, 76% yield, 1:1.5 d.r., unassigned).

^{1}H NMR (500 MHz, CDCl$_3$) (summary of diastereomers) δ 7.80 (dd, *J* = 7.8, 1.6 Hz, 1H), 7.50 – 7.43 (m, 1H), 7.01 – 6.96 (m, 2H), 4.09 – 4.00 (m, 2H), 3.91 (s, 1.24H), 3.90 (s, 1.72H), 3.07 – 3.04 (m, 0.38H), 2.99 (d, *J* = 5.4 Hz, 0.57H), 2.19 – 2.14 (m, 0.42H), 2.05 – 1.94 (m, 1.31H), 1.91 – 1.78 (m, 1H), 1.71 – 1.64 (m, 0.86H), 1.63 – 1.52 (m, 1.66H), 1.43 – 1.33 (m, 1.33H), 1.30 (s, 3H), 1.23 – 1.14 (m, 0.72H), 0.94 (s, 3H), 0.93 (s, 1.12H), 0.90 (s, 1.69H).

^{13}C NMR (126 MHz, CDCl$_3$) (summary of diastereomers) δ 166.73, 166.55, 159.41, 159.35, 133.60, 131.90, 131.79, 120.46, 120.37, 120.24, 112.11, 112.08, 71.69, 71.64, 61.42, 59.67, 57.76, 57.71, 55.97, 55.94, 39.03, 36.04, 35.69, 35.16, 31.40, 29.96, 26.92, 25.57, 24.60, 23.09, 22.45, 22.16, 22.09, 21.55, 20.09.

IR (film) v_{max} 2962, 1701, 1466, 1303, 1256, 1131, 751 cm^{-1}.

HRMS (ESI-TOF) *m/z* calculated for $C_{19}H_{26}NaO_4^+$ ([M+Na]$^+$) 341.1723, found 341.1724.

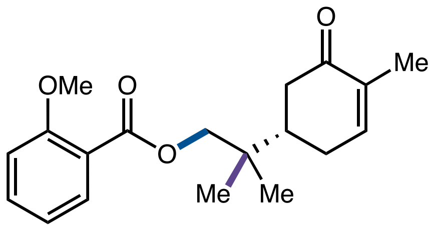

(*S*)-2-methyl-2-(4-methyl-5-oxocyclohex-3-en-1-yl)propyl 2-methoxybenzoate (15): The title compound was prepared according to General Procedure B with Ni(acac)$_2$ (5 mol%, 25 µmol, 6.4 mg), KTp* (5 mol%, 25 µmol, 8.4 mg), (Ir[dF(CF$_3$)ppy]$_2$(dtbbpy))(PF$_6$) (2 mol%, 10 µmol, 11.2 mg), (1,3-dioxoisoindolin-2-yl)acetate (2 equiv, 1 mmol, 205.2 mg), 2-methoxybenzoic acid (1

equiv, 0.5 mmol, 76.1 mg), (*S*)-2-methyl-5-(prop-1-en-2-yl)cyclohex-2-en-1-one (2 equiv, 1 mmol, 150.2 mg, 157.4 µL), *tert*-butylimino-tri(pyrrolidino)phosphorane (0.2 equiv, 0.1 mmol, 31.2 mg, 30.6 µL), and 10 mL *t*-amyl alcohol. The crude reaction mixture was concentrated, dissolved in DMSO/MeCN, and purified by automated reverse-phase chromatography (25 g C18 column, 20-50% gradient of 0.1% ammonium hydroxide in water/0.1% ammonium hydroxide in acetonitrile). Fractions were directly concentrated and the product dissolved in acetonitrile and filtered through a cotton plug into a 40 mL vial. The solvent was removed and the product dried on high-vac to yield the title compound as a yellow oil (95.1 mg, 0.301 mmol, 60% yield).

^{1}H NMR (500 MHz, CDCl$_3$) δ 7.77 (dd, *J* = 7.7, 1.9 Hz, 1H), 7.47 (ddd, *J* = 8.4, 7.4, 1.9 Hz, 1H), 7.00 – 6.95 (m, 2H), 6.79 – 6.74 (m, 1H), 4.13 (d, *J* = 11.1 Hz, 1H), 4.04 (d, *J* = 11.1 Hz, 1H), 3.86 (s, 3H), 2.63 – 2.56 (m, 1H), 2.44 – 2.33 (m, 1H), 2.26 – 2.18 (m, 3H), 1.77 (s, 3H), 1.03 (s, 3H), 0.99 (s, 3H).

^{13}C NMR (126 MHz, CDCl$_3$) δ 200.57, 166.59, 159.31, 145.48, 135.32, 133.75, 131.86, 120.27, 120.14, 112.07, 71.07, 55.90, 41.52, 39.82, 36.01, 27.54, 22.69, 21.66, 15.71.

IR (film) v_{max} 2962, 2841, 1703, 1673, 1302, 1254, 1130, 756 cm^{-1}.

HRMS (ESI-TOF) *m/z* calculated for $C_{19}H_{25}O_4^+$ ([M+H]$^+$) 317.1747, found 317.1744.

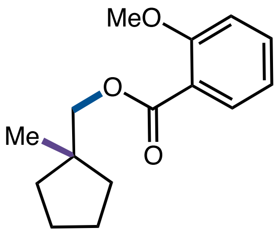

(1-methylcyclopentyl)methyl 2-methoxybenzoate (16): The title compound was prepared according to General Procedure B with Ni(acac)$_2$ (5 mol%, 25 µmol, 6.4 mg), KTp* (5 mol%, 25 µmol, 8.4 mg), (Ir[dF(CF$_3$)ppy]$_2$(dtbbpy))(PF$_6$) (2 mol%, 10 µmol, 11.2 mg), (1,3-dioxoisoindolin-

2-yl)acetate (2 equiv, 1 mmol, 205.2 mg), 2-methoxybenzoic acid (1 equiv, 0.5 mmol, 76.1 mg), methylenecyclopentane (2 equiv, 1 mmol, 82.14 mg, 105.3 µL), *tert*-butylimino-tri(pyrrolidino)phosphorane (0.2 equiv, 0.1 mmol, 31.2 mg, 30.6 µL), and 10 mL *t*-amyl alcohol. The crude reaction mixture was concentrated, dissolved in DMSO/MeCN, and purified by automated reverse-phase chromatography (25 g C18 column, 20-60% gradient of 0.1% ammonium hydroxide in water/0.1% ammonium hydroxide in acetonitrile). Fractions were directly concentrated and the product dissolved in acetonitrile and filtered through a cotton plug into a 40 mL vial. The solvent was removed and the product dried on high-vac to yield the title compound as a yellow oil (83.1 mg, 0.335 mmol, 67% yield).

^{1}H NMR (500 MHz, CDCl$_3$) δ 7.81 (dd, *J* = 7.9, 1.8 Hz, 1H), 7.46 (ddd, *J* = 8.4, 7.4, 1.8 Hz, 1H), 7.01 – 6.95 (m, 2H), 4.08 (s, 2H), 3.90 (s, 3H), 1.71 – 1.59 (m, 6H), 1.43 – 1.35 (m, 2H), 1.11 (s, 3H).

^{13}C NMR (126 MHz, CDCl$_3$) δ 166.71, 159.38, 133.49, 131.77, 120.61, 120.21, 112.13, 72.58, 56.00, 42.65, 36.76, 25.14, 25.08.

IR (film) v_{max} 2957, 2871, 1702, 1302, 1253, 1130, 756 cm^{-1}.

HRMS (ESI-TOF) *m/z* calculated for $C_{15}H_{20}NaO_3^+$ ([M+Na]$^+$) 271.1305, found 271.1300.

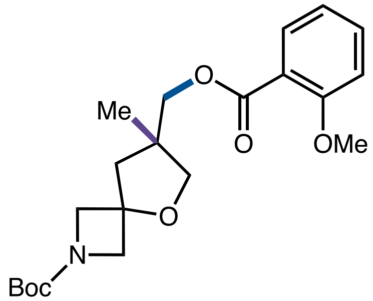

(±)-*tert*-butyl 7-(((2-methoxybenzoyl)oxy)methyl)-7-methyl-5-oxa-2-azaspiro[3.4]octane-2-carboxylate (17): The title compound was prepared according to General Procedure B with

$Ni(acac)_2$ (5 mol%, 25 μmol, 6.4 mg), KTp* (5 mol%, 25 μmol, 8.4 mg), $(Ir[dF(CF_3)ppy]_2(dtbbpy))(PF_6)$ (2 mol%, 10 μmol, 11.2 mg), (1,3-dioxoisoindolin-2-yl)acetate (2 equiv, 1 mmol, 205.2 mg), 2-methoxybenzoic acid (1 equiv, 0.5 mmol, 76.1 mg), *tert*-butyl 7-methylene-5-oxa-2-azaspiro[3.4]octane-2-carboxylate (2 equiv, 1 mmol, 225.3 mg, added before sparge), *tert*-butylimino-tri(pyrrolidino)phosphorane (0.2 equiv, 0.1 mmol, 31.2 mg, 30.6 μL), and 10 mL *t*-amyl alcohol. The crude reaction mixture was concentrated, dissolved in DMSO/MeCN, and purified by automated reverse-phase chromatography (25 g C18 column, 20-35% gradient of 0.1% ammonium hydroxide in water/0.1% ammonium hydroxide in acetonitrile). Fractions were directly concentrated and the product dissolved in acetonitrile and filtered through a cotton plug into a 40 mL vial. The solvent was removed and the product dried on high-vac to yield the title compound as a clear oil (85.5 mg, 0.218 mmol, 44% yield).

^{1}H NMR (500 MHz, $CDCl_3$) δ 7.77 (dd, *J* = 8.0, 1.8 Hz, 1H), 7.51 – 7.45 (m, 1H), 7.02 – 6.95 (m, 2H), 4.17 – 4.09 (m, 2H), 4.08 – 4.00 (m, 2H), 3.96 – 3.90 (m, 2H), 3.89 (s, 3H), 3.86 (d, *J* = 8.7 Hz, 1H), 3.58 (d, *J* = 8.7 Hz, 1H), 2.24 (d, *J* = 13.2 Hz, 1H), 1.98 (d, *J* = 13.2 Hz, 1H), 1.42 (s, 9H), 1.20 (s, 3H).

^{13}C NMR (126 MHz, $CDCl_3$) δ 166.45, 159.34, 156.39, 133.89, 131.78, 120.31, 119.92, 112.12, 79.69, 78.69, 76.50, 69.27, 63.08, 62.88, 55.97, 46.40, 44.02, 28.50, 22.12.

IR (film) v_{max} 2970, 2875, 1697, 1402, 1366, 1301, 1248, 1130, 1074, 913, 729 cm^{-1}.

HRMS (ESI-TOF) *m/z* calculated for $C_{21}H_{29}NNaO_6^+$ ($[M+Na]^+$) 414.1887, found 414.1886.

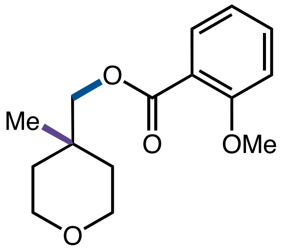

(4-methyltetrahydro-2*H*-pyran-4-yl)methyl 2-methoxybenzoate (18): The title compound was prepared according to General Procedure B with Ni(acac)$_2$ (5 mol%, 25 μmol, 6.4 mg), KTp* (5 mol%, 25 μmol, 8.4 mg), (Ir[dF(CF$_3$)ppy]$_2$(dtbbpy))(PF$_6$) (2 mol%, 10 μmol, 11.2 mg), (1,3-dioxoisoindolin-2-yl)acetate (2 equiv, 1 mmol, 205.2 mg), 2-methoxybenzoic acid (1 equiv, 0.5 mmol, 76.1 mg), 4-methylenetetrahydro-2*H*-pyran (2 equiv, 1 mmol, 98.1 mg, 108.8 μL), *tert*-butylimino-tri(pyrrolidino)phosphorane (0.2 equiv, 0.1 mmol, 31.2 mg, 30.6 μL), and 10 mL *t*-amyl alcohol. The crude reaction mixture was concentrated, dissolved in DMSO/MeCN, and purified by automated reverse-phase chromatography (25 g C18 column, 20-65% gradient of 0.1% ammonium hydroxide in water/0.1% ammonium hydroxide in acetonitrile). Fractions were directly concentrated and the product dissolved in acetonitrile and filtered through a cotton plug into a 40 mL vial. The solvent was removed and the product dried on high-vac to yield the title compound as a yellow oil (123.5 mg, 0.467 mmol, 93% yield).

^{1}H NMR (500 MHz, CDCl$_3$) δ 7.81 (dd, *J* = 7.9, 1.9 Hz, 1H), 7.52 – 7.43 (m, 1H), 7.01 – 6.94 (m, 2H), 4.11 (s, 2H), 3.90 (s, 3H), 3.80 – 3.73 (m, 2H), 3.70 – 3.63 (m, 2H), 1.76 – 1.68 (m, 2H), 1.47 – 1.43 (m, 2H), 1.14 (s, 3H).

^{13}C NMR (126 MHz, CDCl$_3$) δ 166.52, 159.41, 133.71, 131.83, 120.25, 120.24, 112.14, 72.55, 63.84, 55.99, 34.44, 32.11, 22.13.

IR (film) v_{max} 2956, 2874, 1703, 1303, 1254, 1079, 757 cm^{-1}.

HRMS (ESI-TOF) *m/z* calculated for $C_{15}H_{21}O_4^+$ ([M+H]$^+$) 265.1434, found 265.1436.

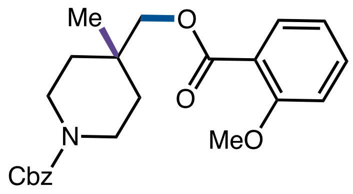

benzyl 4-(((2-methoxybenzoyl)oxy)methyl)-4-methylpiperidine-1-carboxylate (5): The title compound was prepared according to General Procedure B with $Ni(acac)_2$ (5 mol%, 25 µmol, 6.4 mg), KTp* (5 mol%, 25 µmol, 8.4 mg), $(Ir[dF(CF_3)ppy]_2(dtbbpy))(PF_6)$ (2 mol%, 10 µmol, 11.2 mg), (1,3-dioxoisoindolin-2-yl)acetate (2 equiv, 1 mmol, 205.2 mg), 2-methoxybenzoic acid (1 equiv, 0.5 mmol, 76.1 mg), benzyl 4-methylenepiperidine-1-carboxylate (2 equiv, 1 mmol, 231.3 mg, 208.4 µL), *tert*-butylimino-tri(pyrrolidino)phosphorane (0.2 equiv, 0.1 mmol, 31.2 mg, 30.6 µL), and 10 mL *t*-amyl alcohol. The crude reaction mixture was concentrated, dissolved in DMSO/MeCN, and purified by automated reverse-phase chromatography (25 g C18 column, 20-60% gradient of 0.1% ammonium hydroxide in water/0.1% ammonium hydroxide in acetonitrile). Fractions were directly concentrated and the product dissolved in acetonitrile and filtered through a cotton plug into a 40 mL vial. The solvent was removed and the product dried on high-vac to yield the title compound as a yellow oil (148.4 mg, 0.373 mmol, 75% yield).

^{1}H NMR (500 MHz, $CDCl_3$) δ 7.80 (dd, *J* = 7.7, 1.9 Hz, 1H), 7.48 (ddd, *J* = 8.4, 7.4, 1.8 Hz, 1H), 7.38 – 7.28 (m, 5H), 6.98 (ddd, *J* = 9.4, 7.8, 1.5 Hz, 2H), 5.13 (s, 2H), 4.09 (s, 2H), 3.87 (s, 3H), 3.81 – 3.70 (m, 2H), 3.31 (ddd, *J* = 13.5, 9.8, 3.6 Hz, 2H), 1.67 – 1.59 (m, 2H), 1.46 – 1.36 (m, 2H), 1.10 (s, 3H).

^{13}C NMR (126 MHz, $CDCl_3$) δ 166.53, 159.36, 155.46, 137.04, 133.75, 131.84, 128.61, 128.08, 127.97, 120.27, 120.15, 112.12, 72.17, 67.14, 55.93, 40.02, 33.60, 32.94, 21.66.

IR (film) v_{max} 2946, 2878, 1699, 1302, 1251, 1180, 1079, 755 cm^{-1}.

HRMS (ESI-TOF) *m/z* calculated for $C_{23}H_{28}NO_5^+$ ($[M+H]^+$) 398.1962, found 398.1961.

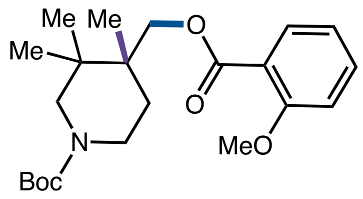

(±)-*tert*-butyl 4-(((2-methoxybenzoyl)oxy)methyl)-3,3,4-trimethylpiperidine-1-carboxylate (19): The title compound was prepared according to General Procedure B with Ni(acac)$_2$ (5 mol%, 25 μmol, 6.4 mg), KTp* (5 mol%, 25 μmol, 8.4 mg), (Ir[dF(CF$_3$)ppy]$_2$(dtbbpy))(PF$_6$) (2 mol%, 10 μmol, 11.2 mg), (1,3-dioxoisoindolin-2-yl)acetate (2 equiv, 1 mmol, 205.2 mg), 2-methoxybenzoic acid (1 equiv, 0.5 mmol, 76.1 mg), *tert*-butyl 3,3-dimethyl-4-methylenepiperidine-1-carboxylate (2 equiv, 1 mmol, 225.3 mg), *tert*-butylimino-tri(pyrrolidino)phosphorane (0.2 equiv, 0.1 mmol, 31.2 mg, 30.6 μL), and 10 mL *t*-amyl alcohol. The crude reaction mixture was concentrated, dissolved in DMSO/MeCN, and purified by automated reverse-phase chromatography (25 g C18 column, 20-100% gradient of 0.1% ammonium hydroxide in water/0.1% ammonium hydroxide in acetonitrile). Additional purification via Preparative HPLC (XBridge BEH C18 OBD column, 20-63% gradient of 0.1% ammonium hydroxide in water/0.1% ammonium hydroxide in acetonitrile). Fractions were directly concentrated and the product dissolved in DCM and filtered through a cotton plug into a 40 mL vial. The solvent was removed and the product dried on high-vac to yield the title compound as a pale yellow oil (140.1 mg, 0.358 mmol, 72% yield).

^{1}H NMR (500 MHz, CDCl$_3$) δ 7.79 (dd, *J* = 7.9, 1.8 Hz, 1H), 7.49 – 7.44 (m, 1H), 7.00 – 6.95 (m, 2H), 4.33 – 4.22 (m, 1H), 4.19 (d, *J* = 11.1 Hz, 1H), 3.88 (s, 3H), 3.56 (b, 1H), 3.37 – 3.29 (m, 1H), 3.29 – 3.20 (m, 1H), 3.15 (d, *J* = 13.6 Hz, 1H), 1.79 – 1.71 (m, 1H), 1.48 – 1.42 (m, 10H), 1.06 (s, 3H), 0.97 (s, 3H), 0.96 (s, 3H).

^{13}C NMR (126 MHz, CDCl$_3$) δ 166.71, 159.31, 155.19, 133.68, 131.79, 120.29, 120.25, 112.09, 79.36, 69.22, 55.89, 52.96, 51.81, 40.69, 39.75, 37.86, 35.95, 30.89, 28.59, 22.61, 21.79, 18.53.

IR (film) v_{max} 2965, 2876, 1719, 1697, 1303, 1255, 1179, 752 cm^{-1}.

HRMS (ESI-TOF) *m/z* calculated for $C_{22}H_{33}NNaO_5^+$ ([M+Na]$^+$) 414.2251, found 414.2251.

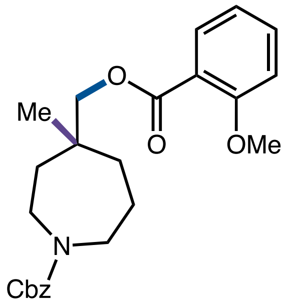

(±)-benzyl 4-(((2-methoxybenzoyl)oxy)methyl)-4-methylazepane-1-carboxylate (20): The title compound was prepared according to General Procedure B with Ni(acac)$_2$ (5 mol%, 25 µmol, 6.4 mg), KTp* (5 mol%, 25 µmol, 8.4 mg), (Ir[dF(CF$_3$)ppy]$_2$(dtbbpy))(PF$_6$) (2 mol%, 10 µmol, 11.2 mg), (1,3-dioxoisoindolin-2-yl)acetate (2 equiv, 1 mmol, 205.2 mg), 2-methoxybenzoic acid (1 equiv, 0.5 mmol, 76.1 mg), benzyl 4-methyleneazepane-1-carboxylate (2 equiv, 1 mmol, 245.3 mg, added before sparge), *tert*-butylimino-tri(pyrrolidino)phosphorane (0.2 equiv, 0.1 mmol, 31.2 mg, 30.6 µL), and 10 mL *t*-amyl alcohol. The crude reaction mixture was concentrated, dissolved in DMSO/MeCN, and purified by automated reverse-phase chromatography (25 g C18 column, 20-45% gradient of 0.1% ammonium hydroxide in water/0.1% ammonium hydroxide in acetonitrile). Fractions were directly concentrated and the product dissolved in acetonitrile and filtered through a cotton plug into a 40 mL vial. The solvent was removed and the product dried on high-vac to yield the title compound as a yellow oil (113.3 mg, 0.275 mmol, 55% yield).

^{1}H NMR (500 MHz, CDCl$_3$) (summary of rotamers) δ 7.79 (ddd, *J* = 7.7, 3.8, 1.8 Hz, 1H), 7.51 – 7.44 (m, 1H), 7.39 – 7.27 (m, 5H), 7.01 – 6.94 (m, 2H), 5.14 (d, *J* = 3.9 Hz, 2H), 4.06 – 4.00 (m, 2H), 3.87 (d, *J* = 5.0 Hz, 3H), 3.64 – 3.34 (m, 4H), 1.81 – 1.70 (m, 3H), 1.67 – 1.50 (m, 3H), 1.03 (d, *J* = 6.3 Hz, 3H).

^{13}C NMR (126 MHz, $CDCl_3$) (summary of rotamers) δ 166.56, 166.54, 159.35, 156.19, 156.14, 137.19, 137.15, 133.69, 133.66, 131.82, 131.81, 128.58, 127.99, 127.88, 120.28, 120.24, 112.12, 73.11, 73.06, 67.10, 67.06, 55.93, 46.43, 46.24, 42.41, 42.07, 36.91, 36.77, 36.74, 36.58, 36.02, 35.80, 23.52, 23.21, 23.13, 22.86.

IR (film) v_{max} 2967, 2879, 1724, 1696, 1303, 1328, 1258, 1131, 750 cm^{-1}.

HRMS (ESI-TOF) *m/z* calculated for $C_{24}H_{30}NO_5^+$ ($[M+H]^+$) 412.2118, found 412.2120.

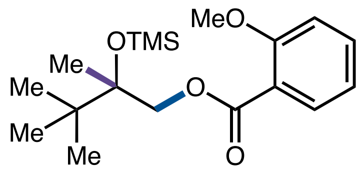

(±)-2,3,3-trimethyl-2-((trimethylsilyl)oxy)butyl 2-methoxybenzoate (21): The title compound was prepared according to General Procedure B with $Ni(acac)_2$ (5 mol%, 25 μmol, 6.4 mg), KTp* (5 mol%, 25 μmol, 8.4 mg), $(Ir[dF(CF_3)ppy]_2(dtbbpy))(PF_6)$ (2 mol%, 10 μmol, 11.2 mg), (1,3-dioxoisoindolin-2-yl)acetate (2 equiv, 1 mmol, 205.2 mg), 2-methoxybenzoic acid (1 equiv, 0.5 mmol, 76.1 mg), ((3,3-dimethylbut-1-en-2-yl)oxy)trimethylsilane (2 equiv, 1 mmol, 172.3 mg, 216.0 μL), *tert*-butylimino-tri(pyrrolidino)phosphorane (0.2 equiv, 0.1 mmol, 31.2 mg, 30.6 μL), and 10 mL *t*-amyl alcohol. The crude reaction mixture was concentrated, dissolved in DMSO/MeCN, and purified by automated reverse-phase chromatography (25 g C18 column, 20-77% gradient of 0.1% ammonium hydroxide in water/0.1% ammonium hydroxide in acetonitrile). Fractions were directly concentrated and the product dissolved in acetonitrile and filtered through a cotton plug into a 40 mL vial. The solvent was removed and the product dried on high-vac to yield the title compound as a yellow oil (141.7 mg, 0.419 mmol, 84% yield).

^{1}H NMR (500 MHz, $CDCl_3$) δ 7.80 (dd, J = 8.1, 1.8 Hz, 1H), 7.50 – 7.44 (m, 1H), 7.01 – 6.96 (m, 2H), 4.38 (d, J = 11.3 Hz, 1H), 4.22 (d, J = 11.3 Hz, 1H), 3.89 (s, 3H), 1.31 (s, 3H), 0.97 (s, 9H), 0.06 (s, 9H).

^{13}C NMR (126 MHz, $CDCl_3$) δ 166.64, 159.15, 133.49, 131.61, 120.61, 120.17, 111.95, 79.08, 69.95, 55.85, 38.22, 25.81, 19.92, 2.46.

IR (film) v_{max} 2959, 2877, 1700, 1303, 1257, 1130, 752 cm^{-1}.

HRMS (ESI-TOF) *m/z* calculated for $C_{18}H_{31}O_4Si^+$ ($[M+H]^+$) 339.1986, found 339.1980.

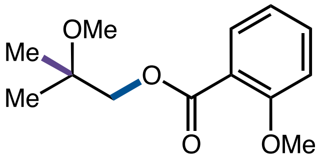

2-methoxy-2-methylpropyl 2-methoxybenzoate (22): The title compound was prepared according to General Procedure B with $Ni(acac)_2$ (5 mol%, 25 µmol, 6.4 mg), KTp* (5 mol%, 25 µmol, 8.4 mg), $(Ir[dF(CF_3)ppy]_2(dtbbpy))(PF_6)$ (2 mol%, 10 µmol, 11.2 mg), (1,3-dioxoisoindolin-2-yl)acetate (2 equiv, 1 mmol, 205.2 mg), 2-methoxybenzoic acid (1 equiv, 0.5 mmol, 76.1 mg), 2-methoxyprop-1-ene (2 equiv, 1 mmol, 72.1 mg, 95.8 µL), *tert*-butylimino-tri(pyrrolidino)phosphorane (0.2 equiv, 0.1 mmol, 31.2 mg, 30.6 µL), and 10 mL *t*-amyl alcohol. The crude reaction mixture was concentrated, dissolved in DMSO/MeCN, and purified by automated reverse-phase chromatography (25 g C18 column, 20-35% gradient of 0.1% ammonium hydroxide in water/0.1% ammonium hydroxide in acetonitrile). Fractions were directly concentrated and the product dissolved in acetonitrile and filtered through a cotton plug into a 40 mL vial. The solvent was removed and the product dried on high-vac to yield the title compound as a yellow oil (91.1 mg, 0.382 mmol, 76% yield).

^{1}H NMR (500 MHz, CDCl$_3$) δ 7.83 (dd, *J* = 8.0, 1.8 Hz, 1H), 7.47 (ddd, *J* = 8.3, 7.4, 1.8 Hz, 1H), 7.01 – 6.95 (m, 2H), 4.22 (s, 2H), 3.90 (s, 3H), 3.29 (s, 3H), 1.28 (s, 6H).

^{13}C NMR (126 MHz, CDCl$_3$) δ 166.18, 159.45, 133.71, 131.92, 120.25, 120.15, 112.11, 74.02, 69.35, 56.01, 50.11, 22.71.

IR (film) v_{max} 2973, 2839, 1703, 1304, 1256, 1131, 1081, 754 cm^{-1}.

HRMS (ESI-TOF) *m/z* calculated for $C_{13}H_{18}NaO_4^+$ ([M+Na]$^+$) 261.1097, found 261.1096.

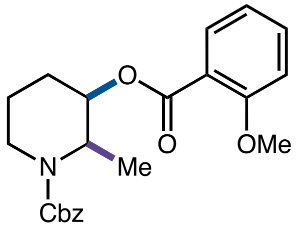

(±)-benzyl 3-((2-methoxybenzoyl)oxy)-2-methylpiperidine-1-carboxylate (23): The title compound was prepared according to General Procedure B with Ni(acac)$_2$ (5 mol%, 25 μmol, 6.4 mg), KTp* (5 mol%, 25 μmol, 8.4 mg), (Ir[dF(CF$_3$)ppy]$_2$(dtbbpy))(PF$_6$) (2 mol%, 10 μmol, 11.2 mg), (1,3-dioxoisoindolin-2-yl)acetate (2 equiv, 1 mmol, 205.2 mg), 2-methoxybenzoic acid (1 equiv, 0.5 mmol, 76.1 mg), benzyl 3,4-dihydropyridine-1(2*H*)-carboxylate (2 equiv, 1 mmol, 217.3 mg, 194.0 μL), *tert*-butylimino-tri(pyrrolidino)phosphorane (0.2 equiv, 0.1 mmol, 31.2 mg, 30.6 μL), and 10 mL *t*-amyl alcohol. The crude reaction mixture was concentrated, dissolved in DMSO/MeCN, and purified by automated reverse-phase chromatography (25 g C18 column, 20-100% gradient of 0.1% ammonium hydroxide in water/0.1% ammonium hydroxide in acetonitrile). Further purification via preparative SFC (ColumnTek EnantioCell® A5-5 (30 x 250 mm); 85 mL/min at 20% EtOH / 80% CO_2 (100 bar backpressure)) to afford the two diastereomers. Fractions were directly concentrated and the products dissolved in acetonitrile and filtered through a cotton plug into a 40 mL vial. The solvent was removed and the products dried on high-vac to

yield the title compound as a yellow oil (130.9 mg (66.0 mg and 64.9 mg), 0.341 mmol, 68% yield, 1:1 d.r., unassigned).

^{1}H NMR (500 MHz, CDCl$_3$) (diastereomer 1) δ 7.76 (dd, *J* = 7.9, 1.9 Hz, 1H), 7.46 (ddd, *J* = 8.4, 7.3, 1.8 Hz, 1H), 7.39 – 7.26 (m, 1H), 7.22 (b, 4H), 6.97 – 6.90 (m, 2H), 5.14 – 4.95 (m, 3H), 4.69 – 4.60 (m, 1H), 4.25 – 4.08 (m, 1H), 3.83 (s, 3H), 2.99 (td, *J* = 13.1, 2.9 Hz, 1H), 1.99 – 1.84 (m, 3H), 1.56 – 1.48 (m, 1H), 1.26 (d, *J* = 7.2 Hz, 3H).

^{13}C NMR (126 MHz, CDCl$_3$) (diastereomer 1) δ 165.50, 159.57, 155.84, 137.01, 133.70, 131.97, 128.45, 127.82, 127.60, 120.22, 112.14, 71.25, 66.96, 55.97, 50.39, 38.52, 23.59, 20.12, 14.82.

^{1}H NMR (500 MHz, CDCl$_3$) (diastereomer 2) δ 7.76 (dd, *J* = 7.9, 1.9 Hz, 1H), 7.46 (ddd, *J* = 8.4, 7.4, 1.8 Hz, 1H), 7.39 – 7.27 (m, 1H), 7.22 (b, 4H), 6.97 – 6.90 (m, 2H), 5.13 – 4.97 (m, 3H), 4.68 – 4.60 (m, 1H), 4.23 – 4.10 (m, 1H), 3.83 (s, 3H), 2.99 (td, *J* = 13.1, 2.8 Hz, 1H), 1.98 – 1.83 (m, 3H), 1.56 – 1.48 (m, 1H), 1.26 (d, *J* = 7.2 Hz, 3H).

^{13}C NMR (126 MHz, CDCl$_3$) (diastereomer 2) δ 165.50, 159.58, 155.84, 137.02, 133.70, 131.98, 128.45, 127.82, 127.60, 120.22, 112.15, 71.25, 66.96, 55.97, 50.40, 38.53, 23.61, 20.13, 14.83.

IR (film) v_{max} 2971, 2839, 1726, 1699, 1304, 1257, 1131, 1081, 752 cm^{-1}.

HRMS (ESI-TOF) *m/z* calculated for $C_{22}H_{26}NO_5^+$ ([M+H]$^+$) 384.1805, found 384.1805.

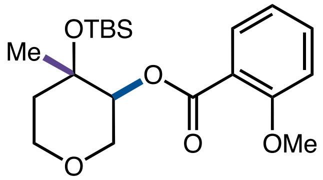

(±)-4-((*tert*-butyldimethylsilyl)oxy)-4-methyltetrahydro-2*H*-pyran-3-yl 2-methoxybenzoate (24): The title compound was prepared according to General Procedure B with Ni(acac)$_2$ (5 mol%, 25 μmol, 6.4 mg), KTp* (5 mol%, 25 μmol, 8.4 mg), (Ir[dF(CF$_3$)ppy]$_2$(dtbbpy))(PF$_6$) (2 mol%, 10

μmol, 11.2 mg), (1,3-dioxoisoindolin-2-yl)acetate (2 equiv, 1 mmol, 205.2 mg), 2-methoxybenzoic acid (1 equiv, 0.5 mmol, 76.1 mg), *tert*-butyl((3,6-dihydro-2*H*-pyran-4-yl)oxy)dimethylsilane (2 equiv, 1 mmol, 214.4 mg), *tert*-butylimino-tri(pyrrolidino)phosphorane (0.2 equiv, 0.1 mmol, 31.2 mg, 30.6 μL), and 10 mL *t*-amyl alcohol. The crude reaction mixture was concentrated, dissolved in DMSO/MeCN, and purified by automated reverse-phase chromatography (25 g C18 column, 20-75% gradient of 0.1% ammonium hydroxide in water/0.1% ammonium hydroxide in acetonitrile). Fractions were directly concentrated and the product dissolved in acetonitrile and filtered through a cotton plug into a 40 mL vial. The solvent was removed and the product dried on high-vac to yield the title compound as a yellow oil (122.5 mg, 0.322 mmol, 64% yield, 1:4 d.r., unassigned).

^{1}H NMR (500 MHz, $CDCl_3$) (summary of diastereomers) δ 7.87 – 7.81 (m, 1H), 7.51 – 7.44 (m, 1H), 7.00 – 6.94 (m, 2H), 4.91 (dd, *J* = 9.8, 4.8 Hz, 0.81H), 4.75 (q, *J* = 1.8 Hz, 0.18H), 4.03 (dd, *J* = 12.4, 1.7 Hz, 0.21H), 3.90 (s, 0.58H), 3.90 (s, 2.42H), 3.86 – 3.79 (m, 2H), 3.75 – 3.66 (m, 1.80H), 2.08 – 2.00 (m, 0.26H), 1.84 – 1.77 (m, 0.86H), 1.75 – 1.70 (m, 0.82H), 1.51 (dd, *J* = 13.6, 2.1 Hz, 0.18H), 1.35 (s, 2.37H), 1.34 (s, 0.60H), 0.92 (s, 1.78H), 0.92 (s, 7.20H), 0.16 (s, 0.54H), 0.14 (s, 0.54H), 0.07 (s, 2.40H), 0.07 (s, 2.39H).

^{13}C NMR (126 MHz, $CDCl_3$) (summary of diastereomers) δ 165.55, 165.42, 159.60, 159.52, 133.85, 133.75, 132.07, 131.66, 120.26, 120.03, 120.01, 119.95, 112.13, 111.99, 74.52, 74.02, 72.17, 71.24, 66.10, 64.81, 63.62, 63.56, 56.00, 55.98, 40.18, 36.57, 26.56, 26.29, 26.09, 18.66, 18.45, -1.70, -1.81, -1.89, -2.01.

IR (film) v_{max} 2956, 2857, 1726, 1699, 1303, 1258, 1082, 752 cm^{-1}.

HRMS (ESI-TOF) *m/z* calculated for $C_{20}H_{32}NaO_5Si^+$ ($[M+Na]^+$) 403.1911, found 403.1914.

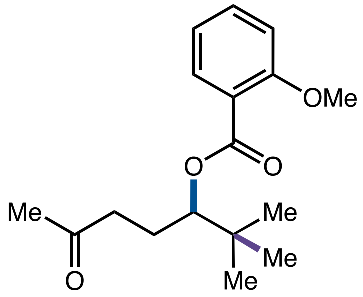

(±)-2,2-dimethyl-6-oxoheptan-3-yl 2-methoxybenzoate (25): The title compound was prepared according to General Procedure B with Ni(acac)$_2$ (5 mol%, 25 μmol, 6.4 mg), KTp* (5 mol%, 25 μmol, 8.4 mg), (Ir[dF(CF$_3$)ppy]$_2$(dtbbpy))(PF$_6$) (2 mol%, 10 μmol, 11.2 mg), (1,3-dioxoisoindolin-2-yl)acetate (2 equiv, 1 mmol, 205.2 mg), 2-methoxybenzoic acid (1 equiv, 0.5 mmol, 76.1 mg), 6-methylhept-5-en-2-one (2 equiv, 1 mmol, 126.2 mg, 147.6 μL), *tert*-butylimino-tri(pyrrolidino)phosphorane (0.2 equiv, 0.1 mmol, 31.2 mg, 30.6 μL), and 10 mL *t*-amyl alcohol. The crude reaction mixture was concentrated, dissolved in DMSO/MeCN, and purified by automated reverse-phase chromatography (25 g C18 column, 20-55% gradient of 0.1% ammonium hydroxide in water/0.1% ammonium hydroxide in acetonitrile). Fractions were directly concentrated and the product dissolved in acetonitrile and filtered through a cotton plug into a 40 mL vial. The solvent was removed and the product dried on high-vac to yield the title compound as a yellow oil (128.8 mg, 0.441 mmol, 88% yield).

^{1}H NMR (500 MHz, CDCl$_3$) δ 7.80 (dd, J = 7.9, 1.8 Hz, 1H), 7.47 (ddd, J = 8.4, 7.4, 1.8 Hz, 1H), 7.03 – 6.96 (m, 2H), 4.94 (dd, J = 11.1, 2.1 Hz, 1H), 3.90 (s, 3H), 2.57 – 2.48 (m, 2H), 2.11 (s, 3H), 2.05 – 1.98 (m, 1H), 1.81 – 1.74 (m, 1H), 0.99 (s, 9H).

^{13}C NMR (126 MHz, CDCl$_3$) δ 208.53, 166.57, 159.33, 133.54, 131.57, 120.59, 120.30, 112.20, 80.72, 55.97, 40.61, 35.07, 30.29, 26.12, 23.84.

IR (film) v_{max} 2965, 2873, 1716, 1301, 1256, 1131, 756 cm^{-1}.

HRMS (ESI-TOF) *m/z* calculated for $C_{17}H_{24}NaO_4^+$ ([M+Na]$^+$) 315.1567, found 315.1561.

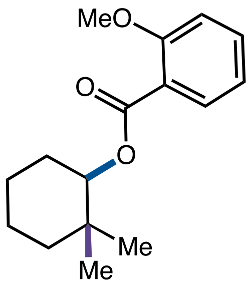

(±)-5-(2-acetoxypropan-2-yl)-2,2-dimethylcyclohexyl 2-methoxybenzoate (26): The title compound was prepared according to General Procedure B with Ni(acac)$_2$ (5 mol%, 25 µmol, 6.4 mg), KTp* (5 mol%, 25 µmol, 8.4 mg), (Ir[dF(CF$_3$)ppy]$_2$(dtbbpy))(PF$_6$) (2 mol%, 10 µmol, 11.2 mg), (1,3-dioxoisoindolin-2-yl)acetate (2 equiv, 1 mmol, 205.2 mg), 2-methoxybenzoic acid (1 equiv, 0.5 mmol, 76.1 mg), 1-methylcyclohex-1-ene (2 equiv, 1 mmol, 96.2 mg, 118.6 µL), *tert*-butylimino-tri(pyrrolidino)phosphorane (0.2 equiv, 0.1 mmol, 31.2 mg, 30.6 µL), and 10 mL *t*-amyl alcohol. The crude reaction mixture was concentrated, dissolved in DMSO/MeCN, and purified by automated reverse-phase chromatography (25 g C18 column, 20-75% gradient of 0.1% ammonium hydroxide in water/0.1% ammonium hydroxide in acetonitrile). Fractions were directly concentrated and the product dissolved in acetonitrile and filtered through a cotton plug into a 40 mL vial. The solvent was removed and the product dried on high-vac to yield the title compound as a dark yellow oil (116.3 mg, 0.443 mmol, 89% yield).

^{1}H NMR (500 MHz, CDCl$_3$) δ 7.81 (dd, *J* = 8.0, 1.8 Hz, 1H), 7.46 (ddd, *J* = 8.3, 7.4, 1.9 Hz, 1H), 7.01 – 6.94 (m, 2H), 4.83 (dd, *J* = 9.4, 3.9 Hz, 1H), 3.90 (s, 3H), 1.89 – 1.82 (m, 1H), 1.74 – 1.68 (m, 1H), 1.63 – 1.53 (m, 2H), 1.50 – 1.38 (m, 3H), 1.34 – 1.28 (m, 1H), 1.01 (d, *J* = 10.2 Hz, 6H).

^{13}C NMR (126 MHz, CDCl$_3$) δ 166.04, 159.37, 133.34, 131.71, 121.03, 120.18, 112.13, 79.50, 55.97, 38.29, 34.74, 28.01, 27.35, 23.89, 21.60, 21.52.

IR (film) v_{max} 2940, 2865, 1700, 1300, 1252, 1130, 1079, 755.

HRMS (ESI-TOF) *m/z* calculated for $C_{16}H_{22}NaO_3^+$ ([M+Na]$^+$) 285.1461, found 285.1459.

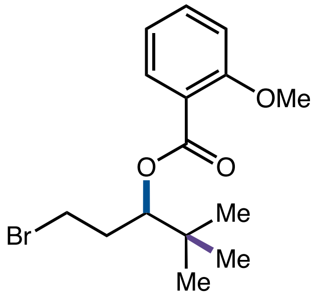

(±)-1-bromo-4,4-dimethylpentan-3-yl 2-methoxybenzoate (27): The title compound was prepared according to General Procedure B with Ni(acac)$_2$ (5 mol%, 25 μmol, 6.4 mg), KTp* (5 mol%, 25 μmol, 8.4 mg), (Ir[dF(CF$_3$)ppy]$_2$(dtbbpy))(PF$_6$) (2 mol%, 10 μmol, 11.2 mg), (1,3-dioxoisoindolin-2-yl)acetate (2 equiv, 1 mmol, 205.2 mg), 2-methoxybenzoic acid (1 equiv, 0.5 mmol, 76.1 mg), 5-bromo-2-methylpent-2-ene (2 equiv, 1 mmol, 163.1 mg, 133.6 μL), *tert*-butylimino-tri(pyrrolidino)phosphorane (0.2 equiv, 0.1 mmol, 31.2 mg, 30.6 μL), and 10 mL *t*-amyl alcohol. The crude reaction mixture was concentrated, dissolved in DMSO/MeCN, and purified by automated reverse-phase chromatography (25 g C18 column, 20-72% gradient of 0.1% ammonium hydroxide in water/0.1% ammonium hydroxide in acetonitrile). Fractions were directly concentrated and the product dissolved in acetonitrile and filtered through a cotton plug into a 40 mL vial. The solvent was removed and the product dried on high-vac to yield the title compound as a yellow oil (94.5 mg, 0.287 mmol, 57% yield).

^{1}H NMR (500 MHz, CDCl$_3$) δ 7.78 (dd, *J* = 7.9, 1.8 Hz, 1H), 7.48 (ddd, *J* = 9.2, 7.6, 1.8 Hz, 1H), 7.02 – 6.96 (m, 2H), 5.06 (dd, *J* = 9.8, 2.7 Hz, 1H), 3.90 (s, 3H), 3.49 – 3.36 (m, 2H), 2.27 – 2.12 (m, 2H), 1.00 (s, 9H).

^{13}C NMR (126 MHz, CDCl$_3$) δ 166.51, 159.23, 133.58, 131.55, 120.51, 120.31, 112.16, 79.75, 55.97, 35.02, 34.40, 30.15, 26.08.

IR (film) v_{max} 2964, 2872, 1700, 1296, 1246, 1129, 1070, 753 cm^{-1}.

HRMS (ESI-TOF) *m/z* calculated for $C_{15}H_{21}BrNaO_3^+$ ([M+Na]$^+$) 351.0566, found 351.0562.

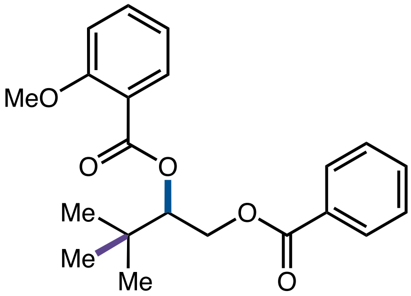

(±)-1-(benzoyloxy)-3,3-dimethylbutan-2-yl 2-methoxybenzoate (28): The title compound was prepared according to General Procedure B with $Ni(acac)_2$ (5 mol%, 25 μmol, 6.4 mg), KTp* (5 mol%, 25 μmol, 8.4 mg), $(Ir[dF(CF_3)ppy]_2(dtbbpy))(PF_6)$ (2 mol%, 10 μmol, 11.2 mg), (1,3-dioxoisoindolin-2-yl)acetate (2 equiv, 1 mmol, 205.2 mg), 2-methoxybenzoic acid (1 equiv, 0.5 mmol, 76.1 mg), 3-methylbut-2-en-1-yl benzoate (2 equiv, 1 mmol, 190.2 mg, 184.7 μL), *tert*-butylimino-tri(pyrrolidino)phosphorane (0.2 equiv, 0.1 mmol, 31.2 mg, 30.6 μL), and 10 mL *t*-amyl alcohol. The crude reaction mixture was concentrated, dissolved in DMSO/MeCN, and purified by automated reverse-phase chromatography (25 g C18 column, 20-60% gradient of 0.1% ammonium hydroxide in water/0.1% ammonium hydroxide in acetonitrile). Fractions were directly concentrated and the product dissolved in acetonitrile and filtered through a cotton plug into a 40 mL vial. The solvent was removed and the product dried on high-vac to yield the title compound as a yellow oil (94.3 mg, 0.265 mmol, 53% yield).

^{1}H NMR (500 MHz, $CDCl_3$) δ 8.01 (dd, *J* = 8.3, 1.4 Hz, 2H), 7.74 (dd, *J* = 7.9, 1.8 Hz, 1H), 7.54 – 7.48 (m, 1H), 7.46 – 7.35 (m, 3H), 6.94 (t, *J* = 7.6 Hz, 2H), 5.41 (dd, *J* = 9.0, 2.7 Hz, 1H), 4.64 (dd, *J* = 11.6, 2.7 Hz, 1H), 4.43 (dd, *J* = 11.7, 9.0 Hz, 1H), 3.82 (s, 3H), 1.11 (s, 9H).

^{13}C NMR (126 MHz, $CDCl_3$) δ 166.63, 166.15, 159.04, 133.32, 133.00, 131.33, 130.03, 129.83, 128.39, 120.80, 120.17, 112.03, 77.97, 64.33, 55.81, 33.97, 26.31.

IR (film) v_{max} 2966, 2874, 1721, 1301, 1258, 1129, 752 cm^{-1}.

HRMS (ESI-TOF) *m/z* calculated for $C_{21}H_{25}O_5^+$ ($[M+H]^+$) 357.1697, found 357.1692.

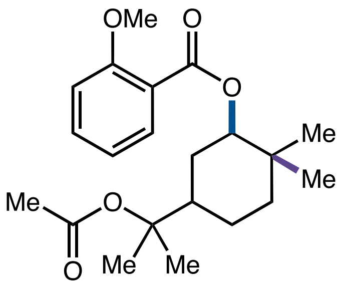

(±)-5-(2-acetoxypropan-2-yl)-2,2-dimethylcyclohexyl 2-methoxybenzoate (29): The title compound was prepared according to General Procedure B with $Ni(acac)_2$ (5 mol%, 25 μmol, 6.4 mg), KTp* (5 mol%, 25 μmol, 8.4 mg), $(Ir[dF(CF_3)ppy]_2(dtbbpy))(PF_6)$ (2 mol%, 10 μmol, 11.2 mg), (1,3-dioxoisoindolin-2-yl)acetate (2 equiv, 1 mmol, 205.2 mg), 2-methoxybenzoic acid (1 equiv, 0.5 mmol, 76.1 mg), 2-(4-methylcyclohex-3-en-1-yl)propan-2-yl acetate (2 equiv, 1 mmol, 196.3 mg, 203.0 μL), *tert*-butylimino-tri(pyrrolidino)phosphorane (0.2 equiv, 0.1 mmol, 31.2 mg, 30.6 μL), and 10 mL *t*-amyl alcohol. The crude reaction mixture was concentrated, dissolved in DMSO/MeCN, and purified by automated reverse-phase chromatography (25 g C18 column, 20-60% gradient of 0.1% ammonium hydroxide in water/0.1% ammonium hydroxide in acetonitrile). Additional purification via Preparative HPLC (XBridge BEH C18 OBD column, 20-70% gradient of 0.1% ammonium hydroxide in water/0.1% ammonium hydroxide in acetonitrile). Fractions were directly concentrated and the product dissolved in acetonitrile and filtered through a cotton plug into a 40 mL vial. The solvent was removed and the product dried on high-vac to yield the title compound as a yellow oil (115.5 mg, 0.319 mmol, 64% yield, 1:1.3 d.r., unassigned). A small amount of each diastereomer was resolved during purification, allowing the individual compounds to be characterized.

^{1}H NMR (500 MHz, $CDCl_3$) (diastereomer 1) δ 7.81 (dd, J = 7.7, 1.8 Hz, 1H), 7.46 (ddd, J = 8.4, 7.4, 1.8 Hz, 1H), 7.02 – 6.96 (m, 2H), 5.01 (t, J = 3.7 Hz, 1H), 3.90 (s, 3H), 2.17 (tt, J = 12.5, 3.6

Hz, 1H), 1.94 (s, 3H), 1.93 – 1.88 (m, 1H), 1.72 (td, J = 13.8, 4.2 Hz, 1H), 1.66 – 1.58 (m, 2H), 1.42 (s, 3H), 1.41 (s, 3H), 1.40 – 1.32 (m, 2H), 1.02 (s, 3H), 0.95 (s, 3H).

^{13}C NMR (126 MHz, CDCl$_3$) (diastereomer 1) δ 170.49, 165.96, 159.31, 133.34, 131.65, 120.99, 120.25, 112.09, 84.46, 77.56, 55.95, 40.73, 34.16, 33.61, 27.62, 27.27, 24.50, 23.58, 23.56, 22.57, 22.14.

^{1}H NMR (500 MHz, CDCl$_3$) (diastereomer 2) δ 7.82 (dd, J = 7.9, 1.8 Hz, 1H), 7.46 (ddd, J = 8.4, 7.3, 1.8 Hz, 1H), 7.01 – 6.95 (m, 2H), 4.86 (dd, J = 11.6, 4.4 Hz, 1H), 3.90 (s, 3H), 2.01 – 1.90 (m, 5H), 1.59 – 1.50 (m, 2H), 1.48 – 1.40 (m, 7H), 1.40 – 1.28 (m, 2H), 1.04 (s, 3H), 0.99 (s, 3H).

^{13}C NMR (126 MHz, CDCl$_3$) (diastereomer 2) δ 170.55, 165.86, 159.44, 133.53, 131.79, 120.66, 120.20, 112.12, 84.17, 79.72, 55.95, 45.78, 38.72, 34.68, 28.85, 28.19, 23.62, 23.61, 22.62, 21.93, 18.97.

IR (film) v_{max} 2961, 2873, 1725, 1302, 1256, 1130, 756 cm^{-1}.

HRMS (ESI-TOF) *m/z* calculated for $C_{21}H_{30}NaO_5^+$ ([M+Na]$^+$) 385.1985, found 385.1986.

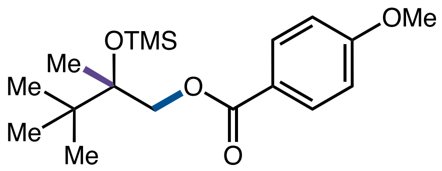

(±)-2,3,3-trimethyl-2-((trimethylsilyl)oxy)butyl 4-methoxybenzoate (30): The title compound was prepared according to General Procedure B with Ni(acac)$_2$ (5 mol%, 25 μmol, 6.4 mg), KTp* (5 mol%, 25 μmol, 8.4 mg), (Ir[dF(CF$_3$)ppy]$_2$(dtbbpy))(PF$_6$) (2 mol%, 10 μmol, 11.2 mg), (1,3-dioxoisoindolin-2-yl)acetate (2 equiv, 1 mmol, 205.2 mg), 4-methoxybenzoic acid (1 equiv, 0.5 mmol, 76.1 mg), ((3,3-dimethylbut-1-en-2-yl)oxy)trimethylsilane (2 equiv, 1 mmol, 172.3 mg, 216.0 μL), *tert*-butylimino-tri(pyrrolidino)phosphorane (0.2 equiv, 0.1 mmol, 31.2 mg, 30.6 μL), and 10 mL *t*-amyl alcohol. The crude reaction mixture was concentrated, dissolved in

DMSO/MeCN, and purified by automated reverse-phase chromatography (25 g C18 column, 40-100% gradient of 0.1% ammonium hydroxide in water/0.1% ammonium hydroxide in acetonitrile). Fractions were directly concentrated and the product dissolved in acetonitrile and filtered through a cotton plug into a 40 mL vial. The solvent was removed and the product dried on high-vac to yield the title compound as a dark yellow oil (148.6 mg, 0.439 mmol, 88% yield).

^{1}H NMR (500 MHz, CDCl$_3$) δ 8.06 – 7.96 (m, 2H), 6.98 – 6.87 (m, 2H), 4.36 (d, J = 11.3 Hz, 1H), 4.26 (d, J = 11.4 Hz, 1H), 3.86 (s, 3H), 1.31 (s, 3H), 0.98 (s, 9H), 0.11 (s, 9H).

^{13}C NMR (126 MHz, CDCl$_3$) δ 166.58, 163.47, 131.78, 122.95, 113.77, 79.16, 69.76, 55.55, 38.29, 25.84, 20.08, 2.60.

IR (film) v_{max} 2957, 2839, 1714, 1256, 1167, 770 cm^{-1}.

HRMS (ESI-TOF) *m/z* calculated for $C_{15}H_{21}O_3^+$ ([M-OTMS]$^+$) 249.1485, found 249.1486.

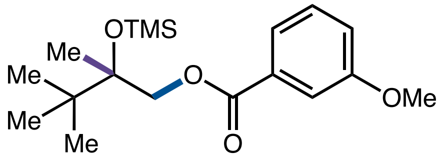

(±)-2,3,3-trimethyl-2-((trimethylsilyl)oxy)butyl 3-methoxybenzoate (31): The title compound was prepared according to General Procedure B with Ni(acac)$_2$ (5 mol%, 25 µmol, 6.4 mg), KTp* (5 mol%, 25 µmol, 8.4 mg), (Ir[dF(CF$_3$)ppy]$_2$(dtbbpy))(PF$_6$) (2 mol%, 10 µmol, 11.2 mg), (1,3-dioxoisoindolin-2-yl)acetate (2 equiv, 1 mmol, 205.2 mg), 3-methoxybenzoic acid (1 equiv, 0.5 mmol, 76.1 mg), ((3,3-dimethylbut-1-en-2-yl)oxy)trimethylsilane (2 equiv, 1 mmol, 172.3 mg, 216.0 µL), *tert*-butylimino-tri(pyrrolidino)phosphorane (0.2 equiv, 0.1 mmol, 31.2 mg, 30.6 µL), and 10 mL *t*-amyl alcohol. The crude reaction mixture was concentrated, dissolved in DMSO/MeCN, and purified by automated reverse-phase chromatography (25 g C18 column, 40-100% gradient of 0.1% ammonium hydroxide in water/0.1% ammonium hydroxide in

acetonitrile). Fractions were directly concentrated and the product dissolved in acetonitrile and filtered through a cotton plug into a 40 mL vial. The solvent was removed and the product dried on high-vac to yield the title compound as a dark yellow oil with residual BTTP (104.9 mg, 94.3% pure; 98.9 mg adjusted mass, 0.292 mmol, 58% yield).

^{1}H NMR (500 MHz, CDCl$_3$) δ 7.66 (dt, *J* = 7.6, 1.3 Hz, 1H), 7.61 – 7.58 (m, 1H), 7.36 (t, *J* = 7.9 Hz, 1H), 7.11 (ddd, *J* = 8.3, 2.7, 1.0 Hz, 1H), 4.40 (d, *J* = 11.3 Hz, 1H), 4.28 (d, *J* = 11.4 Hz, 1H), 3.85 (s, 3H), 1.31 (s, 3H), 0.99 (s, 9H), 0.12 (s, 9H).

^{13}C NMR (126 MHz, CDCl$_3$) δ 166.72, 159.72, 131.84, 129.54, 122.21, 119.67, 114.17, 79.14, 70.17, 55.56, 38.31, 31.83, 20.07, 2.62.

IR (film) v_{max} 2957, 2854, 1721, 1276, 1153, 1041, 754 cm^{-1}.

HRMS (ESI-TOF) *m/z* calculated for $C_{15}H_{21}O_3^+$ ([M-OTMS]$^+$) 249.1485, found 249.1487.

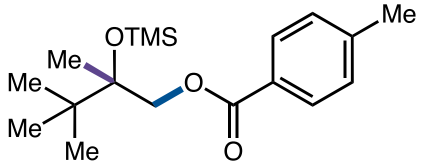

(±)-2,3,3-trimethyl-2-((trimethylsilyl)oxy)butyl 4-methylbenzoate (32): The title compound was prepared according to General Procedure B with Ni(acac)$_2$ (5 mol%, 25 μmol, 6.4 mg), KTp* (5 mol%, 25 μmol, 8.4 mg), (Ir[dF(CF$_3$)ppy]$_2$(dtbbpy))(PF$_6$) (2 mol%, 10 μmol, 11.2 mg), (1,3-dioxoisoindolin-2-yl)acetate (2 equiv, 1 mmol, 205.2 mg), 4-methylbenzoic acid (1 equiv, 0.5 mmol, 68.1 mg), ((3,3-dimethylbut-1-en-2-yl)oxy)trimethylsilane (2 equiv, 1 mmol, 172.3 mg, 216.0 μL), *tert*-butylimino-tri(pyrrolidino)phosphorane (0.2 equiv, 0.1 mmol, 31.2 mg, 30.6 μL), and 10 mL *t*-amyl alcohol. The crude reaction mixture was concentrated, dissolved in DMSO/MeCN, and purified by automated reverse-phase chromatography (25 g C18 column, 40-100% gradient of 0.1% ammonium hydroxide in water/0.1% ammonium hydroxide in

acetonitrile). Fractions were directly concentrated and the product dissolved in acetonitrile and filtered through a cotton plug into a 40 mL vial. The solvent was removed and the product dried on high-vac to yield the title compound as a dark yellow oil (112.8 mg, 0.350 mmol, 70% yield).

^{1}H NMR (500 MHz, CDCl$_3$) δ 7.96 (d, J = 8.2 Hz, 2H), 7.25 (d, J = 8.0 Hz, 2H), 4.37 (d, J = 11.3 Hz, 1H), 4.28 (d, J = 11.3 Hz, 1H), 2.42 (s, 3H), 1.32 (s, 3H), 0.99 (s, 9H), 0.11 (s, 9H).

^{13}C NMR (126 MHz, CDCl$_3$) δ 166.93, 143.72, 129.81, 129.25, 127.80, 79.16, 69.90, 38.30, 25.85, 21.80, 20.08, 2.61.

IR (film) v_{max} 2957, 2876, 1721, 1273, 1178, 1105, 753 cm^{-1}.

HRMS (ESI-TOF) *m/z* calculated for $C_{15}H_{21}O_2^+$ ([M-OTMS]$^+$) 233.1536, found 233.1534.

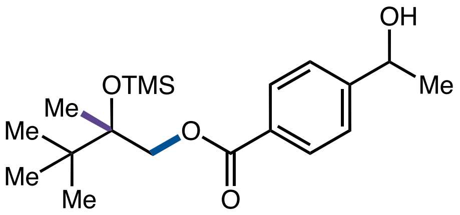

(±)-2,3,3-trimethyl-2-((trimethylsilyl)oxy)butyl 4-(1-hydroxyethyl)benzoate (33): The title compound was prepared according to General Procedure B with Ni(acac)$_2$ (5 mol%, 25 µmol, 6.4 mg), KTp* (5 mol%, 25 µmol, 8.4 mg), (Ir[dF(CF$_3$)ppy]$_2$(dtbbpy))(PF$_6$) (2 mol%, 10 µmol, 11.2 mg), (1,3-dioxoisoindolin-2-yl)acetate (2 equiv, 1 mmol, 205.2 mg), 4-(1-hydroxyethyl)benzoic acid (1 equiv, 0.5 mmol, 83.1 mg), ((3,3-dimethylbut-1-en-2-yl)oxy)trimethylsilane (2 equiv, 1 mmol, 172.3 mg, 216.0 µL), *tert*-butylimino-tri(pyrrolidino)phosphorane (0.2 equiv, 0.1 mmol, 31.2 mg, 30.6 µL), and 10 mL *t*-amyl alcohol. The crude reaction mixture was concentrated, dissolved in DMSO/MeCN, and purified by automated reverse-phase chromatography (25 g C18 column, 20-75% gradient of 0.1% ammonium hydroxide in water/0.1% ammonium hydroxide in acetonitrile). Fractions were directly concentrated and the product dissolved in acetonitrile and

filtered through a cotton plug into a 40 mL vial. The solvent was removed and the product dried on high-vac to yield the title compound as a yellow oil (98.8 mg, 0.280 mmol, 56% yield, single diastereomer, unassigned). Note: The crude reaction mixture exhibited >20:1 d.r., but only the major diastereomer was isolated and characterized.

^{1}H NMR (500 MHz, $CDCl_3$) δ 8.08 – 8.01 (m, 2H), 7.49 – 7.43 (m, 2H), 4.97 (q, *J* = 6.5 Hz, 1H), 4.39 (dd, *J* = 11.3, 1.7 Hz, 1H), 4.28 (dd, *J* = 11.3, 1.1 Hz, 1H), 1.89 (s, 1H), 1.52 (d, *J* = 6.5 Hz, 3H), 1.32 (s, 3H), 0.99 (s, 9H), 0.11 (s, 9H).

^{13}C NMR (126 MHz, $CDCl_3$) δ 166.66, 151.02, 130.07, 129.61, 125.48, 79.13, 70.16, 70.07, 38.31, 25.85, 25.45, 20.08, 2.62.

IR (film) v_{max} 3418, 2958, 2876, 1720, 1272, 1177, 1089, 752 cm^{-1}.

HRMS (ESI-TOF) *m/z* calculated for $C_{16}H_{23}O_3^+$ ([M-OTMS]$^+$) 263.1642, found 263.1643.

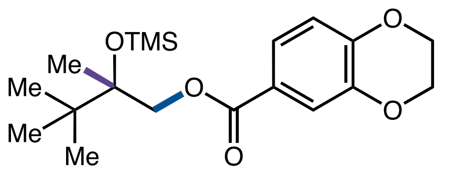

(±)-2,3,3-trimethyl-2-((trimethylsilyl)oxy)butyl 2,3-dihydrobenzo[*b*][1,4]dioxine-6-carboxylate (34): The title compound was prepared according to General Procedure B with $Ni(acac)_2$ (5 mol%, 25 μmol, 6.4 mg), KTp* (5 mol%, 25 μmol, 8.4 mg), (Ir[dF(CF_3)ppy]$_2$(dtbbpy))(PF_6) (2 mol%, 10 μmol, 11.2 mg), (1,3-dioxoisoindolin-2-yl)acetate (2 equiv, 1 mmol, 205.2 mg), 2,3-dihydrobenzo[*b*][1,4]dioxine-6-carboxylic acid (1 equiv, 0.5 mmol, 90.1 mg), ((3,3-dimethylbut-1-en-2-yl)oxy)trimethylsilane (2 equiv, 1 mmol, 172.3 mg, 216.0 μL), *tert*-butylimino-tri(pyrrolidino)phosphorane (0.2 equiv, 0.1 mmol, 31.2 mg, 30.6 μL), and 10 mL *t*-amyl alcohol. The crude reaction mixture was concentrated, dissolved in DMSO/MeCN, and purified by automated reverse-phase chromatography (25 g C18 column, 40-100% gradient of 0.1%

ammonium hydroxide in water/0.1% ammonium hydroxide in acetonitrile). Additional purification via automated flash chromatography (25 g high performance silica column, 0-20% ethyl acetate/hexanes). Fractions were directly concentrated and the product dissolved in acetonitrile and filtered through a cotton plug into a 40 mL vial. The solvent was removed and the product dried on high-vac to yield the title compound as a clear oil (93.5 mg, 0.255 mmol, 51% yield).

^{1}H NMR (500 MHz, $CDCl_3$) δ 7.62 – 7.55 (m, 2H), 6.93 – 6.86 (m, 1H), 4.40 – 4.18 (m, 6H), 1.30 (s, 3H), 0.98 (s, 9H), 0.11 (s, 9H).

^{13}C NMR (126 MHz, $CDCl_3$) δ 166.32, 147.91, 143.31, 123.80, 123.64, 119.20, 117.28, 79.13, 69.90, 64.76, 64.25, 38.29, 25.85, 20.09, 2.62.

IR (film) v_{max} 2957, 2877, 1715, 1290, 1191, 1066, 764 cm^{-1}.

HRMS (ESI-TOF) *m/z* calculated for $C_{16}H_{21}O_4^+$ ([M-OTMS]$^+$) 277.1434, found 277.1436.

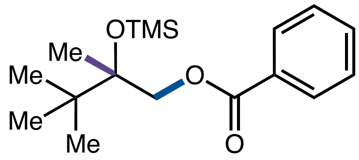

(±)-2,3,3-trimethyl-2-((trimethylsilyl)oxy)butyl benzoate (35): The title compound was prepared according to a modified General Procedure B with Ni(acac)$_2$ (5 mol%, 25 µmol, 6.4 mg), KTp* (5 mol%, 25 µmol, 8.4 mg), (Ir[dF(CF$_3$)ppy]$_2$(dtbbpy))(PF$_6$) (2 mol%, 10 µmol, 11.2 mg), (1,3-dioxoisoindolin-2-yl)acetate (2 equiv, 1 mmol, 205.2 mg), benzoic acid (1 equiv, 0.5 mmol, 61.1 mg), ((3,3-dimethylbut-1-en-2-yl)oxy)trimethylsilane (3 equiv, 1.5 mmol, 258.5 mg, 324.0 µL), *tert*-butylimino-tri(pyrrolidino)phosphorane (0.2 equiv, 0.1 mmol, 31.2 mg, 30.6 µL), and 10 mL *t*-amyl alcohol. The crude reaction mixture was concentrated, dissolved in DMSO/MeCN, and purified by automated reverse-phase chromatography (25 g C18 column, 20-90% gradient of 0.1%

ammonium hydroxide in water/0.1% ammonium hydroxide in acetonitrile). Fractions were directly concentrated and the product dissolved in acetonitrile and filtered through a cotton plug into a 40 mL vial. The solvent was removed and the product dried on high-vac to yield the title compound as a yellow oil (91.2 mg, 0.296 mmol, 59% yield).

^{1}H NMR (500 MHz, $CDCl_3$) δ 8.10 – 8.04 (m, 2H), 7.60 – 7.53 (m, 1H), 7.49 – 7.42 (m, 2H), 4.39 (d, *J* = 11.3 Hz, 1H), 4.30 (d, *J* = 11.3 Hz, 1H), 1.33 (s, 3H), 1.00 (s, 9H), 0.11 (s, 9H).

^{13}C NMR (126 MHz, $CDCl_3$) δ 166.86, 133.06, 130.56, 129.78, 128.54, 79.15, 70.10, 38.32, 25.86, 20.09, 2.61.

IR (film) v_{max} 2958, 2876, 1723, 1273, 1111, 1046, 753 cm^{-1}.

HRMS (ESI-TOF) *m/z* calculated for $C_{14}H_{19}O_2^+$ ([M-OTMS]$^+$) 219.1380, found 219.1380.

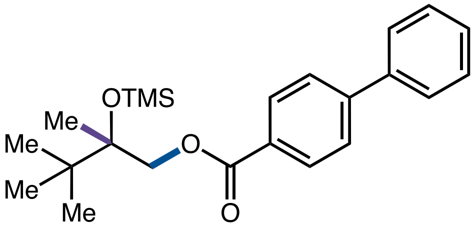

(±)-2,3,3-trimethyl-2-((trimethylsilyl)oxy)butyl [1,1'-biphenyl]-4-carboxylate (36): The title compound was prepared according to General Procedure B with Ni(acac)$_2$ (5 mol%, 25 µmol, 6.4 mg), KTp* (5 mol%, 25 µmol, 8.4 mg), (Ir[dF(CF_3)ppy]$_2$(dtbbpy))(PF_6) (2 mol%, 10 µmol, 11.2 mg), (1,3-dioxoisoindolin-2-yl)acetate (2 equiv, 1 mmol, 205.2 mg), [1,1'-biphenyl]-4-carboxylic acid (1 equiv, 0.5 mmol, 99.1 mg), ((3,3-dimethylbut-1-en-2-yl)oxy)trimethylsilane (2 equiv, 1 mmol, 172.3 mg, 216.0 µL), *tert*-butylimino-tri(pyrrolidino)phosphorane (0.2 equiv, 0.1 mmol, 31.2 mg, 30.6 µL), and 10 mL *t*-amyl alcohol. The crude reaction mixture was concentrated, dissolved in DMSO/MeCN, and purified by automated reverse-phase chromatography (25 g C18 column, 40-100% gradient of 0.1% ammonium hydroxide in water/0.1% ammonium hydroxide in

acetonitrile). Fractions were directly concentrated and the product dissolved in acetonitrile and filtered through a cotton plug into a 40 mL vial. The solvent was removed and the product dried on high-vac to yield the title compound as a dark yellow oil with residual BTTP (141.9 mg, 96.8% pure; 137.3 mg adjusted mass, 0.357 mmol, 71% yield).

^{1}H NMR (500 MHz, $CDCl_3$) δ 8.17 – 8.10 (m, 2H), 7.71 – 7.65 (m, 2H), 7.66 – 7.61 (m, 2H), 7.51 – 7.44 (m, 2H), 7.43 – 7.37 (m, 1H), 4.42 (d, J = 11.3 Hz, 1H), 4.32 (d, J = 11.3 Hz, 1H), 1.35 (s, 3H), 1.01 (s, 9H), 0.13 (s, 9H).

^{13}C NMR (126 MHz, $CDCl_3$) δ 166.74, 145.80, 140.14, 130.30, 129.25, 129.07, 128.29, 127.42, 127.22, 79.16, 70.11, 38.33, 25.87, 20.11, 2.64.

IR (film) v_{max} 2957, 2875, 1714, 1275, 1099, 1039, 746 cm^{-1}.

HRMS (ESI-TOF) *m/z* calculated for $C_{20}H_{23}O_2^+$ ([M-OTMS]$^+$) 295.1693, found 295.1695.

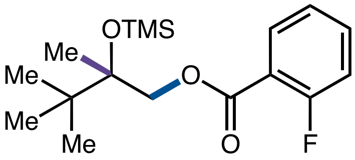

(±)-2,3,3-trimethyl-2-((trimethylsilyl)oxy)butyl 2-fluorobenzoate (37): The title compound was prepared according to a modified General Procedure B with Ni(acac)$_2$ (5 mol%, 25 µmol, 6.4 mg), KTp* (5 mol%, 25 µmol, 8.4 mg), (Ir[dF(CF_3)ppy]$_2$(dtbbpy))(PF_6) (2 mol%, 10 µmol, 11.2 mg), (1,3-dioxoisoindolin-2-yl)acetate (2 equiv, 1 mmol, 205.2 mg), 2-fluorobenzoic acid (1 equiv, 0.5 mmol, 70.1 mg), ((3,3-dimethylbut-1-en-2-yl)oxy)trimethylsilane (3 equiv, 1.5 mmol, 258.5 mg, 324.0 µL), *tert*-butylimino-tri(pyrrolidino)phosphorane (0.2 equiv, 0.1 mmol, 31.2 mg, 30.6 µL), and 10 mL *t*-amyl alcohol. The crude reaction mixture was concentrated, dissolved in DMSO/MeCN, and purified by automated reverse-phase chromatography (25 g C18 column, 20-100% gradient of 0.1% ammonium hydroxide in water/0.1% ammonium hydroxide in

acetonitrile). Fractions were directly concentrated and the product dissolved in acetonitrile and filtered through a cotton plug into a 40 mL vial. The solvent was removed and the product dried on high-vac to yield the title compound as a dark yellow oil (108.3 mg, 0.332 mmol, 66% yield).

^{1}H NMR (500 MHz, CDCl$_3$) δ 7.97 – 7.92 (m, 1H), 7.55 – 7.49 (m, 1H), 7.25 – 7.19 (m, 1H), 7.18 – 7.11 (m, 1H), 4.38 (d, J = 11.3 Hz, 1H), 4.31 (d, J = 11.3 Hz, 1H), 1.33 (s, 3H), 0.98 (s, 9H), 0.08 (s, 9H).

^{13}C NMR (126 MHz, CDCl$_3$) δ 164.75 (d, J = 3.4 Hz), 161.97 (d, J = 259.7 Hz), 134.52 (d, J = 8.9 Hz), 132.16, 124.09 (d, J = 3.9 Hz), 119.22 (d, J = 10.1 Hz), 117.14 (d, J = 22.4 Hz), 79.04, 70.55, 38.25, 25.79, 19.89, 2.47.

^{19}F NMR (376 MHz, CDCl$_3$) δ -108.94 (ddd, J = 11.6, 7.3, 4.9 Hz).

IR (film) v_{max} 2958, 2877, 1718, 1250, 1155, 1036, 755 cm^{-1}.

HRMS (ESI-TOF) *m/z* calculated for $C_{14}H_{18}FO_2^+$ ([M-OTMS]$^+$) 237.1285, found 237.1284.

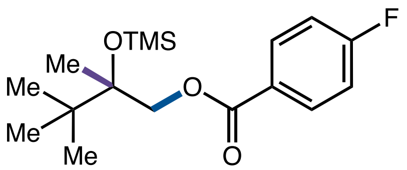

(±)-2,3,3-trimethyl-2-((trimethylsilyl)oxy)butyl 4-fluorobenzoate (38): The title compound was prepared according to a modified General Procedure B with Ni(acac)$_2$ (5 mol%, 25 µmol, 6.4 mg), KTp* (5 mol%, 25 µmol, 8.4 mg), (Ir[dF(CF$_3$)ppy]$_2$(dtbbpy))(PF$_6$) (2 mol%, 10 µmol, 11.2 mg), (1,3-dioxoisoindolin-2-yl)acetate (2 equiv, 1 mmol, 205.2 mg), 4-fluorobenzoic acid (1 equiv, 0.5 mmol, 70.1 mg), ((3,3-dimethylbut-1-en-2-yl)oxy)trimethylsilane (3 equiv, 1.5 mmol, 258.5 mg, 324.0 µL), *tert*-butylimino-tri(pyrrolidino)phosphorane (0.2 equiv, 0.1 mmol, 31.2 mg, 30.6 µL), and 10 mL *t*-amyl alcohol. The crude reaction mixture was concentrated, dissolved in DMSO/MeCN, and purified by automated reverse-phase chromatography (25 g C18 column, 40-

100% gradient of 0.1% ammonium hydroxide in water/0.1% ammonium hydroxide in acetonitrile). Fractions were directly concentrated and the product dissolved in acetonitrile and filtered through a cotton plug into a 40 mL vial. The solvent was removed and the product dried on high-vac to yield the title compound as a dark yellow oil (87.9 mg, 0.269 mmol, 54% yield).

^{1}H NMR (500 MHz, $CDCl_3$) δ 8.11 – 8.04 (m, 2H), 7.17 – 7.08 (m, 2H), 4.37 (d, *J* = 11.3 Hz, 1H), 4.29 (d, *J* = 11.3 Hz, 1H), 1.31 (s, 3H), 0.99 (s, 9H), 0.10 (s, 9H).

^{13}C NMR (126 MHz, $CDCl_3$) δ 165.90 (d, *J* = 253.9 Hz), 165.87, 132.27 (d, *J* = 9.3 Hz), 126.79 (d, *J* = 2.9 Hz), 115.71 (d, *J* = 22.0 Hz), 79.12, 70.22, 38.31, 25.85, 20.09, 2.62.

^{19}F NMR (376 MHz, $CDCl_3$) δ -105.75 (tt, *J* = 8.5, 5.4 Hz).

IR (film) v_{max} 2958, 2877, 1725, 1272, 1152, 1045, 753 cm^{-1}.

HRMS (ESI-TOF) *m/z* calculated for $C_{14}H_{18}FO_2^+$ ([M-OTMS]$^+$) 237.1285, found 237.1286.

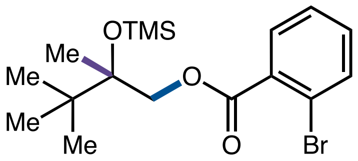

(±)-2,3,3-trimethyl-2-((trimethylsilyl)oxy)butyl 2-bromobenzoate (39): The title compound was prepared according to General Procedure B with Ni(acac)$_2$ (5 mol%, 25 µmol, 6.4 mg), KTp* (5 mol%, 25 µmol, 8.4 mg), (Ir[dF(CF_3)ppy]$_2$(dtbbpy))(PF_6) (2 mol%, 10 µmol, 11.2 mg), (1,3-dioxoisoindolin-2-yl)acetate (2 equiv, 1 mmol, 205.2 mg), 2-bromobenzoic acid (1 equiv, 0.5 mmol, 100.5 mg), ((3,3-dimethylbut-1-en-2-yl)oxy)trimethylsilane (2 equiv, 1 mmol, 172.3 mg, 216.0 µL), *tert*-butylimino-tri(pyrrolidino)phosphorane (0.2 equiv, 0.1 mmol, 31.2 mg, 30.6 µL), and 10 mL *t*-amyl alcohol. The crude reaction mixture was concentrated, dissolved in DMSO/MeCN, and purified by automated reverse-phase chromatography (25 g C18 column, 40-100% gradient of 0.1% ammonium hydroxide in water/0.1% ammonium hydroxide in

acetonitrile). Fractions were directly concentrated and the product dissolved in acetonitrile and filtered through a cotton plug into a 40 mL vial. The solvent was removed and the product dried on high-vac to yield the title compound as a dark yellow oil (149.9 mg, 0.387 mmol, 77% yield).

^{1}H NMR (500 MHz, CDCl$_3$) δ 7.75 (dd, *J* = 7.5, 1.9 Hz, 1H), 7.67 (dd, *J* = 7.8, 1.4 Hz, 1H), 7.41 – 7.29 (m, 2H), 4.42 (d, *J* = 11.2 Hz, 1H), 4.28 (d, *J* = 11.2 Hz, 1H), 1.31 (s, 3H), 0.98 (s, 9H), 0.05 (s, 9H).

^{13}C NMR (126 MHz, CDCl$_3$) δ 166.62, 134.39, 133.02, 132.50, 130.94, 127.25, 121.57, 78.97, 70.74, 38.24, 25.80, 20.01, 2.52.

IR (film) v_{max} 2957, 2876, 1735, 1292, 1250, 1132, 1043, 744 cm^{-1}.

HRMS (ESI-TOF) *m/z* calculated for $C_{14}H_{18}BrO_2^+$ ([M-OTMS]$^+$) 297.0485, found 297.0487.

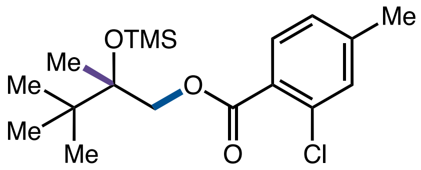

(±)-2,3,3-trimethyl-2-((trimethylsilyl)oxy)butyl 2-chloro-4-methylbenzoate (40): The title compound was prepared according to General Procedure B with Ni(acac)$_2$ (5 mol%, 25 µmol, 6.4 mg), KTp* (5 mol%, 25 µmol, 8.4 mg), (Ir[dF(CF$_3$)ppy]$_2$(dtbbpy))(PF$_6$) (2 mol%, 10 µmol, 11.2 mg), (1,3-dioxoisoindolin-2-yl)acetate (2 equiv, 1 mmol, 205.2 mg), 2-chloro-4-methylbenzoic acid (1 equiv, 0.5 mmol, 85.3 mg), ((3,3-dimethylbut-1-en-2-yl)oxy)trimethylsilane (2 equiv, 1 mmol, 172.3 mg, 216.0 µL), *tert*-butylimino-tri(pyrrolidino)phosphorane (0.2 equiv, 0.1 mmol, 31.2 mg, 30.6 µL), and 10 mL *t*-amyl alcohol. The crude reaction mixture was concentrated, dissolved in DMSO/MeCN, and purified by automated reverse-phase chromatography (25 g C18 column, 40-100% gradient of 0.1% ammonium hydroxide in water/0.1% ammonium hydroxide in acetonitrile). Fractions were directly concentrated and the product dissolved in acetonitrile and

filtered through a cotton plug into a 40 mL vial. The solvent was removed and the product dried on high-vac to yield the title compound as a yellow oil (164.0 mg, 0.459 mmol, 92% yield).

^{1}H NMR (500 MHz, CDCl$_3$) δ 7.73 (d, *J* = 7.9 Hz, 1H), 7.30 – 7.27 (m, 1H), 7.14 – 7.09 (m, 1H), 4.38 (d, *J* = 11.2 Hz, 1H), 4.27 (d, *J* = 11.3 Hz, 1H), 2.37 (s, 3H), 1.31 (s, 3H), 0.97 (s, 9H), 0.05 (s, 9H).

^{13}C NMR (126 MHz, CDCl$_3$) δ 165.97, 143.58, 133.69, 131.74, 131.29, 127.58, 127.50, 78.99, 70.48, 38.23, 25.80, 21.32, 20.02, 2.52.

IR (film) v_{max} 2957, 2876, 1734, 1250, 1126, 1047, 769 cm^{-1}.

HRMS (ESI-TOF) *m/z* calculated for $C_{15}H_{20}ClO_2^+$ ([M-OTMS]$^+$) 267.1146, found 267.1143.

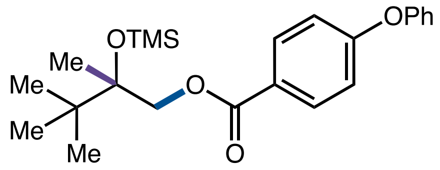

(±)-2,3,3-trimethyl-2-((trimethylsilyl)oxy)butyl 4-phenoxybenzoate (41): The title compound was prepared according to General Procedure B with Ni(acac)$_2$ (5 mol%, 25 µmol, 6.4 mg), KTp* (5 mol%, 25 µmol, 8.4 mg), (Ir[dF(CF$_3$)ppy]$_2$(dtbbpy))(PF$_6$) (2 mol%, 10 µmol, 11.2 mg), (1,3-dioxoisoindolin-2-yl)acetate (2 equiv, 1 mmol, 205.2 mg), 4-phenoxybenzoic acid (1 equiv, 0.5 mmol, 107.1 mg), ((3,3-dimethylbut-1-en-2-yl)oxy)trimethylsilane (2 equiv, 1 mmol, 172.3 mg, 216.0 µL), *tert*-butylimino-tri(pyrrolidino)phosphorane (0.2 equiv, 0.1 mmol, 31.2 mg, 30.6 µL), and 10 mL *t*-amyl alcohol. The crude reaction mixture was concentrated, dissolved in DMSO/MeCN, and purified by automated reverse-phase chromatography (25 g C18 column, 20-100% gradient of 0.1% ammonium hydroxide in water/0.1% ammonium hydroxide in acetonitrile). Fractions were directly concentrated and the product dissolved in acetonitrile and

filtered through a cotton plug into a 40 mL vial. The solvent was removed and the product dried on high-vac to yield the title compound as a yellow oil (163.9 mg, 0.409 mmol, 82% yield).

^{1}H NMR (500 MHz, CDCl$_3$) δ 8.07 – 8.00 (m, 2H), 7.40 (t, *J* = 7.9 Hz, 2H), 7.20 (t, *J* = 7.4 Hz, 1H), 7.08 (d, *J* = 8.0 Hz, 2H), 7.03 – 6.97 (m, 2H), 4.38 (d, *J* = 11.3 Hz, 1H), 4.27 (d, *J* = 11.4 Hz, 1H), 1.31 (s, 3H), 0.99 (s, 9H), 0.11 (s, 9H).

^{13}C NMR (126 MHz, CDCl$_3$) δ 166.32, 162.03, 155.71, 131.85, 130.17, 124.77, 124.69, 120.33, 117.37, 79.14, 69.95, 38.30, 25.85, 20.09, 2.62.

IR (film) v_{max} 2957, 2876, 1718, 1489, 1242, 1161, 1043, 752 cm^{-1}.

HRMS (ESI-TOF) *m/z* calculated for $C_{20}H_{23}O_3^+$ ([M-OTMS]$^+$) 311.1642, found 311.1644.

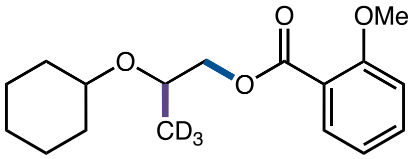

(±)-2-(cyclohexyloxy)propyl-3,3,3-*d*$_3$ 2-methoxybenzoate (42): The title compound was prepared according to General Procedure B with Ni(acac)$_2$ (5 mol%, 25 µmol, 6.4 mg), KTp* (5 mol%, 25 µmol, 8.4 mg), (Ir[dF(CF$_3$)ppy]$_2$(dtbbpy))(PF$_6$) (2 mol%, 10 µmol, 11.2 mg), 1,3-dioxoisoindolin-2-yl acetate-*d*$_3$ (2 equiv, 1 mmol, 208.2 mg), 2-methoxybenzoic acid (1 equiv, 0.5 mmol, 76.1 mg), (vinyloxy)cyclohexane (2 equiv, 1 mmol, 126.2 mg, 141.6 µL), *tert*-butylimino-tri(pyrrolidino)phosphorane (0.2 equiv, 0.1 mmol, 31.2 mg, 30.6 µL), and 10 mL *t*-amyl alcohol. The crude reaction mixture was concentrated, dissolved in DMSO/MeCN, and purified by automated reverse-phase chromatography (25 g C18 column, 20-55% gradient of 0.1% ammonium hydroxide in water/0.1% ammonium hydroxide in acetonitrile). Fractions were directly concentrated and the product dissolved in DCM and filtered through a cotton plug into a 40 mL

vial. The solvent was removed and the product dried on high-vac to yield the title compound as a dark oil (114.2 mg, 0.387 mmol, 77% yield).

^{1}H NMR (500 MHz, CDCl$_3$) δ 7.82 (dd, *J* = 7.9, 1.8 Hz, 1H), 7.47 (ddd, *J* = 8.4, 7.4, 1.8 Hz, 1H), 7.01 – 6.94 (m, 2H), 4.25 (dd, *J* = 11.2, 6.3 Hz, 1H), 4.18 (dd, *J* = 11.2, 5.1 Hz, 1H), 3.90 (s, 3H), 3.87 (t, *J* = 5.7 Hz, 1H), 3.44 – 3.35 (m, 1H), 1.93 – 1.85 (m, 2H), 1.77 – 1.68 (m, 2H), 1.56 – 1.47 (m, 1H), 1.33 – 1.15 (m, 5H).

^{13}C NMR (126 MHz, CDCl$_3$) δ 166.10, 159.46, 133.66, 131.82, 120.20, 120.18, 112.12, 76.57, 70.42, 68.28, 56.05, 33.22, 33.08, 25.88, 24.46, 24.44, 17.70 (m).

IR (film) v_{max} 2934, 2856, 1725, 1301, 1257, 1078, 753 cm^{-1}.

HRMS (ESI-TOF) *m/z* calculated for $C_{17}H_{22}D_3O_4^+$ ([M+H]$^+$) 296.1936, found 296.1933.

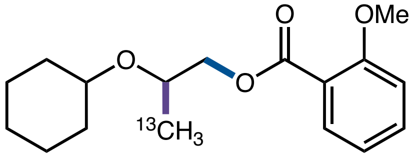

(±)-2-(cyclohexyloxy)propyl-3-^{13}*C* 2-methoxybenzoate (43): The title compound was prepared according to General Procedure B with Ni(acac)$_2$ (5 mol%, 25 µmol, 6.4 mg), KTp* (5 mol%, 25 µmol, 8.4 mg), (Ir[dF(CF$_3$)ppy]$_2$(dtbbpy))(PF$_6$) (2 mol%, 10 µmol, 11.2 mg), 1,3-dioxoisoindolin-2-yl acetate-2-^{13}*C* (2 equiv, 1 mmol, 206.2 mg), 2-methoxybenzoic acid (1 equiv, 0.5 mmol, 76.1 mg), (vinyloxy)cyclohexane (2 equiv, 1 mmol, 126.2 mg, 141.6 µL), *tert*-butylimino-tri(pyrrolidino)phosphorane (0.2 equiv, 0.1 mmol, 31.2 mg, 30.6 µL), and 10 mL *t*-amyl alcohol. The crude reaction mixture was concentrated, dissolved in DMSO/MeCN, and purified by automated reverse-phase chromatography (25 g C18 column, 20-55% gradient of 0.1% ammonium hydroxide in water/0.1% ammonium hydroxide in acetonitrile). Fractions were directly concentrated and the product dissolved in acetonitrile and filtered through a cotton plug into a 40

mL vial. The solvent was removed and the product dried on high-vac to yield the title compound as a dark oil (108.0 mg, 0.368 mmol, 74% yield).

^{1}H NMR (500 MHz, $CDCl_3$) δ 7.82 (dd, *J* = 7.9, 1.9 Hz, 1H), 7.47 (ddd, *J* = 8.3, 7.4, 1.8 Hz, 1H), 7.00 – 6.95 (m, 2H), 4.25 (ddd, *J* = 11.2, 6.3, 3.2 Hz, 1H), 4.18 (ddd, *J* = 11.2, 5.1, 2.9 Hz, 1H), 3.90 (s, 4H), 3.44 – 3.35 (m, 1H), 1.93 – 1.85 (m, 2H), 1.77 – 1.68 (m, 2H), 1.56 – 1.48 (m, 1H), 1.33 – 1.14 (m, 5H), 1.25 (dd, *J* = 126.4, 6.4 Hz, 3H).

^{13}C NMR (126 MHz, $CDCl_3$) δ 166.09, 159.46, 133.67, 131.83, 120.20, 120.15, 112.11, 76.58, 70.55 (d, *J* = 39.1 Hz), 68.30, 56.05, 33.22, 33.07, 25.87, 24.45, 18.52.

IR (film) v_{max} 2933, 2856, 1726, 1297, 1259, 1078, 751 cm^{-1}.

HRMS (ESI-TOF) *m/z* calculated for $C_{16}{}^{13}CH_{25}O_4^+$ ($[M+H]^+$) 294.1781, found 294.1783.

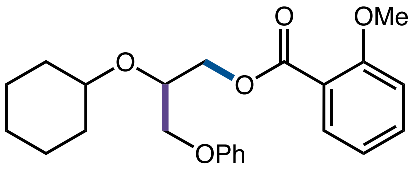

(±)-2-(cyclohexyloxy)-3-phenoxypropyl 2-methoxybenzoate (44): The title compound was prepared according to General Procedure C with $Ni(acac)_2$ (15 mol%, 75 µmol, 19.3 mg), $(Ir[dF(CF_3)ppy]_2(dtbbpy))(PF_6)$ (2 mol%, 10 µmol, 11.2 mg), 1,3-dioxoisoindolin-2-yl 2-phenoxyacetate (3 equiv, 1.5 mmol, 445.9 mg), 2-methoxybenzoic acid (1 equiv, 0.5 mmol, 76.1 mg), (vinyloxy)cyclohexane (3 equiv, 1.5 mmol, 189.3 mg, 212.5 µL), *tert*-butylimino-tri(pyrrolidino)phosphorane (0.2 equiv, 0.1 mmol, 31.2 mg, 30.6 µL), and 10 mL *t*-amyl alcohol. The crude reaction mixture was concentrated, dissolved in DMSO/MeCN, and purified by automated reverse-phase chromatography (25 g C18 column, 20-55% gradient of 0.1% ammonium hydroxide in water/0.1% ammonium hydroxide in acetonitrile). Fractions were directly concentrated and the product dissolved in acetonitrile and filtered through a cotton plug into a 40

mL vial. The solvent was removed and the product dried on high-vac to yield the title compound as a yellow oil (124.8 mg, 0.325 mmol, 65% yield).

^{1}H NMR (500 MHz, $CDCl_3$) δ 7.80 (dd, *J* = 7.9, 1.8 Hz, 1H), 7.47 (ddd, *J* = 8.4, 7.4, 1.8 Hz, 1H), 7.31 – 7.26 (m, 2H), 6.99 – 6.91 (m, 5H), 4.49 (dd, *J* = 11.4, 4.8 Hz, 1H), 4.42 (dd, *J* = 11.5, 5.2 Hz, 1H), 4.16 – 4.04 (m, 3H), 3.86 (s, 3H), 3.57 – 3.49 (m, 1H), 1.97 – 1.89 (m, 2H), 1.78 – 1.70 (m, 2H), 1.56 – 1.49 (m, 1H), 1.40 – 1.31 (m, 2H), 1.29 – 1.17 (m, 3H).

^{13}C NMR (126 MHz, $CDCl_3$) δ 166.11, 159.45, 158.92, 133.78, 131.89, 129.57, 121.03, 120.24, 120.00, 114.76, 112.11, 77.94, 73.42, 68.40, 64.57, 56.02, 33.02, 32.84, 25.82, 24.34, 24.30.

IR (film) v_{max} 2933, 2856, 1727, 1493, 1257, 1078, 752 cm^{-1}.

HRMS (ESI-TOF) *m/z* calculated for $C_{23}H_{29}O_5^+$ ($[M+H]^+$) 385.2010, found 385.2010.

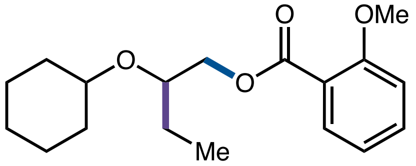

(±)-2-(cyclohexyloxy)butyl 2-methoxybenzoate (45): The title compound was prepared according to General Procedure C with Ni(acac)$_2$ (15 mol%, 75 µmol, 19.3 mg), (Ir[dF(CF$_3$)ppy]$_2$(dtbbpy))(PF$_6$) (2 mol%, 10 µmol, 11.2 mg), 1,3-dioxoisoindolin-2-yl propionate (3 equiv, 1.5 mmol, 328.8 mg), 2-methoxybenzoic acid (1 equiv, 0.5 mmol, 76.1 mg), (vinyloxy)cyclohexane (3 equiv, 1.5 mmol, 189.3 mg, 212.5 µL), *tert*-butylimino-tri(pyrrolidino)phosphorane (0.2 equiv, 0.1 mmol, 31.2 mg, 30.6 µL), and 10 mL *t*-amyl alcohol. The crude reaction mixture was concentrated, dissolved in DMSO/MeCN, and purified by automated reverse-phase chromatography (25 g C18 column, 20-55% gradient of 0.1% ammonium hydroxide in water/0.1% ammonium hydroxide in acetonitrile). Fractions were directly concentrated and the product dissolved in acetonitrile and filtered through a cotton plug into a 40

mL vial. The solvent was removed and the product dried on high-vac to yield the title compound as a yellow oil (96.0 mg, 0.313 mmol, 63% yield).

^{1}H NMR (500 MHz, CDCl$_3$) δ 7.81 (dd, *J* = 7.9, 1.8 Hz, 1H), 7.46 (ddd, *J* = 8.4, 7.4, 1.8 Hz, 1H), 7.00 – 6.94 (m, 2H), 4.29 – 4.21 (m, 2H), 3.90 (s, 3H), 3.65 – 3.58 (m, 1H), 3.43 – 3.35 (m, 1H), 1.93 – 1.86 (m, 2H), 1.76 – 1.69 (m, 2H), 1.68 – 1.60 (m, 1H), 1.58 – 1.49 (m, 2H), 1.32 – 1.16 (m, 5H), 0.99 (t, *J* = 7.4 Hz, 3H).

^{13}C NMR (126 MHz, DMSO-*d*$_6$) δ 165.51, 158.27, 133.56, 130.56, 120.05, 120.03, 112.58, 75.57, 75.06, 66.22, 55.73, 32.72, 32.22, 25.30, 24.84, 23.60, 23.56, 9.71.

(^{13}C NMR taken in DMSO-*d*$_6$ due to peak overlap with CDCl$_3$)

IR (film) v_{max} 2933, 2857, 1724, 1301, 1258, 1079, 751 cm^{-1}.

HRMS (ESI-TOF) *m/z* calculated for $C_{18}H_{27}O_4^+$ ([M+H]$^+$) 307.1904, found 307.1900.

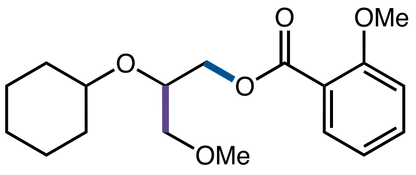

(±)-2-(cyclohexyloxy)-3-methoxypropyl 2-methoxybenzoate (46): The title compound was prepared according to General Procedure C with Ni(acac)$_2$ (15 mol%, 75 µmol, 19.3 mg), (Ir[dF(CF$_3$)ppy]$_2$(dtbbpy))(PF$_6$) (2 mol%, 10 µmol, 11.2 mg), 1,3-dioxoisoindolin-2-yl 2-methoxyacetate (3 equiv, 1.5 mmol, 352.8 mg), 2-methoxybenzoic acid (1 equiv, 0.5 mmol, 76.1 mg), (vinyloxy)cyclohexane (3 equiv, 1.5 mmol, 189.3 mg, 212.5 µL), *tert*-butylimino-tri(pyrrolidino)phosphorane (0.2 equiv, 0.1 mmol, 31.2 mg, 30.6 µL), and 10 mL *t*-amyl alcohol. The crude reaction mixture was concentrated, dissolved in DMSO/MeCN, and purified by automated reverse-phase chromatography (25 g C18 column, 20-50% gradient of 0.1% ammonium hydroxide in water/0.1% ammonium hydroxide in acetonitrile). Fractions were directly

concentrated and the product dissolved in acetonitrile and filtered through a cotton plug into a 40 mL vial. The solvent was removed and the product dried on high-vac to yield the title compound as a yellow oil (95.1 mg, 0.295 mmol, 59% yield).

^{1}H NMR (500 MHz, CDCl$_3$) δ 7.81 (dd, *J* = 7.9, 1.8 Hz, 1H), 7.47 (ddd, *J* = 8.4, 7.4, 1.8 Hz, 1H), 7.01 – 6.95 (m, 2H), 4.38 (dd, *J* = 11.4, 5.2 Hz, 1H), 4.30 (dd, *J* = 11.4, 5.9 Hz, 1H), 3.90 (s, 3H), 3.90 – 3.86 (m, 1H), 3.54 – 3.48 (m, 2H), 3.48 – 3.41 (m, 1H), 3.39 (s, 3H), 1.94 – 1.87 (m, 2H), 1.77 – 1.69 (m, 2H), 1.54 – 1.48 (m, 1H), 1.36 – 1.13 (m, 5H).

^{13}C NMR (126 MHz, CDCl$_3$) δ 166.06, 159.45, 133.71, 131.85, 120.21, 120.09, 112.11, 77.71, 73.90, 73.29, 64.80, 59.55, 56.04, 33.07, 32.92, 25.83, 24.44, 24.41.

IR (film) v_{max} 2933, 2856, 1728, 1298, 1257, 1077, 753 cm^{-1}.

HRMS (ESI-TOF) *m/z* calculated for $C_{18}H_{27}O_5^+$ ($[M+H]^+$) 323.1853, found 323.1851.

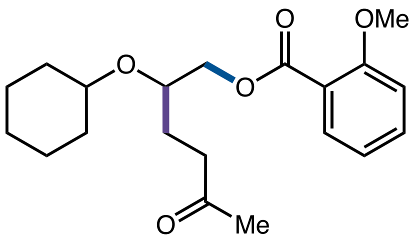

(±)-2-(cyclohexyloxy)-5-oxohexyl 2-methoxybenzoate (47): The title compound was prepared according to General Procedure C with Ni(acac)$_2$ (15 mol%, 75 µmol, 19.3 mg), (Ir[dF(CF$_3$)ppy]$_2$(dtbbpy))(PF$_6$) (2 mol%, 10 µmol, 11.2 mg), 1,3-dioxoisoindolin-2-yl 4-oxopentanoate (3 equiv, 1.5 mmol, 391.8 mg), 2-methoxybenzoic acid (1 equiv, 0.5 mmol, 76.1 mg), (vinyloxy)cyclohexane (3 equiv, 1.5 mmol, 189.3 mg, 212.5 µL), *tert*-butylimino-tri(pyrrolidino)phosphorane (0.2 equiv, 0.1 mmol, 31.2 mg, 30.6 µL), and 10 mL *t*-amyl alcohol. The crude reaction mixture was concentrated, dissolved in DMSO/MeCN, and purified by automated reverse-phase chromatography (25 g C18 column, 20-50% gradient of 0.1% ammonium

hydroxide in water/0.1% ammonium hydroxide in acetonitrile). Fractions were directly concentrated and the product dissolved in acetonitrile and filtered through a cotton plug into a 40 mL vial. The solvent was removed and the product dried on high-vac to yield the title compound as a yellow oil (104.7 mg, 0.300 mmol, 60% yield).

^{1}H NMR (500 MHz, $CDCl_3$) δ 7.80 (dd, *J* = 7.9, 1.8 Hz, 1H), 7.47 (ddd, *J* = 8.4, 7.4, 1.9 Hz, 1H), 7.01 – 6.94 (m, 2H), 4.29 (dd, *J* = 11.4, 5.3 Hz, 1H), 4.20 (dd, *J* = 11.3, 5.7 Hz, 1H), 3.90 (s, 3H), 3.76 – 3.68 (m, 1H), 3.43 – 3.34 (m, 1H), 2.65 – 2.52 (m, 2H), 2.15 (s, 3H), 1.99 – 1.92 (m, 1H), 1.90 – 1.84 (m, 2H), 1.80 – 1.68 (m, 3H), 1.55 – 1.49 (m, 1H), 1.30 – 1.15 (m, 5H).

^{13}C NMR (126 MHz, $CDCl_3$) δ 208.71, 166.09, 159.46, 133.78, 131.84, 120.22, 119.92, 112.09, 77.04, 73.45, 66.61, 56.03, 39.47, 33.48, 32.68, 30.11, 26.63, 25.79, 24.48, 24.41.

IR (film) v_{max} 2933, 2856, 1720, 1300, 1257, 1077, 752 cm^{-1}.

HRMS (ESI-TOF) *m/z* calculated for $C_{20}H_{28}NaO_5^+$ ($[M+Na]^+$) 371.1829, found 371.1825.

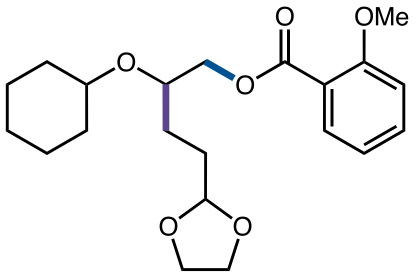

(±)-2-(cyclohexyloxy)-4-(1,3-dioxolan-2-yl)butyl 2-methoxybenzoate (48): The title compound was prepared according to General Procedure C with $Ni(acac)_2$ (15 mol%, 75 µmol, 19.3 mg), $(Ir[dF(CF_3)ppy]_2(dtbbpy))(PF_6)$ (2 mol%, 10 µmol, 11.2 mg), 1,3-dioxoisoindolin-2-yl 3-(1,3-dioxolan-2-yl)propanoate (3 equiv, 1.5 mmol, 436.9 mg), 2-methoxybenzoic acid (1 equiv, 0.5 mmol, 76.1 mg), (vinyloxy)cyclohexane (3 equiv, 1.5 mmol, 189.3 mg, 212.5 µL), *tert*-butylimino-tri(pyrrolidino)phosphorane (0.2 equiv, 0.1 mmol, 31.2 mg, 30.6 µL), and 10 mL *t*-amyl alcohol.

The crude reaction mixture was concentrated, dissolved in DMSO/MeCN, and purified by automated reverse-phase chromatography (25 g C18 column, 20-60% gradient of 0.1% ammonium hydroxide in water/0.1% ammonium hydroxide in acetonitrile). Additional purification via automated flash chromatography (25 g high performance silica column, 0-30% ethyl acetate/hexanes). Fractions were directly concentrated and the product dissolved in DCM and filtered through a cotton plug into a 40 mL vial. The solvent was removed and the product dried on high-vac to yield the title compound as a pale yellow oil (100.3 mg, 0.265 mmol, 53% yield).

^{1}H NMR (500 MHz, $CDCl_3$) δ 7.81 (dd, J = 7.9, 1.8 Hz, 1H), 7.47 (ddd, J = 8.9, 7.5, 1.8 Hz, 1H), 7.01 – 6.93 (m, 2H), 4.89 (t, J = 4.5 Hz, 1H), 4.29 (dd, J = 11.3, 5.6 Hz, 1H), 4.22 (dd, J = 11.3, 5.4 Hz, 1H), 3.98 – 3.94 (m, 2H), 3.90 (s, 3H), 3.87 – 3.83 (m, 2H), 3.76 – 3.70 (m, 1H), 3.43 – 3.37 (m, 1H), 1.92 – 1.87 (m, 2H), 1.80 – 1.61 (m, 6H), 1.53 – 1.48 (m, 1H), 1.30 – 1.16 (m, 5H).

^{13}C NMR (126 MHz, DMSO-d_6) δ 166.04, 158.75, 134.07, 131.07, 120.53, 113.08, 104.07, 76.04, 74.05, 66.78, 64.72, 64.68, 64.64, 56.19, 33.28, 32.59, 29.87, 26.94, 25.76, 24.07, 24.03.

(^{13}C NMR taken in DMSO-d_6 due to peak overlap with $CDCl_3$)

IR (film) v_{max} 2933, 2856, 1725, 1299, 1259, 1075, 751 cm^{-1}.

HRMS (ESI-TOF) *m/z* calculated for $C_{21}H_{31}O_6^+$ ($[M+H]^+$) 379.2115, found 379.2118.

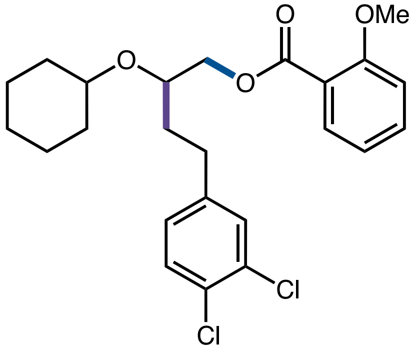

(±)-2-(cyclohexyloxy)-4-(3,4-dichlorophenyl)butyl 2-methoxybenzoate (49): The title compound was prepared according to General Procedure C with Ni(acac)$_2$ (15 mol%, 75 µmol, 19.3 mg), (Ir[dF(CF$_3$)ppy]$_2$(dtbbpy))(PF$_6$) (2 mol%, 10 µmol, 11.2 mg), 1,3-dioxoisoindolin-2-yl 3-(3,4-dichlorophenyl)propanoate (3 equiv, 1.5 mmol, 546.3 mg), 2-methoxybenzoic acid (1 equiv, 0.5 mmol, 76.1 mg), (vinyloxy)cyclohexane (3 equiv, 1.5 mmol, 189.3 mg, 212.5 µL), *tert*-butylimino-tri(pyrrolidino)phosphorane (0.2 equiv, 0.1 mmol, 31.2 mg, 30.6 µL), and 10 mL *t*-amyl alcohol. The reported 45% yield was obtained based on crude ^{1}H NMR assay with 64.9 mg of mesitylene as an internal standard (see below). The crude reaction mixture was concentrated, dissolved in DMSO/MeCN, and purified by automated reverse-phase chromatography (25 g C18 column, 20-75% gradient of 0.1% ammonium hydroxide in water/0.1% ammonium hydroxide in acetonitrile). Additional purification was attempted via automated flash chromatography (25 g high performance silica column, 0-30% ethyl acetate/hexanes) and Preparative HPLC (XBridge BEH C18 OBD column, 40-90% gradient of 0.1% ammonium hydroxide in water/0.1% ammonium hydroxide in acetonitrile), but the desired product was not fully resolved from impurities. Pure fractions were directly concentrated and the product dissolved in acetonitrile and filtered through a cotton plug into a 40 mL vial. The solvent was removed and the product dried on high-vac to yield the title compound as a pale yellow oil (54.6 mg, 0.121 mmol, 24% yield).

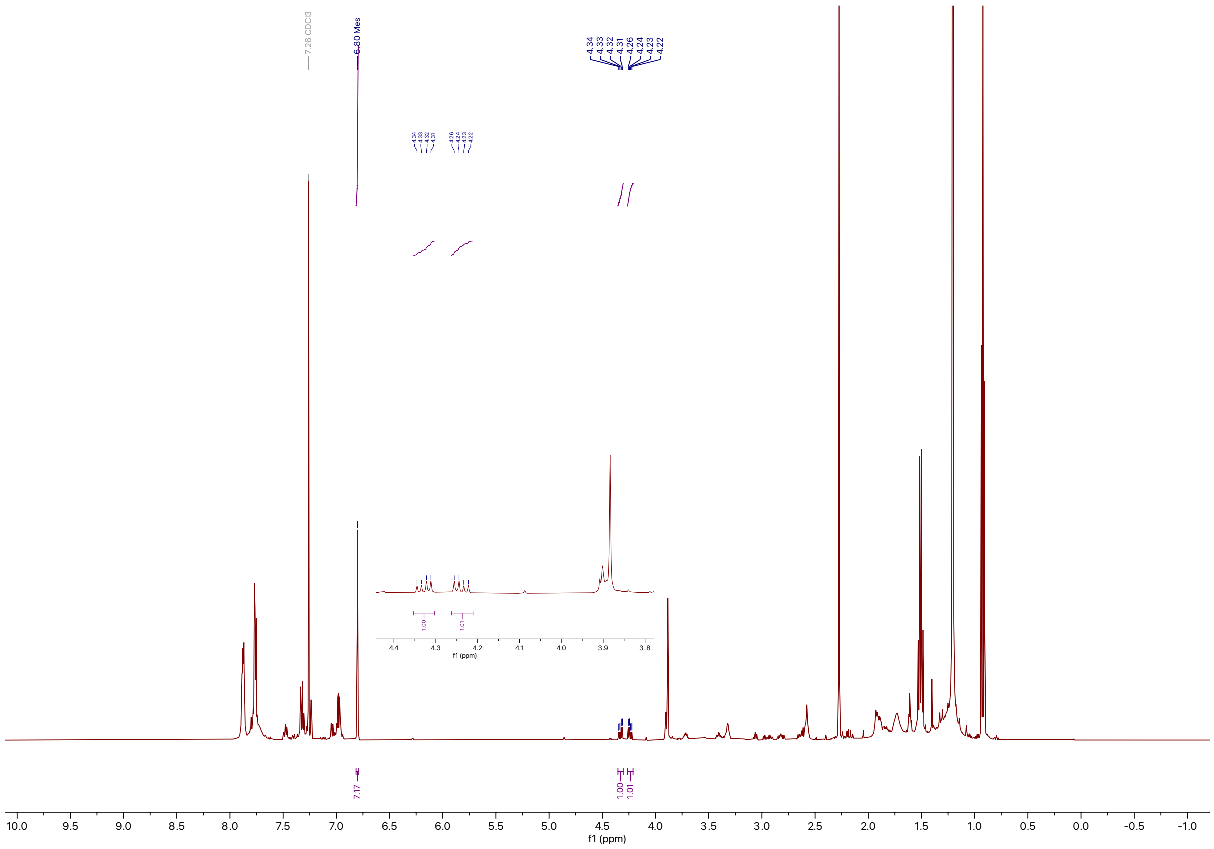

^{1}H NMR (500 MHz, $CDCl_3$) δ 7.79 (dd, *J* = 7.9, 1.8 Hz, 1H), 7.48 (ddd, *J* = 8.4, 7.4, 1.8 Hz, 1H), 7.33 (d, *J* = 8.2 Hz, 1H), 7.30 (d, *J* = 2.1 Hz, 1H), 7.04 (dd, *J* = 8.2, 2.1 Hz, 1H), 7.01 – 6.95 (m, 2H), 4.33 (dd, *J* = 11.3, 5.3 Hz, 1H), 4.24 (dd, *J* = 11.3, 5.7 Hz, 1H), 3.88 (s, 3H), 3.76 – 3.68 (m, 1H), 3.45 – 3.37 (m, 1H), 2.86 – 2.78 (m, 1H), 2.68 – 2.58 (m, 1H), 1.95 – 1.80 (m, 4H), 1.78 – 1.70 (m, 2H), 1.56 – 1.51 (m, 1H), 1.37 – 1.17 (m, 5H).

^{13}C NMR (126 MHz, DMSO-d_6) δ 165.55, 158.25, 143.36, 133.61, 130.81, 130.61, 130.38, 130.28, 128.76, 128.27, 120.07, 119.98, 112.60, 75.58, 73.27, 66.25, 55.72, 33.30, 32.76, 32.16, 30.10, 25.28, 23.61, 23.59.

(^{13}C NMR taken in DMSO-d_6 due to peak overlap with $CDCl_3$)

IR (film) v_{max} 2931, 2855, 1725, 1466, 1297, 1248, 1131, 1072, 754 cm^{-1}.

HRMS (ESI-TOF) *m/z* calculated for $C_{24}H_{29}Cl_2O_4^+$ ($[M+H]^+$) 451.1437, found 451.1441.

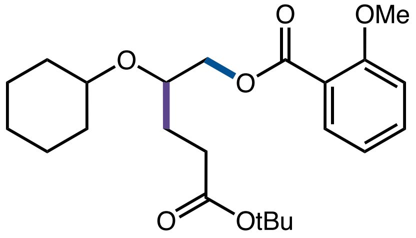

(±)-5-(*tert*-butoxy)-2-(cyclohexyloxy)-5-oxopentyl 2-methoxybenzoate (50): The title compound was prepared according to General Procedure C with Ni(acac)$_2$ (15 mol%, 75 µmol, 19.3 mg), (Ir[dF(CF$_3$)ppy]$_2$(dtbbpy))(PF$_6$) (2 mol%, 10 µmol, 11.2 mg), *tert*-butyl (1,3-dioxoisoindolin-2-yl) succinate (3 equiv, 1.5 mmol, 479.0 mg), 2-methoxybenzoic acid (1 equiv, 0.5 mmol, 76.1 mg), (vinyloxy)cyclohexane (3 equiv, 1.5 mmol, 189.3 mg, 212.5 µL), *tert*-butylimino-tri(pyrrolidino)phosphorane (0.2 equiv, 0.1 mmol, 31.2 mg, 30.6 µL), and 10 mL *t*-amyl alcohol. The reported 50% yield was obtained based on crude ^{1}H NMR assay with 74.3 mg of trimethoxybenzene as an internal standard (see below). The crude reaction mixture was concentrated, dissolved in DMSO/MeCN, and purified by automated reverse-phase chromatography (25 g C18 column, 20-75% gradient of 0.1% ammonium hydroxide in water/0.1% ammonium hydroxide in acetonitrile). Additional purification was attempted via automated flash chromatography (25 g high performance silica column, 0-14% ethyl acetate/hexanes), but the desired product was not fully resolved from impurities. Pure fractions were directly concentrated and the product dissolved in acetonitrile and filtered through a cotton plug into a 40 mL vial. The solvent was removed and the product dried on high-vac to yield the title compound as a pale yellow oil (90.2 mg, 0.222 mmol, 44% yield).

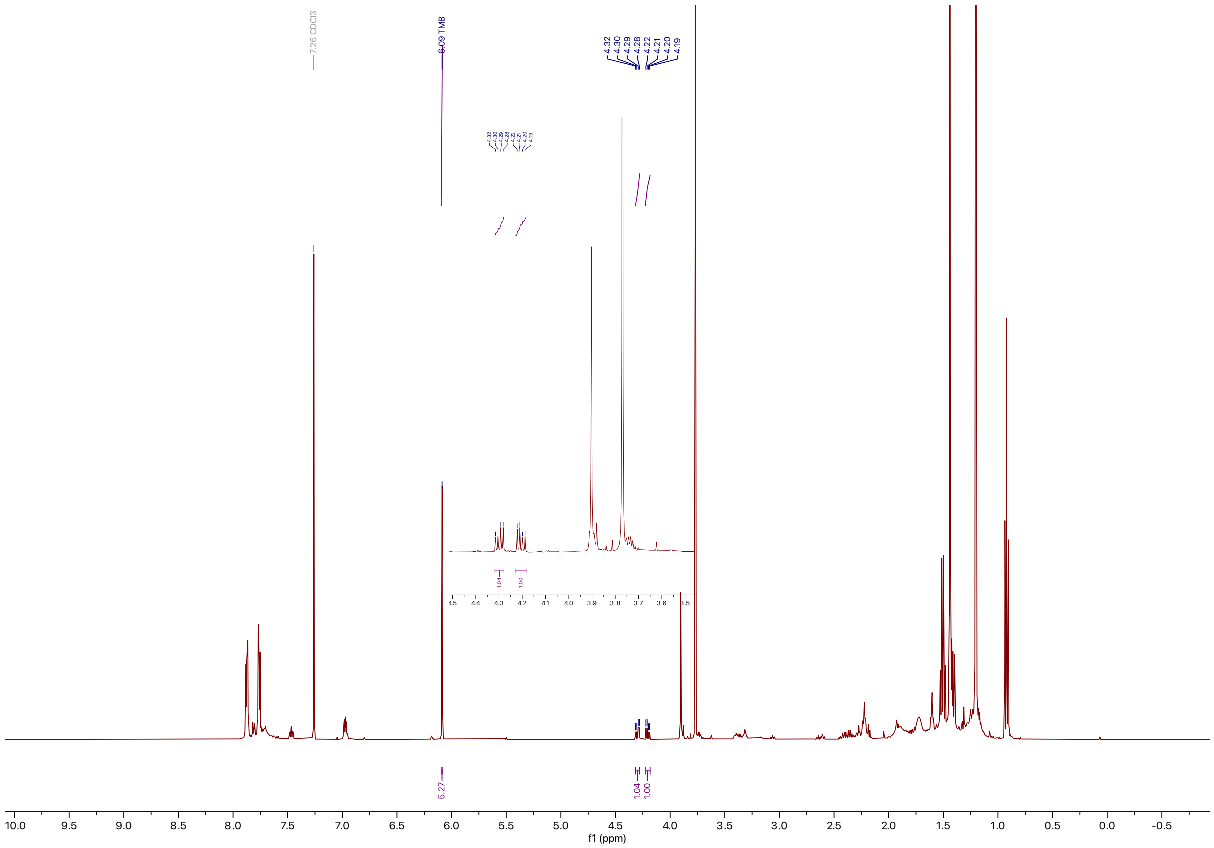

^{1}H NMR (500 MHz, CDCl$_3$) δ 7.80 (dd, *J* = 7.9, 1.8 Hz, 1H), 7.46 (ddd, *J* = 8.9, 7.4, 1.8 Hz, 1H), 6.99 – 6.94 (m, 2H), 4.29 (dd, *J* = 11.3, 5.3 Hz, 1H), 4.20 (dd, *J* = 11.3, 5.6 Hz, 1H), 3.90 (s, 3H), 3.77 – 3.69 (m, 1H), 3.44 – 3.35 (m, 1H), 2.47 – 2.29 (m, 2H), 1.99 – 1.84 (m, 3H), 1.84 – 1.67 (m, 3H), 1.56 – 1.47 (m, 1H), 1.43 (s, 9H), 1.32 – 1.13 (m, 5H).

^{13}C NMR (126 MHz, (CD$_3$)$_2$CO) δ 173.13, 166.61, 159.83, 134.21, 131.92, 121.70, 120.84, 113.25, 80.21, 77.01, 74.15, 67.15, 56.22, 34.07, 33.17, 31.83, 28.80, 28.28, 26.49, 24.79, 24.73. (^{13}C NMR taken in (CD$_3$)$_2$CO due to peak overlap with CDCl$_3$)

IR (film) v_{max} 2932, 2856, 1725, 1297, 1250, 1150, 1074, 754 cm^{-1}.

HRMS (ESI-TOF) *m/z* calculated for $C_{23}H_{34}NaO_6^+$ ([M+Na]$^+$) 429.2248, found 429.2243.

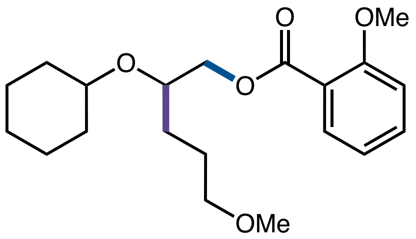

(±)-2-(cyclohexyloxy)-5-methoxypentyl 2-methoxybenzoate (51): The title compound was prepared according to General Procedure C with Ni(acac)$_2$ (15 mol%, 75 μmol, 19.3 mg), (Ir[dF(CF$_3$)ppy]$_2$(dtbbpy))(PF$_6$) (2 mol%, 10 μmol, 11.2 mg), 1,3-dioxoisoindolin-2-yl 4-methoxybutanoate (3 equiv, 1.5 mmol, 394.9 mg), 2-methoxybenzoic acid (1 equiv, 0.5 mmol, 76.1 mg), (vinyloxy)cyclohexane (3 equiv, 1.5 mmol, 189.3 mg, 212.5 μL), *tert*-butylimino-tri(pyrrolidino)phosphorane (0.2 equiv, 0.1 mmol, 31.2 mg, 30.6 μL), and 10 mL *t*-amyl alcohol. The crude reaction mixture was concentrated, dissolved in DMSO/MeCN, and purified by automated reverse-phase chromatography (25 g C18 column, 20-51% gradient of 0.1% ammonium hydroxide in water/0.1% ammonium hydroxide in acetonitrile). Fractions were directly concentrated and the product dissolved in acetonitrile and filtered through a cotton plug into a 40 mL vial. The solvent was removed and the product dried on high-vac to yield the title compound as a yellow oil (123.0 mg, 0.351 mmol, 70% yield).

^{1}H NMR (500 MHz, CDCl$_3$) δ 7.81 (dd, J = 7.9, 1.9 Hz, 1H), 7.47 (ddd, J = 8.4, 7.4, 1.8 Hz, 1H), 7.00 – 6.93 (m, 2H), 4.28 (dd, J = 11.3, 5.7 Hz, 1H), 4.22 (dd, J = 11.3, 5.3 Hz, 1H), 3.90 (s, 3H), 3.73 – 3.67 (m, 1H), 3.43 – 3.37 (m, 3H), 3.33 (s, 3H), 1.93 – 1.85 (m, 2H), 1.75 – 1.60 (m, 5H), 1.59 – 1.49 (m, 2H), 1.32 – 1.16 (m, 5H).

^{13}C NMR (126 MHz, DMSO-d_6) δ 165.53, 158.26, 133.54, 130.56, 120.04, 120.01, 112.56, 75.51, 73.68, 71.92, 66.38, 57.76, 55.68, 32.77, 32.12, 28.75, 25.28, 25.12, 23.57, 23.53.

(^{13}C NMR taken in DMSO-d_6 due to peak overlap with CDCl$_3$)

IR (film) v_{max} 2932, 2856, 1726, 1259, 1075, 751 cm^{-1}.

HRMS (ESI-TOF) *m/z* calculated for $C_{20}H_{31}O_5^+$ ([M+H]$^+$) 351.2166, found 351.2167.

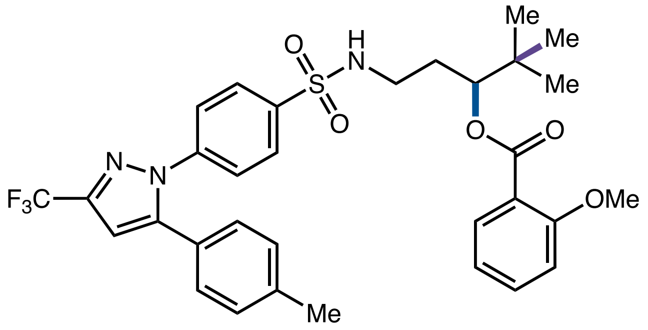

(±)-4,4-dimethyl-1-((4-(5-(*p*-tolyl)-3-(trifluoromethyl)-1*H*-pyrazol-1-yl)phenyl)sulfonamido) pentan-3-yl 2-methoxybenzoate (52): The title compound was prepared according to General Procedure B with Ni(acac)$_2$ (5 mol%, 25 µmol, 6.4 mg), KTp* (5 mol%, 25 µmol, 8.4 mg), (Ir[dF(CF$_3$)ppy]$_2$(dtbbpy))(PF$_6$) (2 mol%, 10 µmol, 11.2 mg), (1,3-dioxoisoindolin-2-yl)acetate (2 equiv, 1 mmol, 205.2 mg), 2-methoxybenzoic acid (1 equiv, 0.5 mmol, 76.1 mg), *N*-(4-methylpent-3-en-1-yl)-4-(5-(*p*-tolyl)-3-(trifluoromethyl)-1*H*-pyrazol-1-yl)benzene sulfonamide (2 equiv, 1 mmol, 463.5 mg, added before sparge), *tert*-butylimino-tri(pyrrolidino)phosphorane (0.2 equiv, 0.1 mmol, 31.2 mg, 30.6 µL), and 10 mL *t*-amyl alcohol. The crude reaction mixture was concentrated, dissolved in DMSO/MeCN, and purified by automated reverse-phase chromatography (25 g C18 column, 20-60% gradient of 0.1% ammonium hydroxide in water/0.1% ammonium hydroxide in acetonitrile). Additional purification via Preparative HPLC (XBridge BEH C18 OBD column, 30-70% gradient of 0.1% ammonium hydroxide in water/0.1% ammonium hydroxide in acetonitrile). Fractions were directly concentrated and the product dissolved in acetonitrile and filtered through a cotton plug into a 40 mL vial. The solvent was removed and the product dried on high-vac to yield the title compound as a yellow oil (160.6 mg, 0.255 mmol, 51% yield).

^{1}H NMR (500 MHz, CDCl$_3$) δ 7.81 – 7.76 (m, 2H), 7.68 (dd, *J* = 7.9, 1.8 Hz, 1H), 7.46 (ddd, *J* = 8.5, 7.4, 1.8 Hz, 1H), 7.39 – 7.34 (m, 2H), 7.16 (d, *J* = 7.7 Hz, 2H), 7.10 – 7.06 (m, 2H), 6.99 – 6.93 (m, 2H), 6.72 (s, 1H), 5.63 (dd, *J* = 8.3, 4.9 Hz, 1H), 4.90 (dd, *J* = 11.3, 2.3 Hz, 1H), 3.86 (s, 3H), 3.19 – 3.10 (m, 1H), 2.83 – 2.73 (m, 1H), 2.37 (s, 3H), 2.00 – 1.90 (m, 1H), 1.73 – 1.64 (m, 1H), 0.95 (s, 9H).

^{13}C NMR (126 MHz, CDCl$_3$) δ 167.52, 159.24, 145.29, 144.08 (q, *J* = 38.5 Hz), 142.39, 139.87, 139.85, 134.09, 131.89, 129.88, 128.82, 128.12, 125.87, 125.45, 121.25 (q, *J* = 269.1 Hz), 120.44, 119.56, 112.23, 106.33 (d, *J* = 2.3 Hz), 78.76, 55.96, 40.05, 34.67, 29.89, 26.03, 21.45.

^{19}F NMR (376 MHz, CDCl$_3$) δ -62.44.

IR (film) v_{max} 3275, 2965, 2873, 1702, 1600, 1470, 1340, 1301, 1237, 1161, 1096, 976, 843, 758 cm^{-1}.

HRMS (ESI-TOF) *m/z* calculated for $C_{32}H_{34}F_3N_3NaO_5S^+$ ([M+Na]$^+$) 652.2063, found 652.2082.

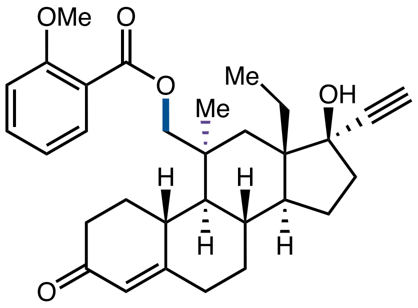

((8*S*,9*S*,10*R*,11*S*,13*S*,14*S*,17*R*)-13-ethyl-17-ethynyl-17-hydroxy-11-methyl-3-oxo-2,3,6,7,8,9,10,11,12,13,14,15,16,17-tetradecahydro-1*H*-cyclopenta[*a*]phenanthren-11-yl)methyl 2-methoxybenzoate (53): The title compound was prepared according to General Procedure B with Ni(acac)$_2$ (5 mol%, 25 µmol, 6.4 mg), KTp* (5 mol%, 25 µmol, 8.4 mg), (Ir[dF(CF$_3$)ppy]$_2$(dtbbpy))(PF$_6$) (2 mol%, 10 µmol, 11.2 mg), (1,3-dioxoisoindolin-2-yl)acetate (2 equiv, 1 mmol, 205.2 mg), 2-methoxybenzoic acid (1 equiv, 0.5 mmol, 76.1 mg), etonogestrel (2

equiv, 1 mmol, 324.5 mg, added before sparge), *tert*-butylimino-tri(pyrrolidino)phosphorane (0.2 equiv, 0.1 mmol, 31.2 mg, 30.6 μL), and 10 mL *t*-amyl alcohol. The crude reaction mixture was concentrated, dissolved in DMSO/MeCN, and purified by automated reverse-phase chromatography (25 g C18 column, 20-45% gradient of 0.1% ammonium hydroxide in water/0.1% ammonium hydroxide in acetonitrile). Fractions were directly concentrated and the product dissolved in acetonitrile and filtered through a cotton plug into a 40 mL vial. The solvent was removed and the product dried on high-vac to yield the title compound as a white solid (88.9 mg, 0.181 mmol, 36% yield).

^{1}H NMR (500 MHz, $CDCl_3$) δ 7.87 (dd, *J* = 7.9, 1.8 Hz, 1H), 7.51 (ddd, *J* = 8.4, 7.3, 1.8 Hz, 1H), 7.06 – 6.97 (m, 2H), 5.86 (s, 1H), 4.73 (d, *J* = 10.2 Hz, 1H), 4.26 (d, *J* = 11.3 Hz, 1H), 3.93 (s, 3H), 2.68 (s, 1H), 2.54 – 2.43 (m, 2H), 2.42 – 2.26 (m, 6H), 2.17 – 2.06 (m, 1H), 2.03 – 1.93 (m, 1H), 1.75 – 1.47 (m, 9H), 1.40 – 1.34 (m, 1H), 1.33 (s, 3H), 1.15 – 1.07 (m, 4H).

^{13}C NMR (126 MHz, $CDCl_3$) δ 199.89, 168.83, 166.61, 159.56, 133.94, 131.81, 124.12, 120.33, 119.92, 112.16, 87.83, 83.18, 75.35, 67.03, 57.14, 55.99, 51.84, 48.26, 40.92, 39.78, 39.62, 39.43, 38.20, 36.95, 35.12, 33.23, 30.56, 27.79, 22.91, 21.01, 9.90.

IR (solid) v_{max} 3417, 3294, 2935, 2877, 1710, 1657, 1600, 1299, 1244, 1129, 1048, 908, 726 cm^{-1}.

HRMS (ESI-TOF) *m/z* calculated for $C_{31}H_{39}O_5^+$ ($[M+H]^+$) 491.2792, found 491.2794.

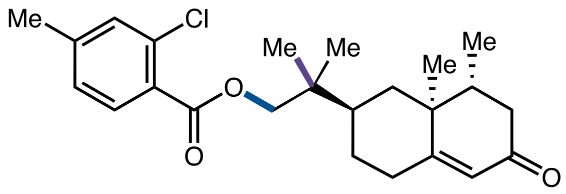

2-((2*R*,8*R*,8a*S*)-8,8a-dimethyl-6-oxo-1,2,3,4,6,7,8,8a-octahydronaphthalen-2-yl)-2-methylpropyl 2-chloro-4-methylbenzoate (54): The title compound was prepared according to General Procedure B with Ni(acac)$_2$ (5 mol%, 25 µmol, 6.4 mg), KTp* (5 mol%, 25 µmol, 8.4 mg), (Ir[dF(CF$_3$)ppy]$_2$(dtbbpy))(PF$_6$) (2 mol%, 10 µmol, 11.2 mg), (1,3-dioxoisoindolin-2-yl)acetate (2 equiv, 1 mmol, 205.2 mg), 2-chloro-4-methylbenzoic acid (1 equiv, 0.5 mmol, 85.3 mg), (+)-nootkatone (2 equiv, 1 mmol, 218.3 mg, added before sparge), *tert*-butylimino-tri(pyrrolidino)phosphorane (0.2 equiv, 0.1 mmol, 31.2 mg, 30.6 µL), and 10 mL *t*-amyl alcohol. The crude reaction mixture was concentrated, dissolved in DMSO/MeCN, and purified by automated reverse-phase chromatography (25 g C18 column, 20-100% gradient of 0.1% ammonium hydroxide in water/0.1% ammonium hydroxide in acetonitrile). Additional purification via Preparative HPLC (XBridge BEH C18 OBD column, 40-75% gradient of 0.1% ammonium hydroxide in water/0.1% ammonium hydroxide in acetonitrile). Fractions were directly concentrated and the product dissolved in acetonitrile and filtered through a cotton plug into a 40 mL vial. The solvent was removed and the product dried on high-vac to yield the title compound as a yellow oil (130.3 mg, 0.323 mmol, 65% yield).

^{1}H NMR (500 MHz, CDCl$_3$) δ 7.78 (d, *J* = 7.9 Hz, 1H), 7.29 (d, *J* = 0.9 Hz, 1H), 7.13 (ddd, *J* = 7.9, 1.7, 0.8 Hz, 1H), 5.74 (s, 1H), 4.17 (d, *J* = 11.0 Hz, 1H), 4.11 (d, *J* = 11.0 Hz, 1H), 2.49 – 2.34 (m, 5H), 2.31 – 2.19 (m, 2H), 2.05 – 1.89 (m, 4H), 1.87 – 1.80 (m, 1H), 1.29 – 1.15 (m, 1H), 1.05 (s, 3H), 1.00 (s, 3H), 0.98 (s, 3H), 0.96 (d, *J* = 6.8 Hz, 3H).

^{13}C NMR (126 MHz, CDCl$_3$) δ 199.76, 170.87, 166.01, 143.92, 133.75, 131.90, 131.81, 127.63, 127.22, 124.52, 72.36, 42.24, 40.75, 39.57, 39.41, 38.62, 36.19, 33.37, 27.56, 22.51, 22.20, 21.32, 17.04, 15.13.

IR (film) v_{max} 2965, 2878, 1730, 1669, 1289, 1252, 1125, 1048, 770 cm^{-1}.

HRMS (ESI-TOF) *m/z* calculated for $C_{24}H_{32}ClO_3^+$ ([M+H]$^+$) 403.2034, found 403.2036.

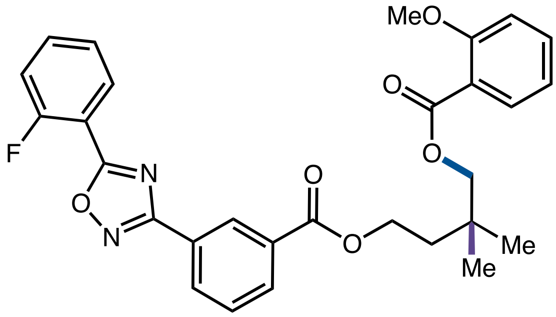

4-((3-(5-(2-fluorophenyl)-1,2,4-oxadiazol-3-yl)benzoyl)oxy)-2,2-dimethylbutyl 2-methoxy benzoate (55): The title compound was prepared according to General Procedure B with Ni(acac)$_2$ (5 mol%, 25 μmol, 6.4 mg), KTp* (5 mol%, 25 μmol, 8.4 mg), (Ir[dF(CF$_3$)ppy]$_2$(dtbbpy))(PF$_6$) (2 mol%, 10 μmol, 11.2 mg), (1,3-dioxoisoindolin-2-yl)acetate (2 equiv, 1 mmol, 205.2 mg), 2-methoxybenzoic acid (1 equiv, 0.5 mmol, 76.1 mg), 3-methylbut-3-en-1-yl 3-(5-(2-fluorophenyl)-1,2,4-oxadiazol-3-yl)benzoate (2 equiv, 1 mmol, 352.4 mg, added before sparge), *tert*-butylimino-tri(pyrrolidino)phosphorane (0.2 equiv, 0.1 mmol, 31.2 mg, 30.6 μL), and 10 mL *t*-amyl alcohol. The crude reaction mixture was concentrated, dissolved in DCM, and purified via automated flash chromatography (25 g high performance silica column, 0-15% ethyl acetate/hexanes). Additional purification via Preparative HPLC (XBridge BEH C18 OBD column, 20-80% gradient of 0.1% ammonium hydroxide in water/0.1% ammonium hydroxide in acetonitrile). Fractions were directly concentrated and the product dissolved in acetonitrile and filtered through a cotton plug

into a 40 mL vial. The solvent was removed and the product dried on high-vac to yield the title compound as a yellow oil (107.6 mg, 0.208 mmol, 42% yield).

^{1}H NMR (500 MHz, CDCl$_3$) δ 8.80 (t, *J* = 1.8 Hz, 1H), 8.33 (dt, *J* = 7.8, 1.5 Hz, 1H), 8.20 (td, *J* = 7.4, 1.8 Hz, 1H), 8.16 (dt, *J* = 7.9, 1.4 Hz, 1H), 7.81 (dd, *J* = 7.6, 1.8 Hz, 1H), 7.62 – 7.53 (m, 2H), 7.43 (ddd, *J* = 8.3, 7.4, 1.8 Hz, 1H), 7.32 (td, *J* = 7.7, 1.1 Hz, 1H), 7.27 (ddd, *J* = 10.5, 8.4, 1.1 Hz, 1H), 6.98 – 6.92 (m, 2H), 4.50 (t, *J* = 7.2 Hz, 2H), 4.11 (s, 2H), 3.88 (s, 3H), 1.94 (t, *J* = 7.2 Hz, 2H), 1.14 (s, 6H).

^{13}C NMR (126 MHz, CDCl$_3$) δ 173.08 (d, *J* = 4.3 Hz), 168.09, 166.47, 165.88, 160.84 (d, *J* = 260.8 Hz), 159.29, 134.79 (d, *J* = 8.7 Hz), 133.59, 132.18, 131.83, 131.72, 131.28, 131.03, 129.11, 128.71, 127.27, 124.80 (d, *J* = 3.7 Hz), 120.15, 120.12, 117.23 (d, *J* = 20.8 Hz), 112.75 (d, *J* = 11.4 Hz), 112.00, 72.81, 62.45, 55.85, 37.57, 33.60, 24.74.

^{19}F NMR (376 MHz, CDCl$_3$) δ -108.12 – -108.21 (m).

IR (film) v_{max} 2963, 2884, 1719, 1621, 1465, 1252, 1131, 1081, 825, 749 cm^{-1}.

HRMS (ESI-TOF) *m/z* calculated for $C_{29}H_{27}FN_2NaO_6^+$ ([M+Na]$^+$) 541.1745, found 541.1736.

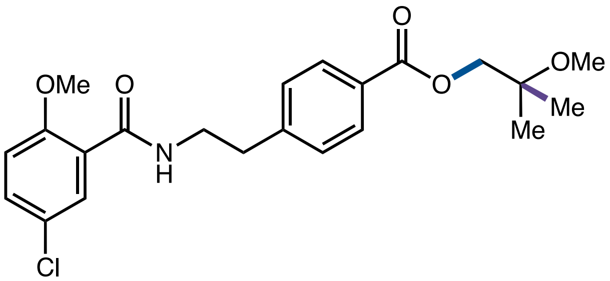

2-methoxy-2-methylpropyl 4-(2-(5-chloro-2-methoxybenzamido)ethyl)benzoate (56): The title compound was prepared according to a modified General Procedure B with Ni(acac)$_2$ (5 mol%, 25 μmol, 6.4 mg), KTp* (5 mol%, 25 μmol, 8.4 mg), (Ir[dF(CF$_3$)ppy]$_2$(dtbbpy))(PF$_6$) (2 mol%, 10 μmol, 11.2 mg), (1,3-dioxoisoindolin-2-yl)acetate (2 equiv, 1 mmol, 205.2 mg),

meglitinide (**S6**) (1 equiv, 0.5 mmol, 166.9 mg), 2-methoxyprop-1-ene (3 equiv, 1.5 mmol, 108.2 mg, 143.6 µL), *tert*-butylimino-tri(pyrrolidino)phosphorane (0.2 equiv, 0.1 mmol, 31.2 mg, 30.6 µL), and 10 mL *t*-amyl alcohol. The crude reaction mixture was concentrated, dissolved in DMSO/MeCN, and purified by automated reverse-phase chromatography (25 g C18 column, 20-50% gradient of 0.1% ammonium hydroxide in water/0.1% ammonium hydroxide in acetonitrile). Fractions were directly concentrated and the product dissolved in acetonitrile and filtered through a cotton plug into a 40 mL vial. The solvent was removed and the product dried on high-vac to yield the title compound as a yellow oil (127.8 mg, 0.304 mmol, 61% yield).

^{1}H NMR (500 MHz, $CDCl_3$) δ 8.17 (d, *J* = 2.8 Hz, 1H), 8.03 (d, *J* = 8.2 Hz, 2H), 7.79 (s, 1H), 7.40 – 7.31 (m, 3H), 6.86 (d, *J* = 8.8 Hz, 1H), 4.24 (s, 2H), 3.78 – 3.73 (m, 5H), 3.29 (s, 3H), 2.99 (t, *J* = 6.8 Hz, 2H), 1.29 (s, 6H).

^{13}C NMR (126 MHz, $CDCl_3$) δ 166.40, 164.09, 156.04, 145.02, 132.46, 132.15, 130.12, 129.13, 128.68, 126.94, 122.94, 112.93, 74.01, 69.39, 56.28, 50.10, 40.81, 35.79, 22.65.

IR (film) v_{max} 3393, 2977, 2832, 1716, 1655, 1530, 1482, 1271, 1122, 1020, 764 cm^{-1}.

HRMS (ESI-TOF) *m/z* calculated for $C_{22}H_{27}ClNO_5^+$ ($[M+H]^+$) 420.1572, found 420.1573.

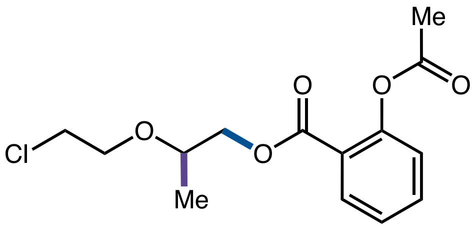

(±)-2-(2-chloroethoxy)propyl 2-acetoxybenzoate (57): The title compound was prepared according to a modified General Procedure B with $Ni(acac)_2$ (5 mol%, 25 µmol, 6.4 mg), KTp* (5 mol%, 25 µmol, 8.4 mg), $(Ir[dF(CF_3)ppy]_2(dtbbpy))(PF_6)$ (2 mol%, 10 µmol, 11.2 mg), (1,3-dioxoisoindolin-2-yl)acetate (2 equiv, 1 mmol, 205.2 mg), aspirin (1 equiv, 0.5 mmol, 90.1 mg),

(2-chloroethoxy)ethene (3 equiv, 1.5 mmol, 159.8 mg, 152.6 μL), *tert*-butylimino-tri(pyrrolidino)phosphorane (0.2 equiv, 0.1 mmol, 31.2 mg, 30.6 μL), and 10 mL *t*-amyl alcohol. The reported 50% yield was obtained based on crude ^{1}H NMR assay with 69.3 mg of mesitylene as an internal standard (see below). The crude reaction mixture was concentrated, dissolved in DMSO/MeCN, and purified by automated reverse-phase chromatography (25 g C18 column, 20-40% gradient of 0.1% ammonium hydroxide in water/0.1% ammonium hydroxide in acetonitrile). Fractions were directly concentrated and the product dissolved in acetonitrile and filtered through a cotton plug into a 40 mL vial. The solvent was removed and the product dried on high-vac to yield the title compound as a yellow oil with an unknown impurity. Additional purification via automated flash chromatography (10 g high performance silica column, 0-15% ethyl acetate/hexanes) allowed a small amount of pure material to be obtained for characterization.

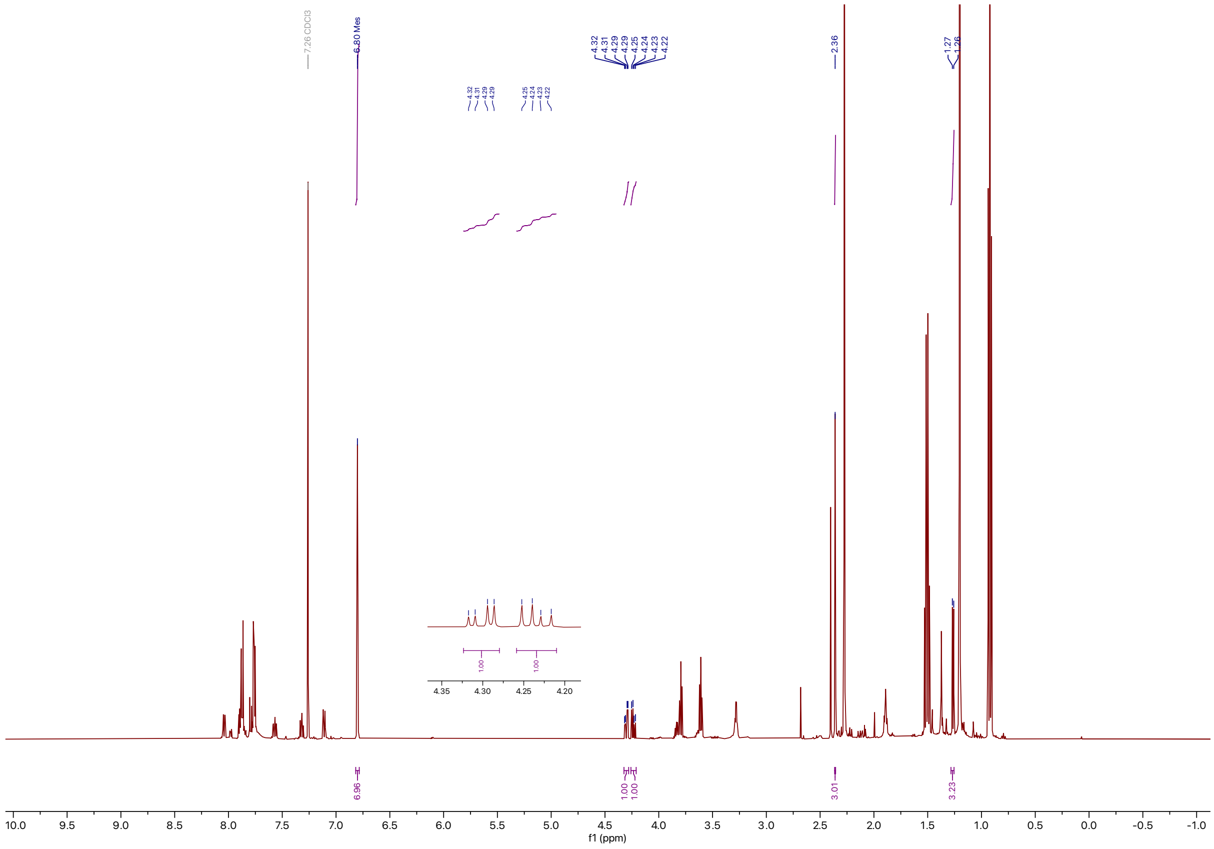

^{1}H NMR (500 MHz, CDCl$_3$) δ 8.04 (dd, *J* = 7.9, 1.7 Hz, 1H), 7.57 (ddd, *J* = 8.1, 7.4, 1.7 Hz, 1H), 7.32 (td, *J* = 7.6, 1.2 Hz, 1H), 7.11 (dd, *J* = 8.1, 1.2 Hz, 1H), 4.30 (dd, *J* = 11.6, 4.0 Hz, 1H), 4.23 (dd, *J* = 11.6, 6.3 Hz, 1H), 3.87 – 3.76 (m, 3H), 3.61 (t, *J* = 5.9 Hz, 2H), 2.36 (s, 3H), 1.26 (d, *J* = 6.4 Hz, 3H).

^{13}C NMR (126 MHz, CDCl$_3$) δ 169.81, 164.29, 150.91, 134.08, 131.83, 126.12, 123.95, 123.15, 74.21, 69.61, 67.79, 43.15, 21.15, 17.10.

IR (film) v_{max} 2975, 2851, 1769, 1719, 1251, 1188, 1078, 704 cm^{-1}.

HRMS (ESI-TOF) *m/z* calculated for $C_{14}H_{18}ClO_5^+$ ([M+H]$^+$) 301.0837, found 301.0839.

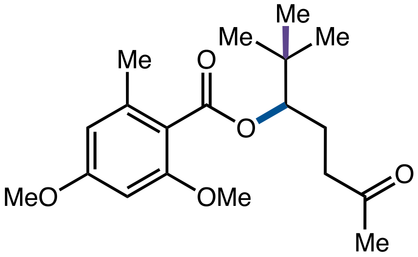

(±)-2,2-dimethyl-6-oxoheptan-3-yl 2,4-dimethoxy-6-methylbenzoate (58): The title compound was prepared according to a modified General Procedure B with Ni(acac)$_2$ (5 mol%, 25 µmol, 6.4 mg), KTp* (5 mol%, 25 µmol, 8.4 mg), (Ir[dF(CF$_3$)ppy]$_2$(dtbbpy))(PF$_6$) (2 mol%, 10 µmol, 11.2 mg), (1,3-dioxoisoindolin-2-yl)acetate (2 equiv, 1 mmol, 205.2 mg), 2,4-dimethoxy-6-methylbenzoic acid (orsellinic acid O-Me, **S7**) (1 equiv, 0.5 mmol, 98.1 mg), 6-methylhept-5-en-2-one (3 equiv, 1.5 mmol, 189.3 mg, 221.4 µL), *tert*-butylimino-tri(pyrrolidino)phosphorane (0.2 equiv, 0.1 mmol, 31.2 mg, 30.6 µL), and 10 mL *t*-amyl alcohol. The crude reaction mixture was concentrated, dissolved in DMSO/MeCN, and purified by automated reverse-phase chromatography (25 g C18 column, 20-45% gradient of 0.1% ammonium hydroxide in water/0.1% ammonium hydroxide in acetonitrile). Fractions were directly concentrated and the product

dissolved in acetonitrile and filtered through a cotton plug into a 40 mL vial. The solvent was removed and the product dried on high-vac to yield the title compound as a yellow oil (116.7 mg, 0.347 mmol, 69% yield).

^{1}H NMR (500 MHz, CDCl$_3$) δ 6.36 – 6.28 (m, 2H), 4.93 (dd, *J* = 11.0, 1.9 Hz, 1H), 3.80 (s, 3H), 3.78 (s, 3H), 2.67 – 2.58 (m, 2H), 2.33 (s, 3H), 2.15 (s, 3H), 2.06 – 1.98 (m, 1H), 1.75 – 1.65 (m, 1H), 0.98 (s, 9H).

^{13}C NMR (126 MHz, CDCl$_3$) δ 208.60, 168.68, 161.30, 158.23, 138.21, 117.20, 106.95, 96.44, 81.22, 55.71, 55.49, 40.45, 34.83, 30.27, 26.20, 23.94, 20.44.

IR (film) ν_{max} 2964, 2841, 1714, 1265, 1159, 1050, 830 cm^{-1}.

HRMS (ESI-TOF) *m/z* calculated for $C_{19}H_{28}NaO_5^+$ ([M+Na]$^+$) 359.1829, found 359.1836.

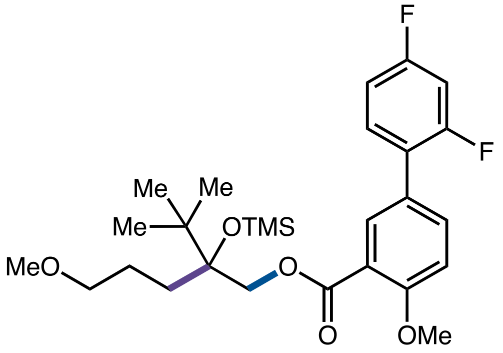

(±)-2-(*tert*-butyl)-5-methoxy-2-((trimethylsilyl)oxy)pentyl 2',4'-difluoro-4-methoxy-[1,1'-biphenyl]-3-carboxylate (59): The title compound was prepared according to General Procedure C with Ni(acac)$_2$ (15 mol%, 75 µmol, 19.3 mg), (Ir[dF(CF$_3$)ppy]$_2$(dtbbpy))(PF$_6$) (2 mol%, 10 µmol, 11.2 mg), 1,3-dioxoisoindolin-2-yl 4-methoxybutanoate (3 equiv, 1.5 mmol, 394.9 mg), 2',4'-difluoro-4-methoxy-[1,1'-biphenyl]-3-carboxylic acid (diflunisal O-Me, **S8**) (1 equiv, 0.5 mmol, 132.1 mg), ((3,3-dimethylbut-1-en-2-yl)oxy)trimethylsilane (3 equiv, 1.5 mmol, 258.5 mg, 324.0 µL), *tert*-butylimino-tri(pyrrolidino)phosphorane (0.2 equiv, 0.1 mmol, 31.2 mg, 30.6 µL), and 10

mL *t*-amyl alcohol. The crude reaction mixture was concentrated, dissolved in DMSO/MeCN, and purified by automated reverse-phase chromatography (25 g C18 column, 20-80% gradient of 0.1% ammonium hydroxide in water/0.1% ammonium hydroxide in acetonitrile). Additional purification via Preparative HPLC (XBridge BEH C18 OBD column, 40-100% gradient of 0.1% ammonium hydroxide in water/0.1% ammonium hydroxide in acetonitrile). Fractions were directly concentrated and the product dissolved in acetonitrile and filtered through a cotton plug into a 40 mL vial. The solvent was removed and the product dried on high-vac to yield the title compound as a yellow oil (134.5 mg, 0.264 mmol, 53% yield).

^{1}H NMR (500 MHz, $CDCl_3$) δ 7.94 (dd, *J* = 2.4, 1.2 Hz, 1H), 7.65 – 7.60 (m, 1H), 7.41 – 7.34 (m, 1H), 7.05 (d, *J* = 8.7 Hz, 1H), 6.98 – 6.87 (m, 2H), 4.43 (d, *J* = 11.5 Hz, 1H), 4.24 (d, *J* = 11.5 Hz, 1H), 3.93 (s, 3H), 3.37 – 3.33 (m, 2H), 3.25 (s, 3H), 1.90 – 1.76 (m, 1H), 1.74 – 1.65 (m, 3H), 0.99 (s, 9H), 0.12 (s, 9H).

^{13}C NMR (126 MHz, $CDCl_3$) δ 165.90, 162.33 (dd, J = 249.0, 11.8 Hz), 159.84 (dd, J = 250.3, 11.8 Hz), 158.88, 133.96 (d, J = 3.5 Hz), 132.15 (d, J = 2.8 Hz), 131.17 (dd, J = 9.4, 4.8 Hz), 126.99 (d, J = 1.4 Hz), 124.10 (dd, J = 13.5, 3.9 Hz), 120.35, 112.17, 111.80 (dd, J = 21.2, 3.8 Hz), 104.58 (dd, J = 26.7, 25.2 Hz), 81.11, 73.72, 66.99, 58.52, 55.99, 38.99, 30.54, 26.41, 25.07, 2.78.

^{19}F NMR (376 MHz, $CDCl_3$) δ -111.44 (p, *J* = 7.6 Hz), -113.68 (q, *J* = 9.0 Hz).

IR (film) v_{max} 2957, 2827, 1729, 1491, 1250, 1070, 753 cm^{-1}.

HRMS (ESI-TOF) *m/z* calculated for $C_{27}H_{38}F_2NaO_5Si^+$ ($[M+Na]^+$) 531.2349, found 531.2351.

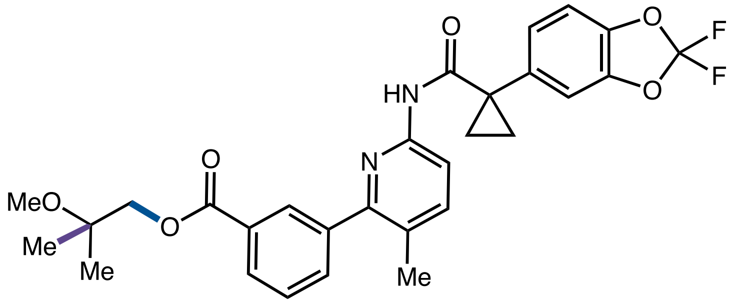

2-methoxy-2-methylpropyl 3-(6-(1-(2,2-difluorobenzo[*d*][1,3]dioxol-5-yl)cyclopropane-1-carboxamido)-3-methylpyridin-2-yl)benzoate (60): The title compound was prepared according to a modified General Procedure B with Ni(acac)$_2$ (5 mol%, 25 μmol, 6.4 mg), KTp* (5 mol%, 25 μmol, 8.4 mg), (Ir[dF(CF$_3$)ppy]$_2$(dtbbpy))(PF$_6$) (2 mol%, 10 μmol, 11.2 mg), (1,3-dioxoisoindolin-2-yl)acetate (2 equiv, 1 mmol, 205.2 mg), lumacaftor (1 equiv, 0.5 mmol, 226.2 mg), 2-methoxyprop-1-ene (3 equiv, 1.5 mmol, 108.2 mg, 143.6 μL), *tert*-butylimino-tri(pyrrolidino)phosphorane (0.2 equiv, 0.1 mmol, 31.2 mg, 30.6 μL), and 10 mL *t*-amyl alcohol. The crude reaction mixture was concentrated, dissolved in DMSO/MeCN, and purified by automated reverse-phase chromatography (25 g C18 column, 20-100% gradient of 0.1% ammonium hydroxide in water/0.1% ammonium hydroxide in acetonitrile). Additional purification via Preparative HPLC (XBridge BEH C18 OBD column, 40-80% gradient of 0.1% ammonium hydroxide in water/0.1% ammonium hydroxide in acetonitrile). Fractions were directly concentrated and the product dissolved in acetonitrile and filtered through a cotton plug into a 40 mL vial. The solvent was removed and the product dried on high-vac to yield the title compound as a white solid (90.4 mg, 0.168 mmol, 34% yield).

^{1}H NMR (500 MHz, CDCl$_3$) δ 8.14 – 8.05 (m, 3H), 7.68 (b, 1H), 7.64 – 7.57 (m, 2H), 7.50 (t, *J* = 7.7 Hz, 1H), 7.23 (dd, *J* = 8.2, 1.8 Hz, 1H), 7.18 (d, *J* = 1.8 Hz, 1H), 7.07 (d, *J* = 8.1 Hz, 1H), 4.25 (s, 2H), 3.28 (s, 3H), 2.26 (s, 3H), 1.75 (q, *J* = 3.8 Hz, 2H), 1.27 (s, 6H), 1.16 (q, *J* = 3.9 Hz, 2H).

^{13}C NMR (126 MHz, $CDCl_3$) δ 171.90, 166.22, 155.38, 149.06, 144.30, 143.77, 141.15, 140.15, 135.03, 133.61, 131.82 (t, J = 256.2 Hz), 130.37, 130.28, 129.53, 128.57, 127.06, 126.72, 113.07, 112.50, 110.31, 73.98, 69.49, 50.07, 31.34, 22.65, 19.29, 17.33.

^{19}F NMR (376 MHz, $CDCl_3$) δ -49.59.

IR (film) v_{max} 3407, 2979, 2832, 1720, 1680, 1235, 1156, 1080, 732 cm^{-1}.

HRMS (ESI-TOF) *m/z* calculated for $C_{29}H_{29}F_2N_2O_6^+$ ($[M+H]^+$) 539.1988, found 539.1983.

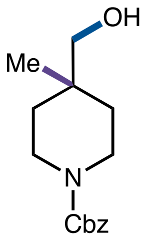

benzyl 4-(hydroxymethyl)-4-methylpiperidine-1-carboxylate (61) The title compound was prepared according to Procedure E. Oxyalkylation was first performed with $Ni(acac)_2$ (5 mol%, 25 μmol, 6.4 mg), KTp* (5 mol%, 25 μmol, 8.4 mg), $(Ir[dF(CF_3)ppy]_2(dtbbpy))(PF_6)$ (2 mol%, 10 μmol, 11.2 mg), (1,3-dioxoisoindolin-2-yl)acetate (2 equiv, 1 mmol, 205.2 mg), 2-methoxybenzoic acid (1 equiv, 0.5 mmol, 76.1 mg), benzyl 4-methylenepiperidine-1-carboxylate (2 equiv, 1 mmol, 231.3 mg, 208.4 μL), *tert*-butylimino-tri(pyrrolidino)phosphorane (0.2 equiv, 0.1 mmol, 31.2 mg, 30.6 μL), and 10 mL *t*-amyl alcohol. The crude reaction mixture was concentrated to removed *tert*-amyl alcohol and carried directly to the next step. Deprotection was next performed with 4 mL THF and lithium hydroxide (20 equiv, 10 mmol, 240 mg) in 4 mL H_2O. The crude reaction mixture was transferred to a separatory funnel. Additional water/brine was added and the mixture extracted with ethyl acetate (3 x 100 mL). The combined organics were dried, filtered through celite, and concentrated. The resulting crude residue was purified via Preparative HPLC (XBridge BEH C18 OBD column, 20-34% gradient of 0.1% ammonium hydroxide in water/0.1% ammonium

hydroxide in acetonitrile). Fractions were directly concentrated and the product dissolved in acetonitrile and filtered through a cotton plug into a 40 mL vial. The solvent was removed and the product dried on high-vac to yield the title compound as a pale yellow oil (82.0 mg, 0.311 mmol, 62% yield over two steps).

^{1}H NMR (500 MHz, CDCl$_3$) (summary of rotamers) δ 7.38 – 7.29 (m, 5H), 5.13 (s, 2H), 3.82 – 3.71 (m, 2H), 3.39 (s, 2H), 3.22 (ddd, J = 13.7, 10.2, 3.5 Hz, 2H), 1.53 – 1.43 (m, 3H), 1.36 – 1.28 (m, 2H), 1.00 (s, 3H).

^{13}C NMR (126 MHz, CDCl$_3$) (summary of rotamers) δ 155.53, 137.07, 128.62, 128.08, 127.99, 71.81, 71.75, 67.13, 40.09, 33.96, 33.22, 20.74.

IR (film) v_{max} 3463, 2933, 2872, 1699, 1433, 1258, 1178, 751 cm^{-1}.

HRMS (ESI-TOF) *m/z* calculated for $C_{15}H_{22}NO_3^+$ ([M+H]$^+$) 264.1594, found 264.1595.

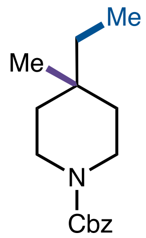

benzyl 4-ethyl-4-methylpiperidine-1-carboxylate (64): Based on literature procedure,[12] an oven-dried 40 mL vial with X-shaped stir bar was charged with 5,7-di-*tert*-butyl-3-phenylbenzo[*d*]oxazol-3-ium tetrafluoroborate (2.7 equiv, 1.35 mmol, 534 mg) and benzyl 4-(hydroxymethyl)-4-methylpiperidine-1-carboxylate (1 equiv, 0.5 mmol, 131.7 mg). The vial was placed under vacuum and refilled with nitrogen gas three times. 5 mL of dry methyl *tert*-butyl ether and methanol (1.5 equiv, 0.75 mmol, 24.0 mg, 30.3 μL) was added and the mixture stirred under N_2 for 5 minutes. Pyridine (2.7 equiv, 1.35 mmol, 106.8 mg, 108.7 μL) added over 30 seconds while vigorously stirring. The resulting mixture was stirred at room temperature for 45

minutes. To a second dry 40 mL vial was added (Ir[dF(CF_3)ppy]$_2$(dtbbpy))(PF_6) (1 mol%, 5 µmol, 5.6 mg), quinuclidine (5 equiv, 2.5 mmol, 278 mg), and 5 mL of dry DMSO. This mixture was sonicated until homogenous, then transferred under air to the vial containing the activated alcohol along with Ni(acac)$_2$ (25 mol%, 125 µmol, 32.1 mg) and benzoyl peroxide (1.5 equiv, 0.75 mmol, 182 mg) added as a single portion. The vial was capped and sonicated for 1 minute until homogenous. The reaction was irradiated with 450 nm light for 1 hour (m2 450 nm LED plate, 100% light intensity, 6800 rpm fan speed, 500 rpm stirring). After irradiation, the vial was removed from the photoreactor and poured into 30 mL of 5 wt% aqueous LiCl in a separatory funnel. EtOAc was used to rinse the vial into the separatory funnel and the mixture extracted with EtOAc (5 x 40 mL). The combined organics were washed with brine, dried over Na_2SO_4, and concentrated. The residue was purified via automated flash chromatography (50 g high performance silica column, 0-20% ethyl acetate/hexanes). Additional purification via Preparative HPLC (XBridge BEH C18 OBD column, 40-100% gradient of 0.1% ammonium hydroxide in water/0.1% ammonium hydroxide in acetonitrile). Fractions were directly concentrated and the product dissolved in acetonitrile and filtered through a cotton plug into a 40 mL vial. The solvent was removed and the product dried on high-vac to yield the title compound as a clear oil (67.2 mg, 0.257 mmol, 51% yield).

^{1}H NMR (500 MHz, CDCl$_3$) δ 7.40 – 7.28 (m, 5H), 5.13 (s, 2H), 3.63 (ddd, *J* = 13.5, 6.2, 4.5 Hz, 2H), 3.29 (ddd, *J* = 13.4, 9.1, 3.8 Hz, 2H), 1.43 – 1.25 (m, 6H), 0.90 (s, 3H), 0.82 (t, *J* = 7.5 Hz, 3H).

^{13}C NMR (126 MHz, CDCl$_3$) δ 155.54, 137.17, 128.57, 128.00, 127.93, 67.01, 40.33, 36.42, 33.94, 31.39, 22.91, 7.63.

IR (film) v_{max} 2932, 2877, 1698, 1245, 1227, 1093, 734 cm^{-1}.

HRMS (ESI-TOF) *m/z* calculated for $C_{16}H_{24}NO_2^+$ ($[M+H]^+$) 262.1802, found 262.1802.

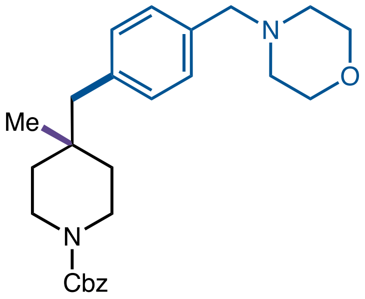

benzyl 4-methyl-4-(4-(morpholinomethyl)benzyl)piperidine-1-carboxylate (65): Based on literature procedure,[13] an oven-dried 20 mL vial with X-shaped stir bar was charged with 5,7-di-*tert*-butyl-3-phenylbenzo[*d*]oxazol-3-ium tetrafluoroborate (1.6 equiv, 0.8 mmol, 316 mg) and benzyl 4-(hydroxymethyl)-4-methylpiperidine-1-carboxylate (1.75 equiv, 0.875 mmol, 230.4 mg). The vial was placed under vacuum and refilled with nitrogen gas three times. 4 mL of dry methyl *tert*-butyl ether was added and the mixture stirred under N_2 for 5 minutes. Pyridine (1.6 equiv, 0.8 mmol, 63.3 mg, 64.4 µL) added over 30 seconds while vigorously stirring. The resulting mixture was stirred at room temperature for 30 minutes. To a separate 40 mL vial with X-shaped stir bar was added (Ir[ppy]$_2$(dtbbpy))(PF$_6$) (1.5 mol%, 7.5 µmol, 6.7 mg), NiBr$_2$(dtbbpy) (5 mol%, 25 µmol, 12.2 mg), quinuclidine (1.75 equiv, 0.875 mmol, 97.3 mg), 4-(4-bromobenzyl)morpholine (1 equiv, 0.5 mmol, 128.1 mg), and 5 mL of dry DMA. Under air, the heterogeneous solution in the 20 mL vial was transferred to a syringe filter and filtered into the 40 mL vial. The vial was capped, sparged with N_2 for 15 minutes, and sealed with parafilm. The reaction was irradiated with 450 nm light for 2 hour (m2 450 nm LED plate, 100% light intensity, 6800 rpm fan speed, 1500 rpm stirring). The crude reaction mixture was concentrated and purified by via automated flash chromatography (50 g high performance silica column, 0-100% ethyl acetate/hexanes). Additional purification via automated reverse-phase chromatography (25 g C18 column, 20-70% gradient of

0.1% ammonium hydroxide in water/0.1% ammonium hydroxide in acetonitrile). Fractions were directly concentrated and the product dissolved in acetonitrile and filtered through a cotton plug into a 40 mL vial. The solvent was removed and the product dried on high-vac to yield the title compound as a white solid (153.3 mg, 0.363 mmol, 73% yield).

^{1}H NMR (500 MHz, $(CD_3)_2CO$) δ 7.41 – 7.34 (m, 4H), 7.34 – 7.28 (m, 1H), 7.25 (d, J = 7.6 Hz, 2H), 7.13 (d, J = 8.0 Hz, 2H), 5.09 (s, 2H), 3.75 (dt, J = 13.7, 5.0 Hz, 2H), 3.60 (t, J = 4.6 Hz, 4H), 3.45 (s, 2H), 3.25 (b, 2H), 2.60 (s, 2H), 2.37 (b, 4H), 1.51 – 1.41 (m, 2H), 1.33 – 1.25 (m, 2H), 0.93 (s, 3H).

^{13}C NMR (126 MHz, $(CD_3)_2CO$) δ 155.63, 138.49, 137.69, 136.88, 131.41, 129.29, 129.25, 128.61, 128.55, 67.48, 67.09, 63.63, 54.48, 48.40, 40.82, 37.18, 33.40, 23.45.

IR (film) v_{max} 2916, 2851, 2804, 1699, 1243, 1117, 867, 698 cm^{-1}.

HRMS (ESI-TOF) *m/z* calculated for $C_{26}H_{35}N_2O_3^+$ ($[M+H]^+$) 423.2642, found 423.2643.

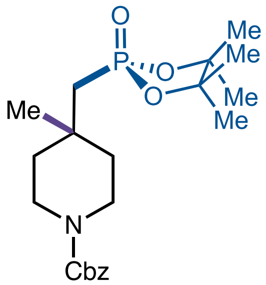

benzyl 4-methyl-4-((4,4,5,5-tetramethyl-2-oxido-1,3,2-dioxaphospholan-2-yl)methyl) piperidine-1-carboxylate (66): Based on literature procedure,[14] an oven-dried 4 mL vial was charged with a stir bar and 2-pyridyldiphenylphosphine (2.2 equiv, 1.1 mmol, 289.6 mg). The vial was placed under vacuum and refilled with nitrogen gas three times. 0.8 mL of acetonitrile was added and the mixture stirred under N_2 for 1 minute. Diisopropyl azodicarboxylate (2.2 equiv, 1.1 mmol, 222.4 mg, 216.5 μL) was added dropwise against the flow of nitrogen and the resulting

orange solution stirred at room temperature for 20 minutes. The entire solution was transferred to a new dry 4 mL vial under N_2 containing a stir bar and 4,4,5,5-tetramethyl-1,3,2-dioxaphospholane 2-oxide (2 equiv, 1 mmol, 164.1 mg) with a 0.1 mL wash of acetonitrile ensuring complete transfer. This mixture was stirred at room temperature under N_2 for 30 minutes (during which time the separate alcohol activation described below was started). The resulting yellow solution was transferred to a new dry 4 mL vial under N_2 containing a stir bar and 3-chloro-4-((4-cyanophenyl)(hydroxy)methyl)benzonitrile (2.2 equiv, 1.1 mmol, 295.6 mg) with a 0.1 mL wash of acetonitrile ensuring complete transfer. The solution was stirred at room temperature under N_2 for 1 hour. To a separate 8 mL vial with stir bar was added 5,7-di-*tert*-butyl-3-(4-(trifluoromethyl)phenyl)benzo[*d*]oxazol-3-ium tetrafluoroborate (1.2 equiv, 0.6 mmol, 278.0 mg) and benzyl 4-(hydroxymethyl)-4-methylpiperidine-1-carboxylate (1 equiv, 0.5 mmol, 131.7 mg). The vial was placed under vacuum and refilled with nitrogen gas three times. 5 mL of dry methyl *tert*-butyl ether was added and the mixture stirred under N_2 for 2 minutes. Pyridine (1.2 equiv, 0.6 mmol, 47.5 mg, 48.3 μL) added over 30 seconds while vigorously stirring. The resulting mixture was stirred at room temperature for approximately 1 hour. Under air, the heterogeneous solution was transferred to a syringe filter and filtered into a separate oven-dried 40 mL vial. Complete transfer of the activated alcohol was ensured by washing the vial with 1.25 mL of methyl *tert*-butyl ether followed by filtration through the same syringe filter (2x). The solvent was carefully removed via rotary evaporation (~250 mbar to 100 mbar, 35 ºC) to give a pale yellow oil. The oil was carefully dried on high vacuum for ~5 minutes to yield a voluminous off-white solid. To the 40 mL vial was added an X-shaped stir bar, CsOAc (1 equiv, 0.5 mmol, 96.0 mg), ($Ir[dF(CF_3)ppy]_2(dtbbpy))(PF_6)$ (1 mol%, 5 μmol, 5.6 mg), and 2.5 mL DMSO. The vial was capped and sparged with N_2 for 10 minutes. The entirety of the acetonitrile solution from the 4 mL

vial was transferred to the 40 mL vial under N_2 with a 0.1 mL wash of acetonitrile ensuring complete transfer. The vial was sealed with parafilm and electrical tape and the reaction irradiated with 450 nm light for 6 hours (m2 450 nm LED plate, 100% light intensity, 6800 rpm fan speed, 1000 rpm stirring). After irradiation, the vial was poured into a 100 mL separatory funnel. EtOAc (30 mL) was added and the organics washed with water (4 x 25 mL). The organics were dried over Na_2SO_4 and concentrated. The residue was purified via automated flash chromatography (50 g high performance silica column, 40-100% ethyl acetate/hexanes). Fractions were directly concentrated and the product dissolved in dichloromethane and filtered through a cotton plug into a 40 mL vial. The solvent was removed and the product dried on high-vac to yield the title compound as a dark oil (171.6 mg, 0.419 mmol, 84% yield).

^{1}H NMR (500 MHz, CDCl$_3$) δ 7.37 – 7.27 (m, 5H), 5.12 (s, 2H), 3.61 – 3.53 (m, 2H), 3.43 – 3.35 (m, 2H), 1.94 – 1.84 (m, 2H), 1.67 – 1.59 (m, 2H), 1.58 – 1.51 (m, 2H), 1.48 (s, 6H), 1.33 (s, 6H), 1.25 (s, 3H).

^{13}C NMR (126 MHz, CDCl$_3$) δ 155.44, 137.01, 128.60, 128.07, 127.99, 87.94 (d, J = 1.8 Hz), 67.14, 40.17, 40.16 (d, J = 128.2 Hz), 37.93, 31.39 (d, J = 3.2 Hz), 25.04 (d, J = 3.7 Hz), 24.87 (d, J = 3.7 Hz), 24.22 (d, J = 5.5 Hz).

^{31}P NMR (162 MHz, CDCl$_3$) δ 40.98.

IR (film) v_{max} 2988, 2952, 1699, 1431, 1275, 1175, 961, 750 cm^{-1}.

HRMS (ESI-TOF) *m/z* calculated for $C_{21}H_{33}NO_5P^+$ ([M+H]$^+$) 410.2091, found 410.2079.

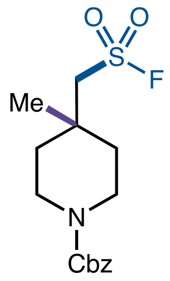

benzyl 4-((fluorosulfonyl)methyl)-4-methylpiperidine-1-carboxylate (67): Based on literature procedure,[15] an oven-dried 40 mL vial with X-shaped stir bar was charged with 5,7-di-*tert*-butyl-3-phenylbenzo[*d*]oxazol-3-ium tetrafluoroborate (1.2 equiv, 0.6 mmol, 237.1 mg) and benzyl 4-(hydroxymethyl)-4-methylpiperidine-1-carboxylate (1 equiv, 0.5 mmol, 131.7 mg). The vial was placed under vacuum and refilled with nitrogen gas three times. 5 mL of dry methyl *tert*-butyl ether was added and the mixture stirred under N_2 for 5 minutes. Pyridine (1.2 equiv, 0.6 mmol, 47.5 mg, 48.3 µL) added over 30 seconds while vigorously stirring. The resulting mixture was stirred at room temperature for 30 minutes. Under air, the heterogeneous solution was transferred to a syringe filter and filtered into a separate oven-dried 40 mL vial containing an X-shaped stir bar, tetrabutylammonium chloride (2 equiv, 1 mmol, 277.9 mg), $K_2S_2O_5$ (2 equiv, 1 mmol, 222.3 mg), $Zn(OAc)_4$ (4 equiv, 2 mmol, 367.0 mg), (Ir[dF(CF_3)ppy]$_2$(dtbbpy))(PF_6) (1 mol%, 5 µmol, 5.6 mg), 7 mL dimethylformamide, and 360.3 µL of water. Complete transfer of the activated alcohol was ensured by washing the vial with 1 mL of methyl *tert*-butyl ether followed by filtration through the same syringe filter (2x). The vial was capped and sparged with N_2 for 10 minutes. The vial was sealed with parafilm and electrical tape and then sonicated for 1 minute. The reaction was irradiated with 450 nm light for 4 hours (m2 450 nm LED plate, 100% light intensity, 6800 rpm fan speed, 1000 rpm stirring). After irradiation, the vial was removed from the photoreactor and Selectfluor I (2 equiv, 1 mmol, 354.3 mg) in 1.5 mL dimethylformamide added against a flow of N_2. The resulting mixture was stirred at room temperature for 16 hours. Then, the mixture was

diluted with 20 mL of diethyl ether and stirred for an additional 10 minutes. The resulting suspension was filtered through a pad of celite, washed with water (3 x 25 mL), and dried over Na_2SO_4. After concentration onto silica, the residue was purified via automated flash chromatography (25 g high performance silica column, 0-25% ethyl acetate/hexanes). Fractions were directly concentrated and the product dissolved in dichloromethane and filtered through a cotton plug into a 40 mL vial. The solvent was removed and the product dried on high-vac to yield the title compound as a yellow oil (86.8 mg, 0.264 mmol, 53% yield).

^{1}H NMR (500 MHz, CDCl$_3$) δ 7.40 – 7.29 (m, 5H), 5.13 (s, 2H), 3.80 – 3.62 (m, 2H), 3.40 – 3.31 (m, 4H), 1.71 – 1.55 (m, 4H), 1.33 (s, 3H).

^{13}C NMR (126 MHz, CDCl$_3$) δ 155.30, 136.72, 128.68, 128.26, 128.10, 67.42, 61.48 (d, *J* = 11.4 Hz), 39.61, 36.17, 32.74, 22.69.

^{19}F NMR (376 MHz, CDCl$_3$) δ 67.25.

IR (film) v_{max} 2964, 2878, 1721, 1466, 1369, 1300, 1255, 750 cm^{-1}.

HRMS (ESI-TOF) *m/z* calculated for $C_{15}H_{21}FNO_4S^+$ ($[M+H]^+$) 330.1170, found 330.1167.

10. NMR Spectra

S1, ^{1}H NMR, 500 MHz, $CDCl_3$

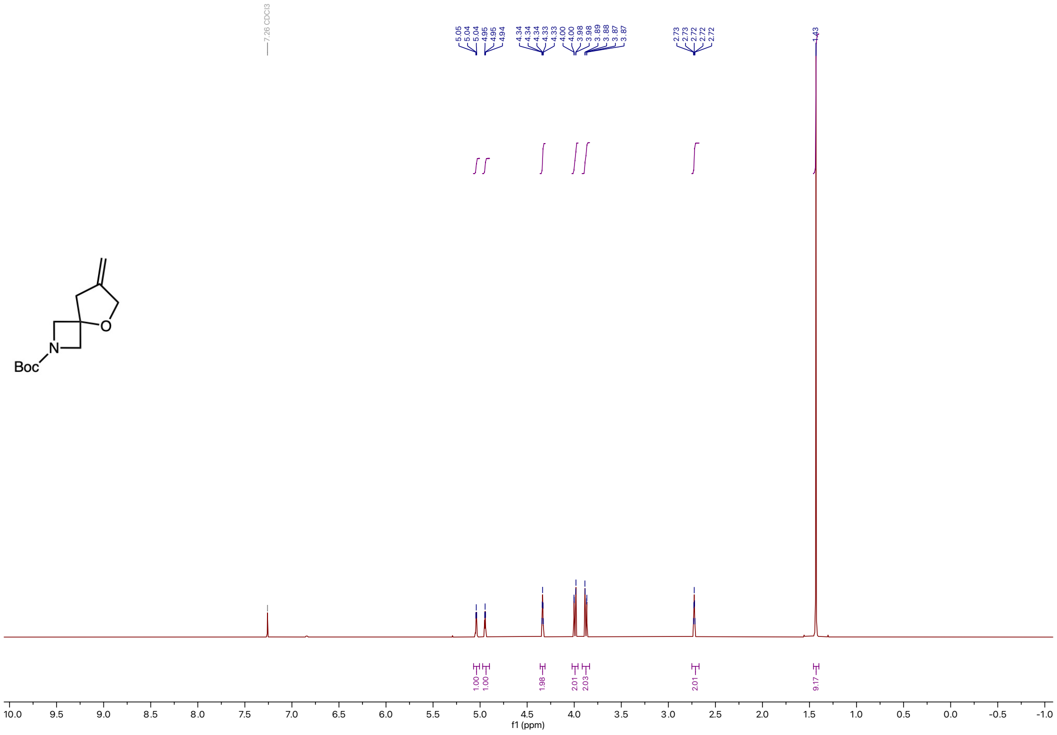

S1, ^{13}C NMR, 126 MHz, $CDCl_3$

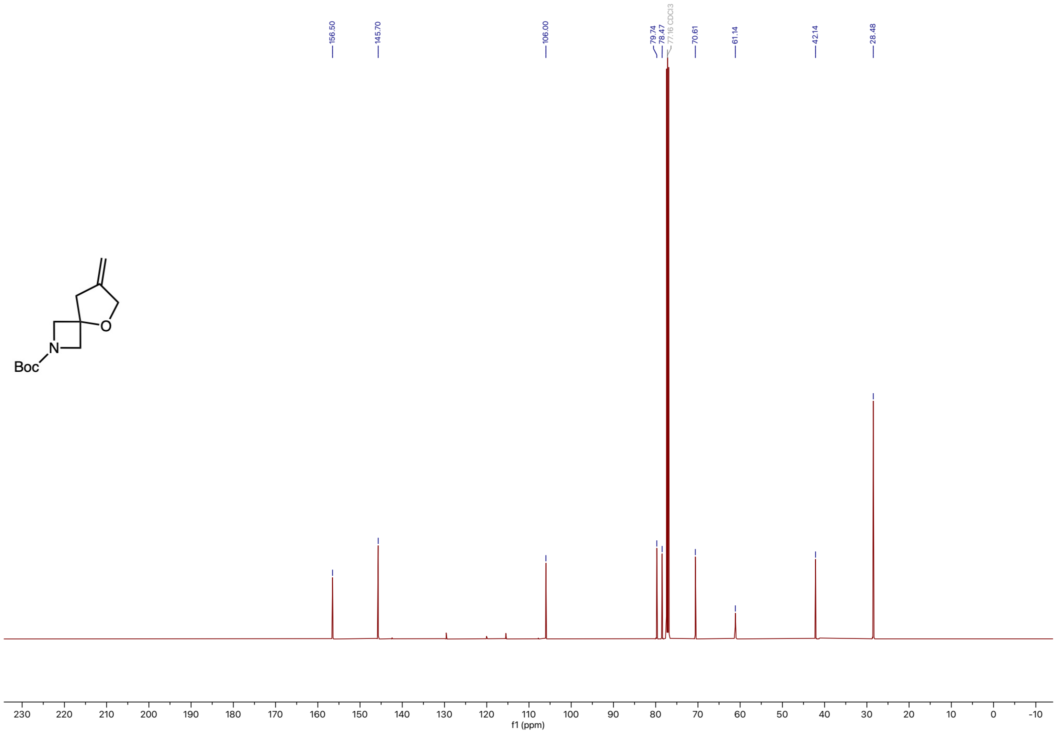

S2, ^{1}H NMR, 500 MHz, $CDCl_3$

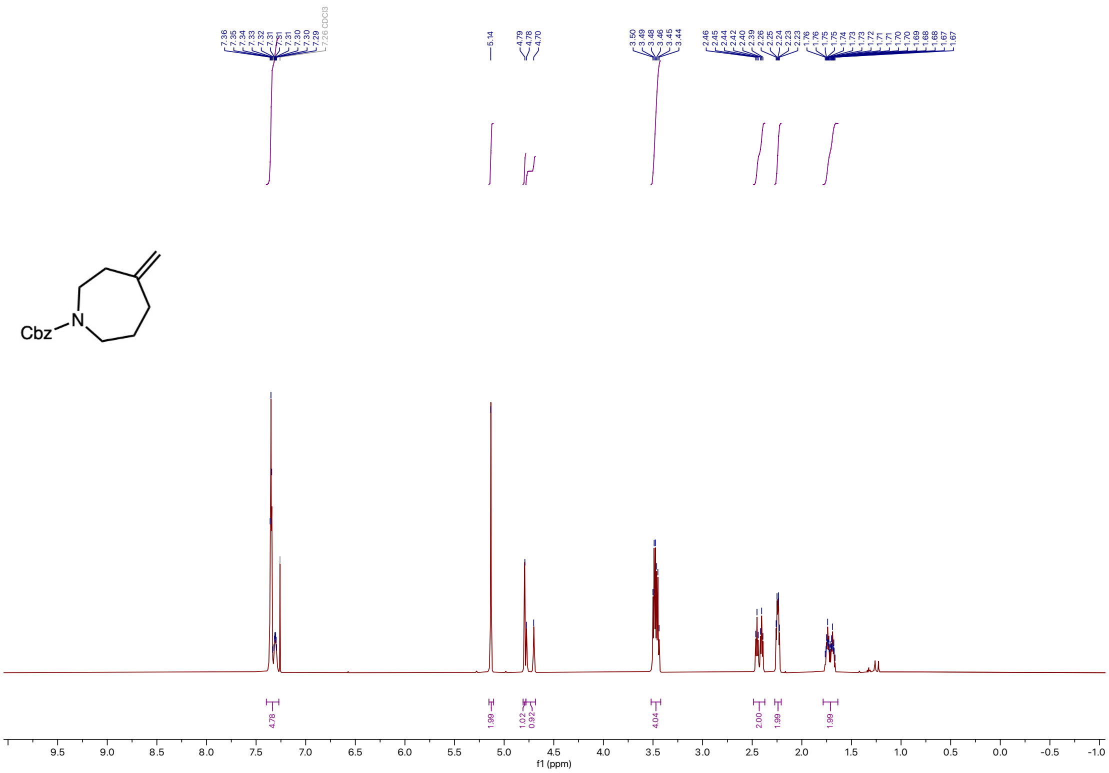

S2, ^{13}C NMR, 126 MHz, $CDCl_3$

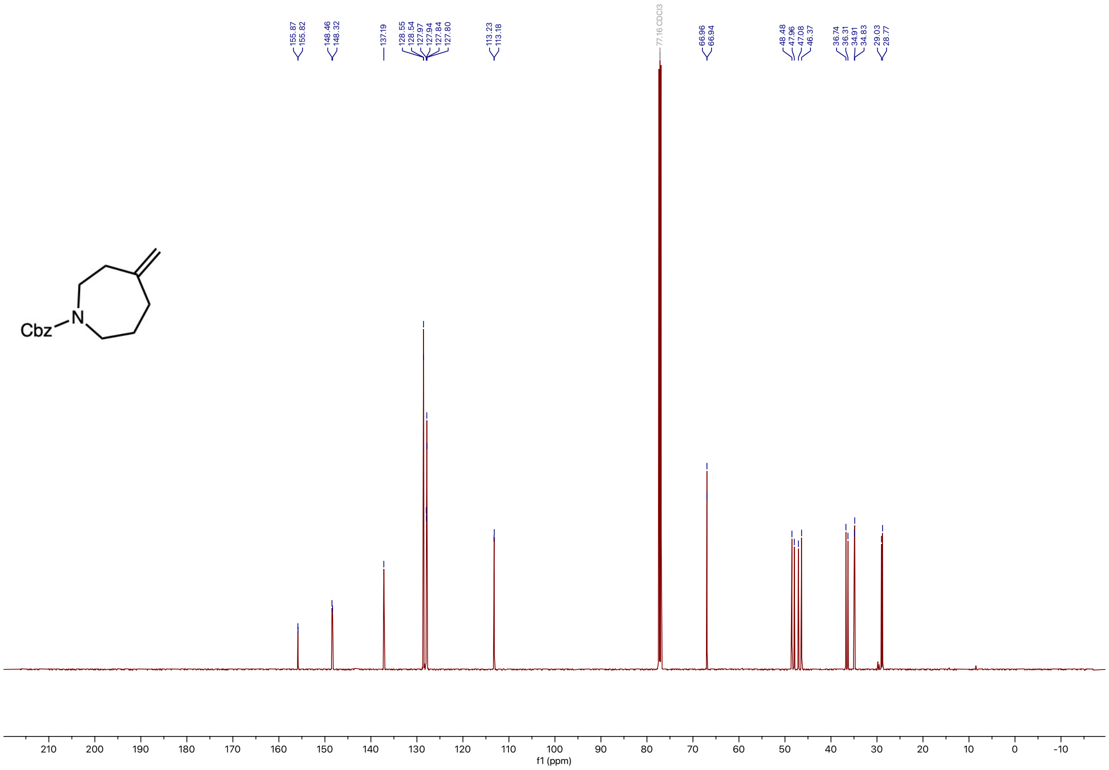

S3, ^{1}H NMR, 500 MHz, $CDCl_3$

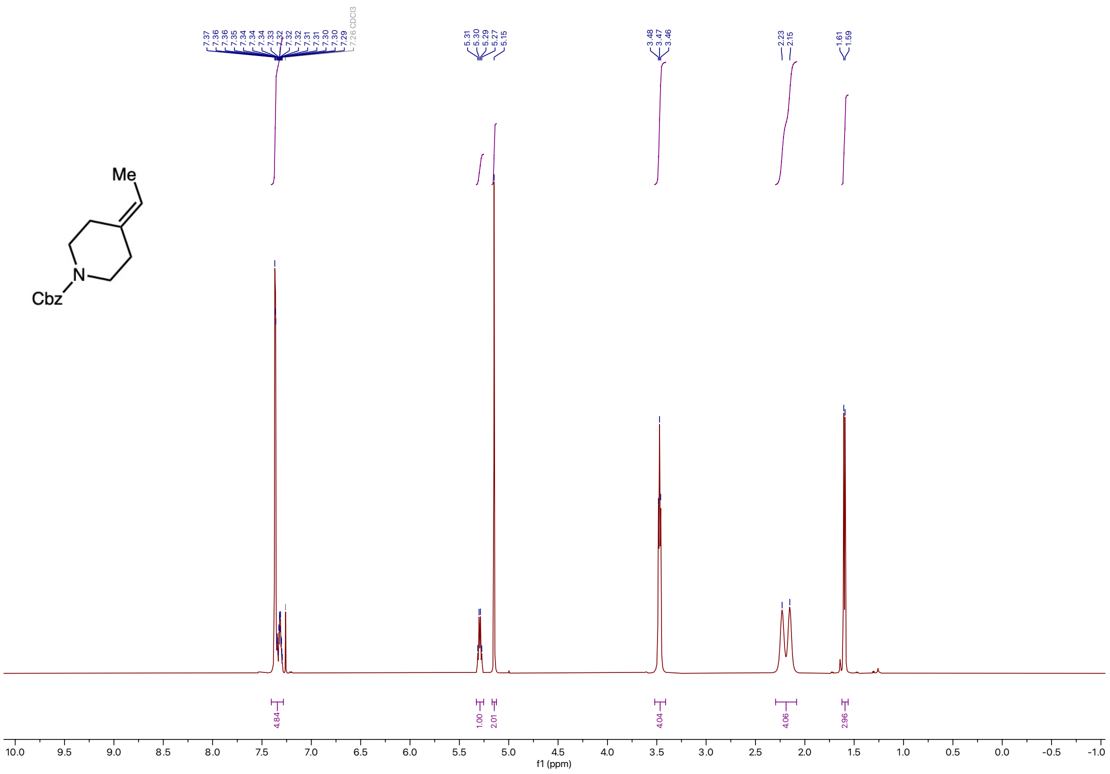

S3, ^{13}C NMR, 126 MHz, $CDCl_3$

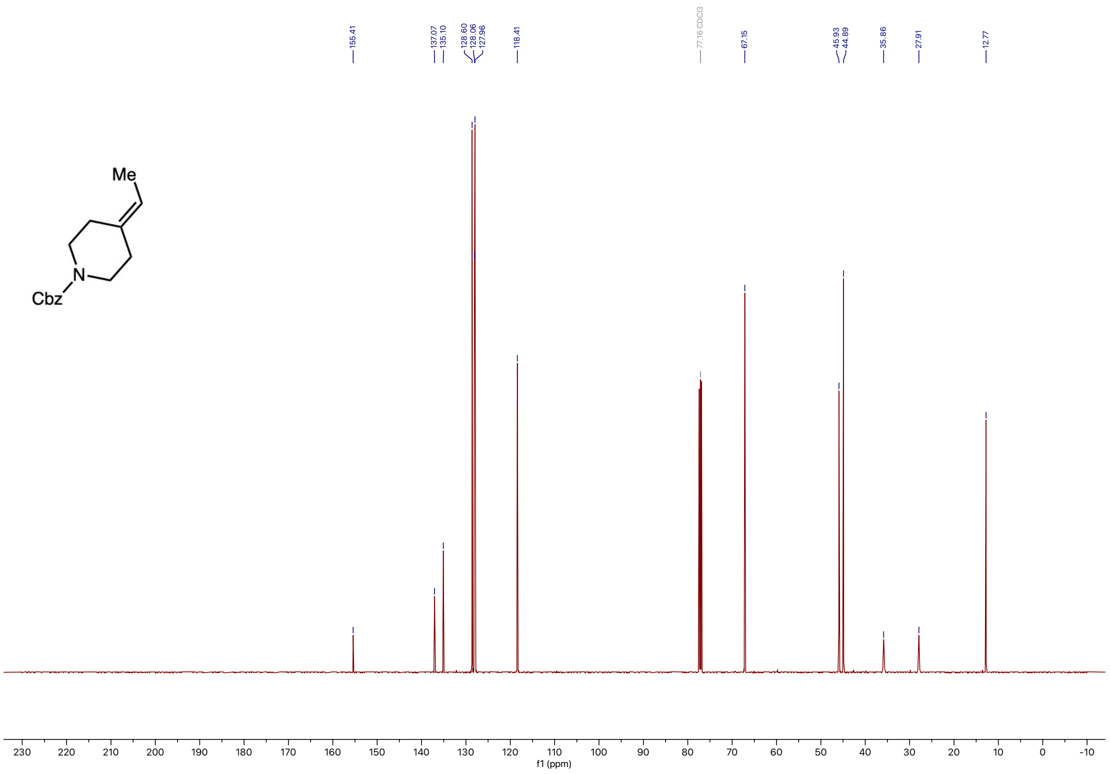

S4, ^{1}H NMR, 500 MHz, $CDCl_3$

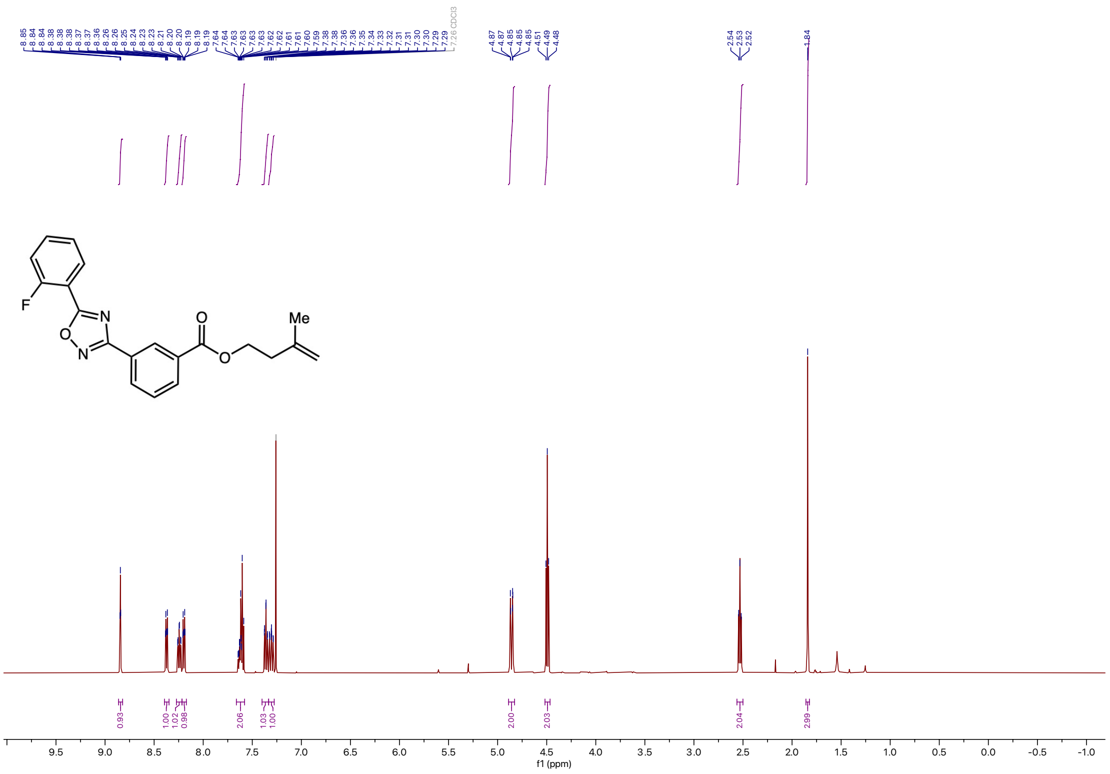

S4, ^{13}C NMR, 126 MHz, $CDCl_3$

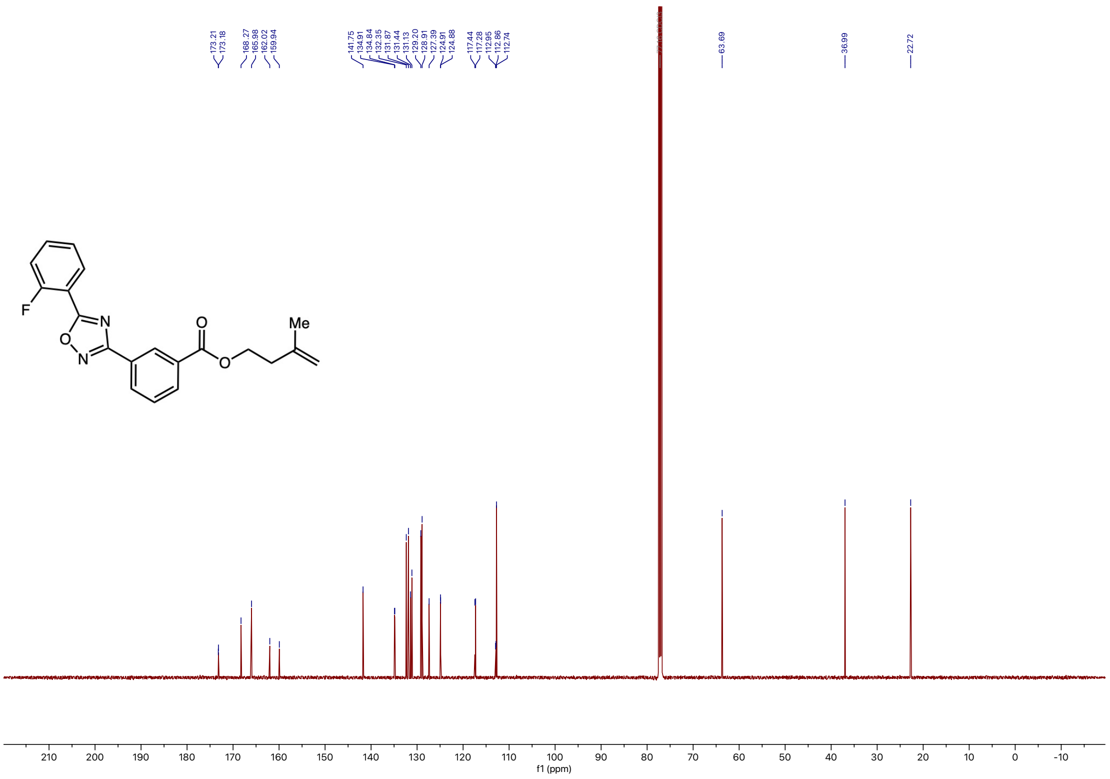

S4, ^{19}F NMR, 376 MHz, $CDCl_3$

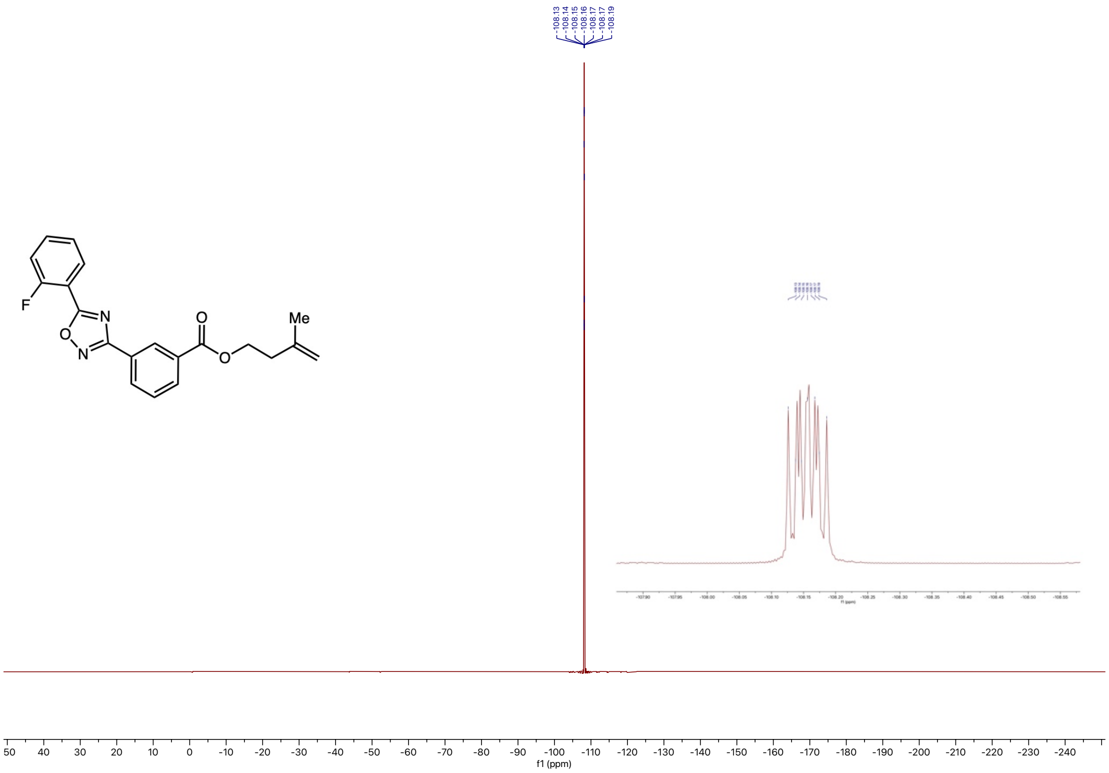

S5, ^{1}H NMR, 500 MHz, $CDCl_3$

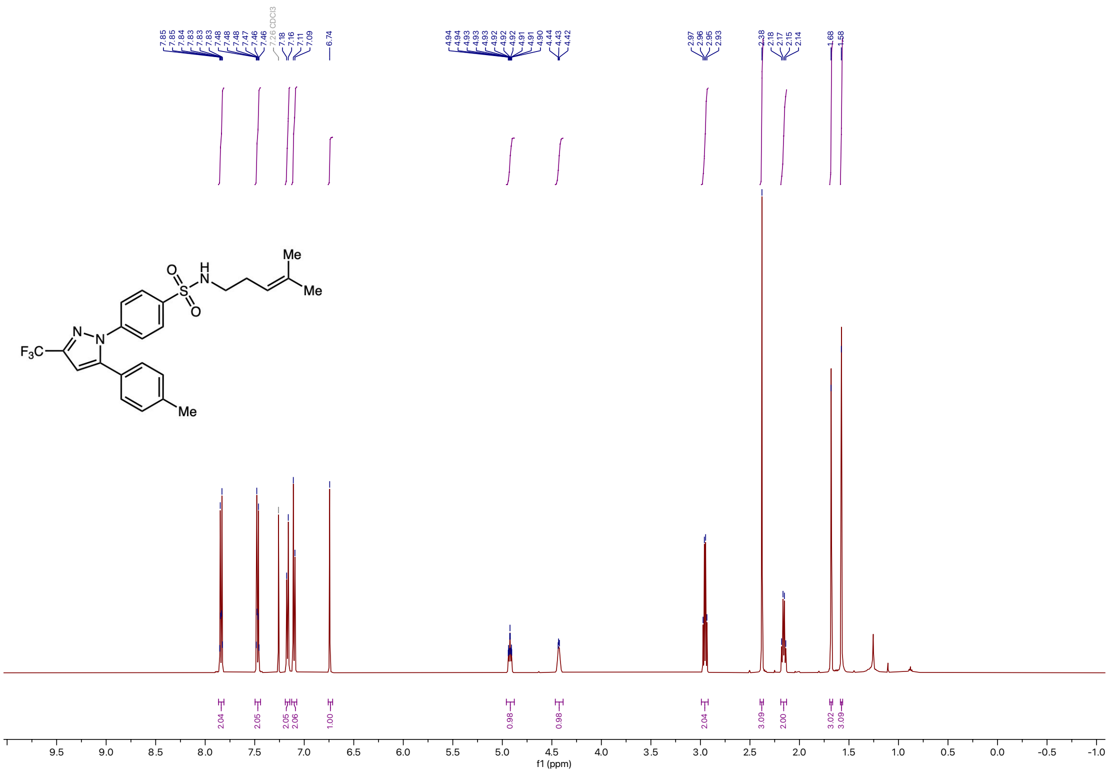

S5, ^{13}C NMR, 126 MHz, $CDCl_3$

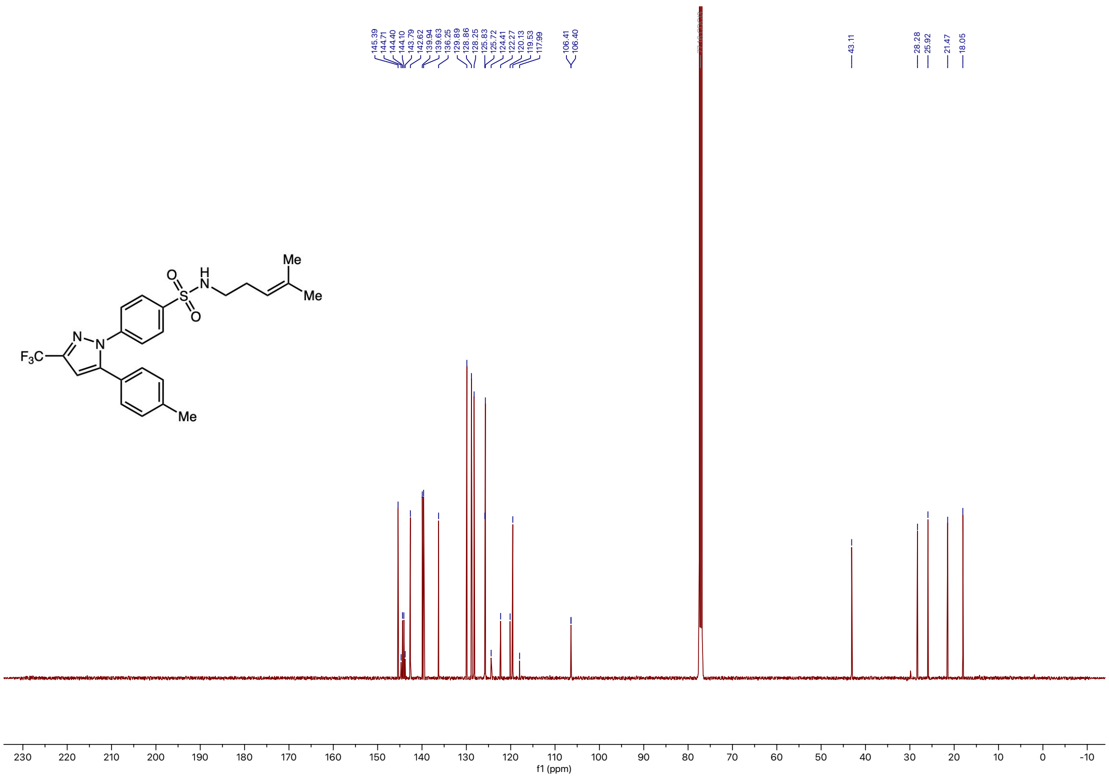

S5, ^{19}F NMR, 376 MHz, $CDCl_3$

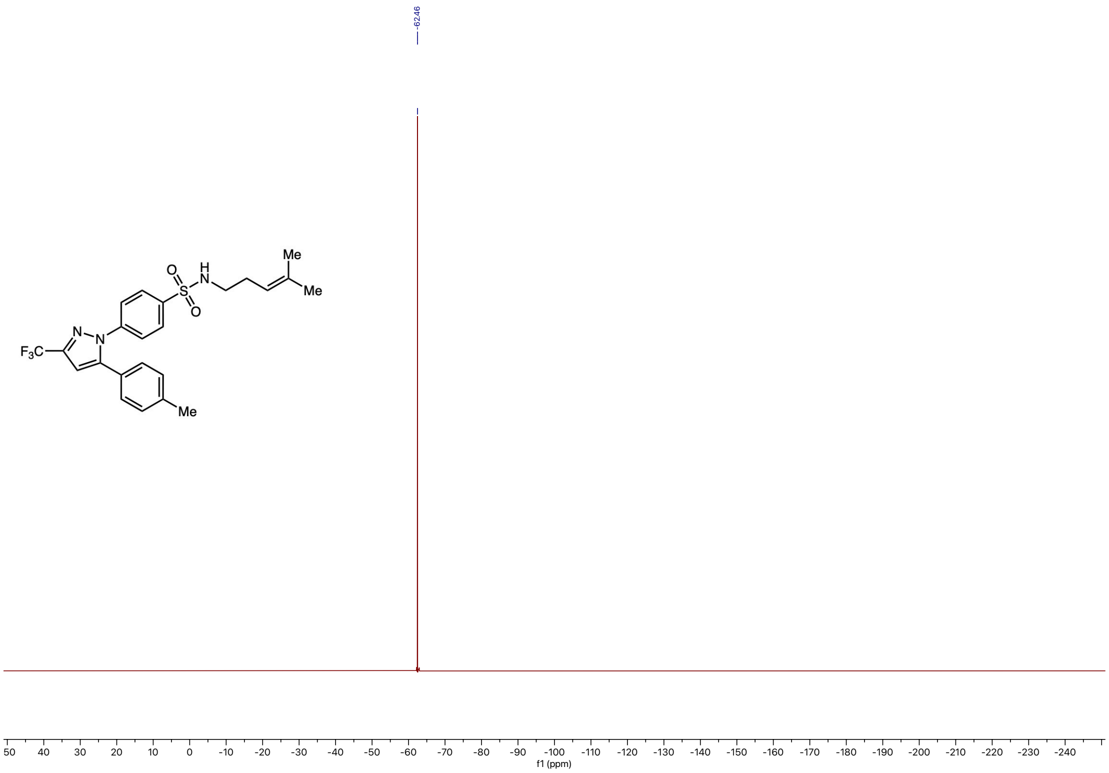

S6, ^{1}H NMR, 500 MHz, $CDCl_3$

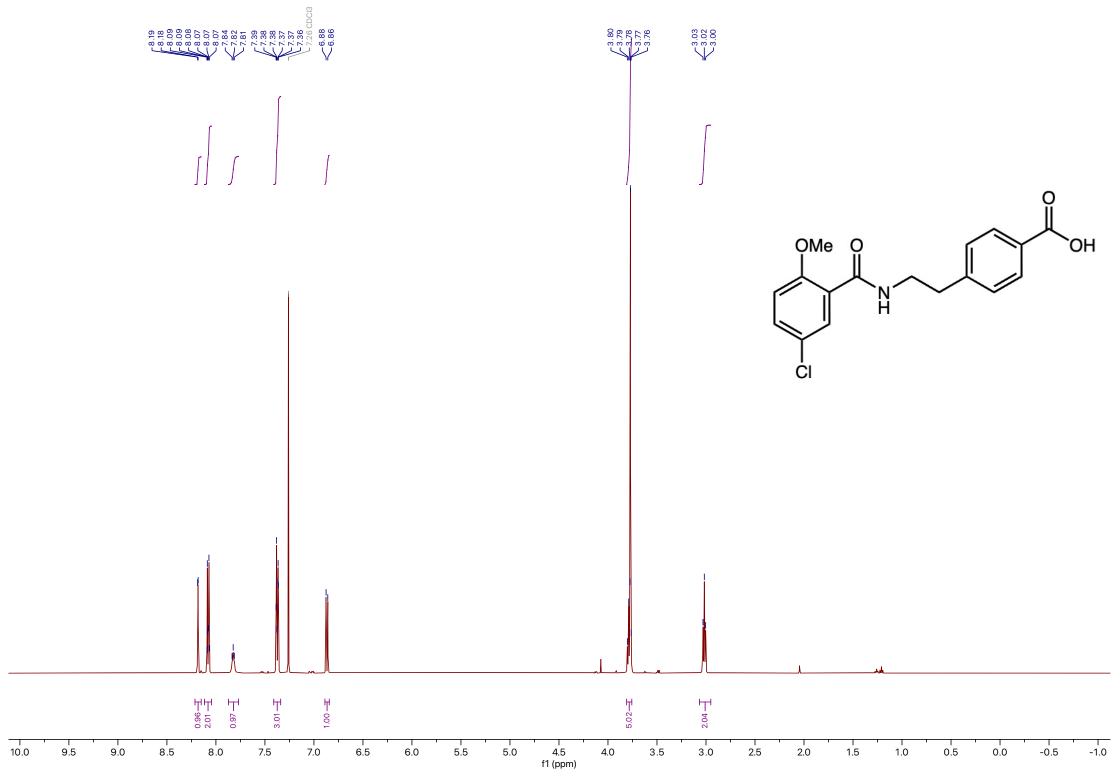

S6, ^{13}C NMR, 126 MHz, $CDCl_3$

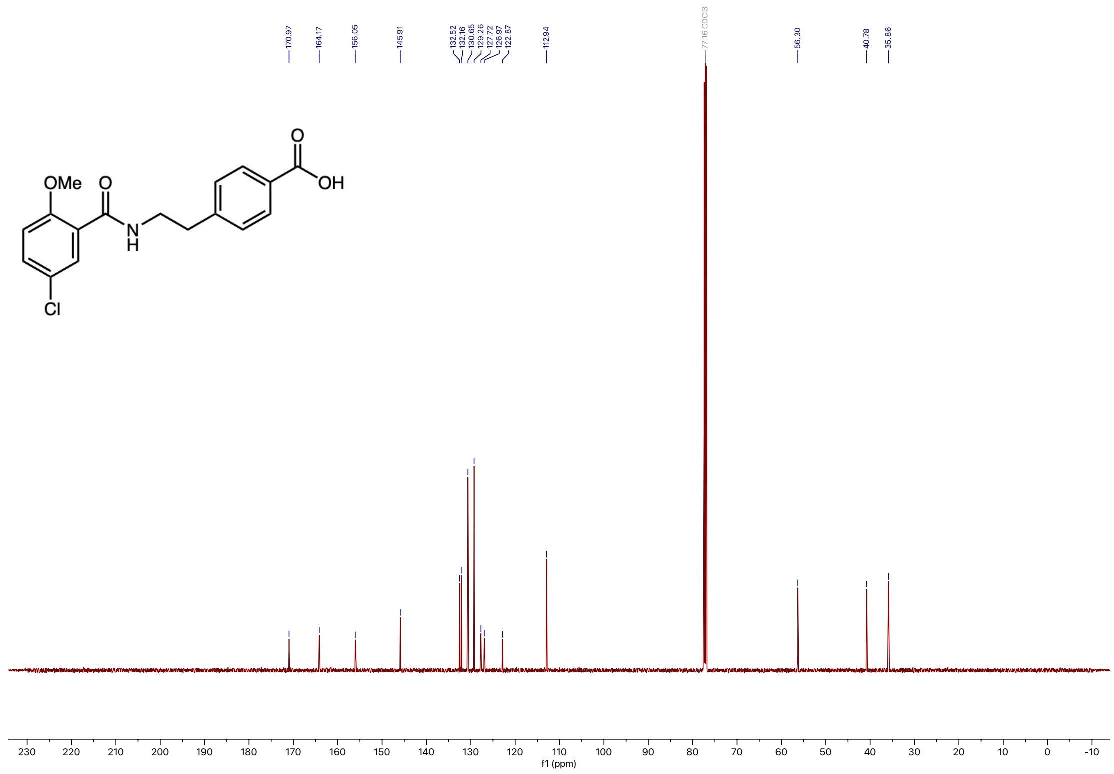

S7, ^{1}H NMR, 500 MHz, $CDCl_3$

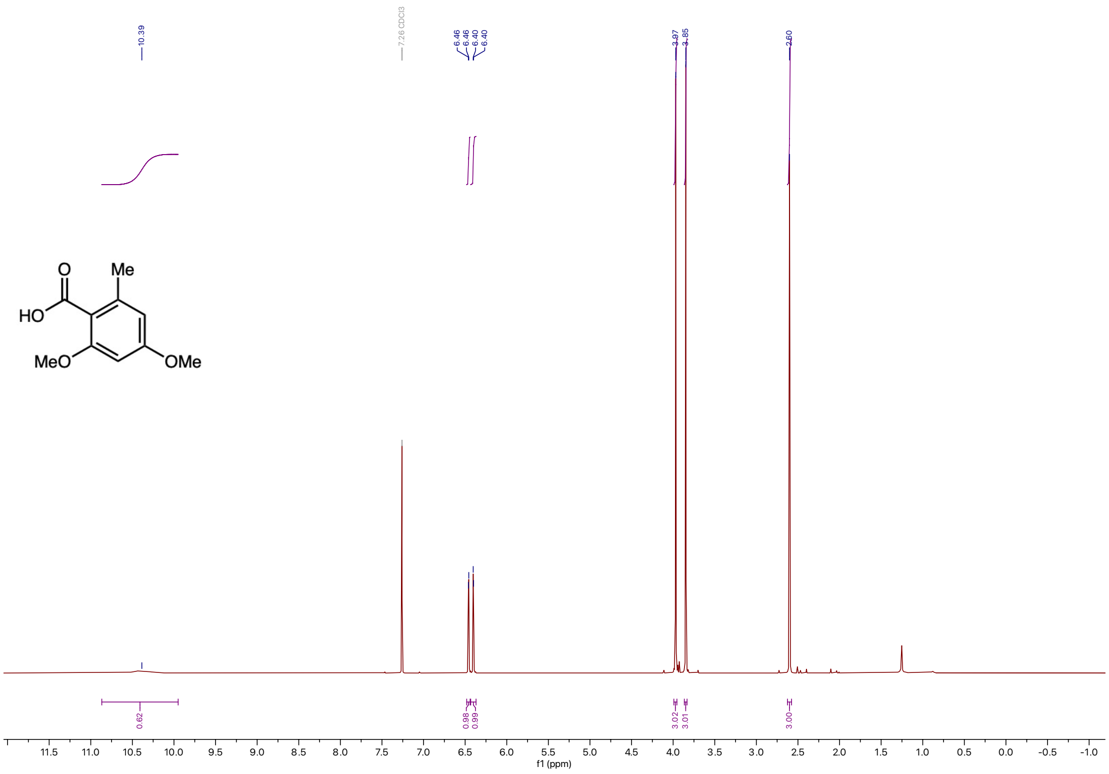

S7, ^{13}C NMR, 126 MHz, $CDCl_3$

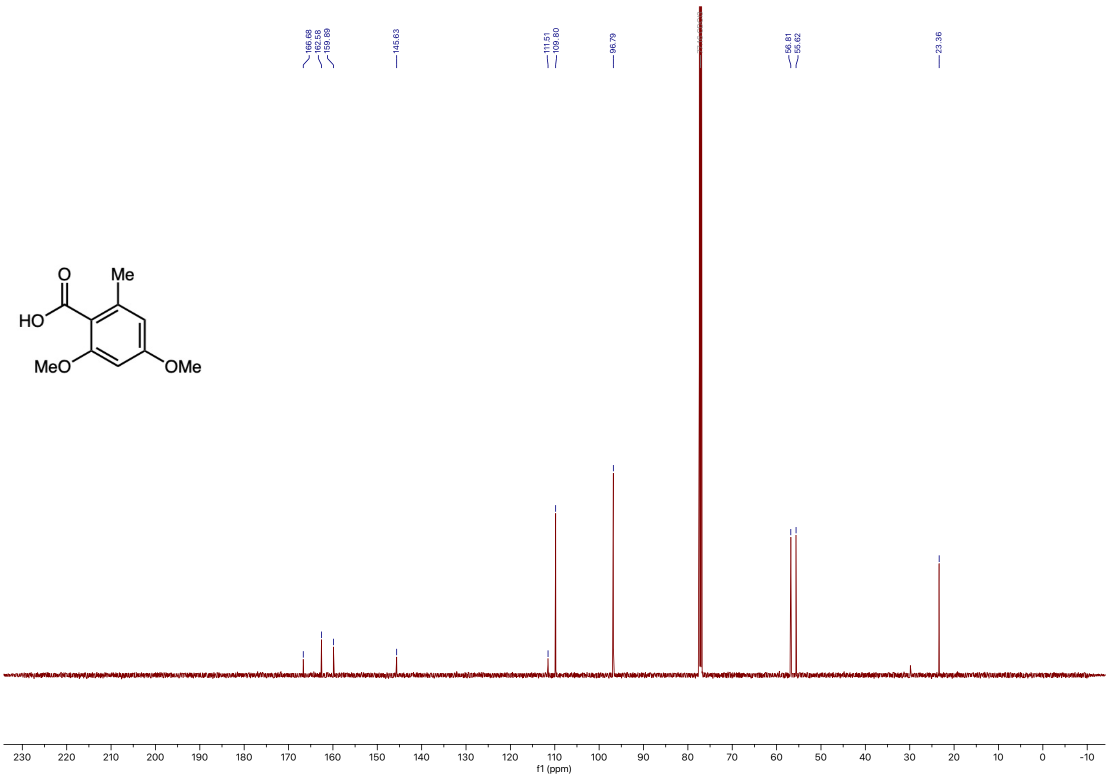

S8, ^{1}H NMR, 500 MHz, $CDCl_3$

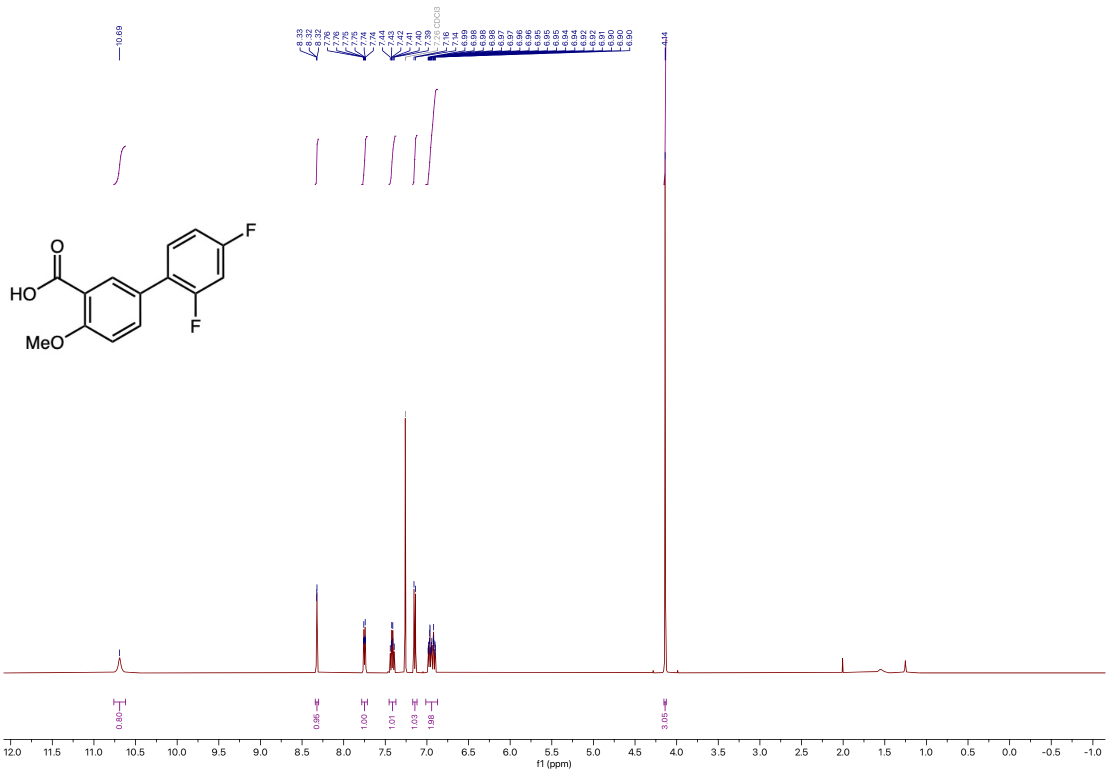

S8, ^{13}C NMR, 126 MHz, $CDCl_3$

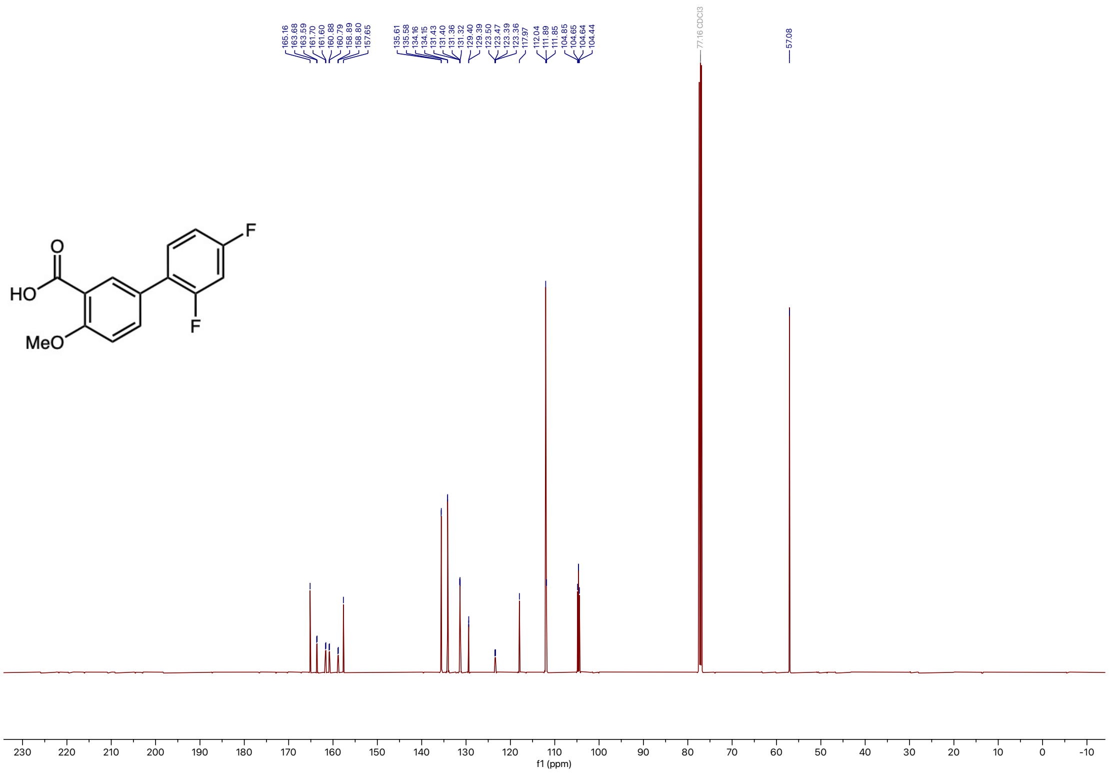

S8, ^{19}F NMR, 376 MHz, $CDCl_3$

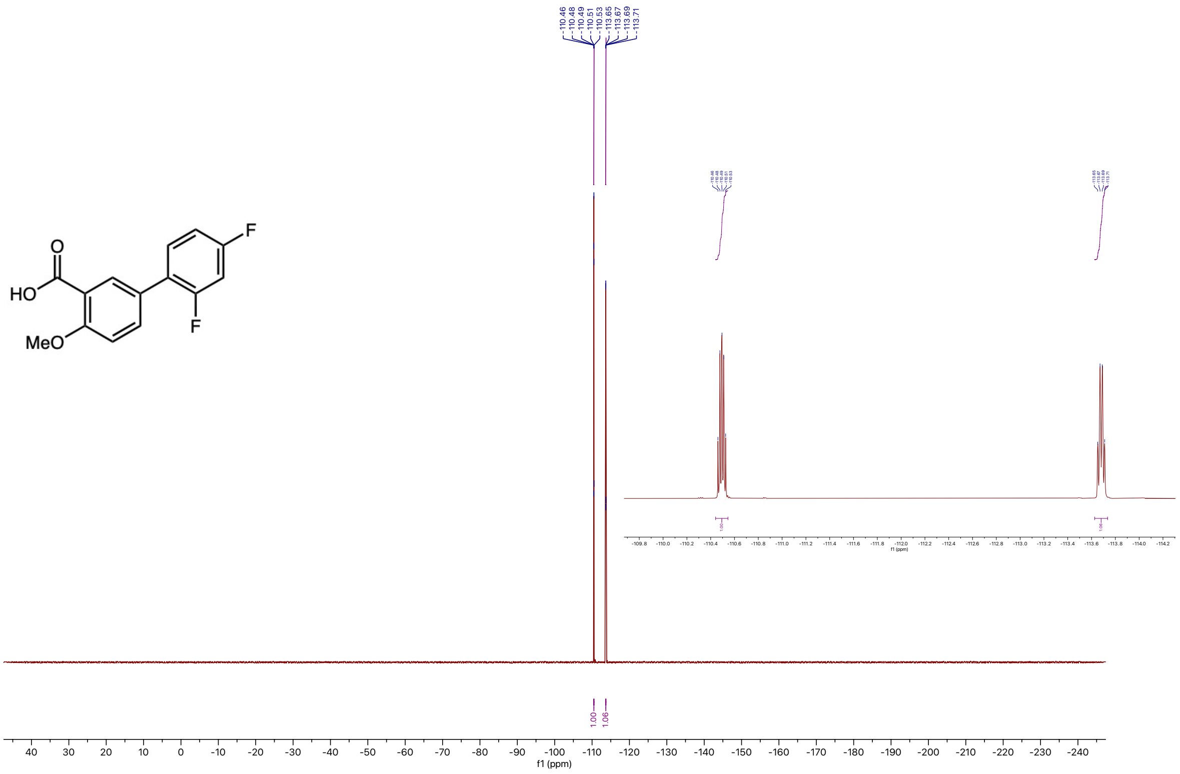

S9, ^{1}H NMR, 500 MHz, $CDCl_3$

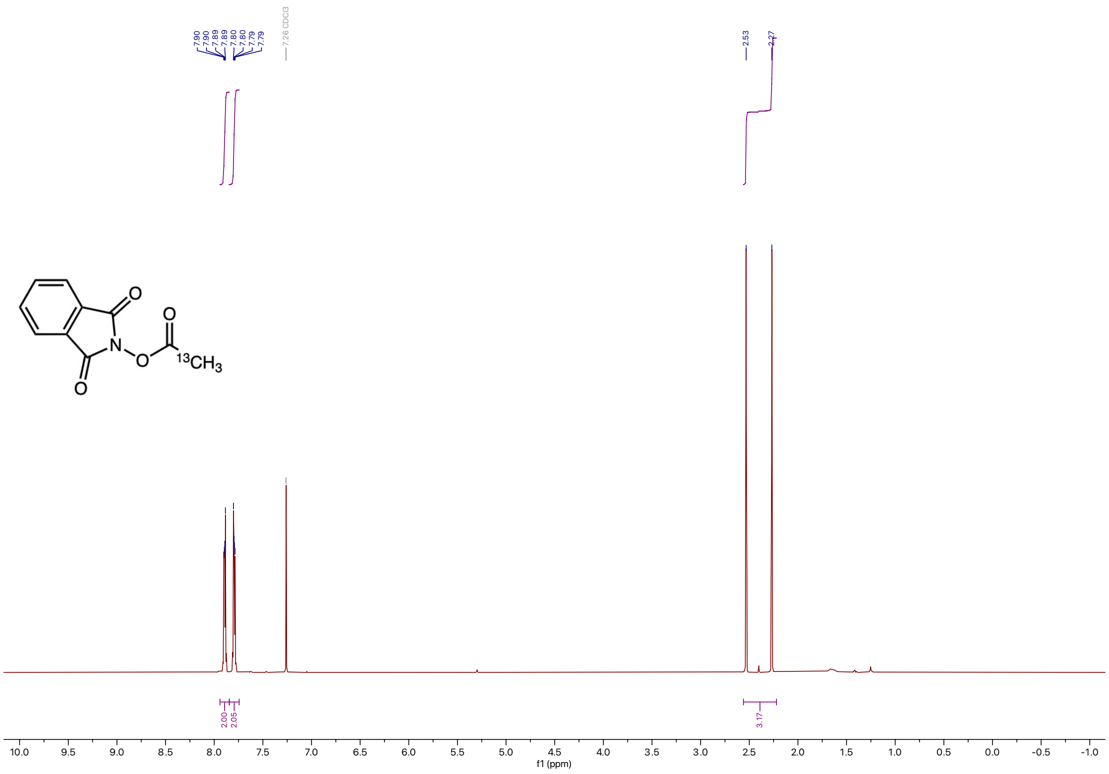

S9, ^{13}C NMR, 126 MHz, $CDCl_3$

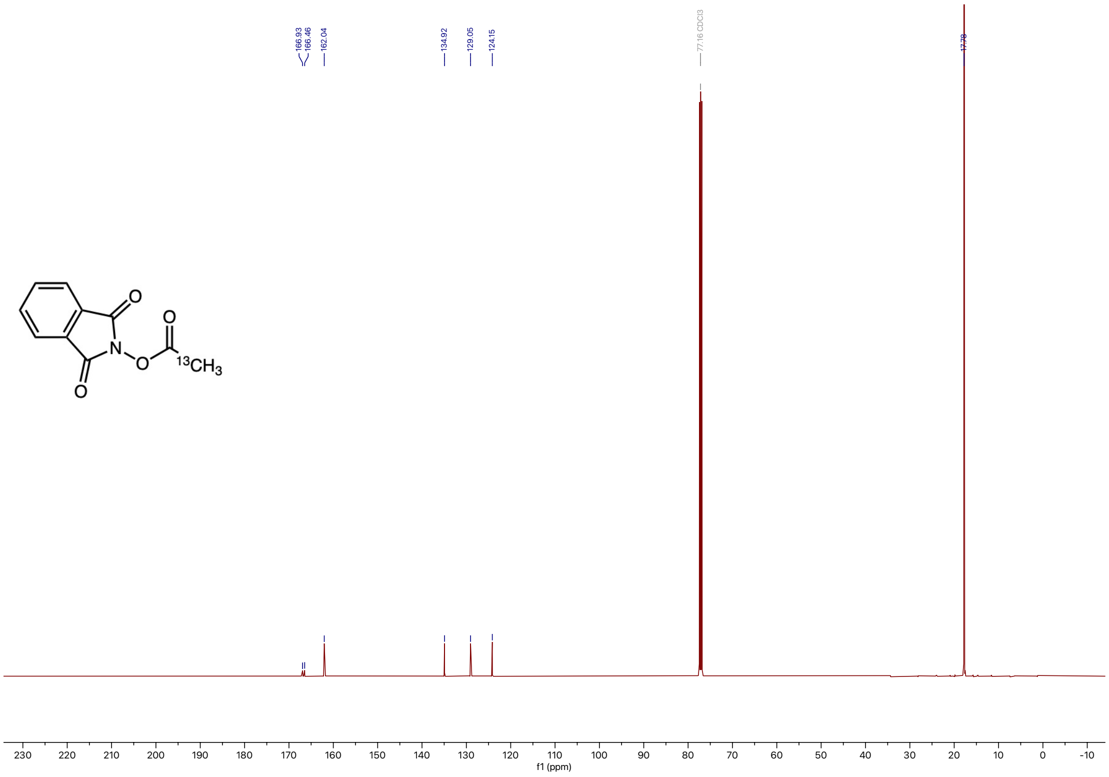

S10, ^{1}H NMR, 500 MHz, $CDCl_3$

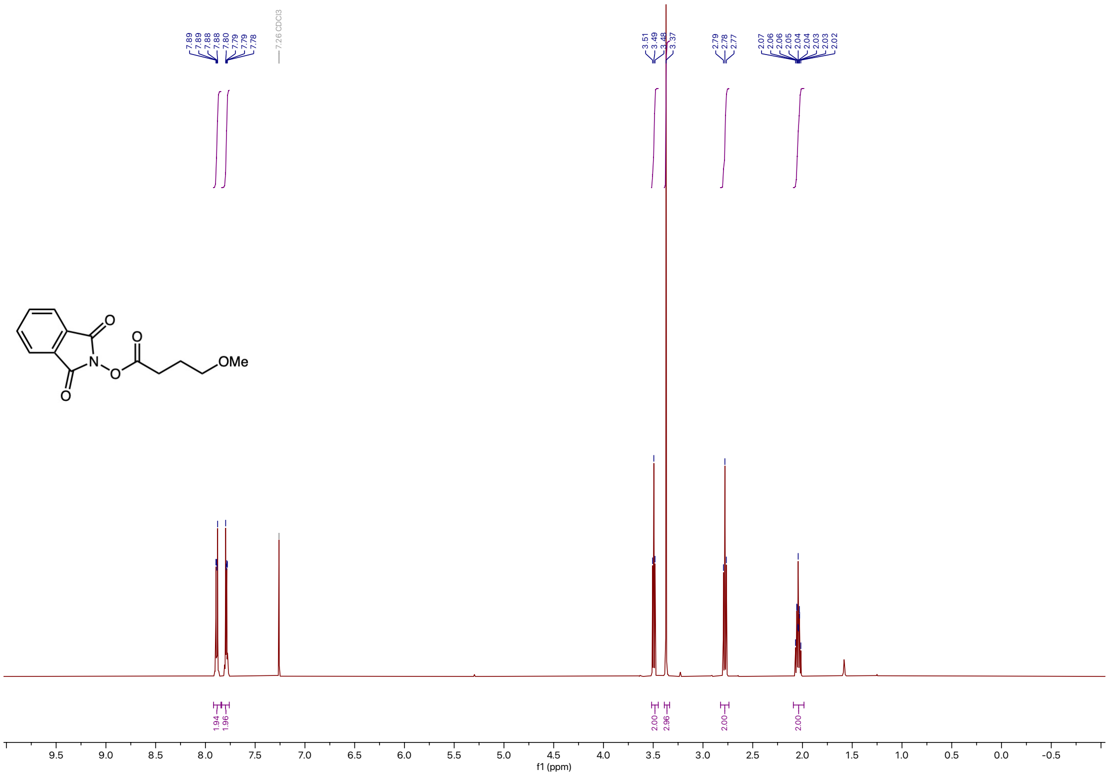

S10, ^{13}C NMR, 126 MHz, $CDCl_3$

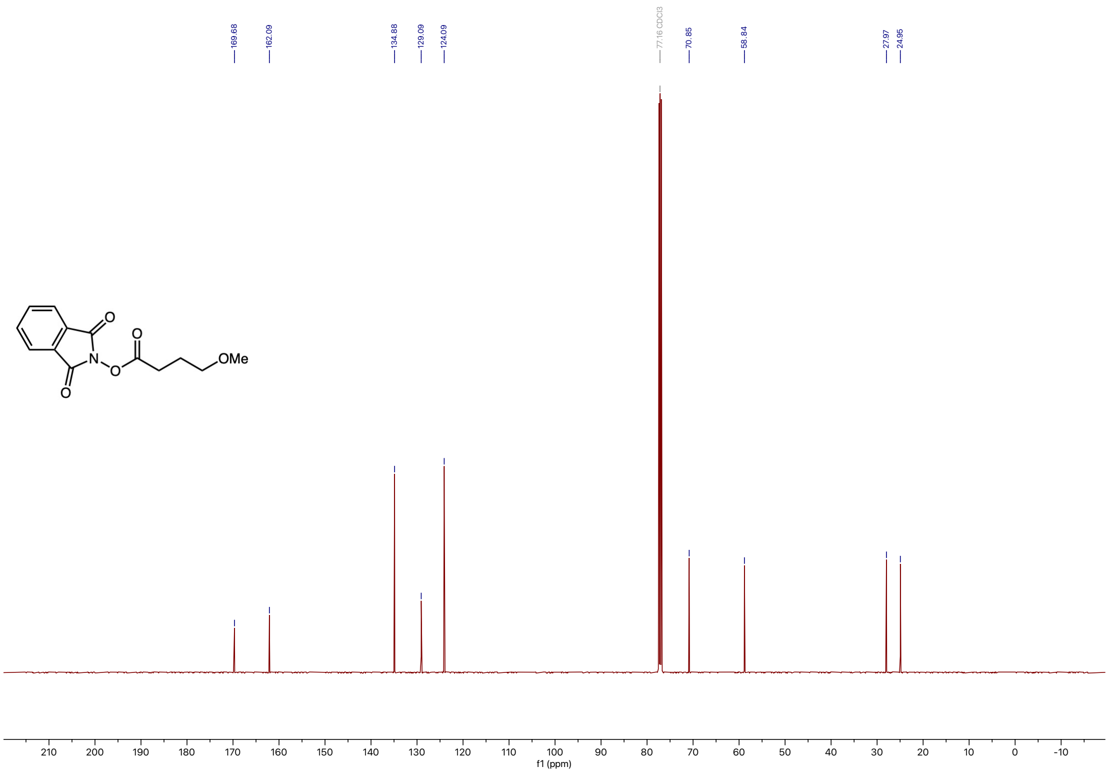

S11, ^{1}H NMR, 500 MHz, $CDCl_3$

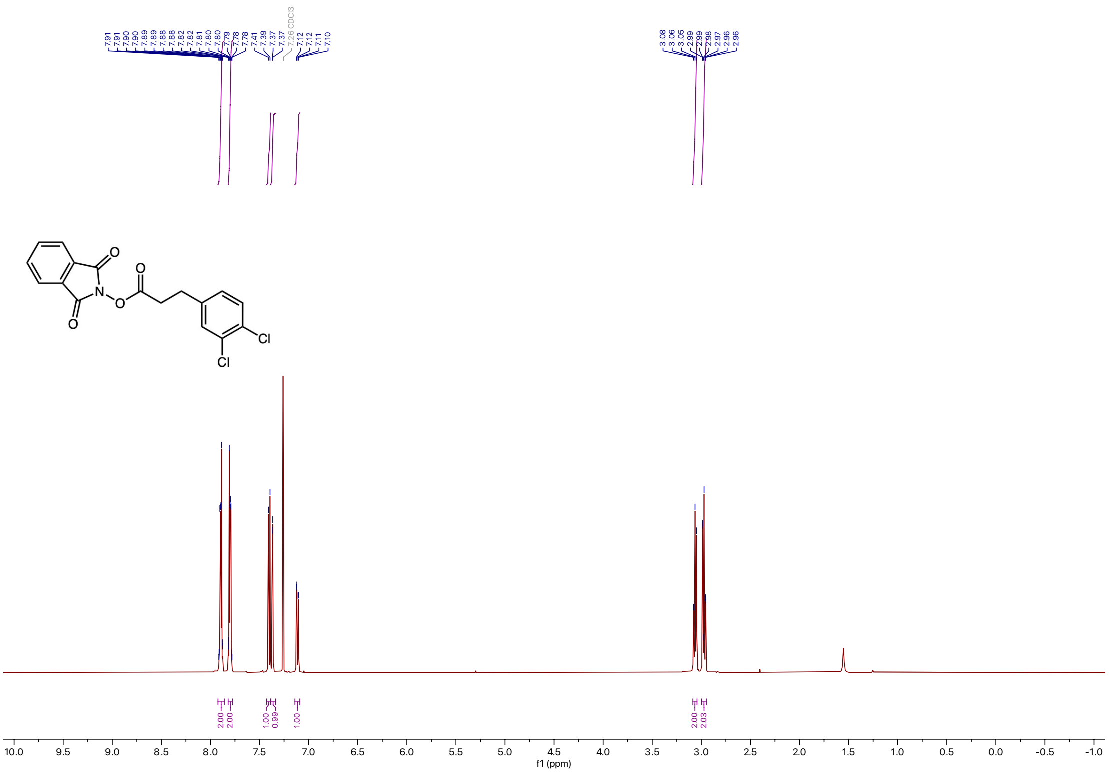

S11, ^{13}C NMR, 126 MHz, $CDCl_3$

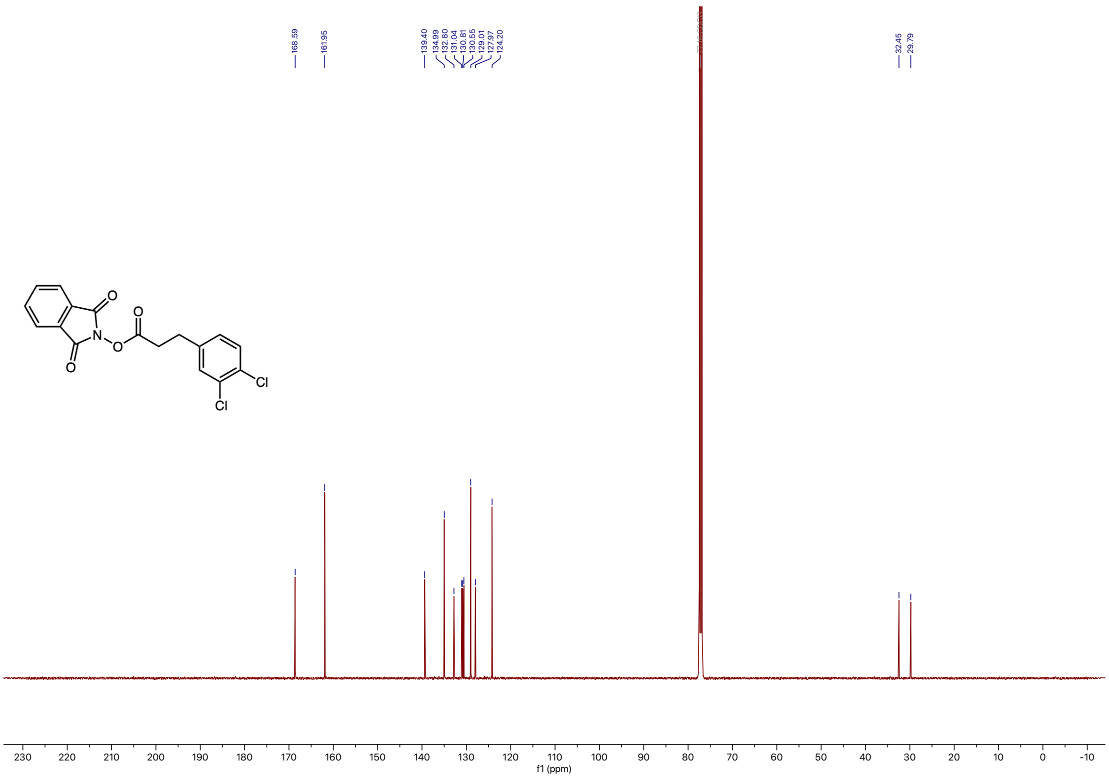

S39, ^{1}H NMR, 500 MHz, $CDCl_3$

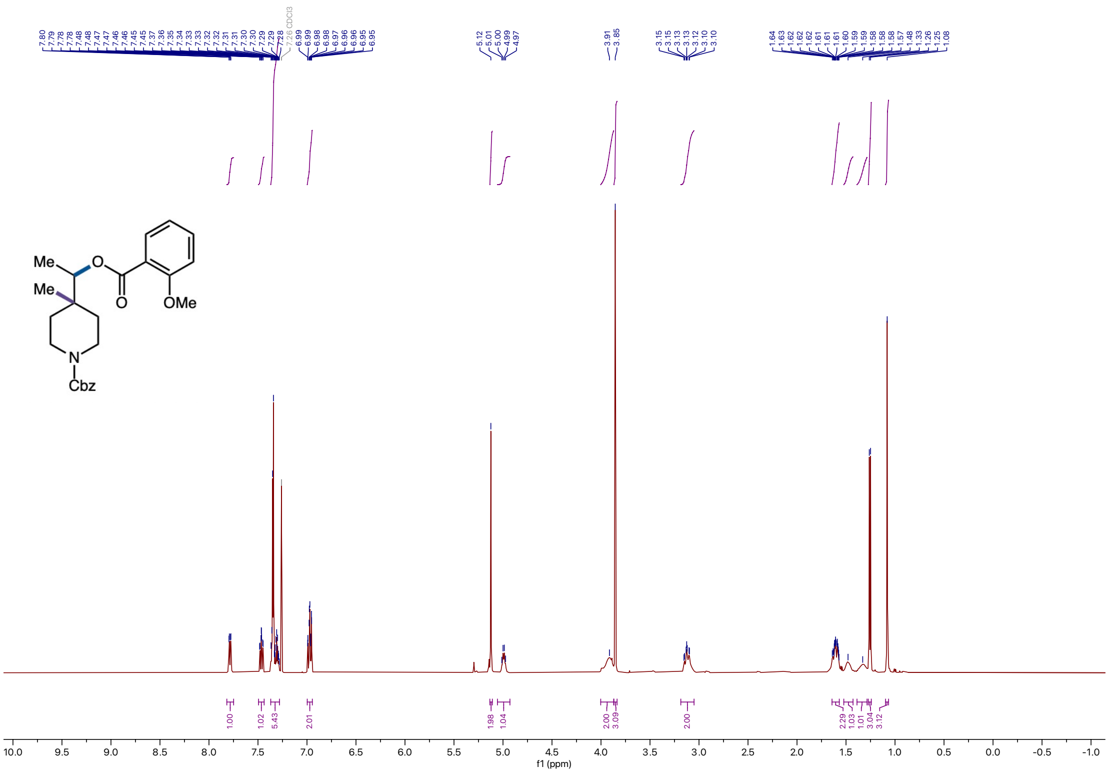

S39, ^{13}C NMR, 126 MHz, $CDCl_3$

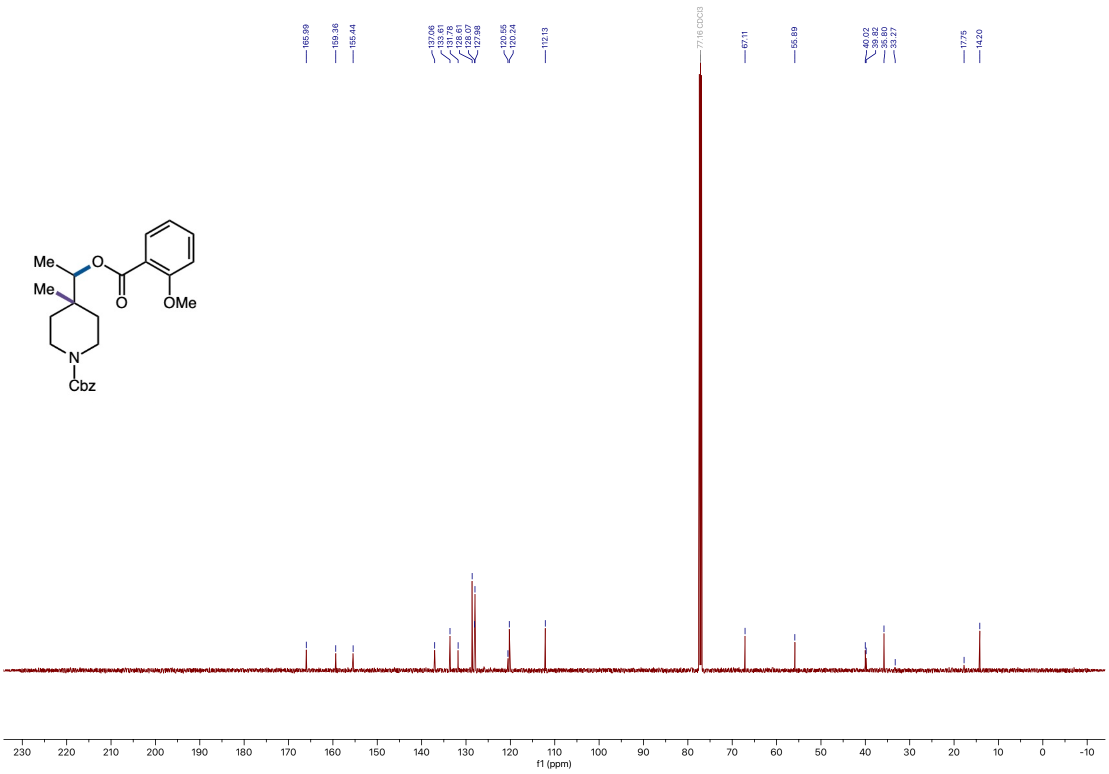

6, ^{1}H NMR, 500 MHz, $CDCl_3$

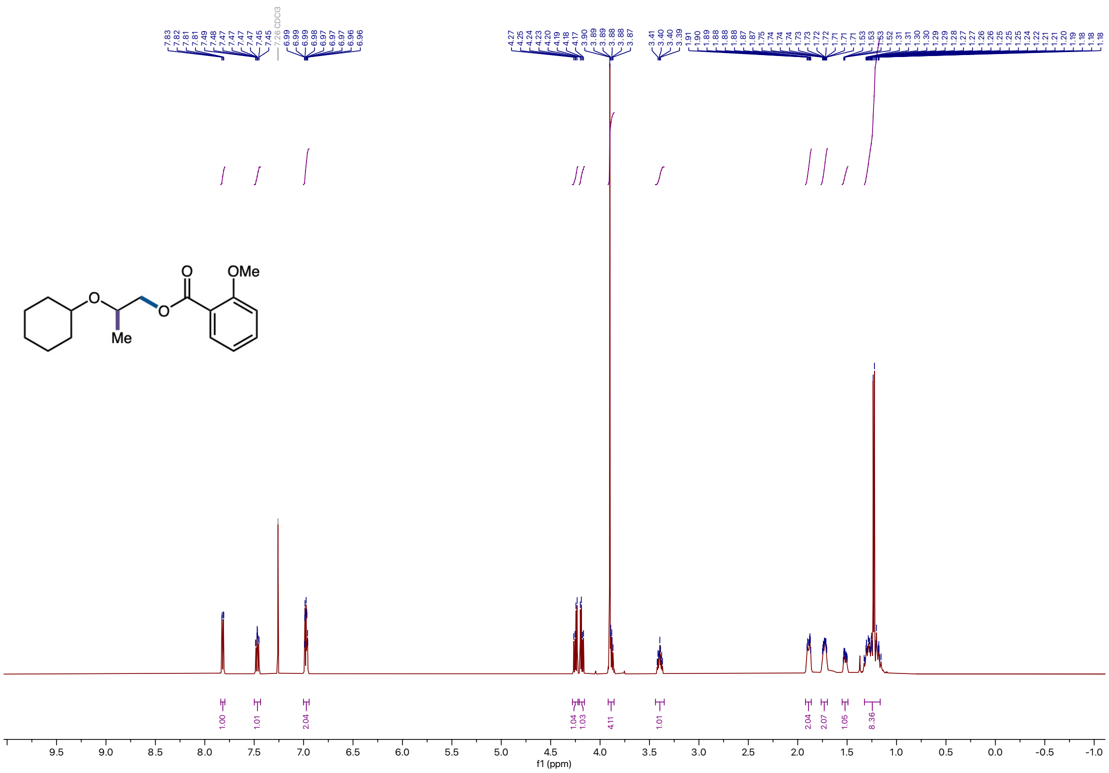

6, ^{13}C NMR, 126 MHz, $CDCl_3$

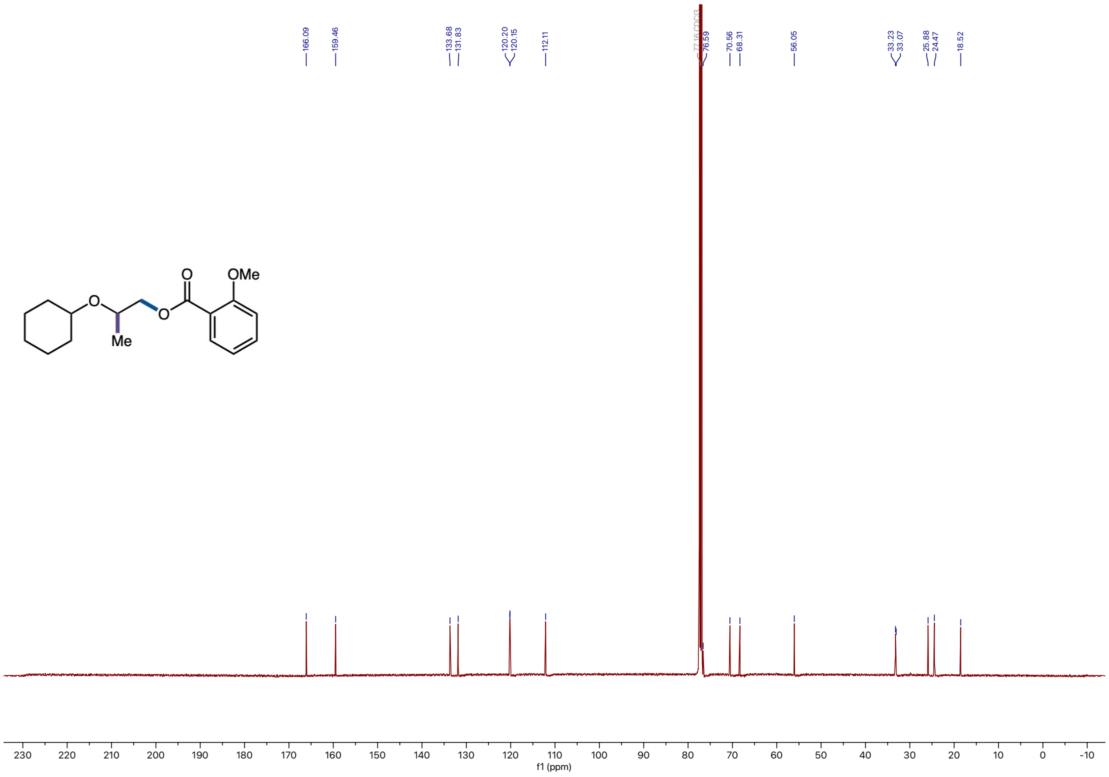

7, ^{1}H NMR, 500 MHz, $CDCl_3$

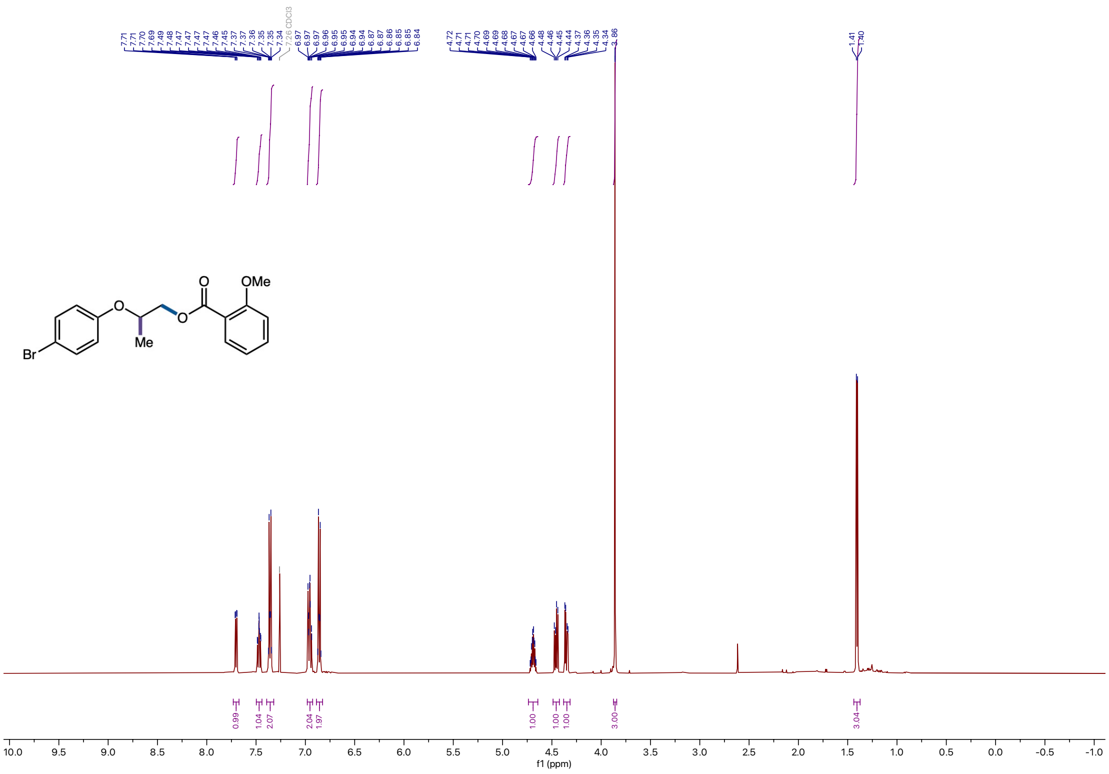

7, ^{13}C NMR, 126 MHz, $CDCl_3$

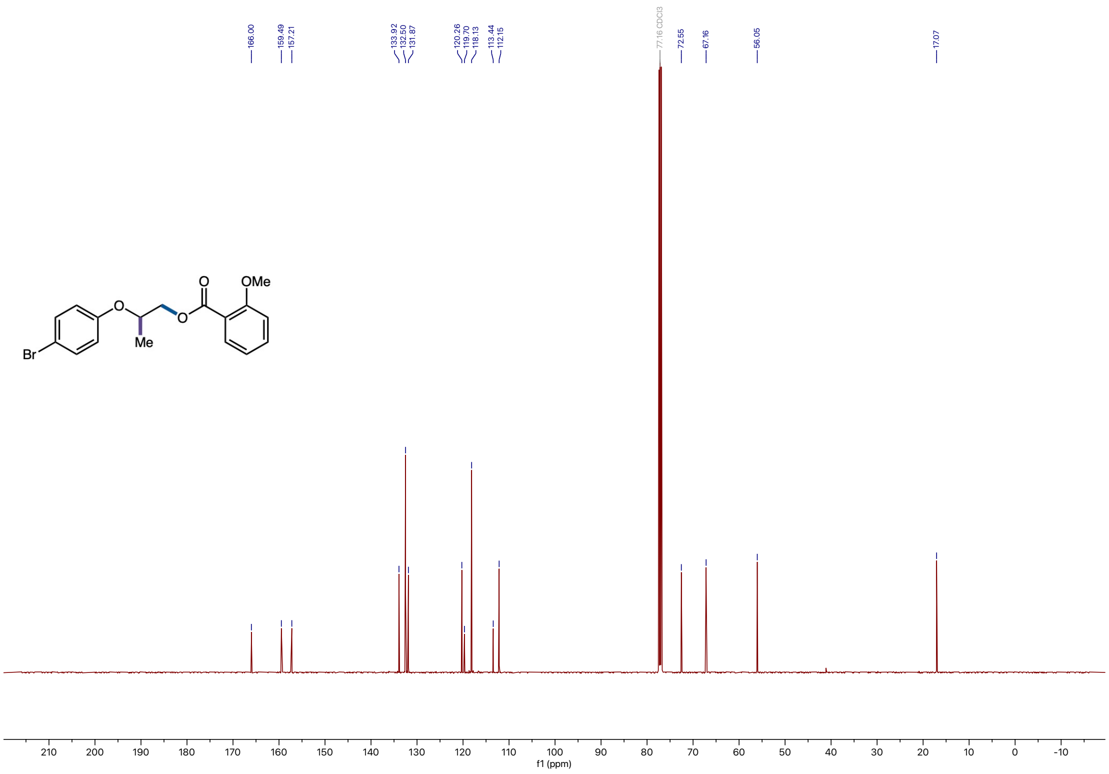

8, ^{1}H NMR, 500 MHz, $CDCl_3$

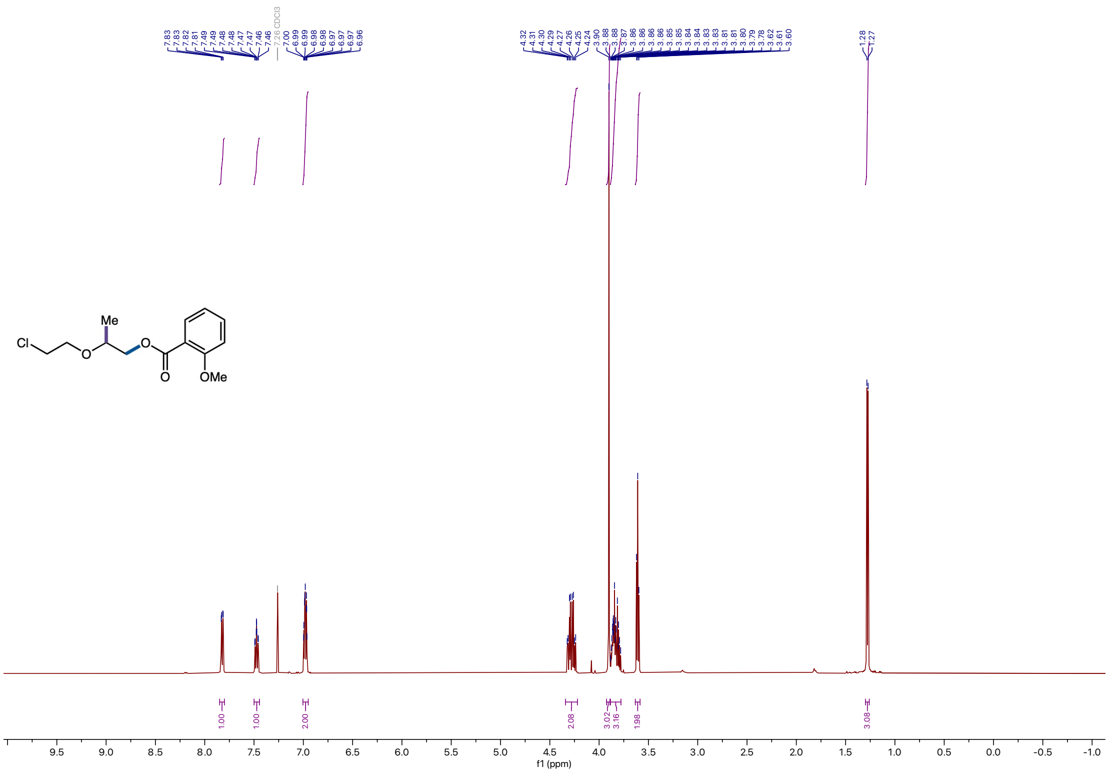

8, ^{13}C NMR, 126 MHz, $CDCl_3$

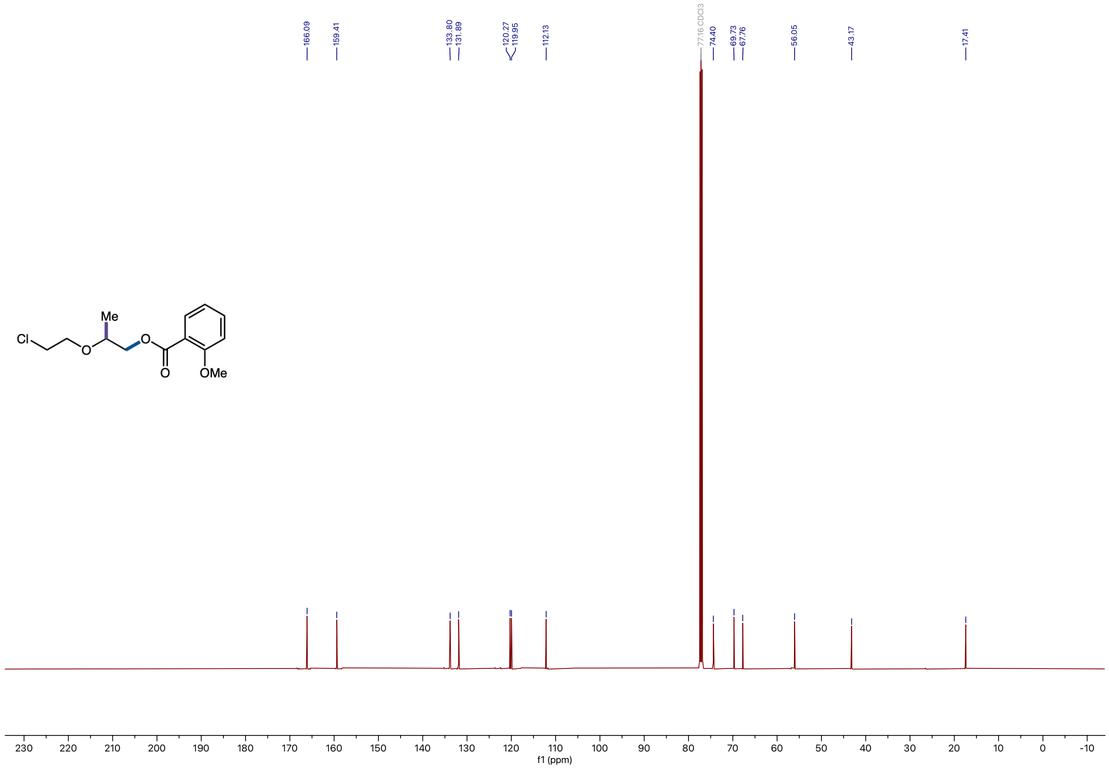

9, ^{1}H NMR, 500 MHz, $CDCl_3$

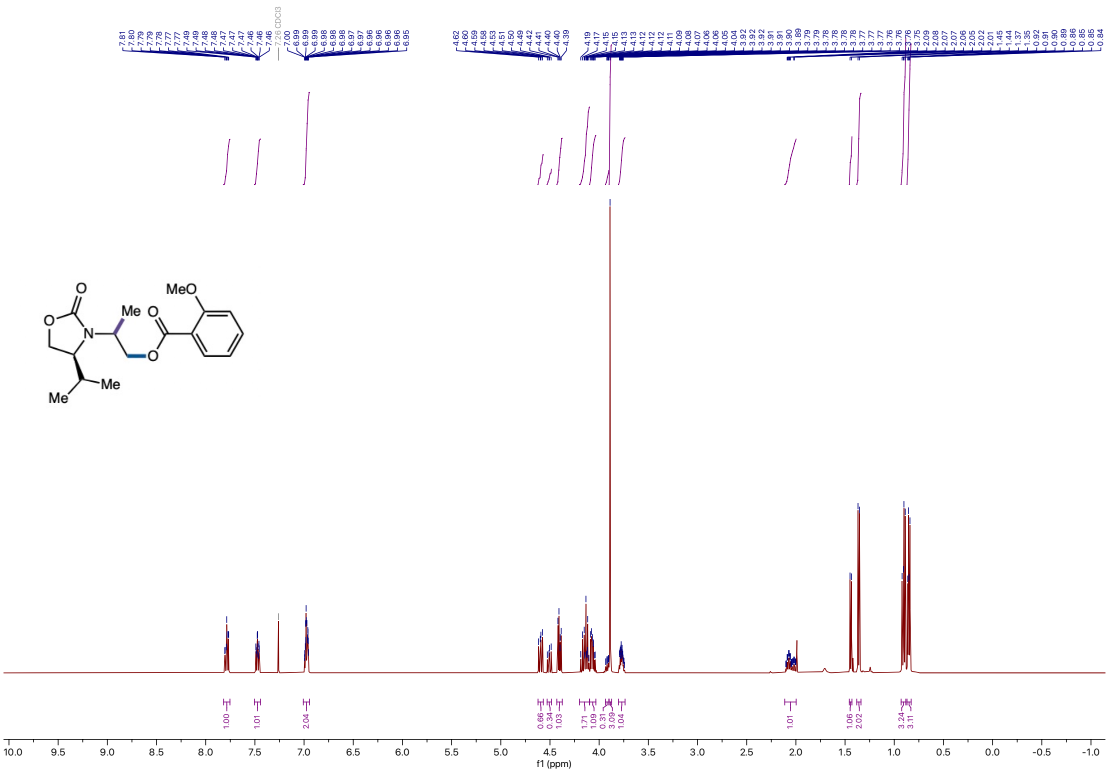

9, ^{13}C NMR, 126 MHz, $CDCl_3$

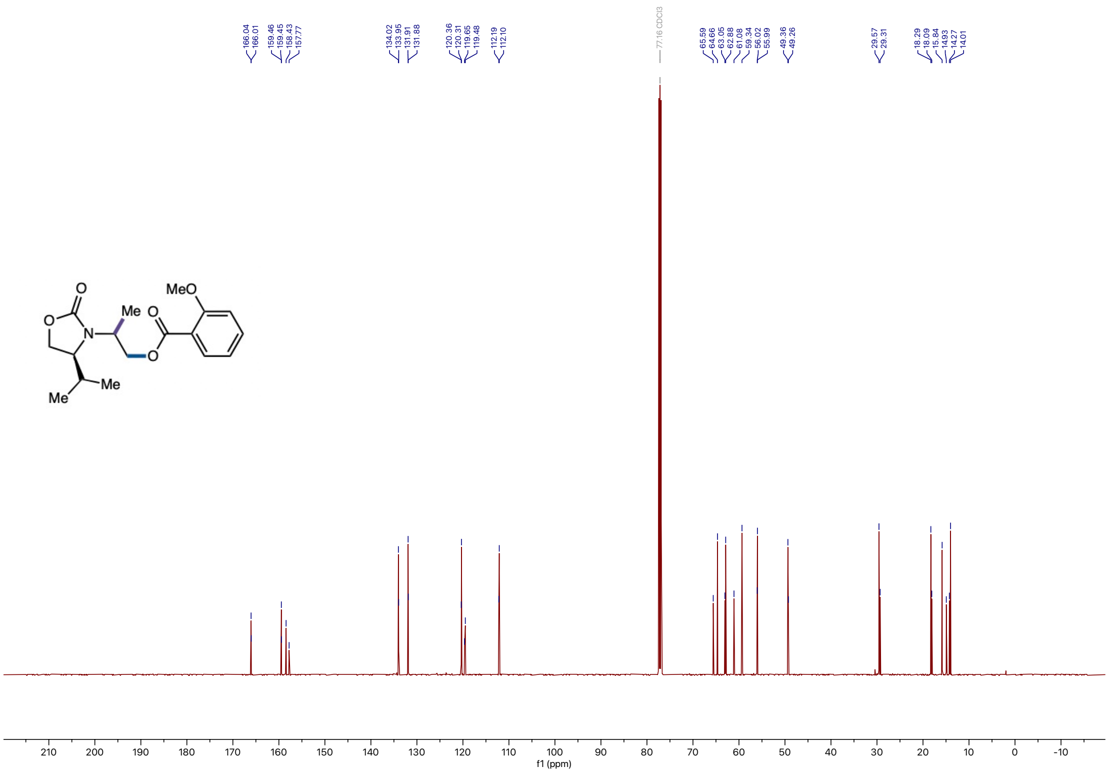

10, ^{1}H NMR, 500 MHz, $CDCl_3$

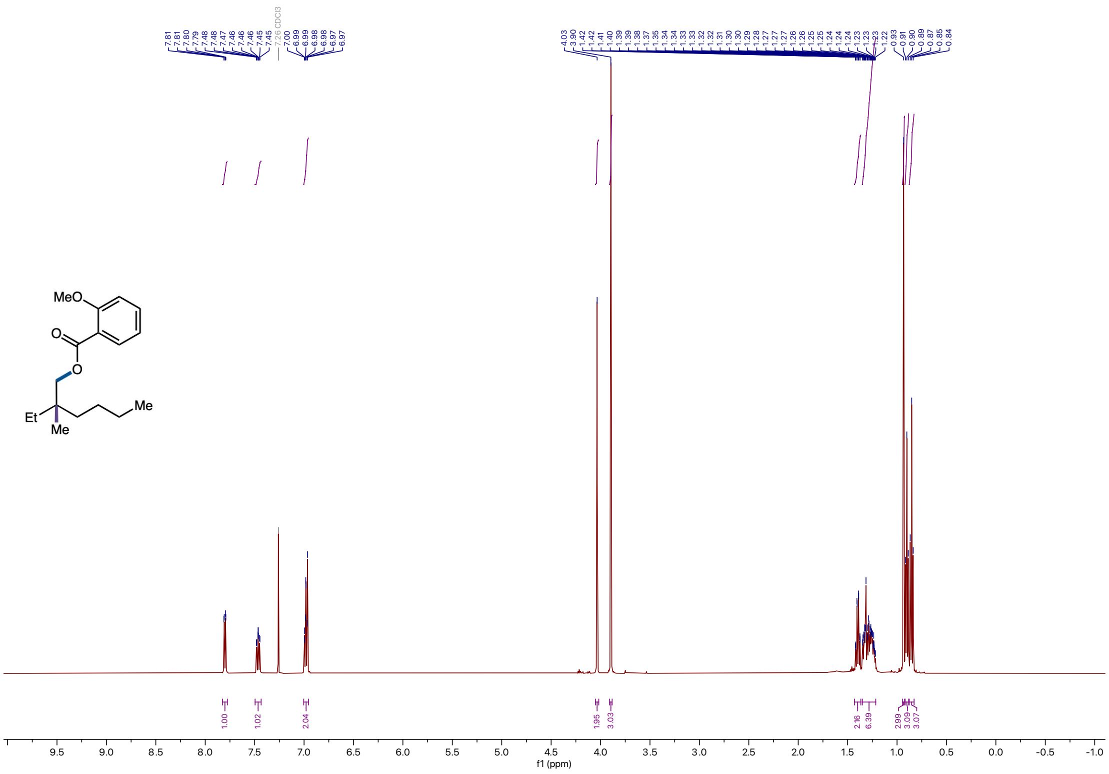

10, ^{13}C NMR, 126 MHz, $CDCl_3$

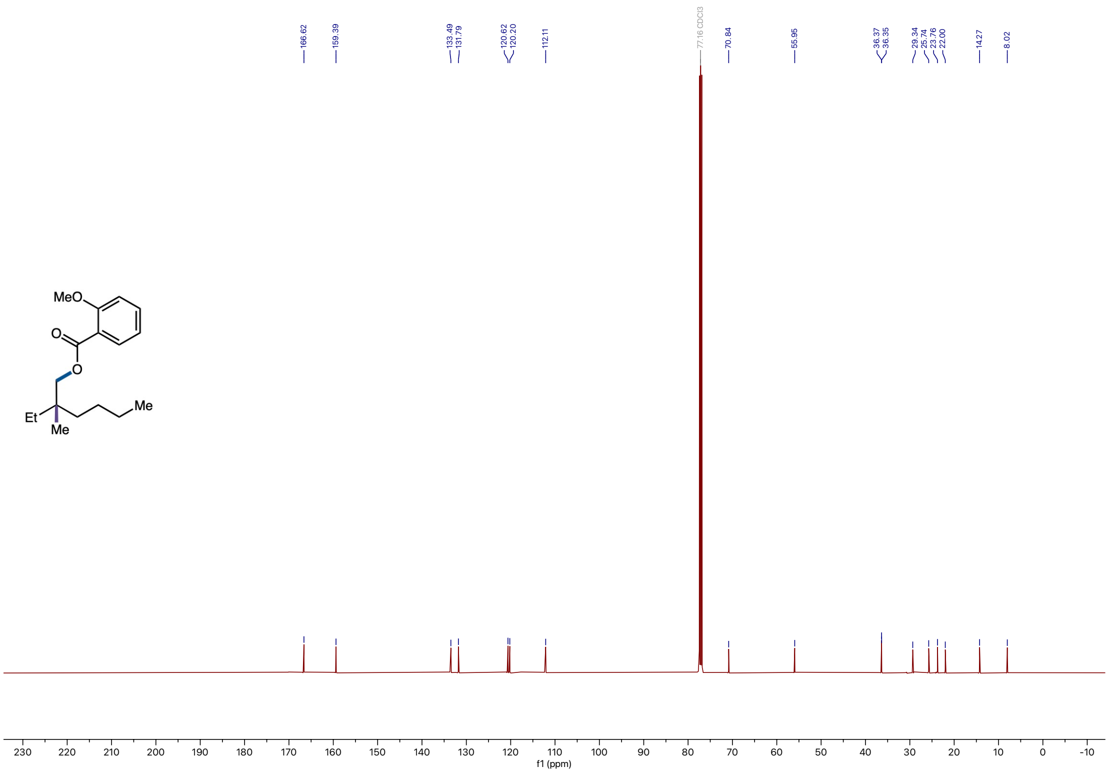

11, ^{1}H NMR, 500 MHz, $CDCl_3$

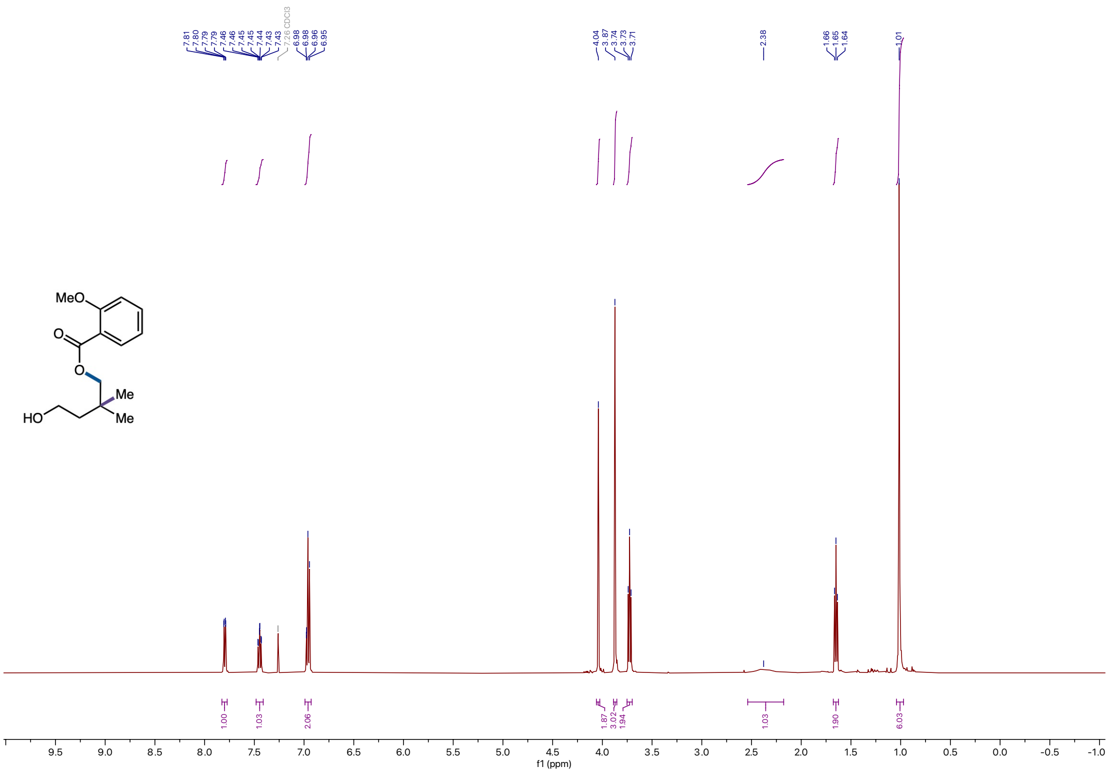

11, ^{13}C NMR, 126 MHz, $CDCl_3$

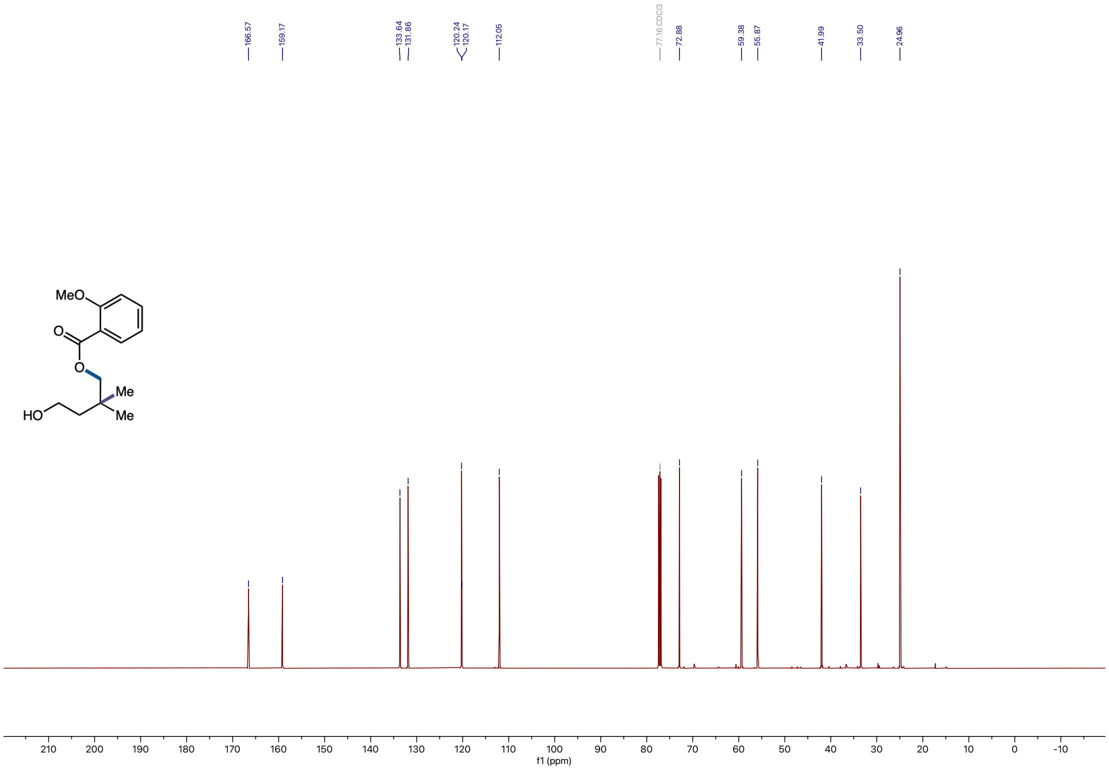

12, ^{1}H NMR, 500 MHz, $CDCl_3$

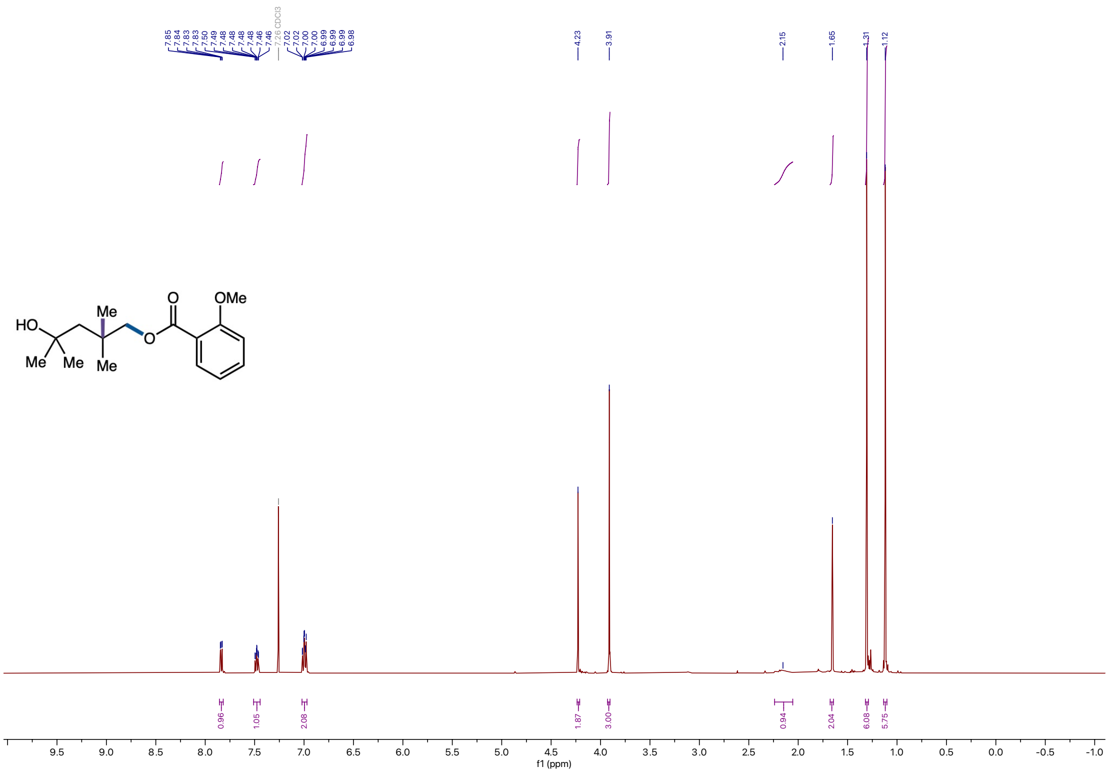

12, ^{13}C NMR, 126 MHz, $CDCl_3$

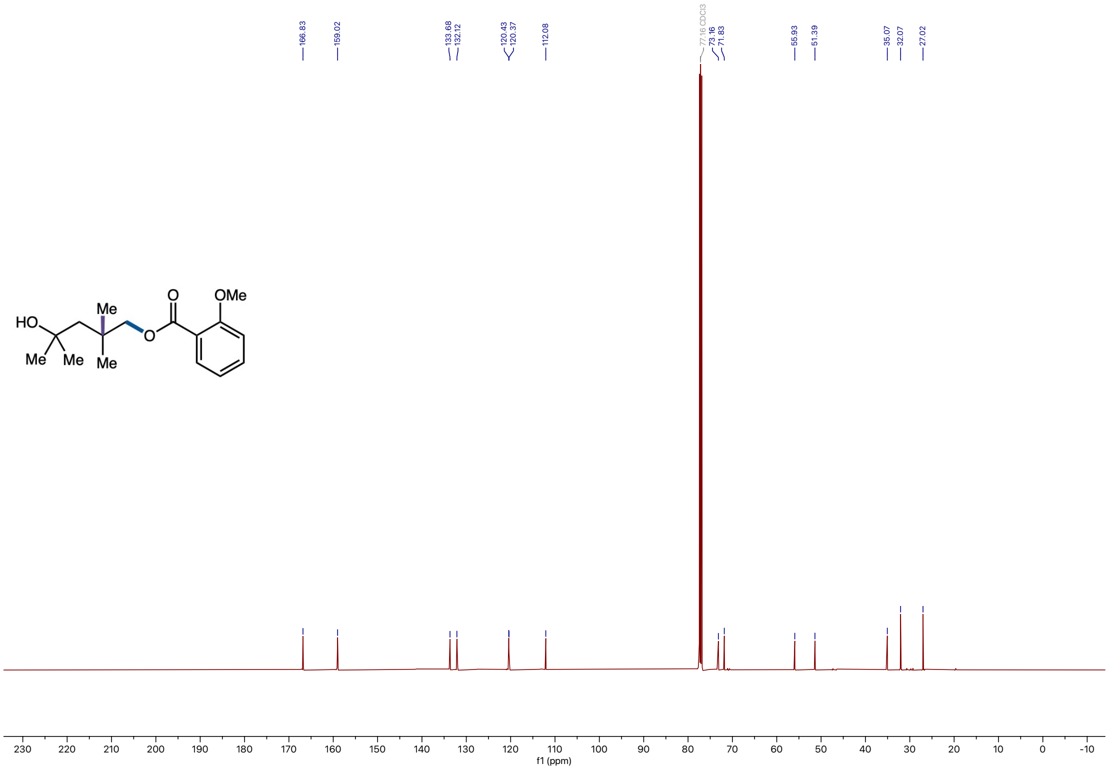

13, ^{1}H NMR, 500 MHz, $CDCl_3$

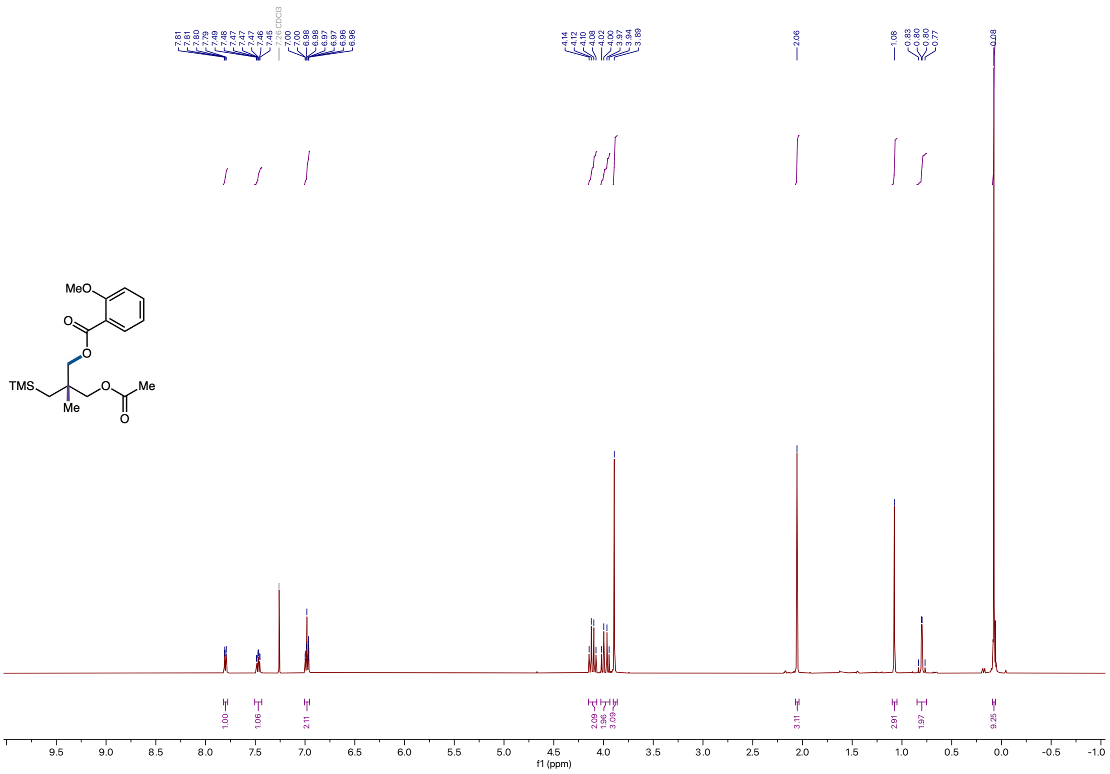

13, ^{13}C NMR, 126 MHz, $CDCl_3$

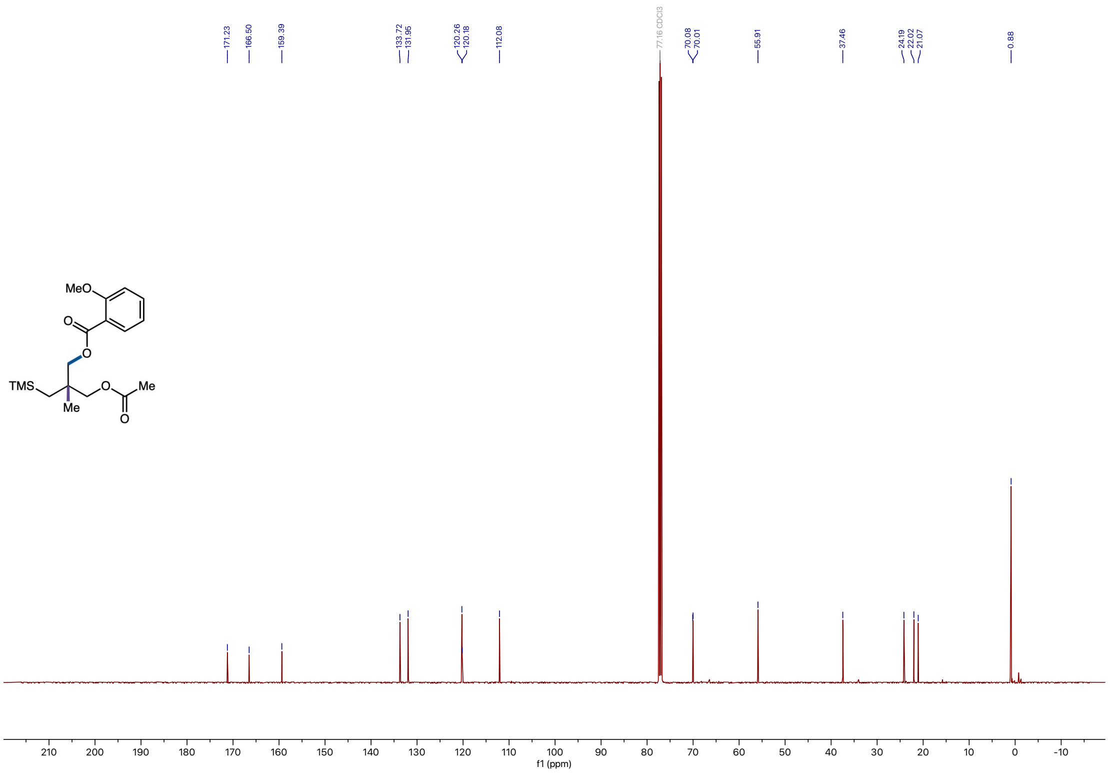

14, ^{1}H NMR, 500 MHz, CDCl$_3$

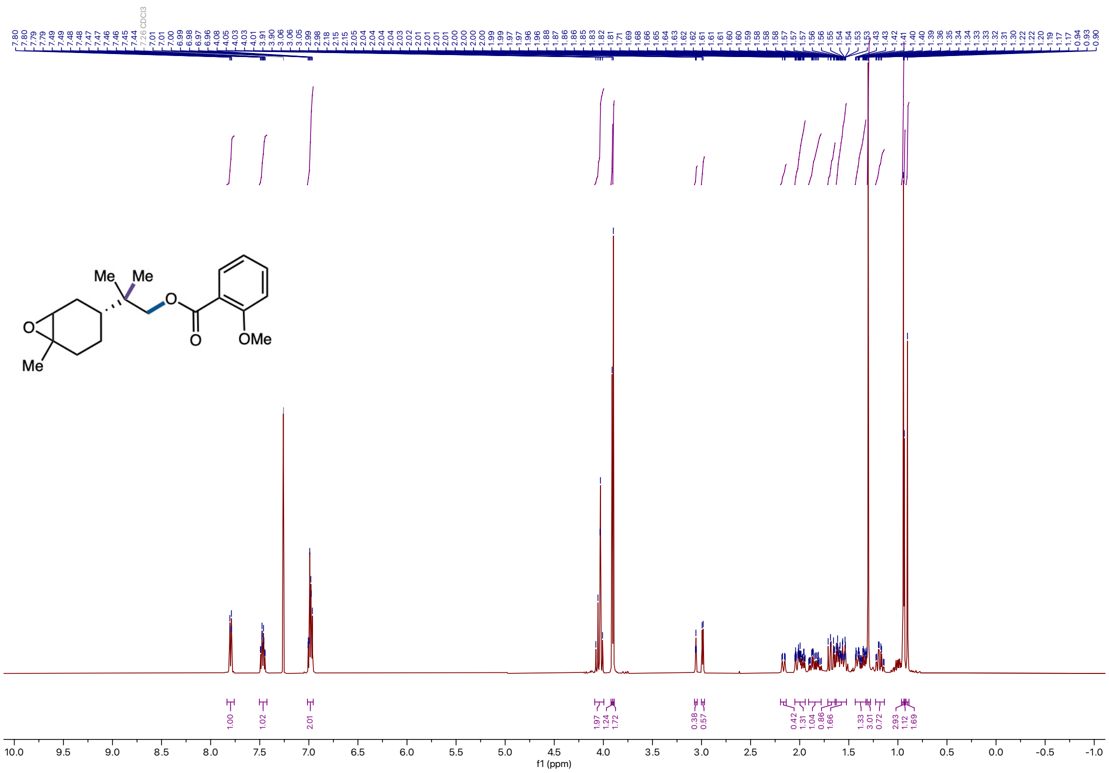

14, ^{13}C NMR, 126 MHz, CDCl$_3$

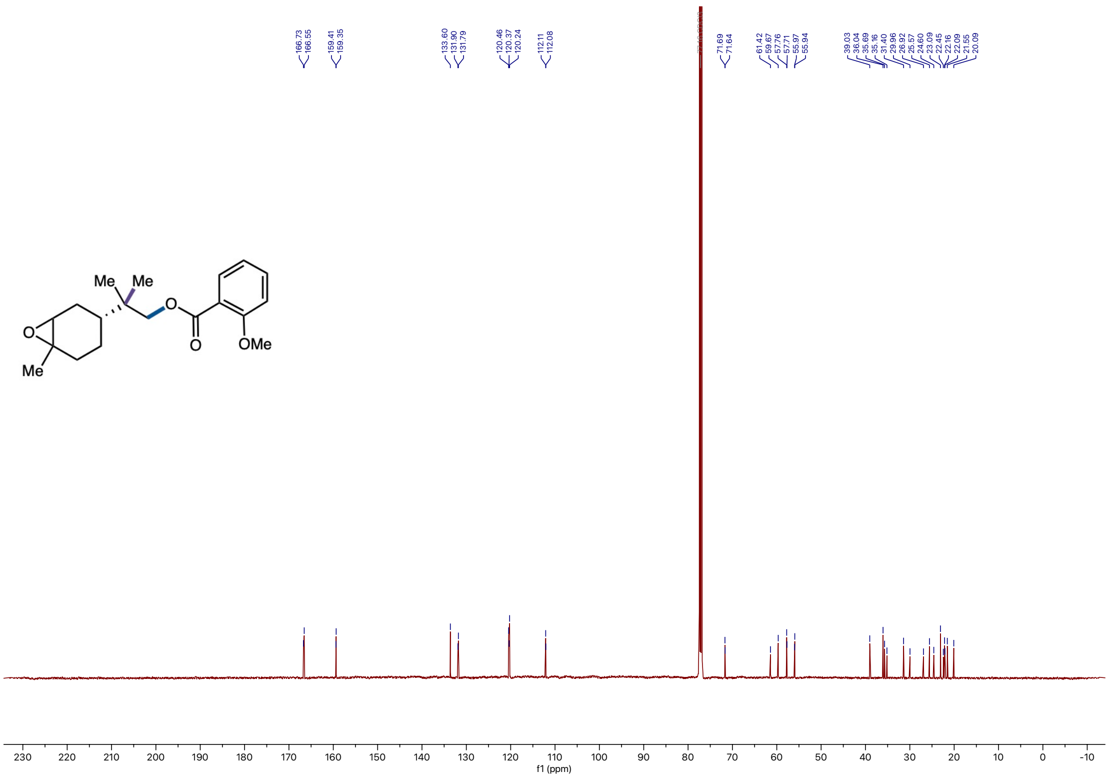

15, ^{1}H NMR, 500 MHz, $CDCl_3$

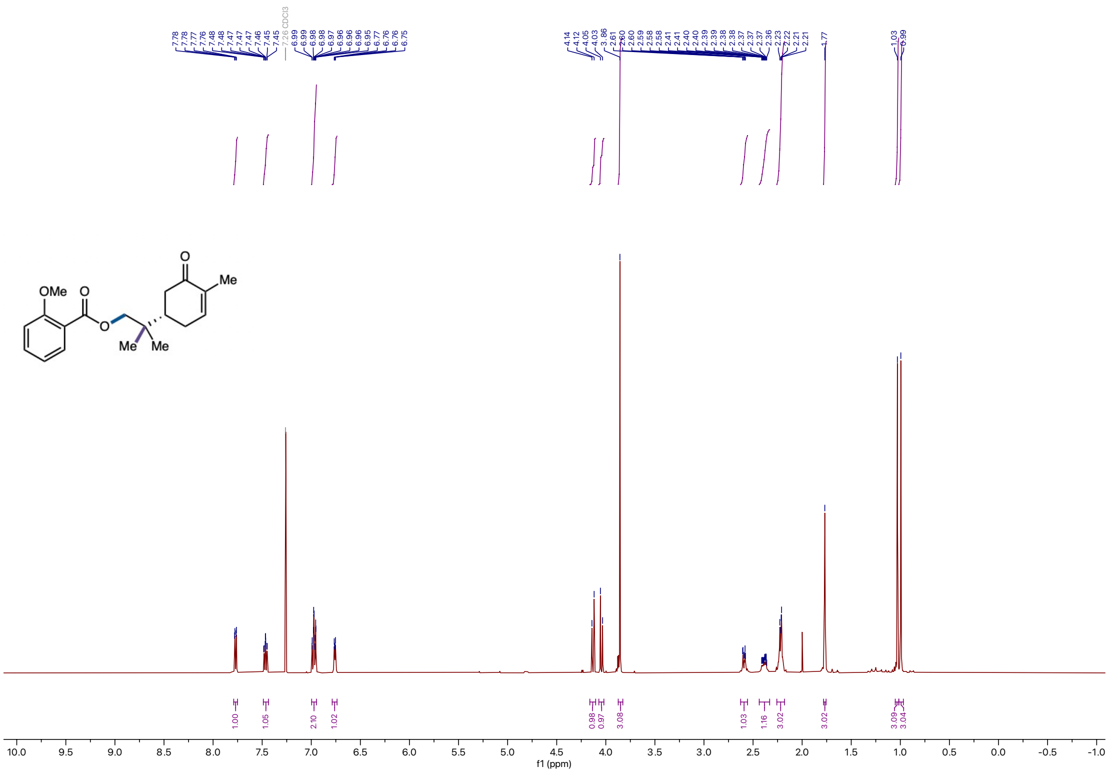

15, ^{13}C NMR, 126 MHz, $CDCl_3$

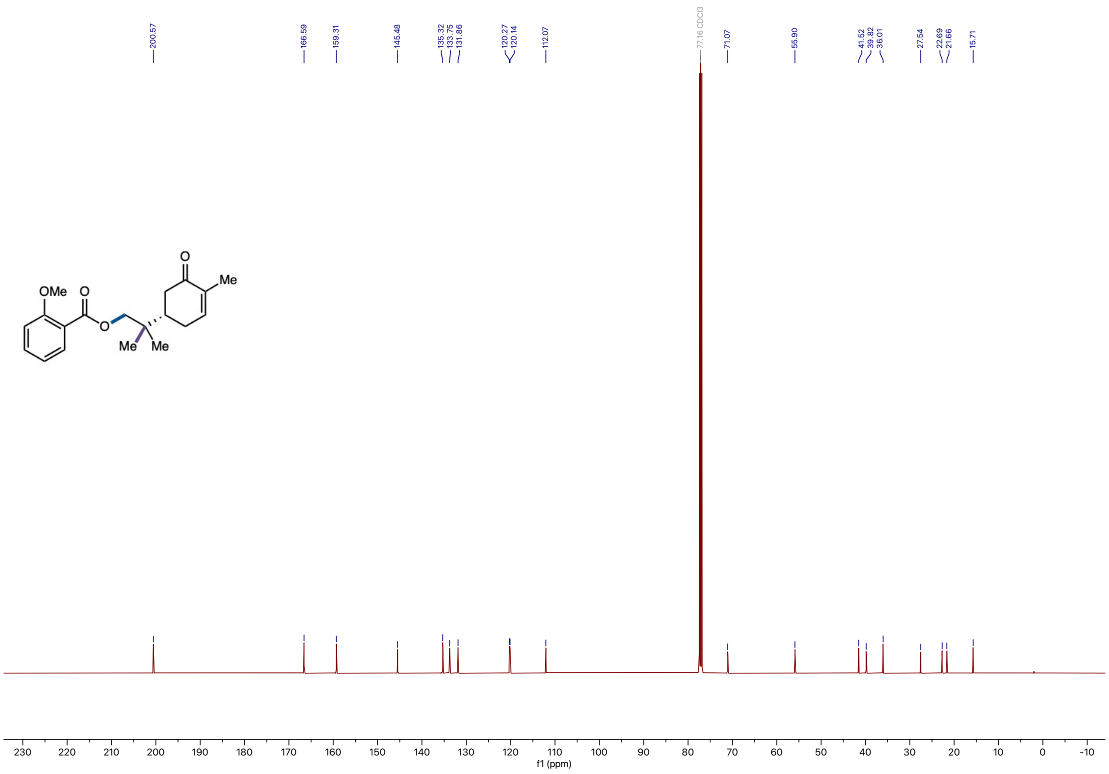

16, ^{1}H NMR, 500 MHz, $CDCl_3$

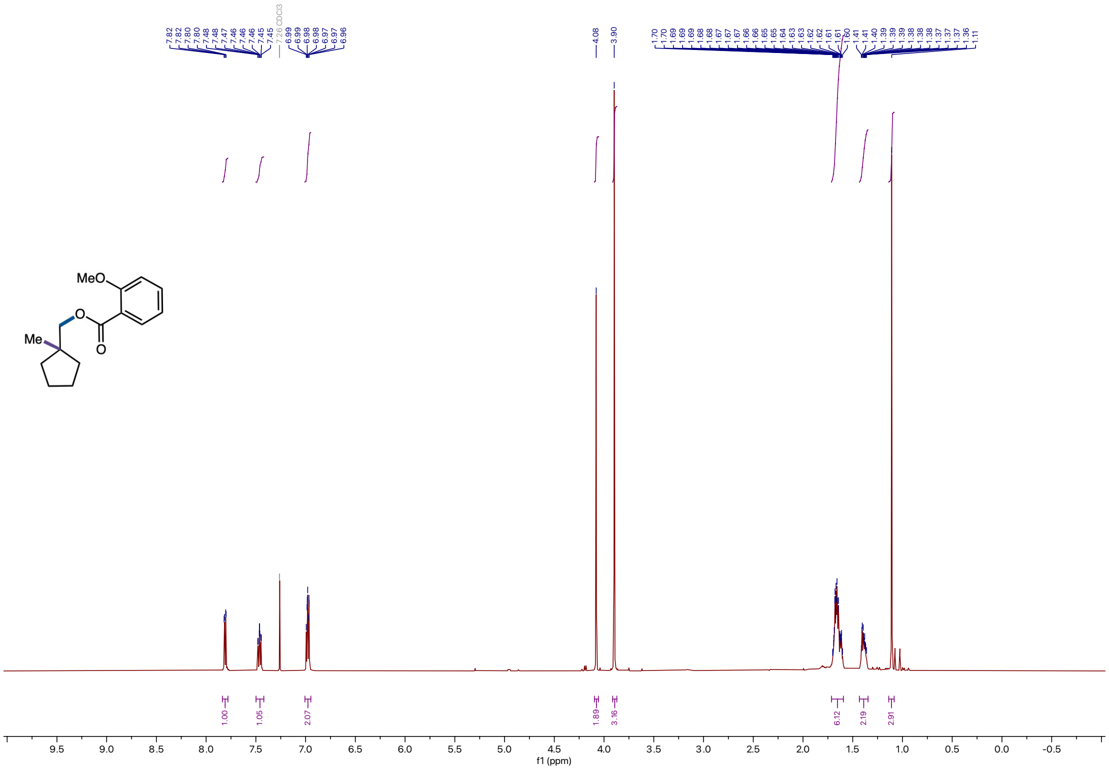

16, ^{13}C NMR, 126 MHz, $CDCl_3$

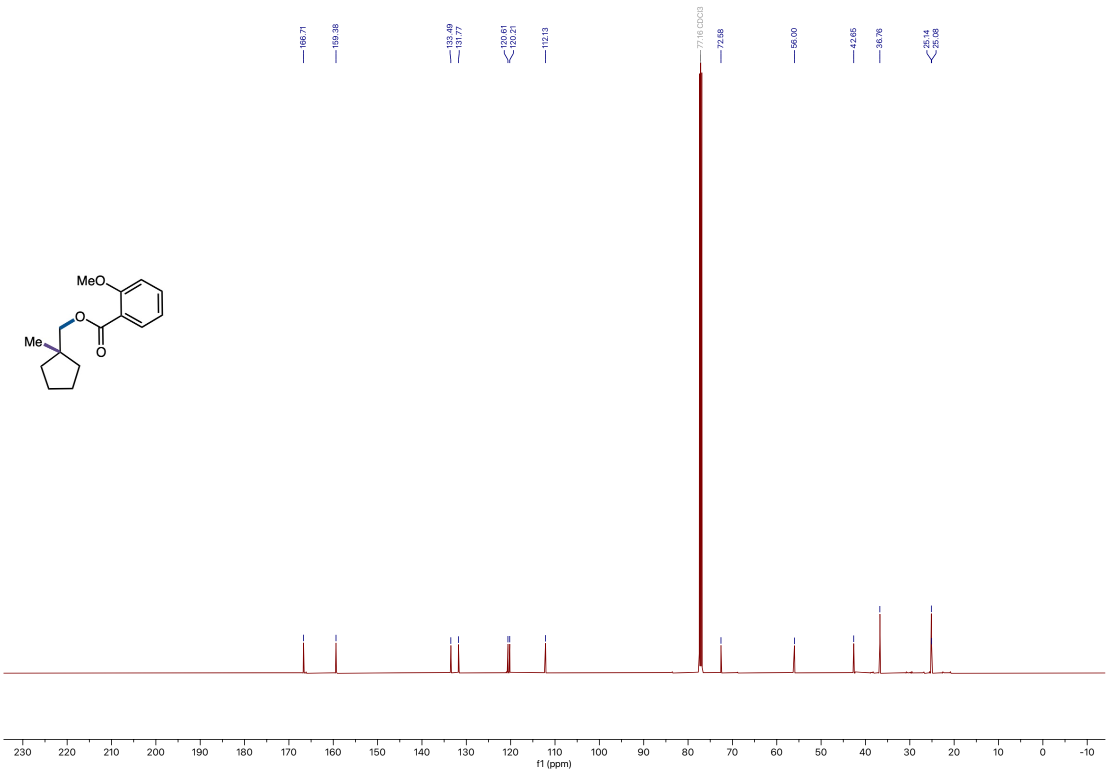

17, ^{1}H NMR, 500 MHz, $CDCl_3$

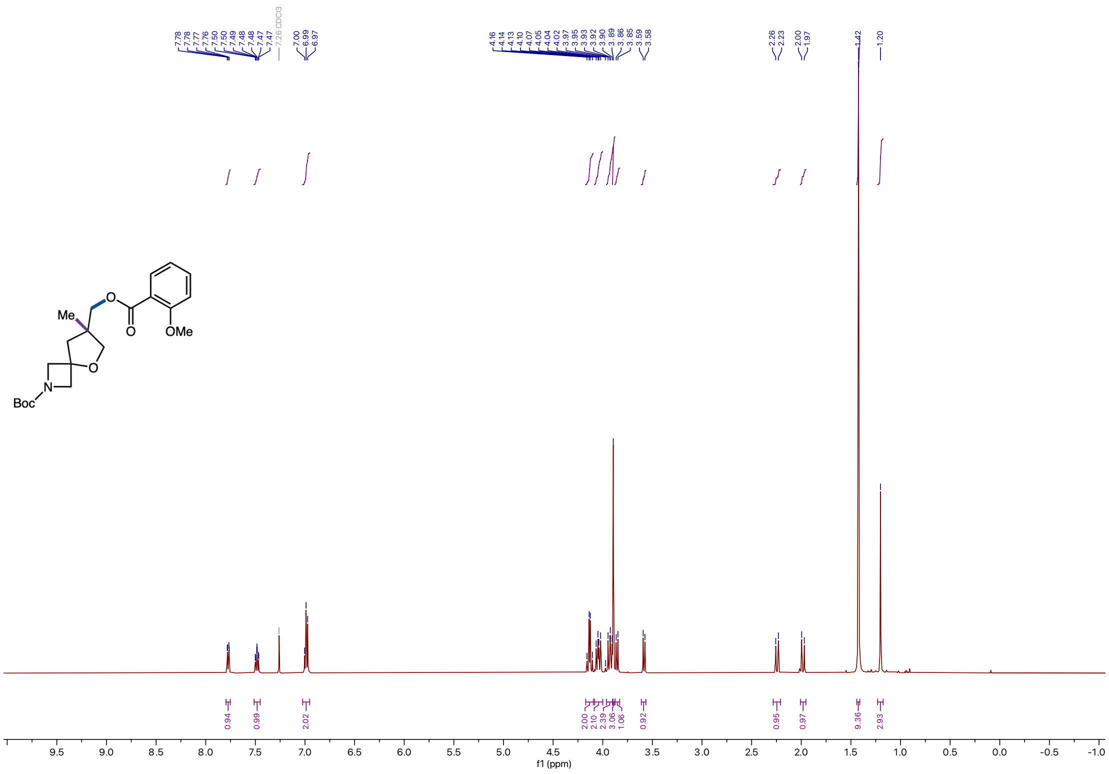

17, ^{13}C NMR, 126 MHz, $CDCl_3$

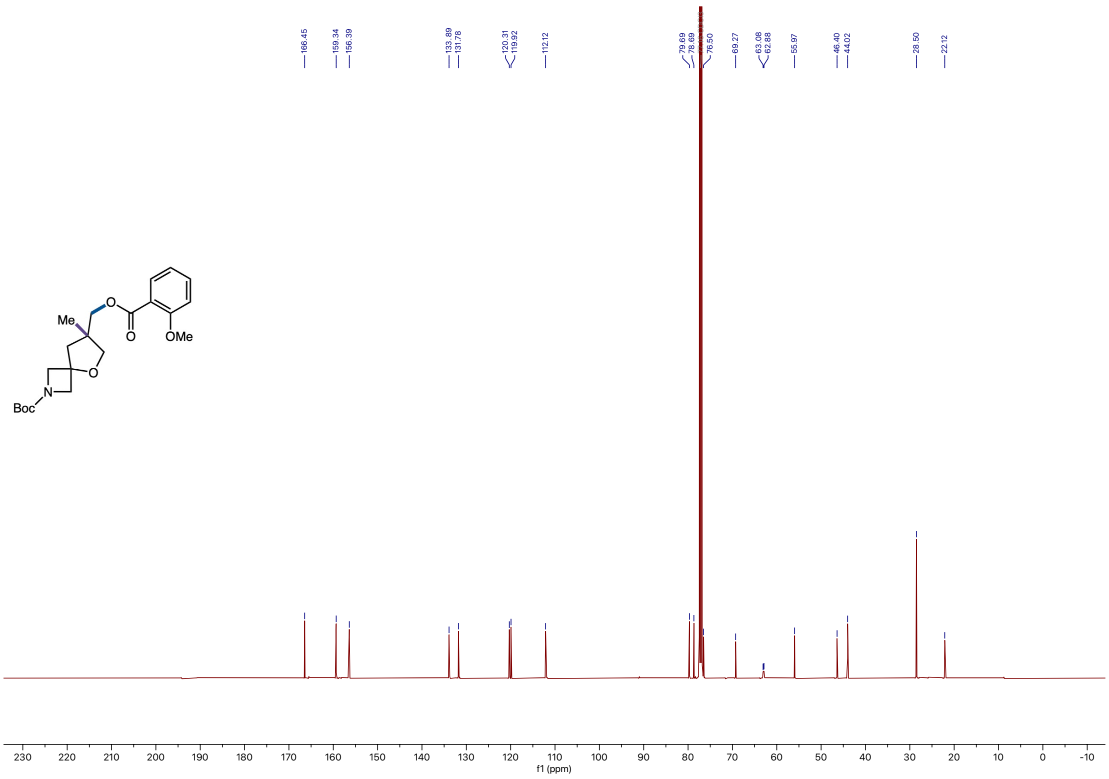

18, ^{1}H NMR, 500 MHz, $CDCl_3$

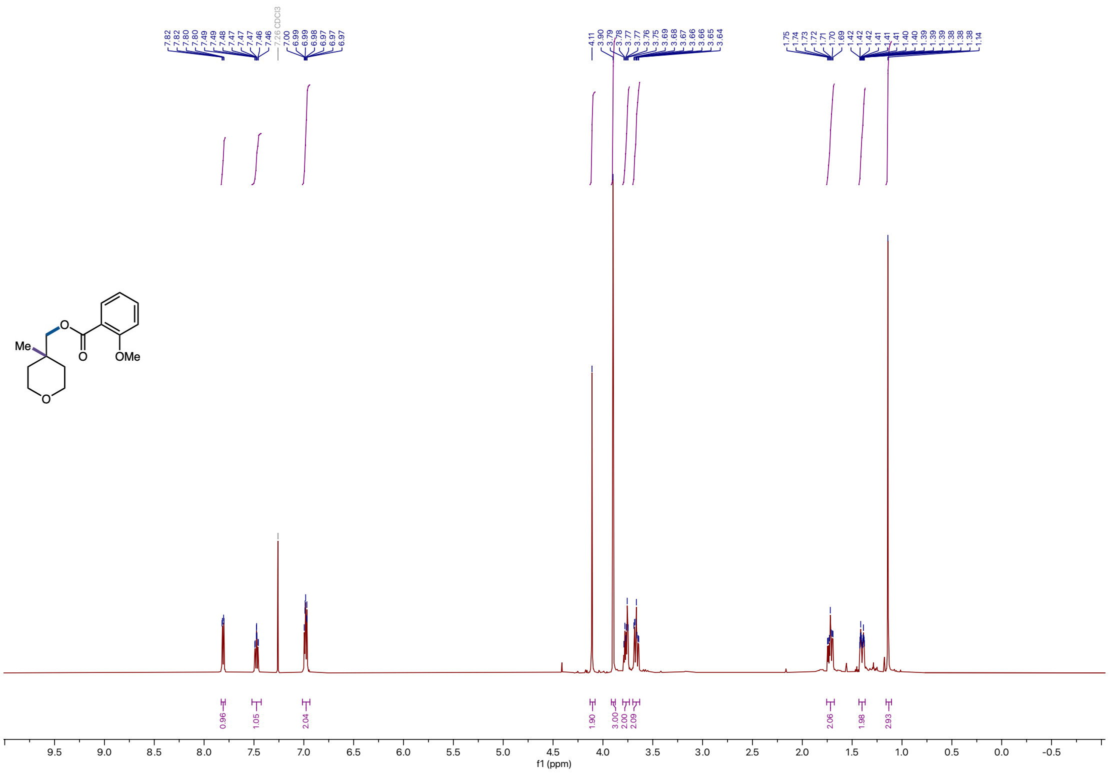

18, ^{13}C NMR, 126 MHz, $CDCl_3$

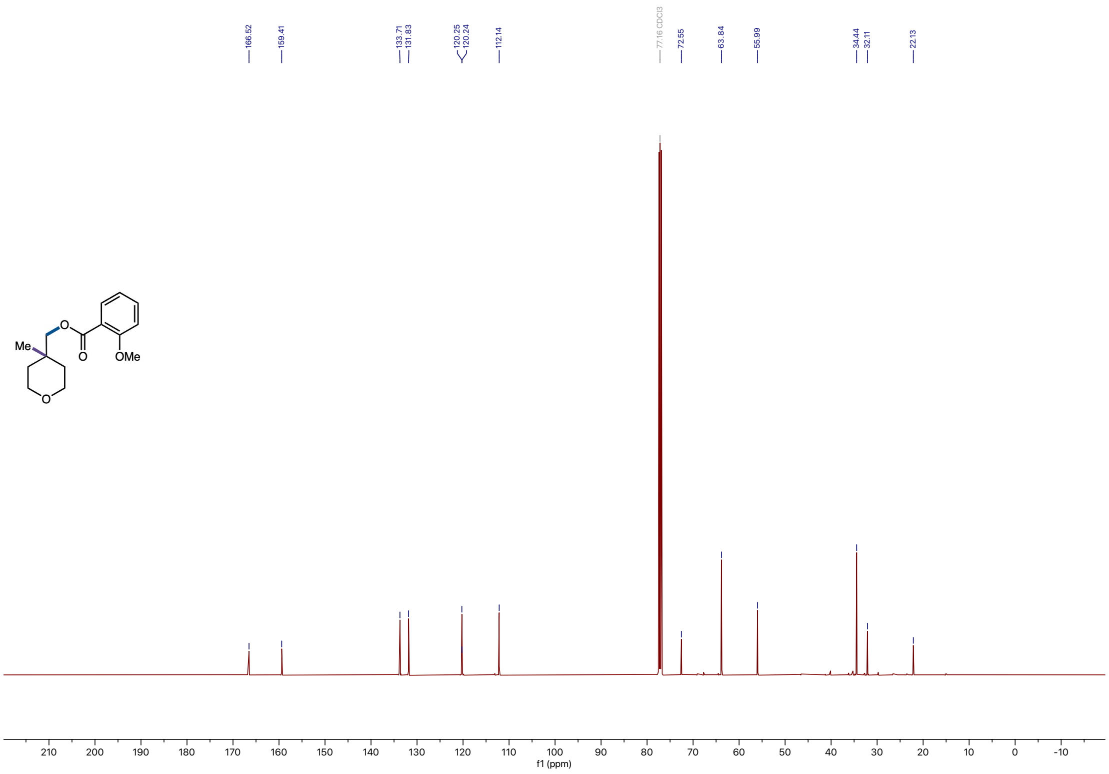

5, ^{1}H NMR, 500 MHz, $CDCl_3$

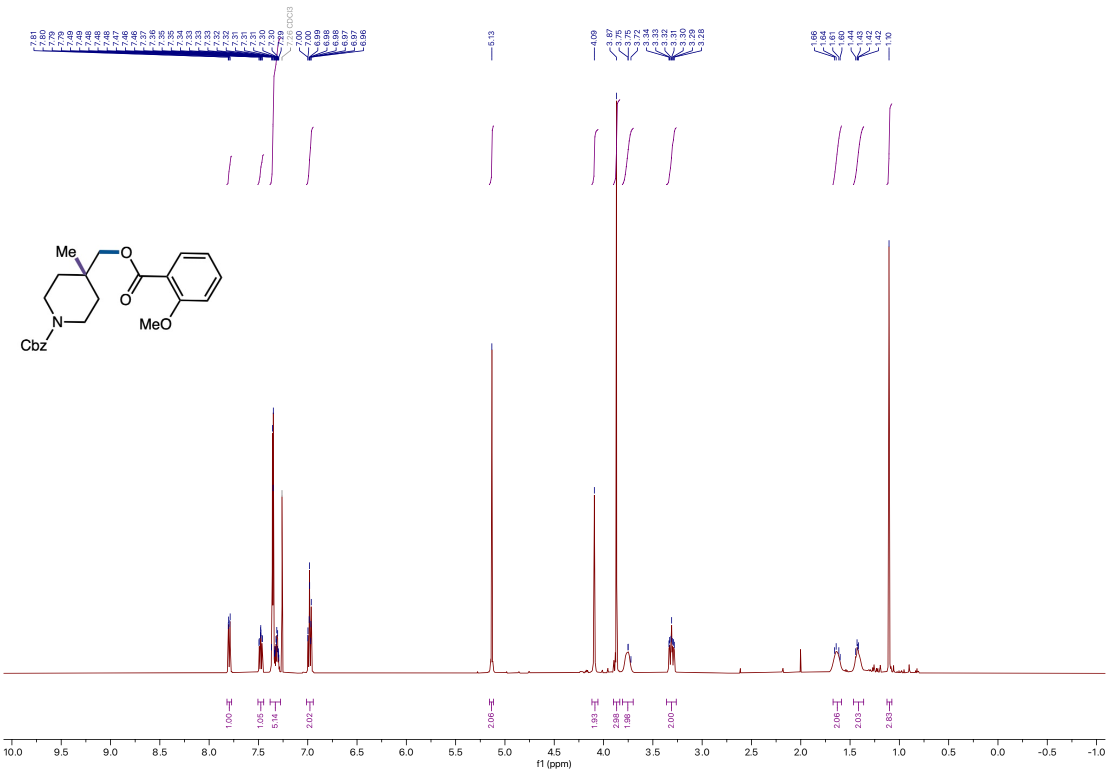

5, ^{13}C NMR, 126 MHz, $CDCl_3$

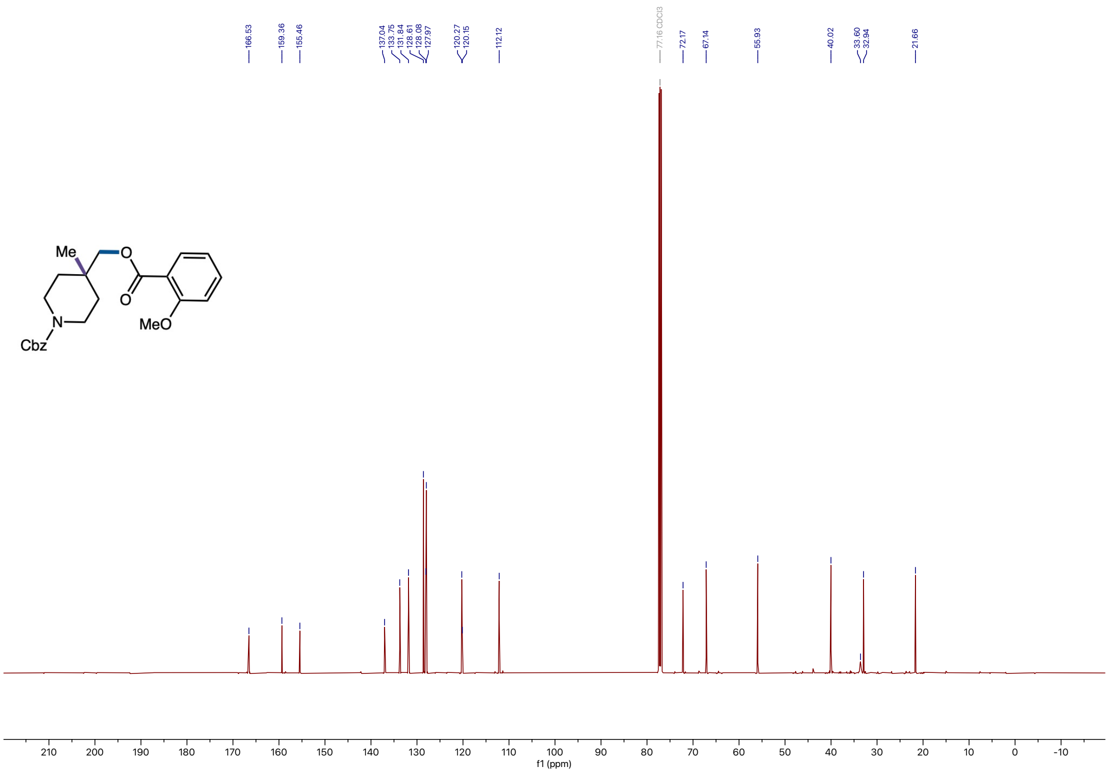

19, ^{1}H NMR, 500 MHz, $CDCl_3$

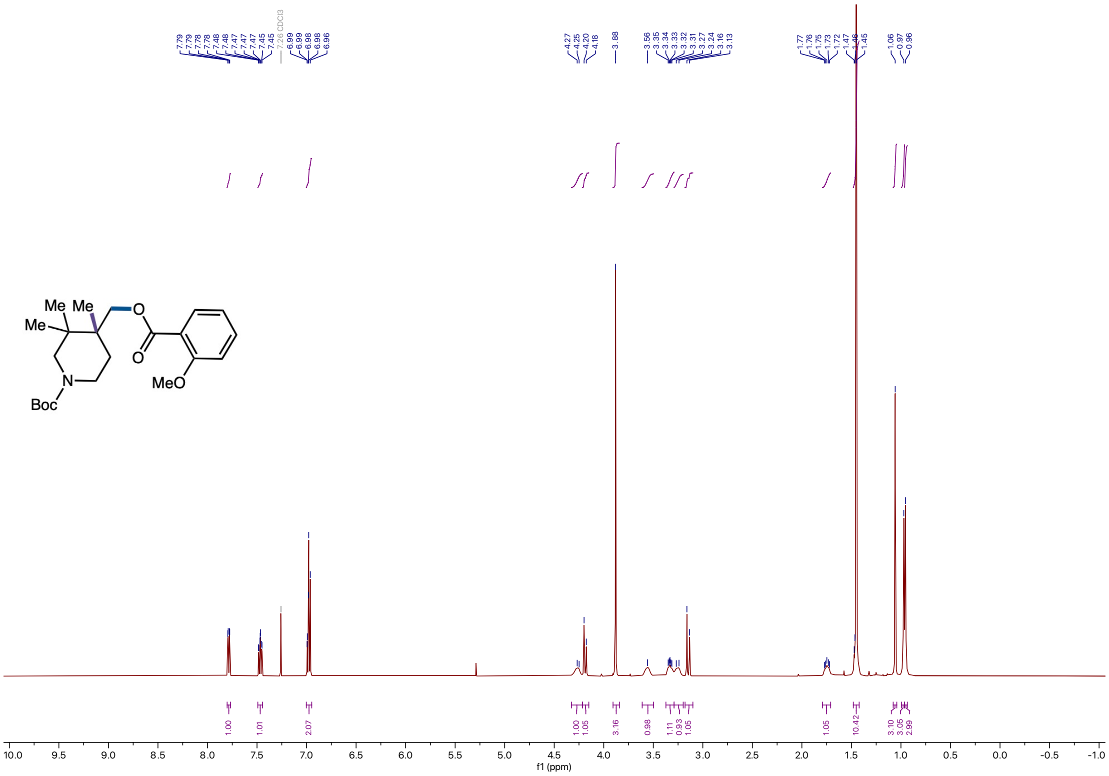

19, ^{13}C NMR, 126 MHz, $CDCl_3$

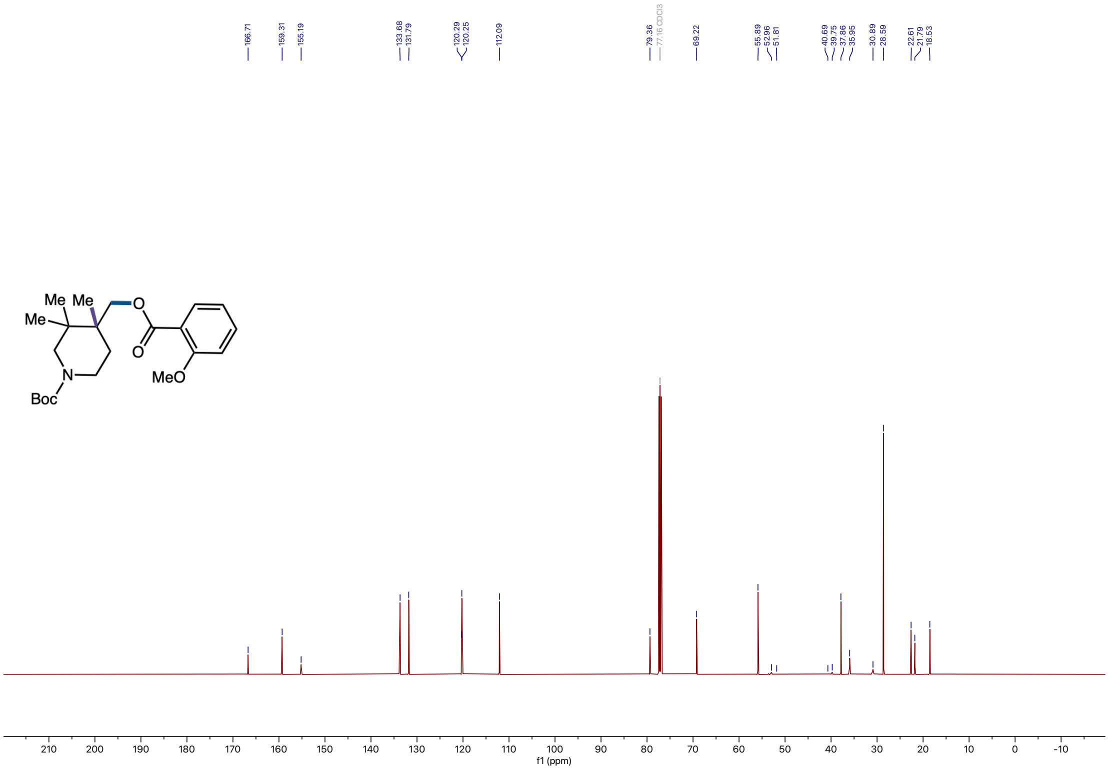

20, ^{1}H NMR, 500 MHz, $CDCl_3$

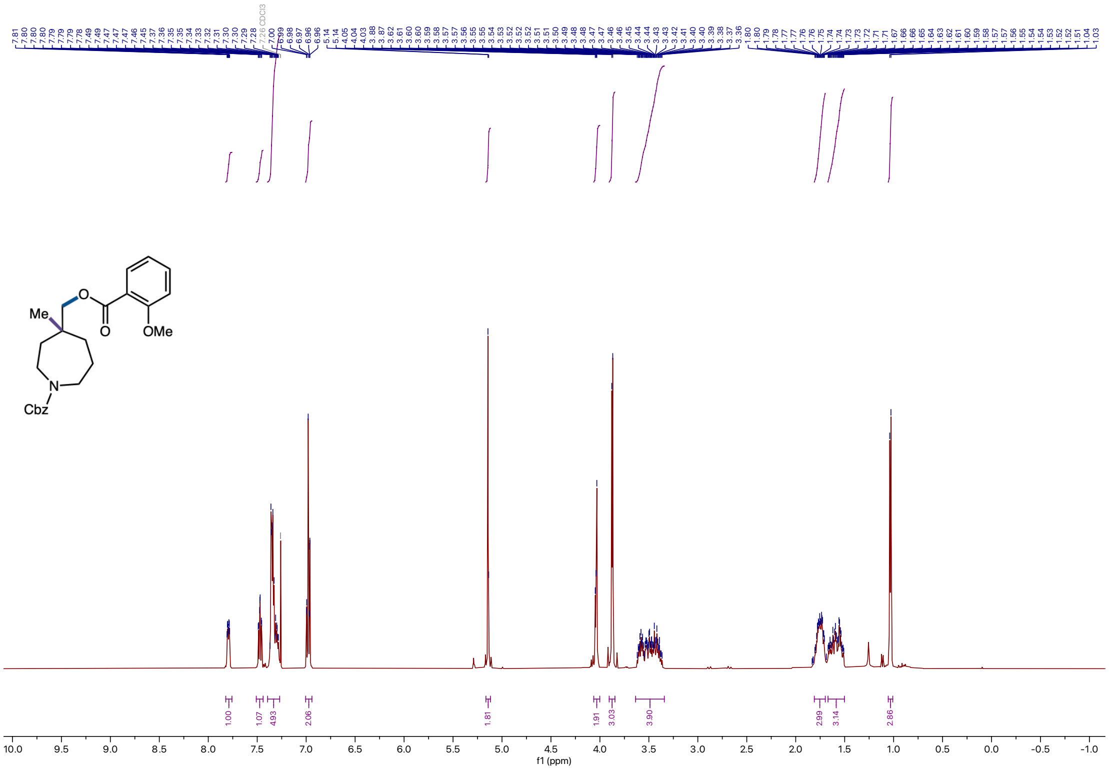

20, ^{13}C NMR, 126 MHz, $CDCl_3$

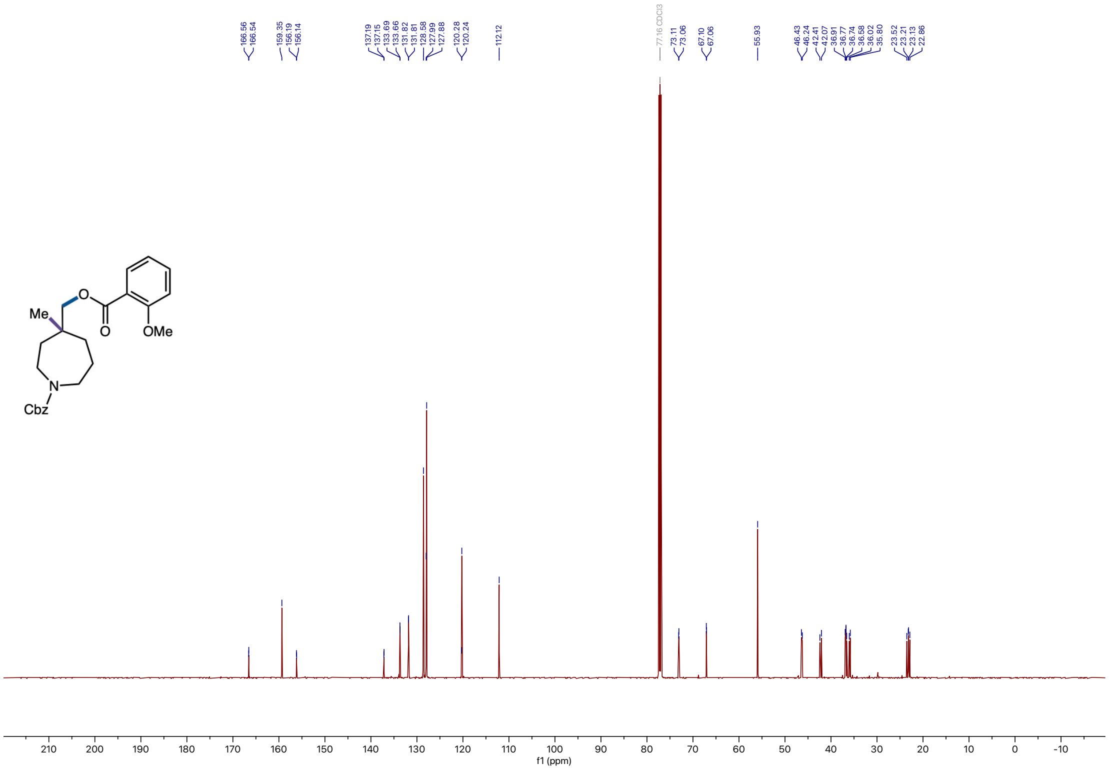

21, ^{1}H NMR, 500 MHz, $CDCl_3$

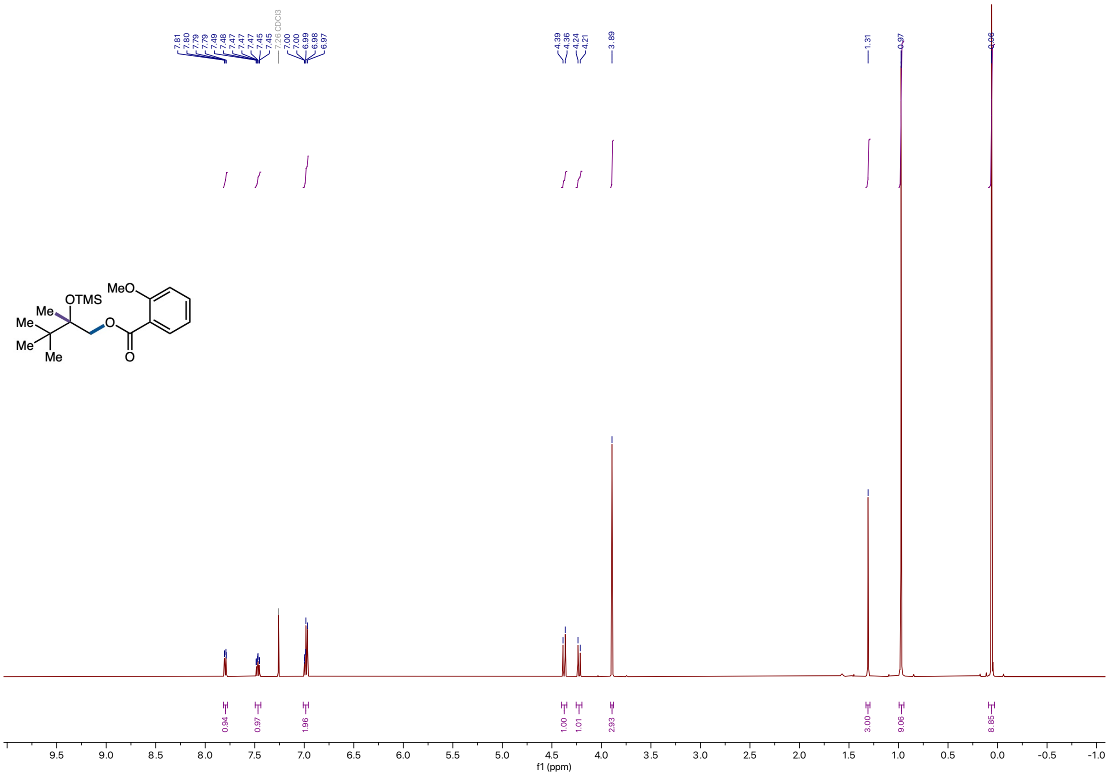

21, ^{13}C NMR, 126 MHz, $CDCl_3$

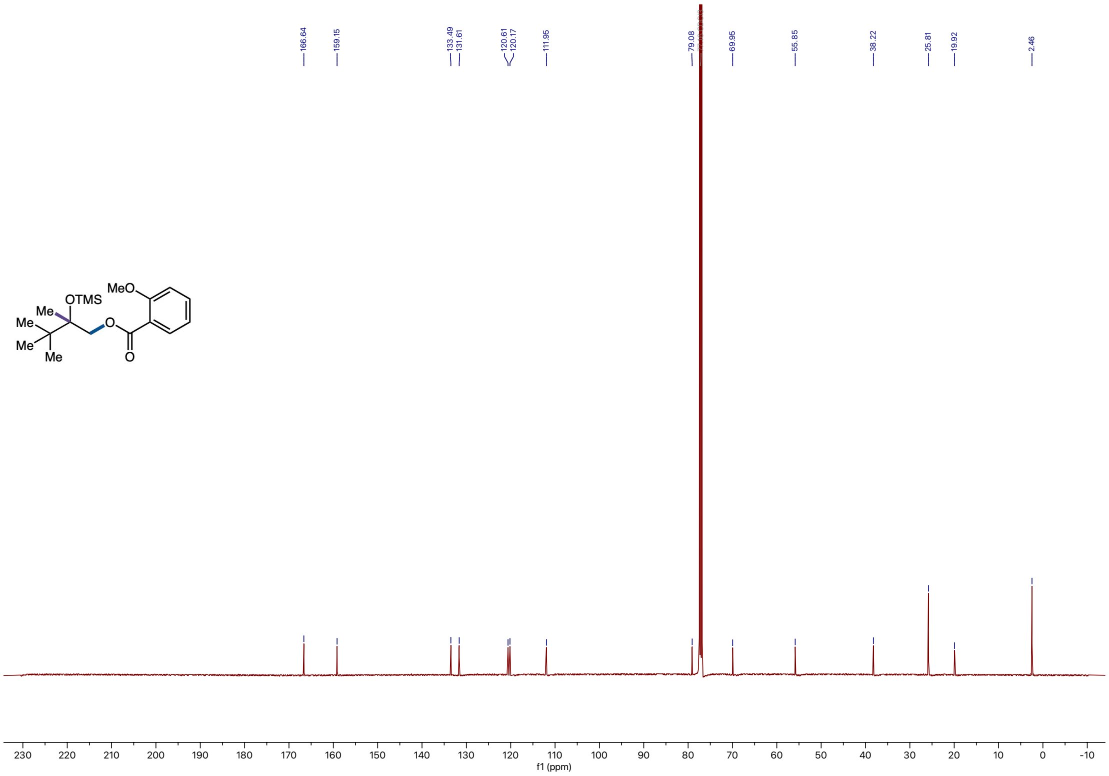

22, ^{1}H NMR, 500 MHz, $CDCl_3$

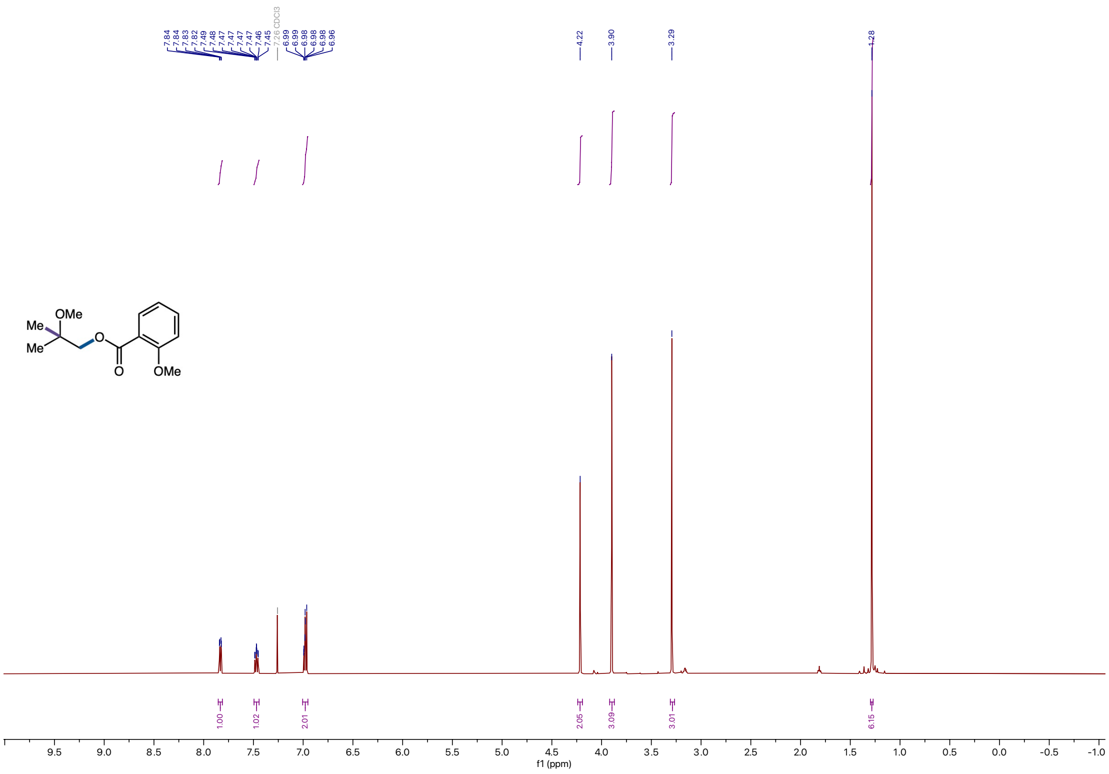

22, ^{13}C NMR, 126 MHz, $CDCl_3$

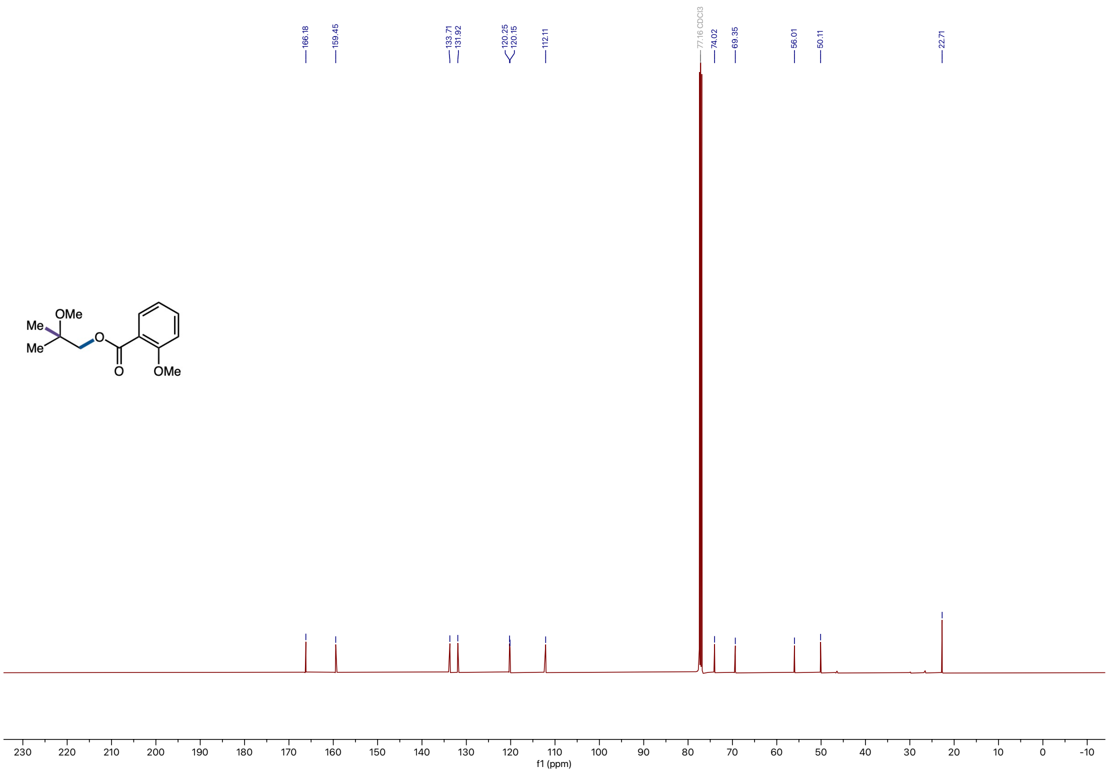

23-1 (diastereomer 1), ^{1}H NMR, 500 MHz, $CDCl_3$

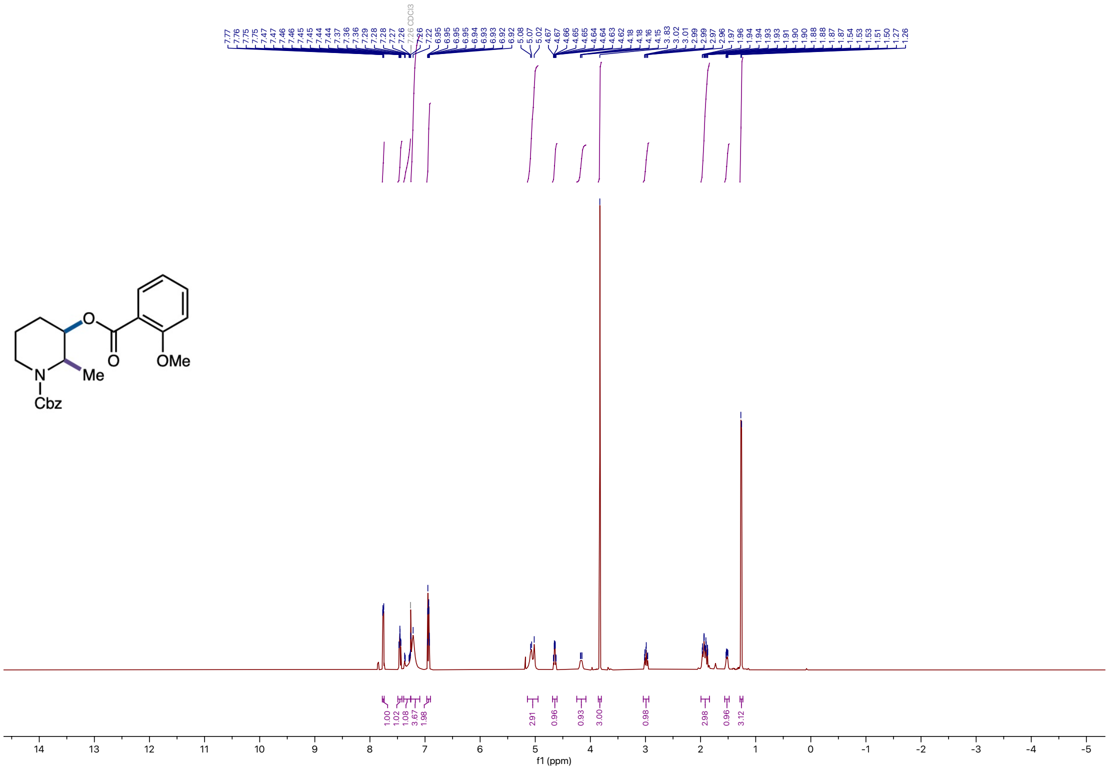

23-1 (diastereomer 1), ^{13}C NMR, 126 MHz, $CDCl_3$

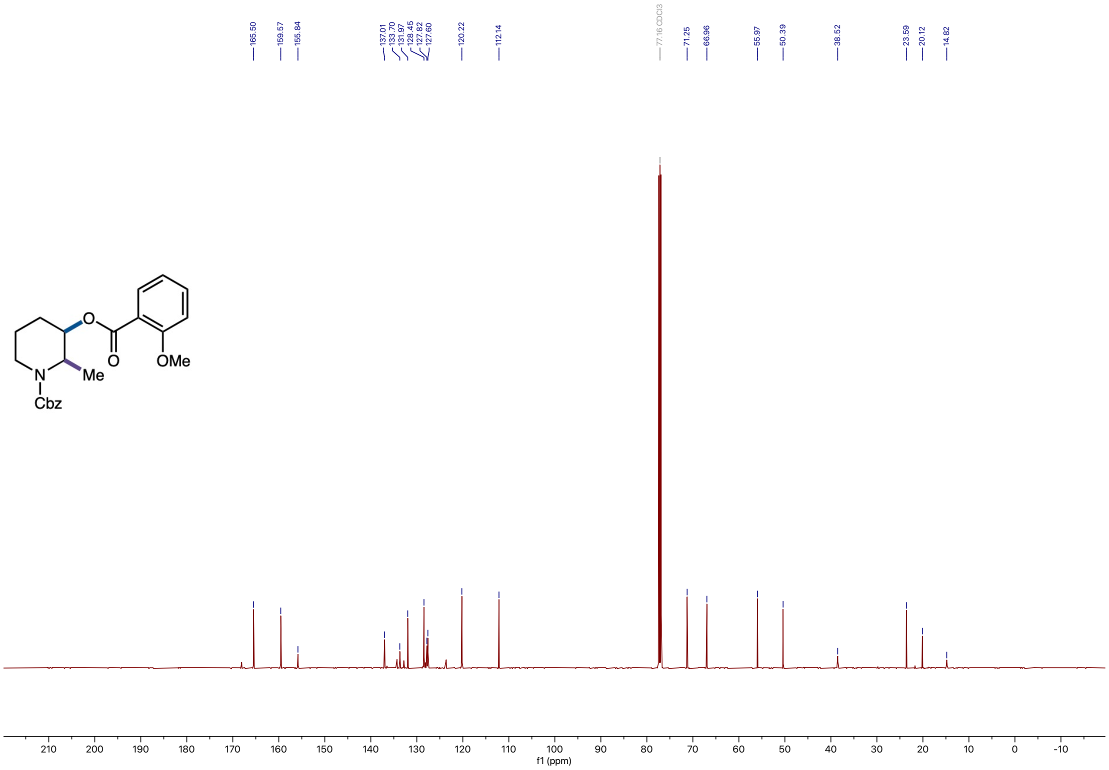

23-2 (diastereomer 2), ^{1}H NMR, 500 MHz, $CDCl_3$

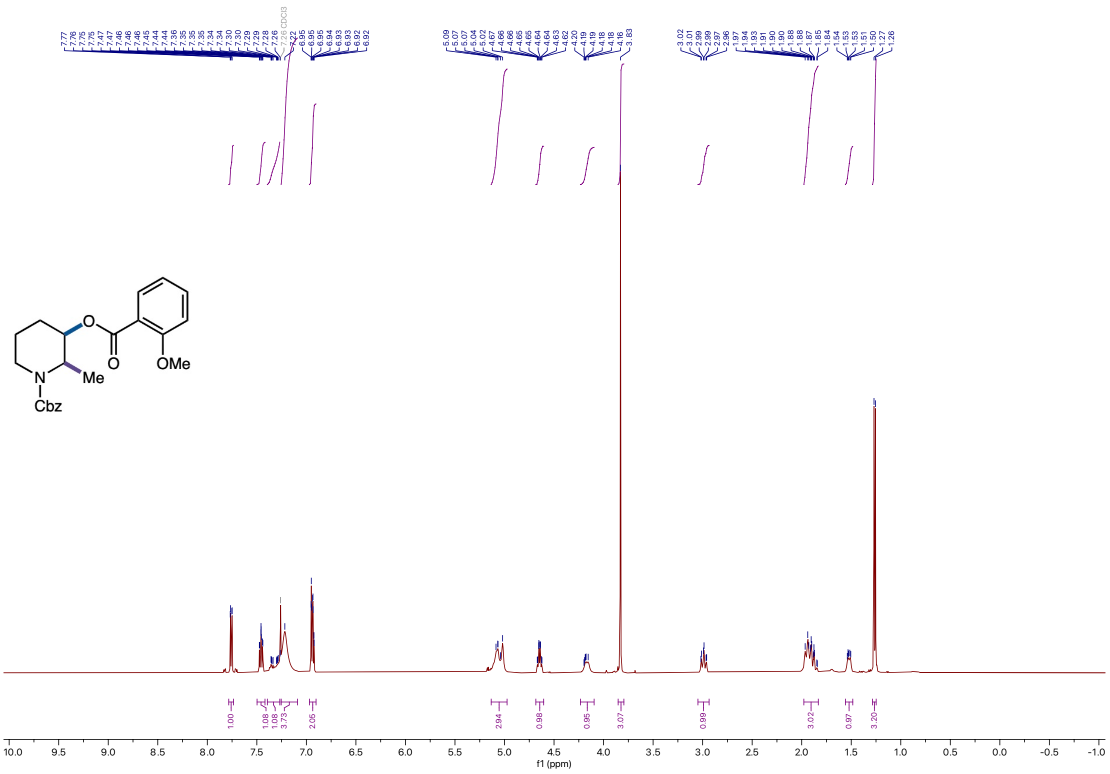

23-2 (diastereomer 2), ^{13}C NMR, 126 MHz, $CDCl_3$

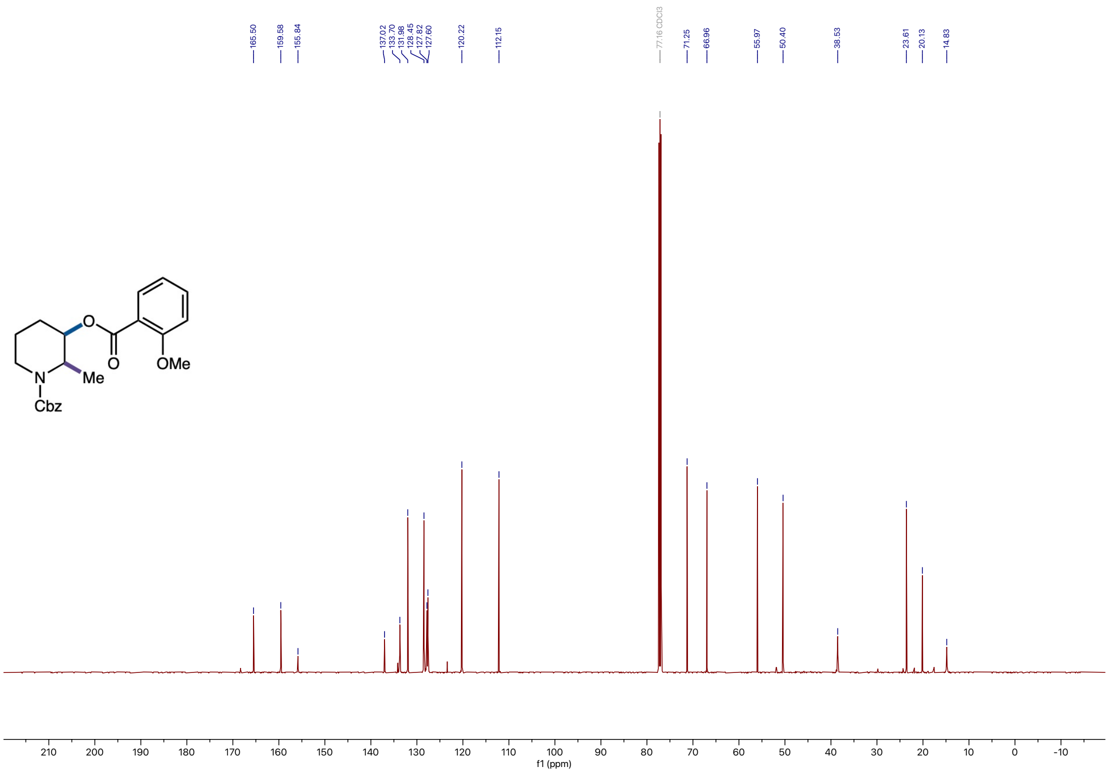

24, ^{1}H NMR, 500 MHz, $CDCl_3$

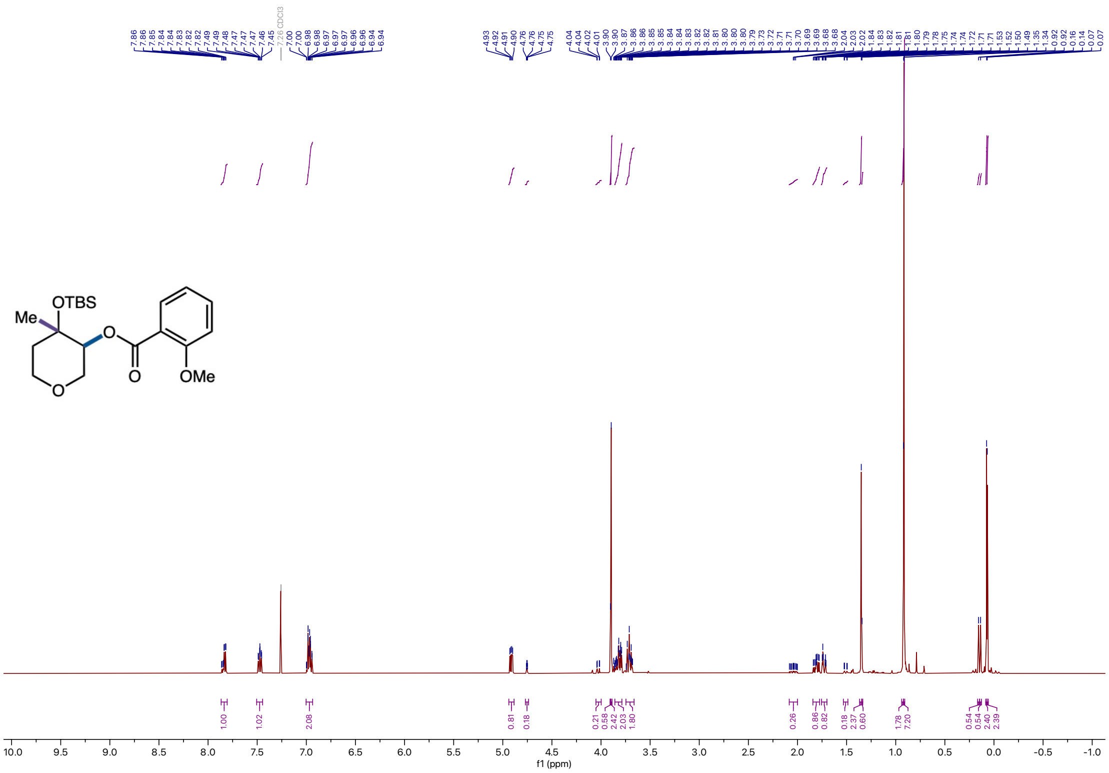

24, ^{13}C NMR, 126 MHz, $CDCl_3$

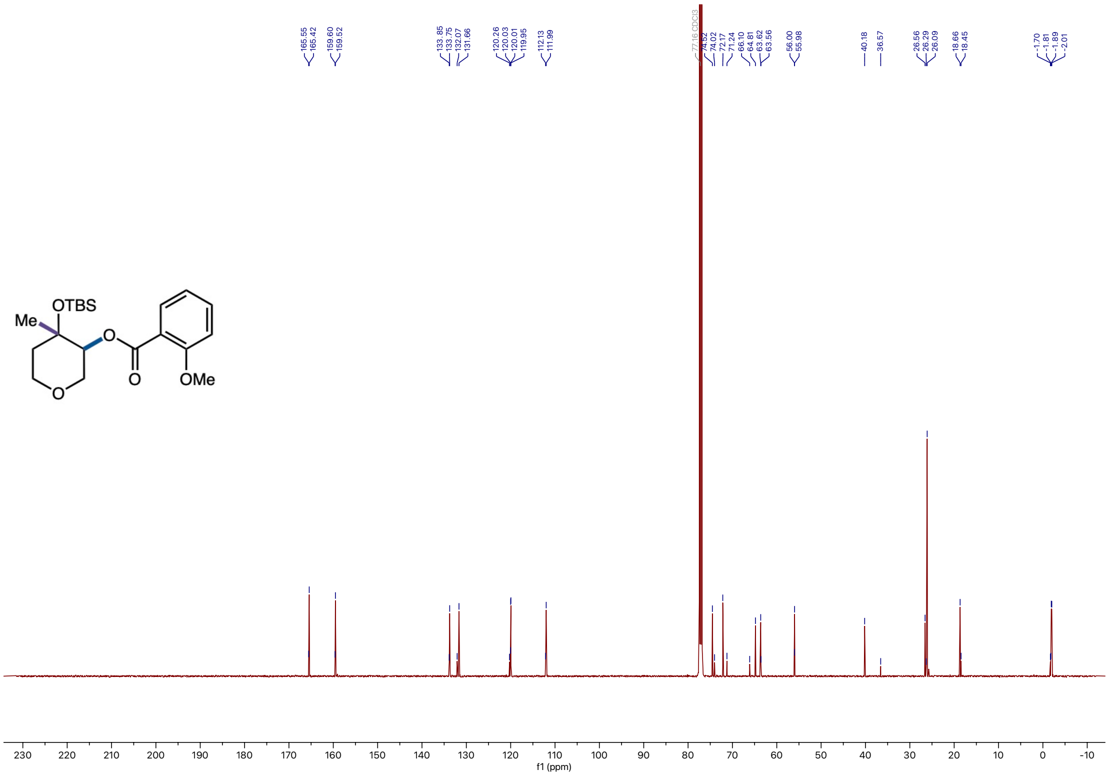

25, ^{1}H NMR, 500 MHz, $CDCl_3$

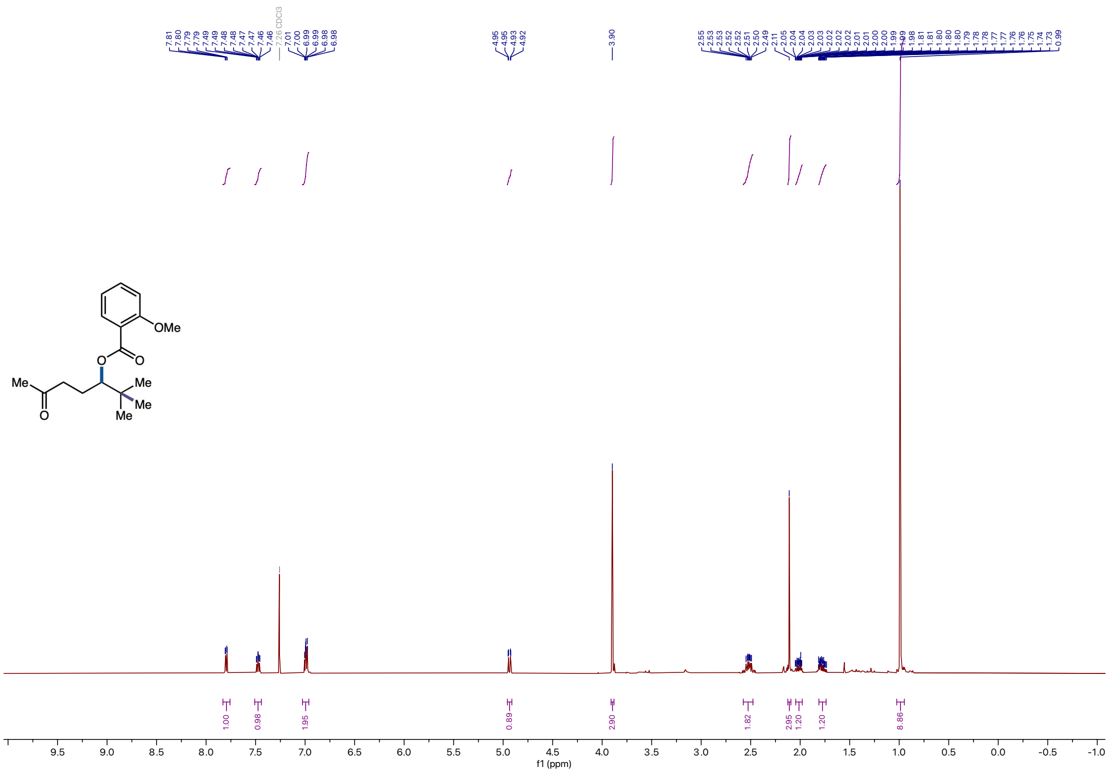

25, ^{13}C NMR, 126 MHz, $CDCl_3$

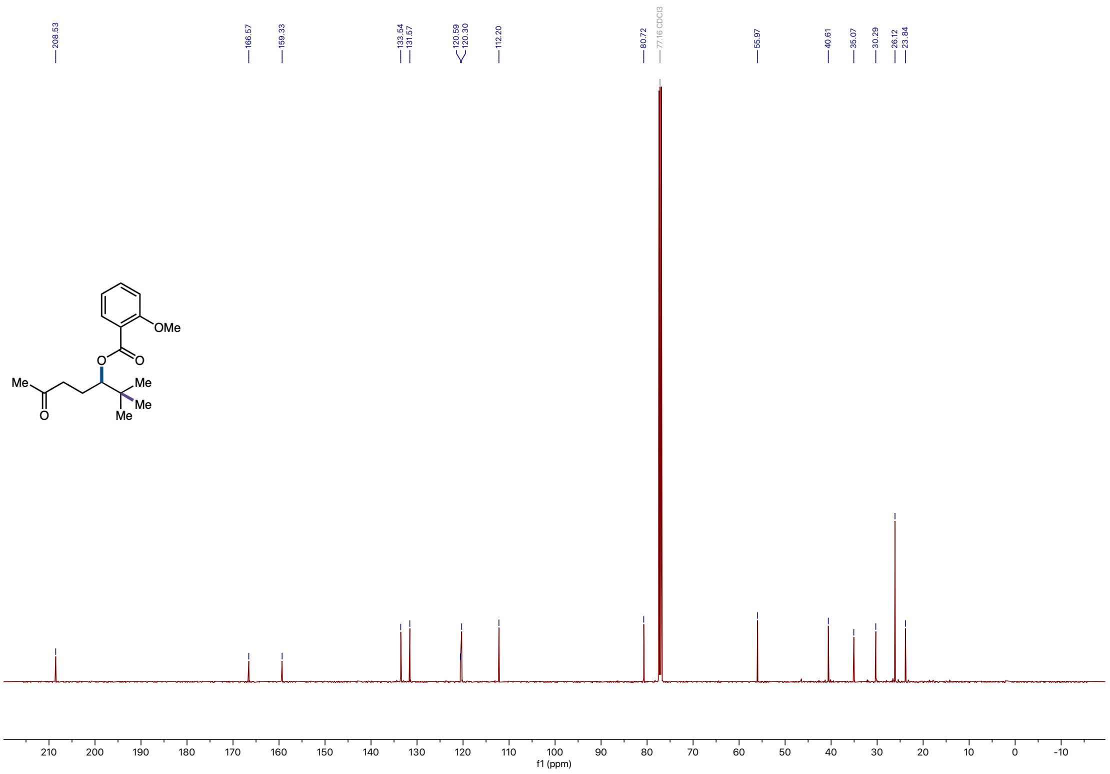

26, ^{1}H NMR, 500 MHz, $CDCl_3$

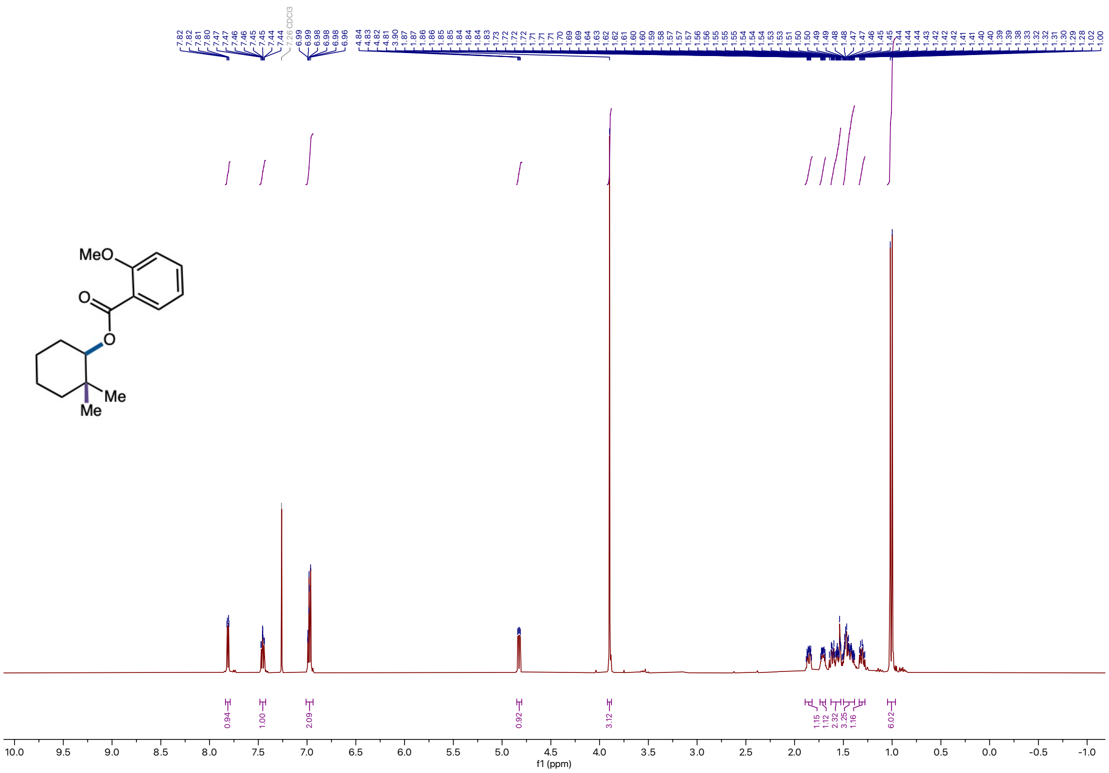

26, ^{13}C NMR, 126 MHz, $CDCl_3$

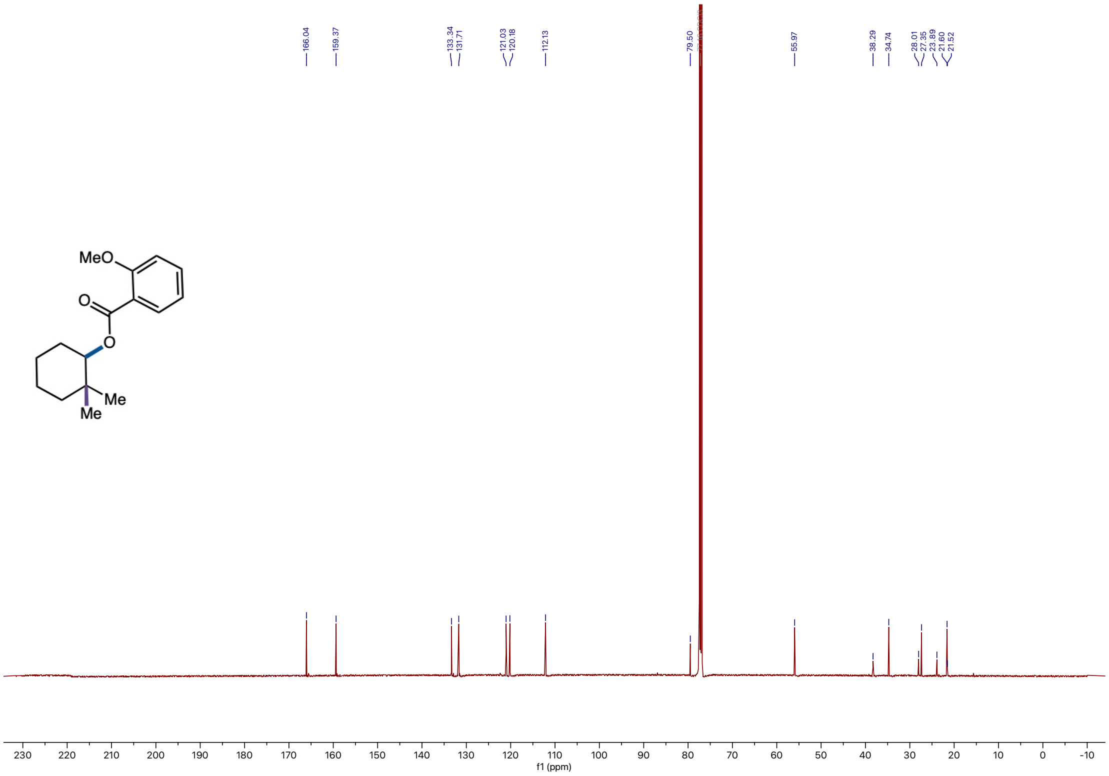

27, ^{1}H NMR, 500 MHz, $CDCl_3$

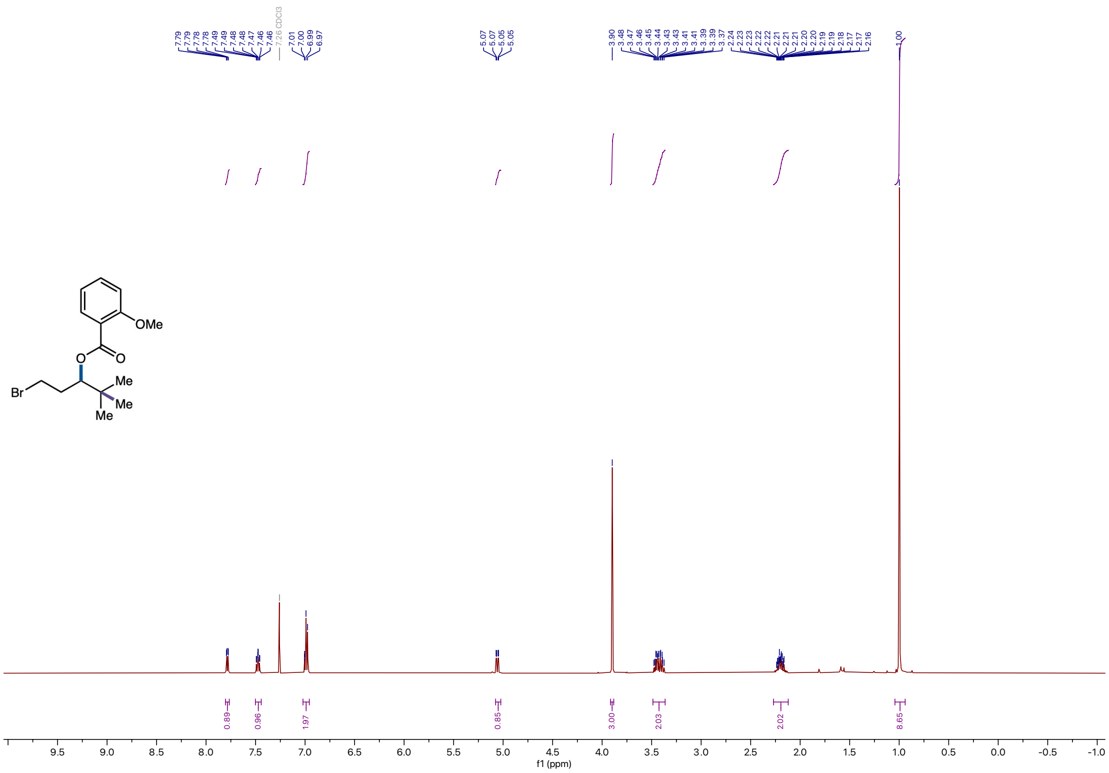

27, ^{13}C NMR, 126 MHz, $CDCl_3$

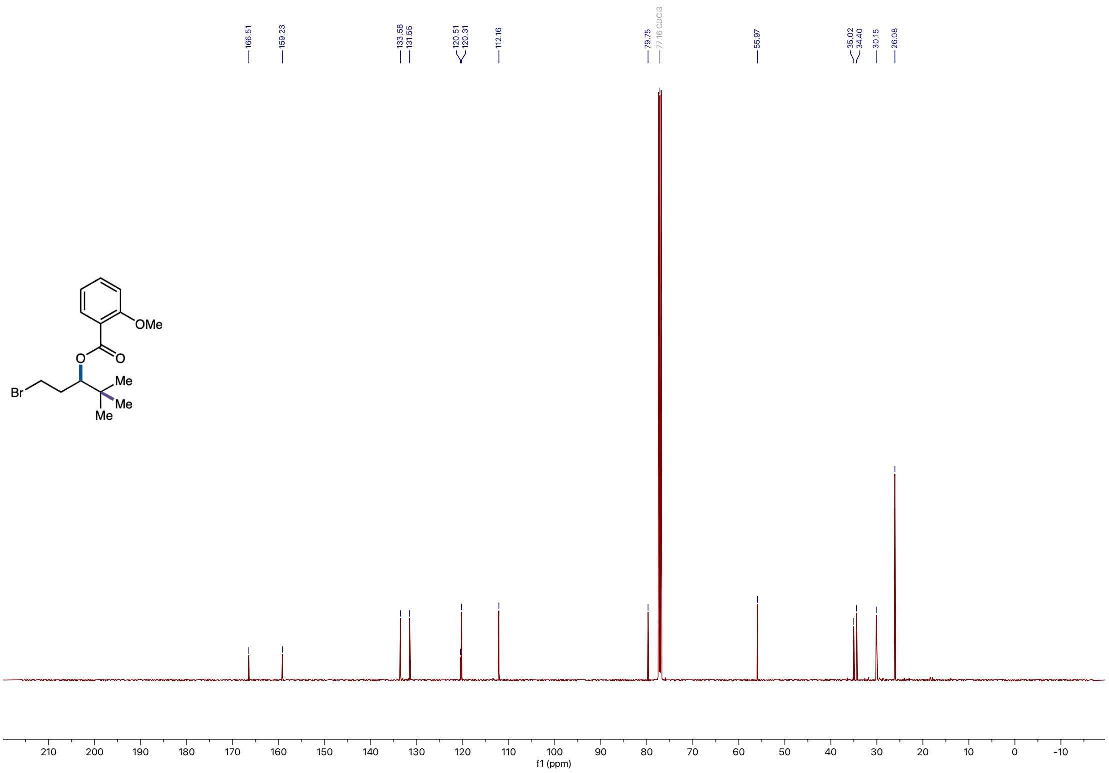

28, ^{1}H NMR, 500 MHz, $CDCl_3$

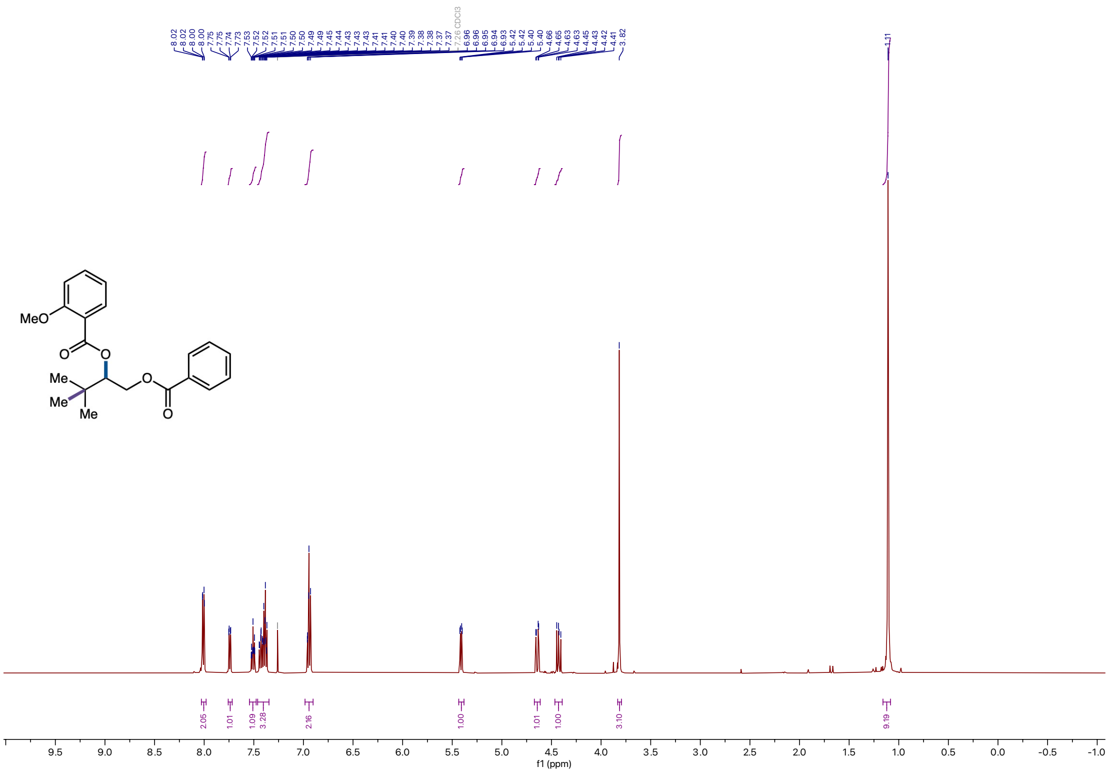

28, ^{13}C NMR, 126 MHz, $CDCl_3$

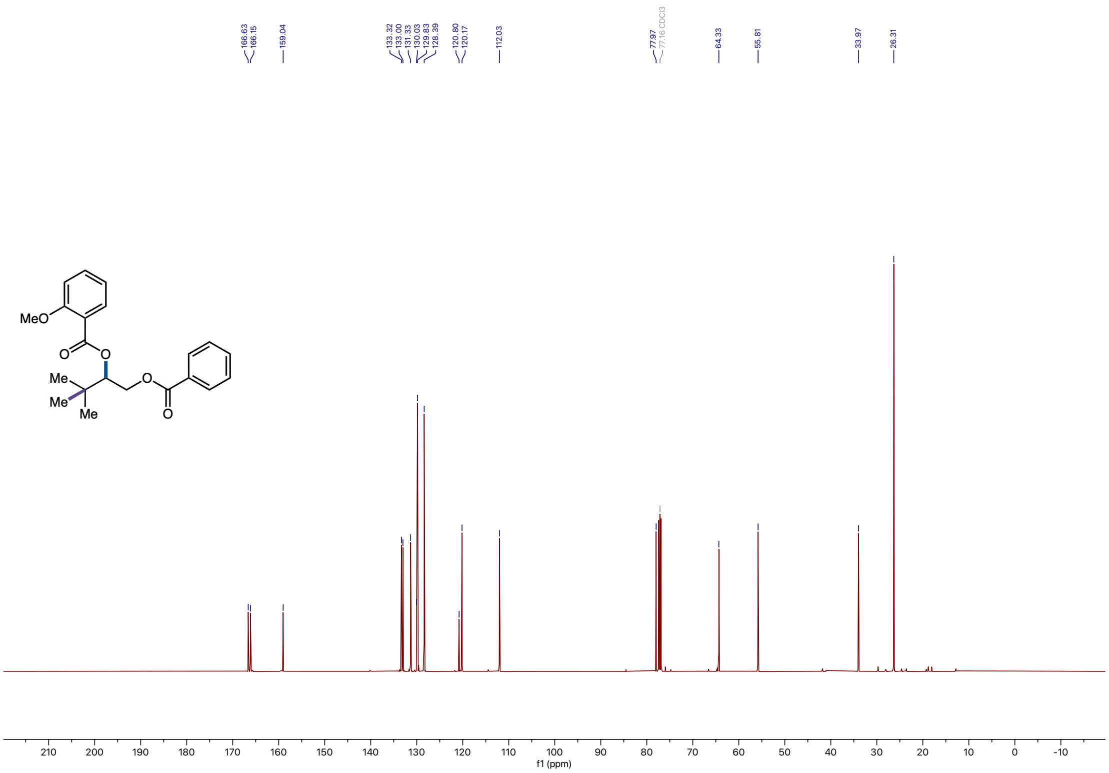

29-1 (diastereomer 1), ^{1}H NMR, 500 MHz, $CDCl_3$

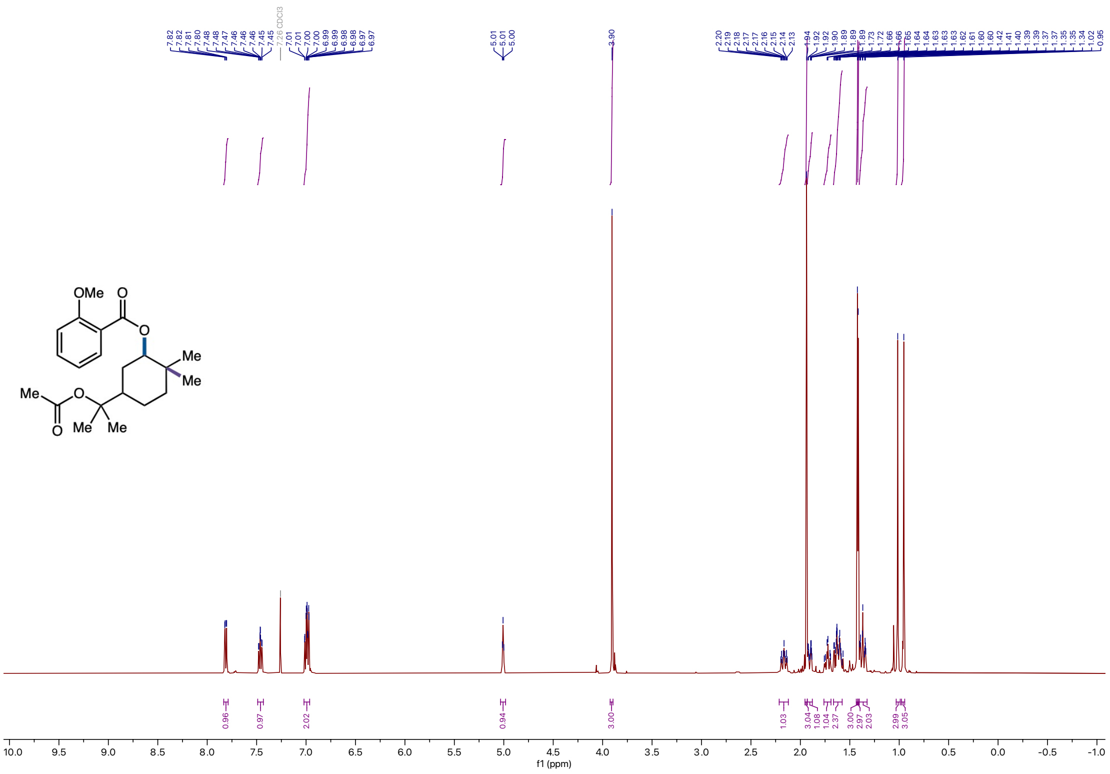

29-1 (diastereomer 1), ^{13}C NMR, 126 MHz, $CDCl_3$

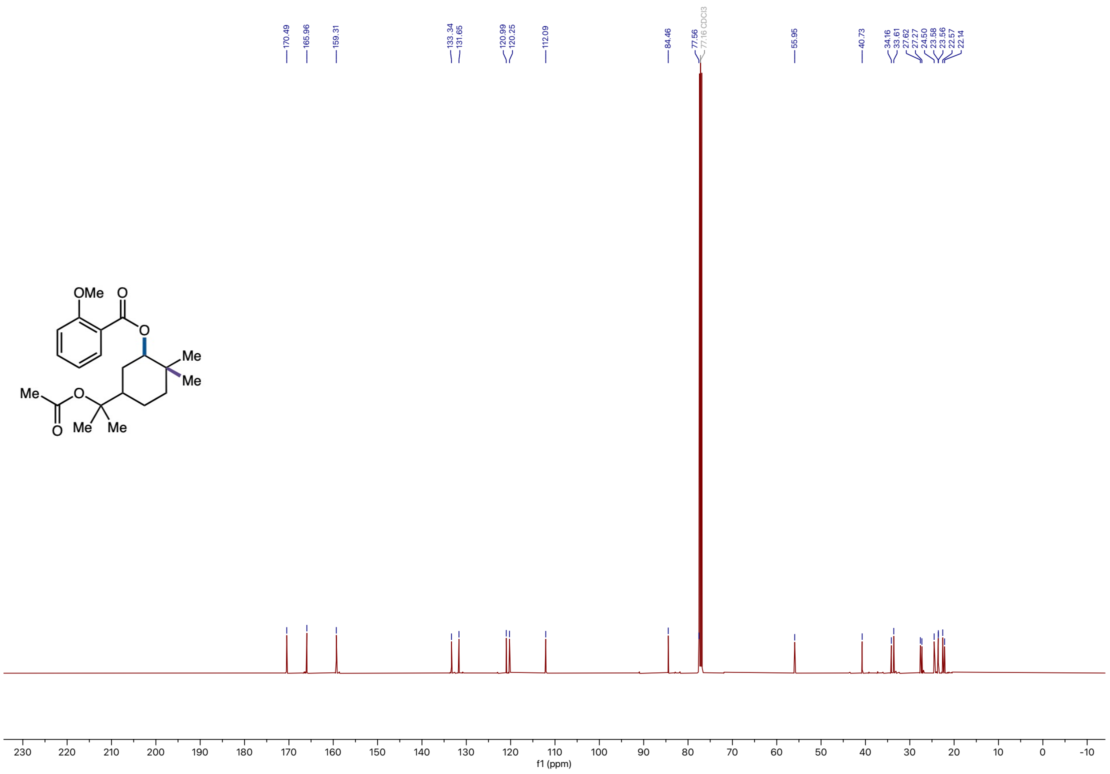

29-2 (diastereomer 2), ^{1}H NMR, 500 MHz, $CDCl_3$

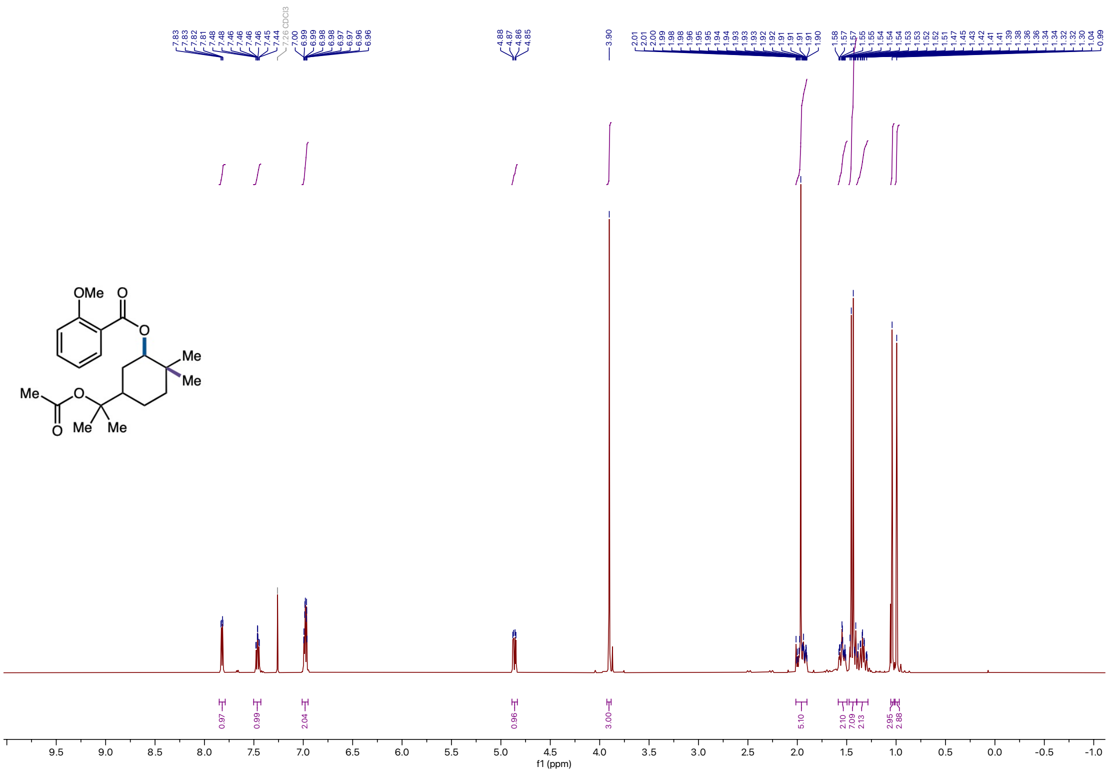

29-2 (diastereomer 2), ^{13}C NMR, 126 MHz, $CDCl_3$

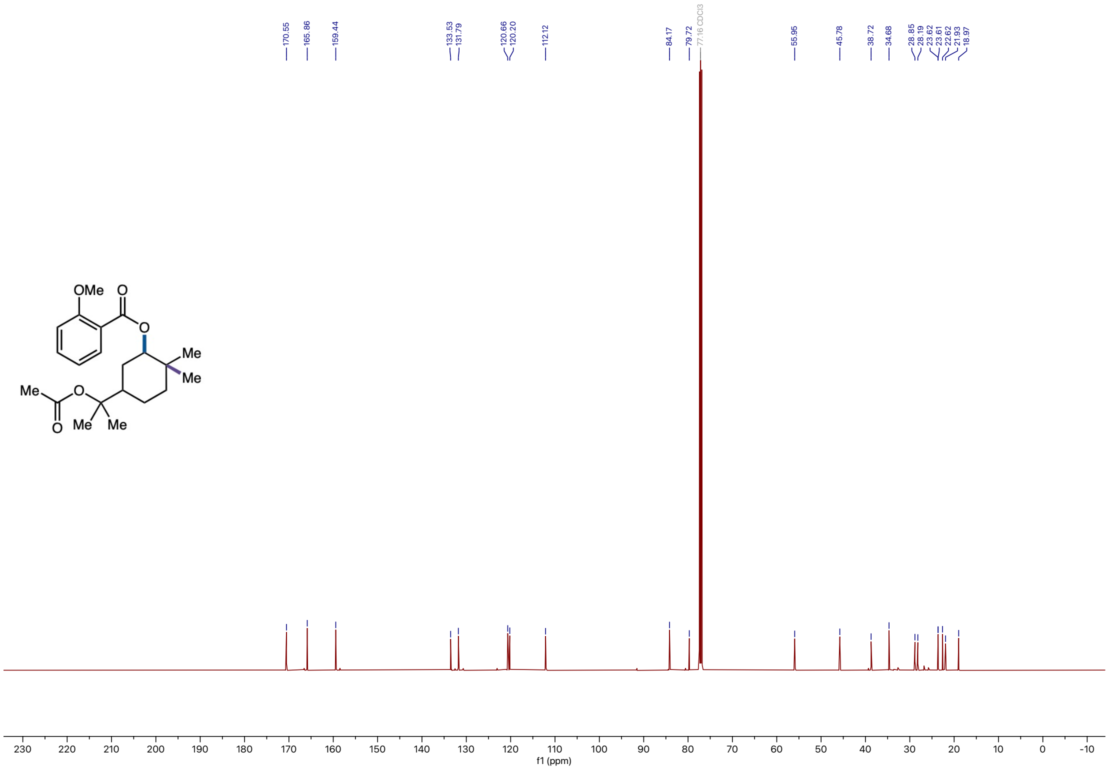

30, ^{1}H NMR, 500 MHz, $CDCl_3$

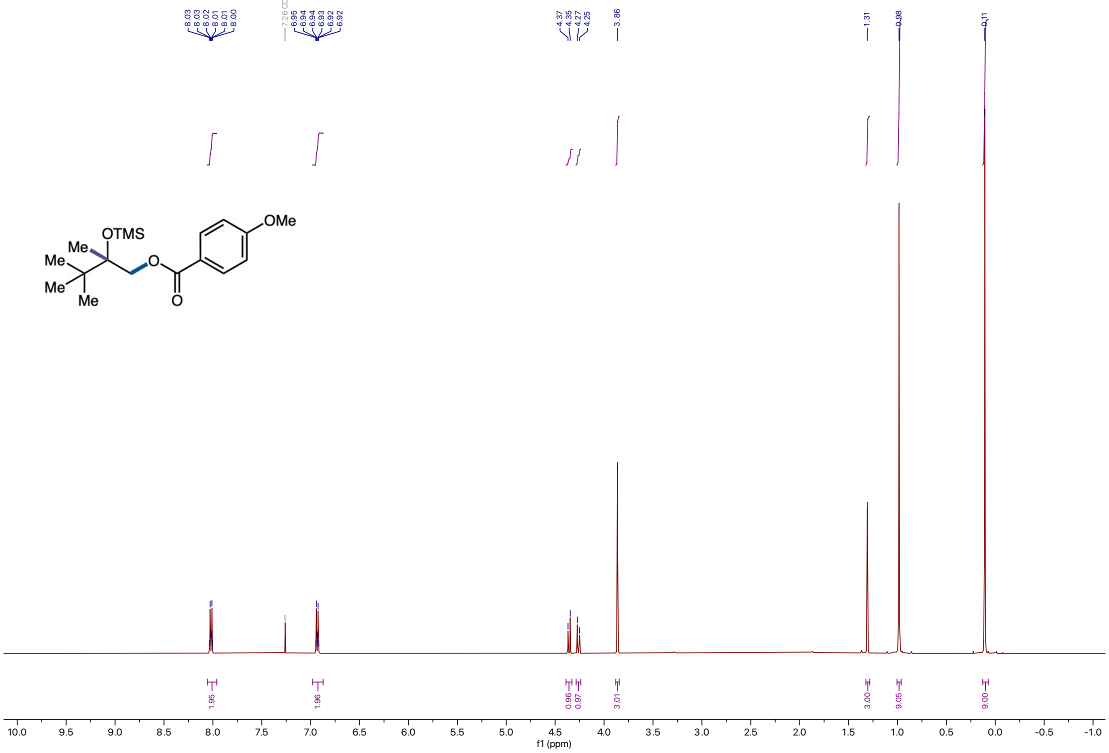

30, ^{13}C NMR, 126 MHz, $CDCl_3$

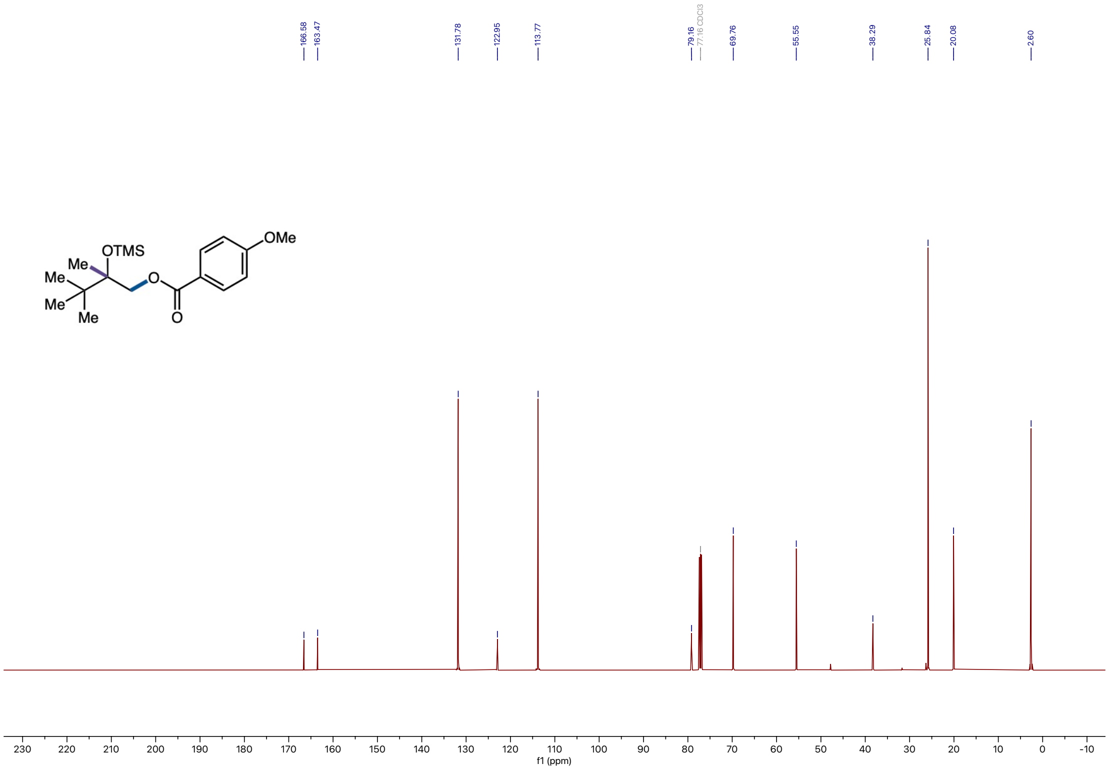

31, ^{1}H NMR, 500 MHz, $CDCl_3$

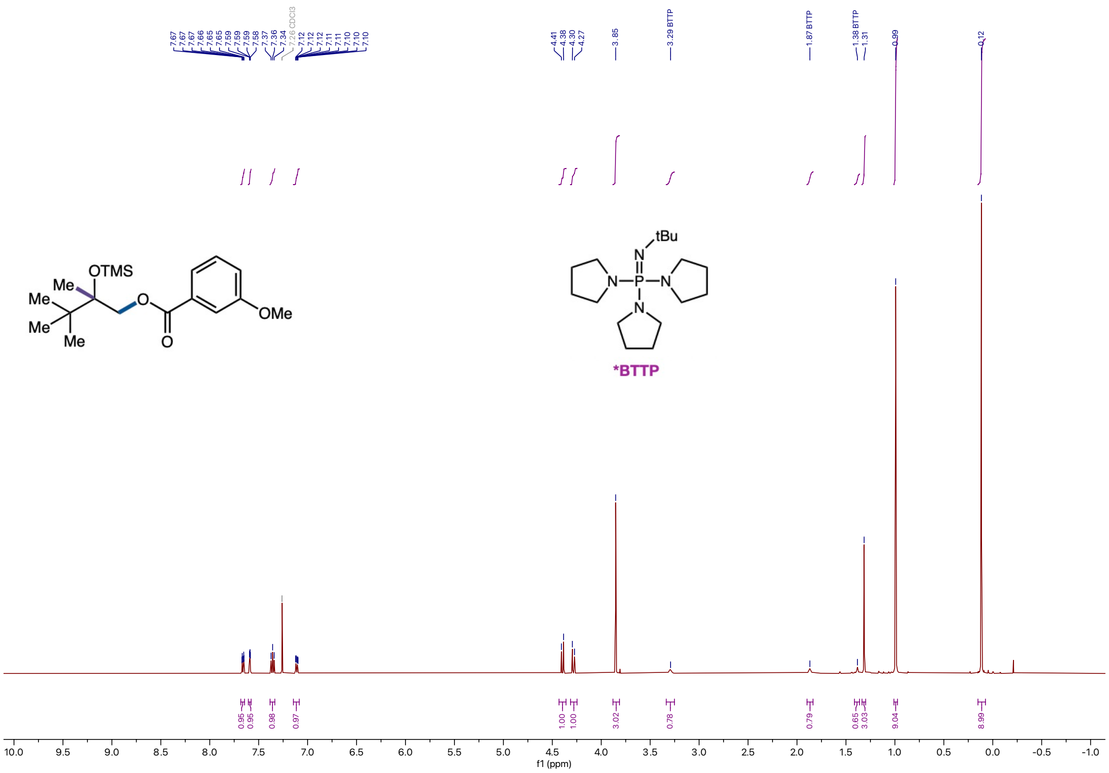

31, ^{13}C NMR, 126 MHz, $CDCl_3$

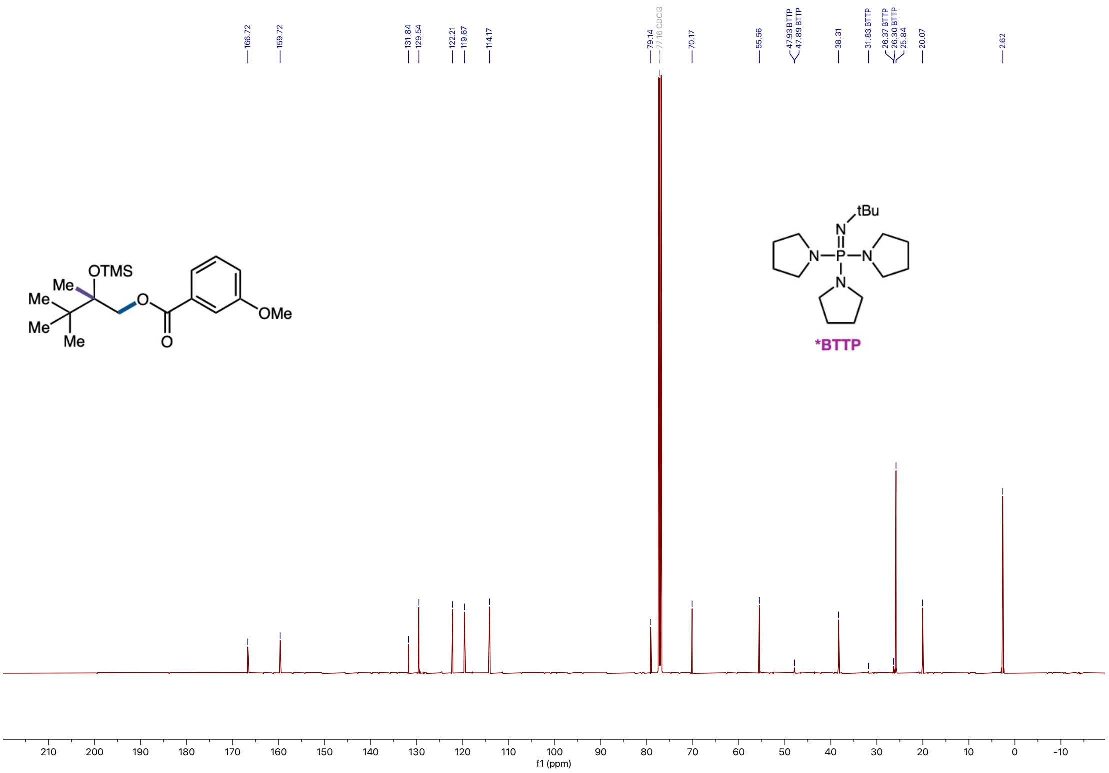

32, ^{1}H NMR, 500 MHz, $CDCl_3$

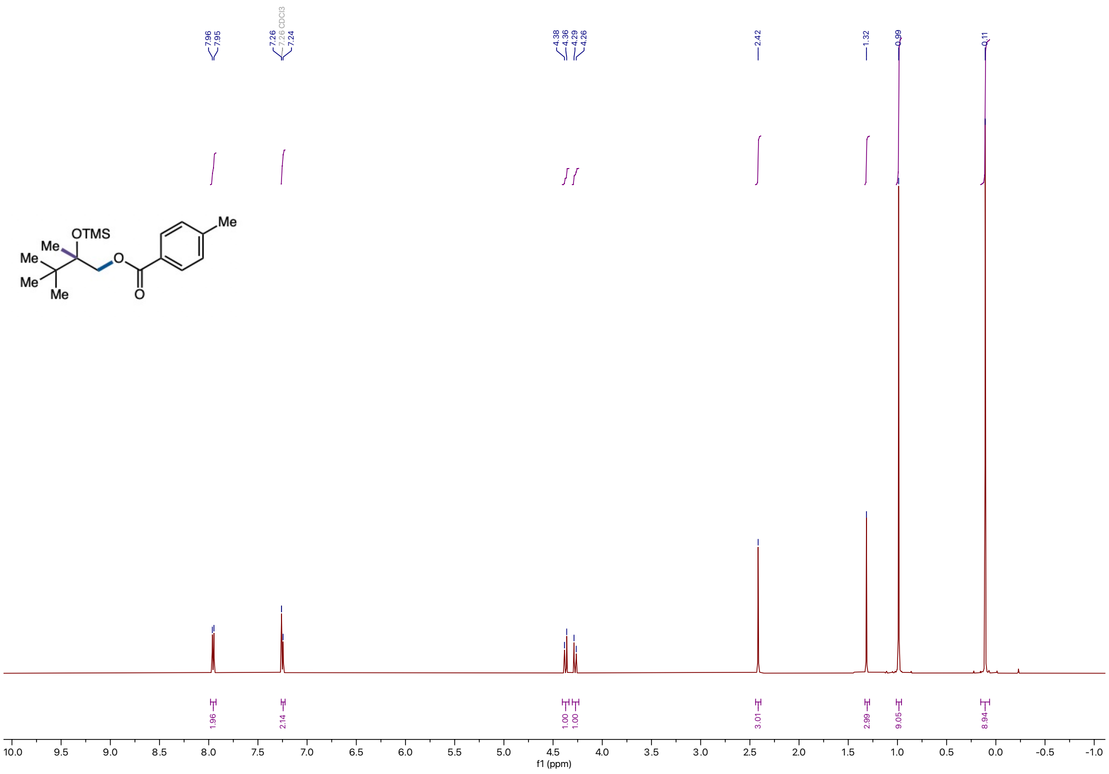

32, ^{13}C NMR, 126 MHz, $CDCl_3$

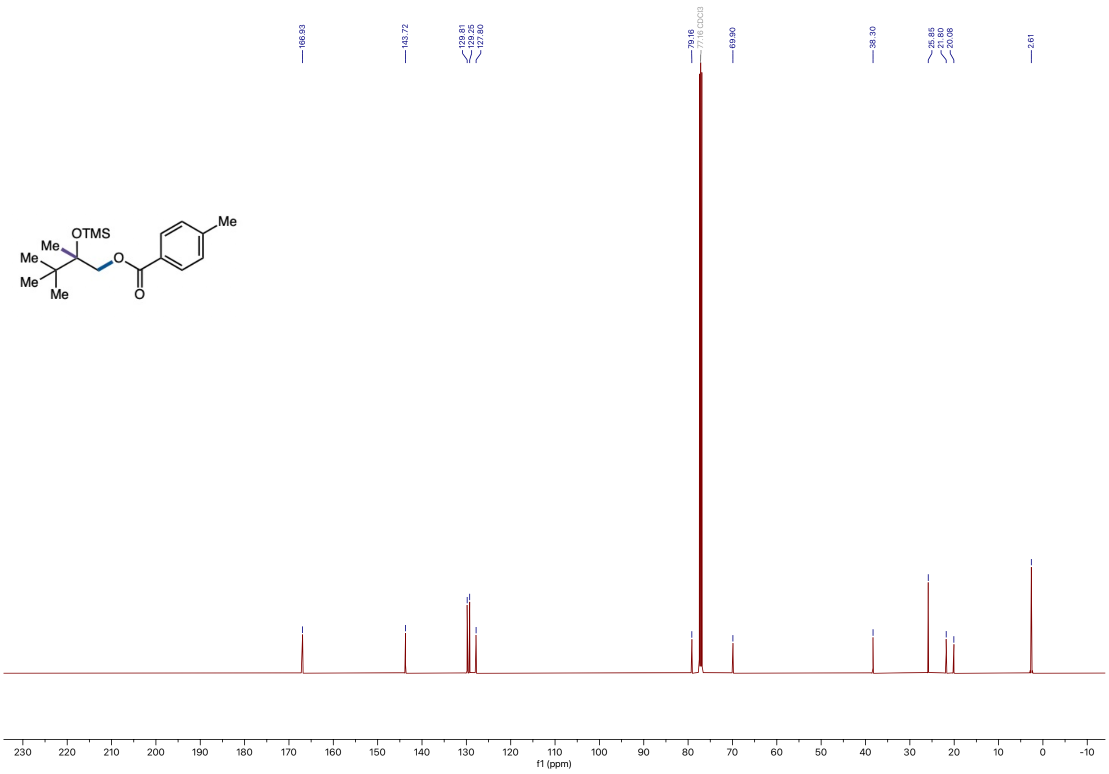

33, ^{1}H NMR, 500 MHz, $CDCl_3$

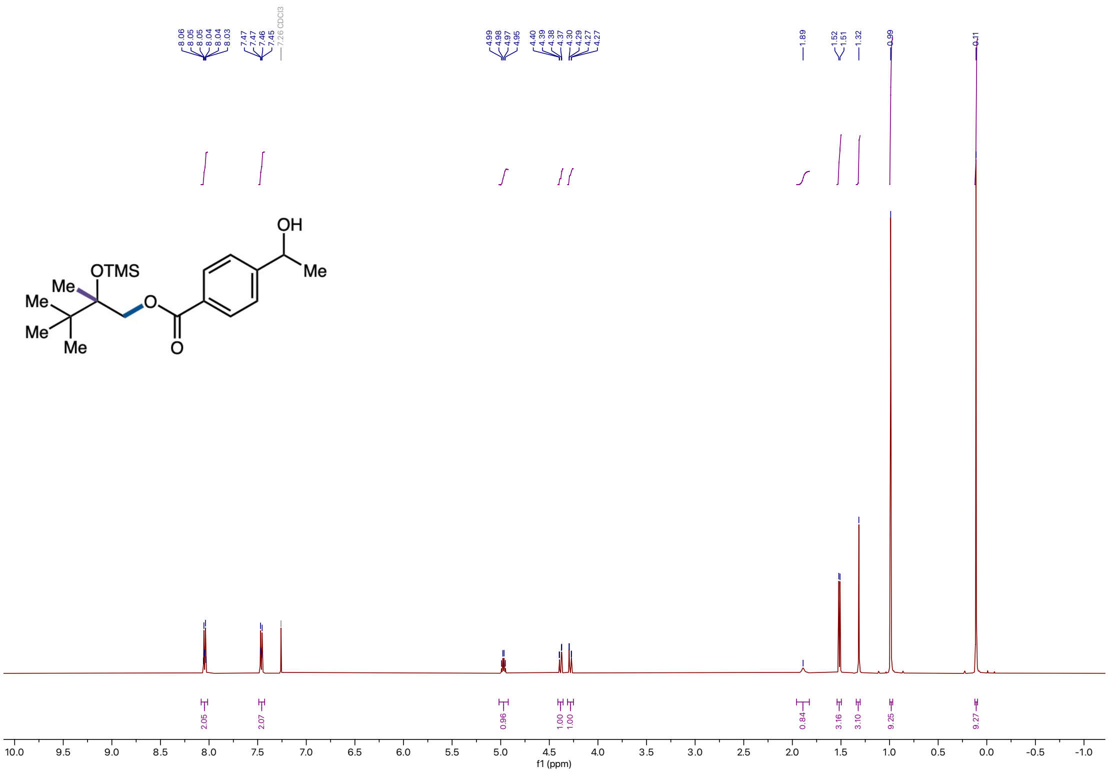

33, ^{13}C NMR, 126 MHz, $CDCl_3$

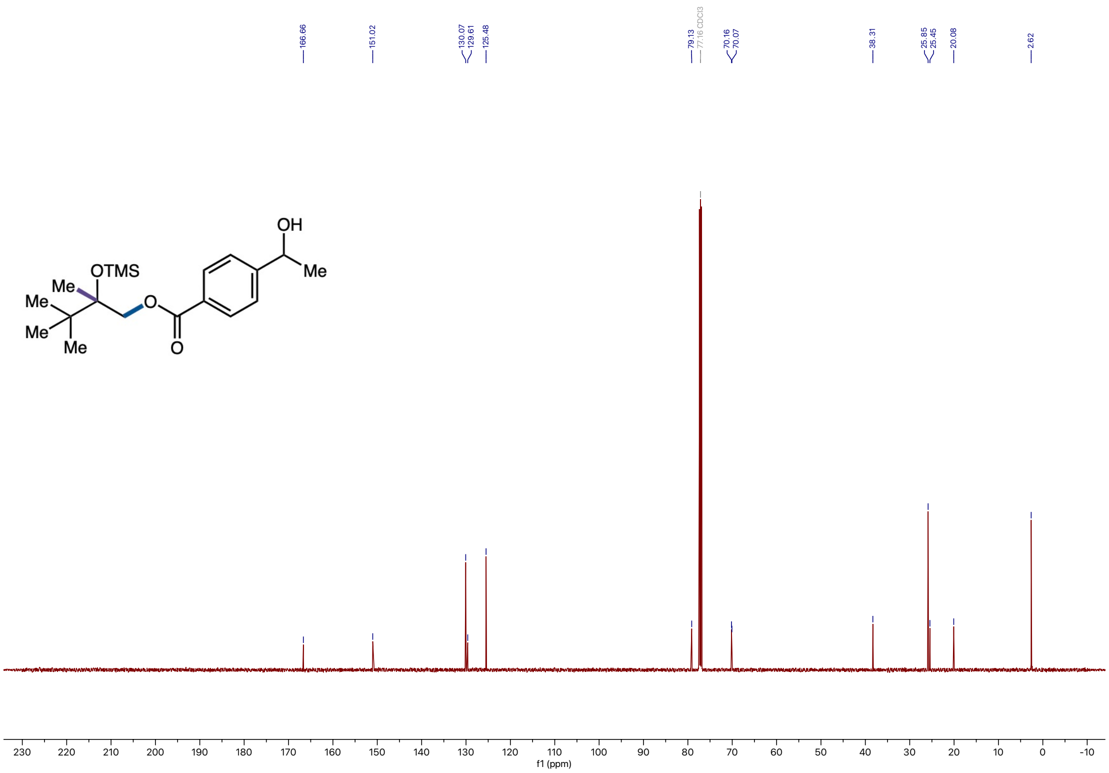

34, ¹H NMR, 500 MHz, $CDCl_3$

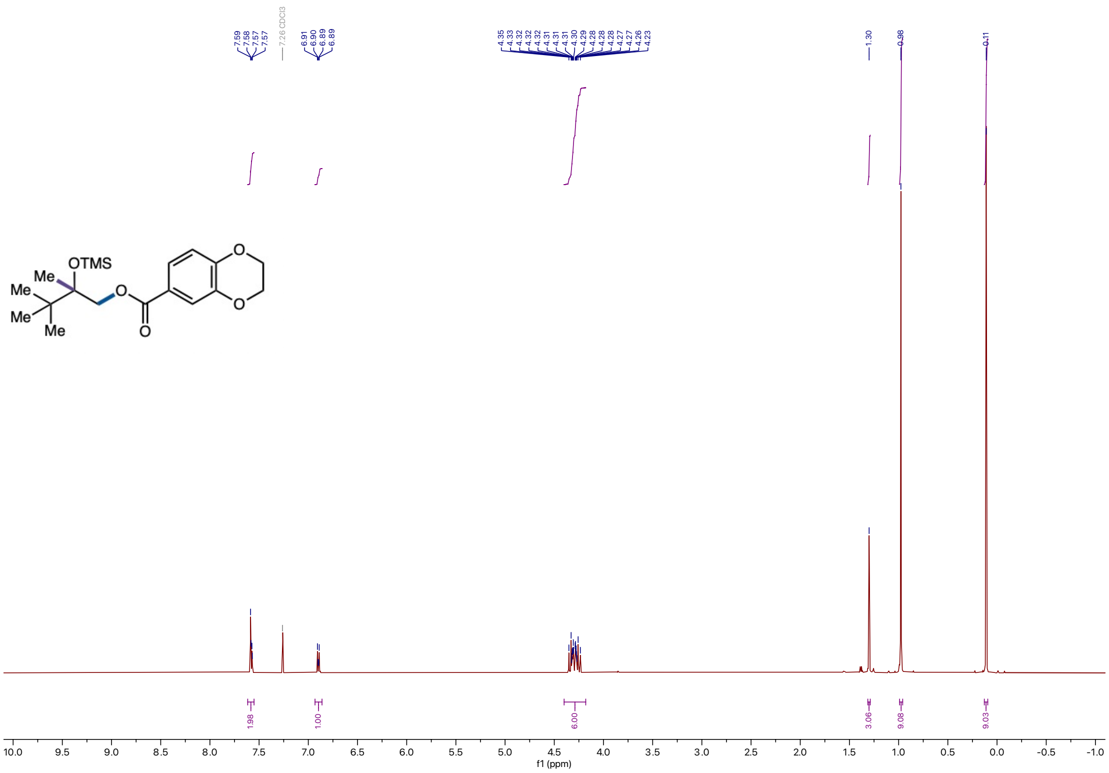

34, ¹³C NMR, 126 MHz, $CDCl_3$

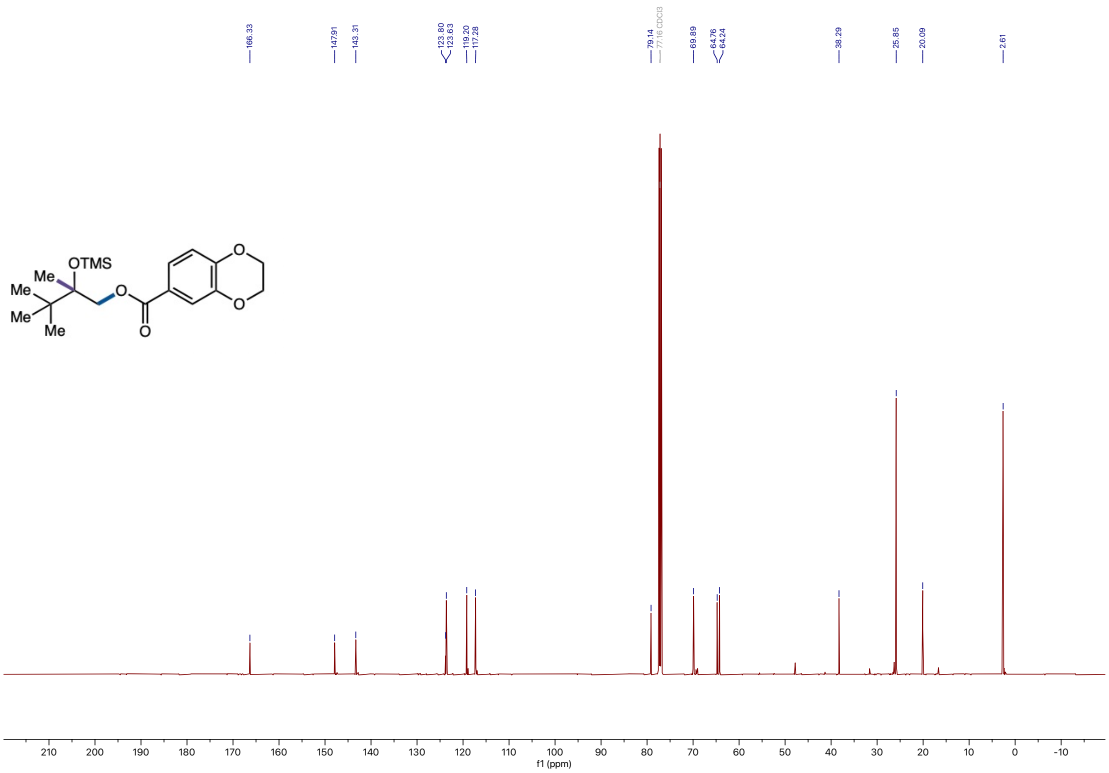

35, ^{1}H NMR, 500 MHz, $CDCl_3$

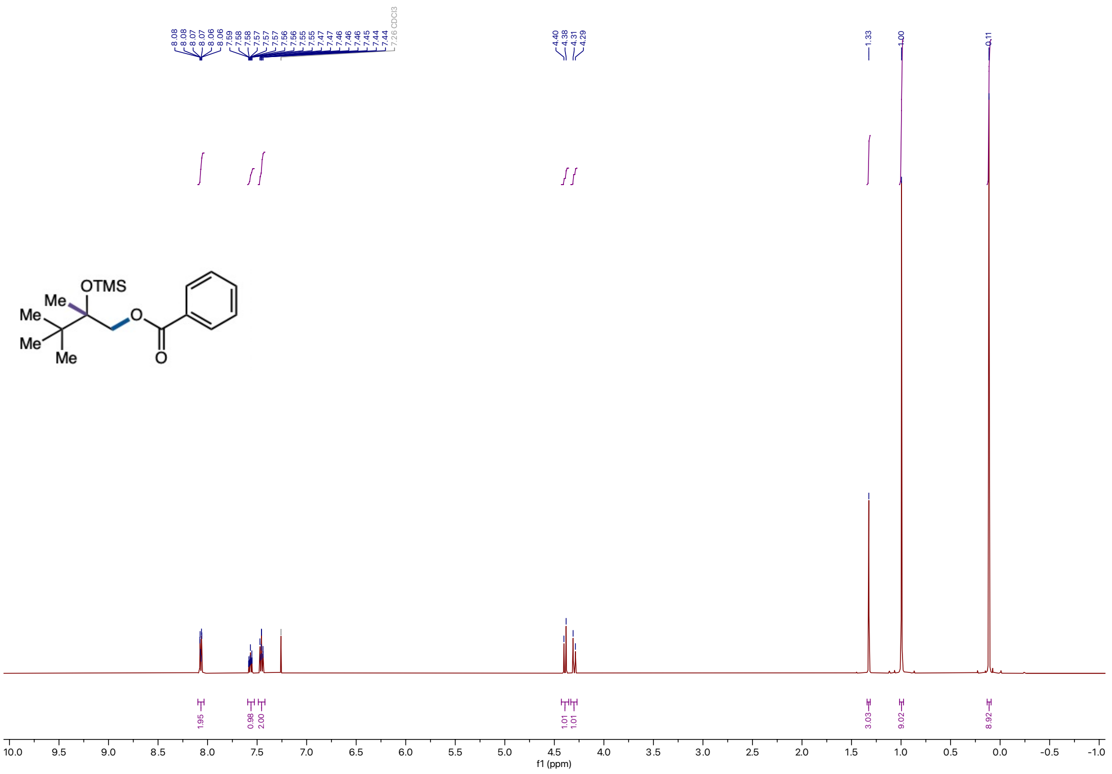

35, ^{13}C NMR, 126 MHz, $CDCl_3$

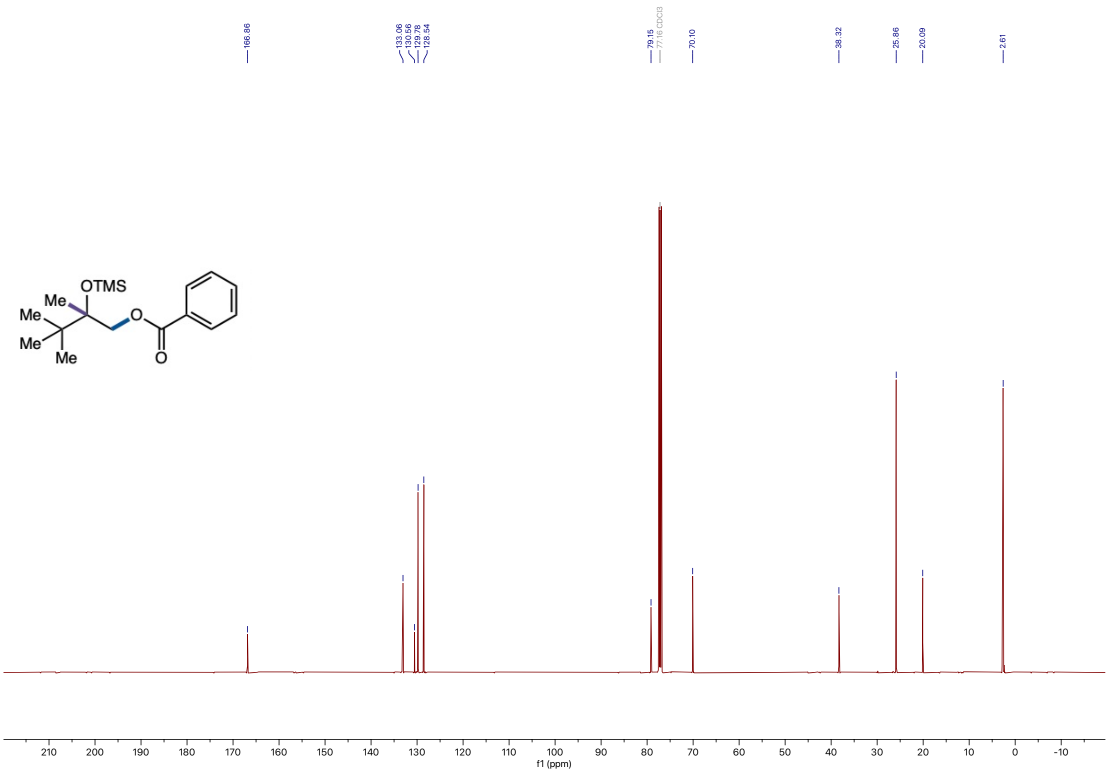

36, ^{1}H NMR, 500 MHz, $CDCl_3$

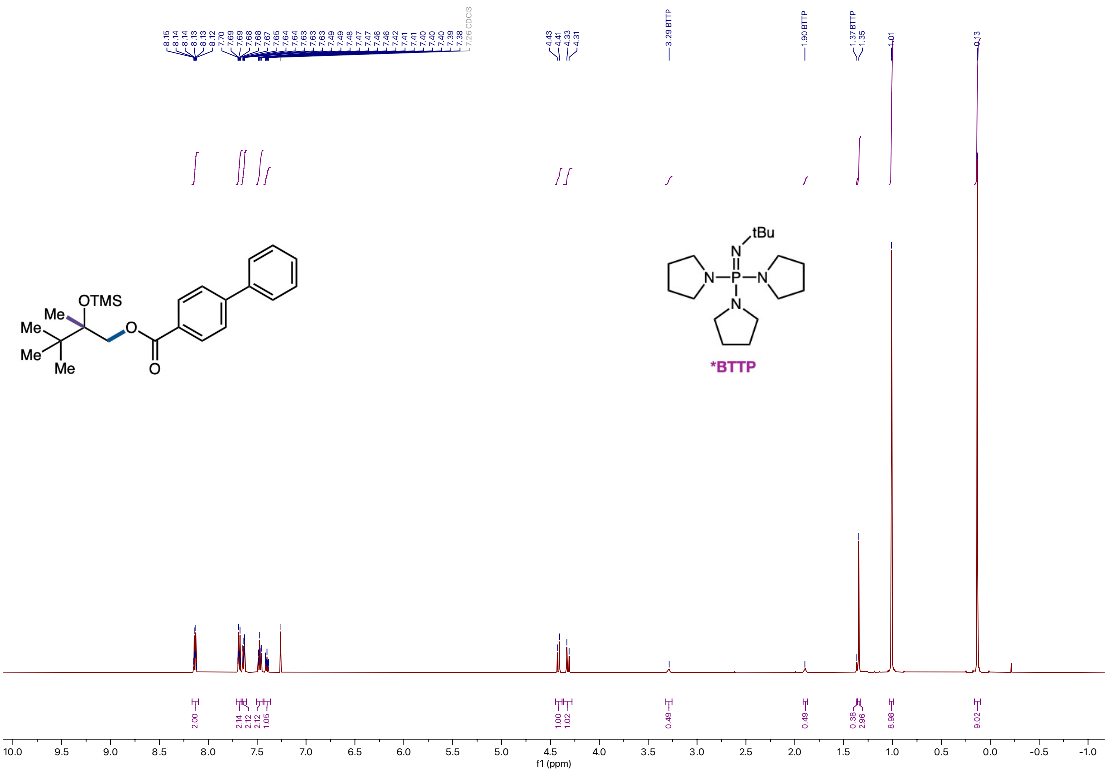

36, ^{13}C NMR, 126 MHz, $CDCl_3$

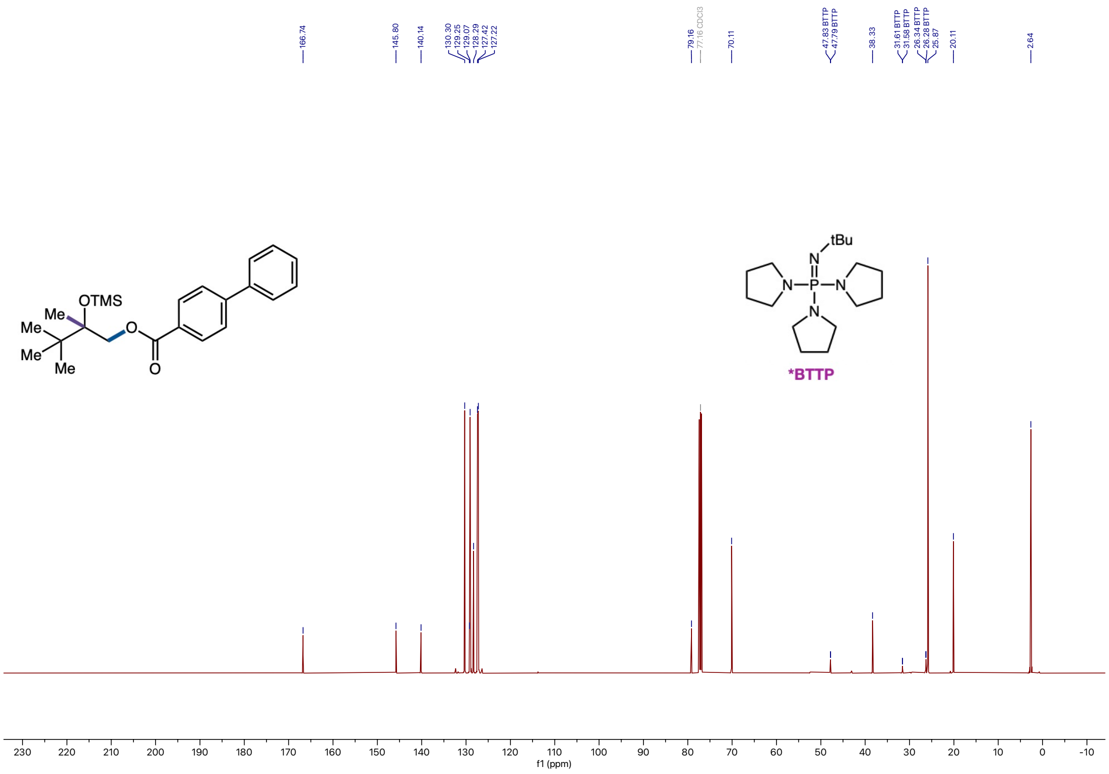

37, ^{1}H NMR, 500 MHz, $CDCl_3$

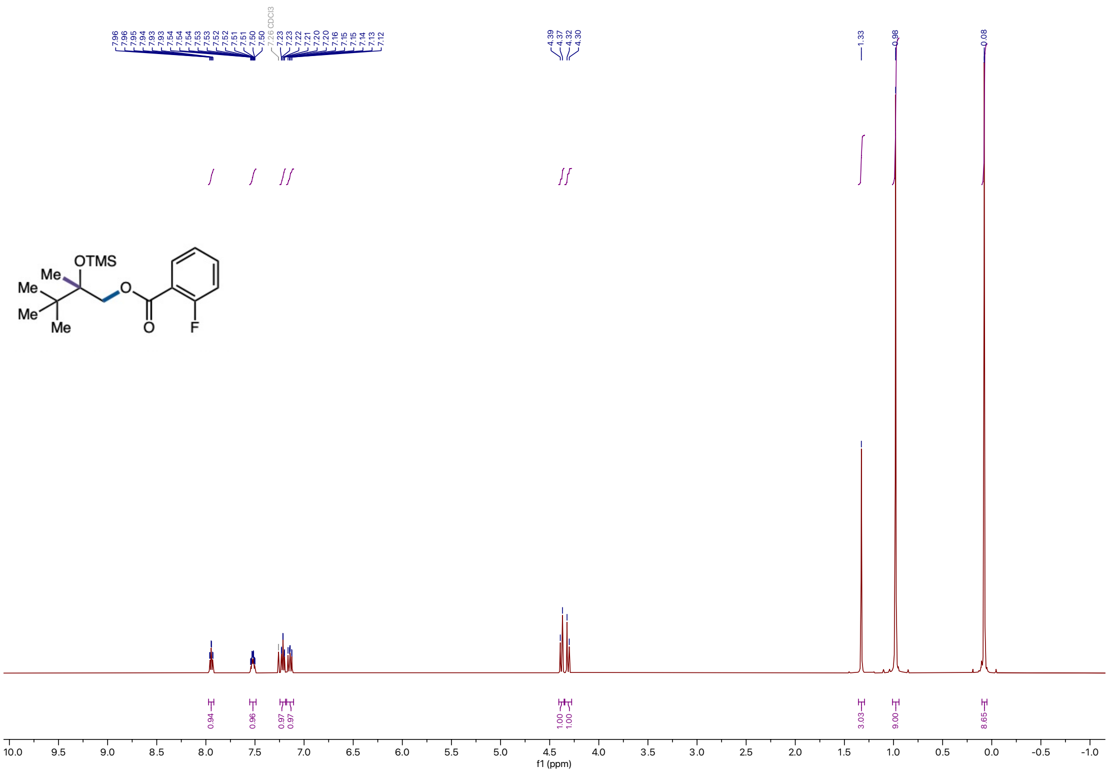

37, ^{13}C NMR, 126 MHz, $CDCl_3$

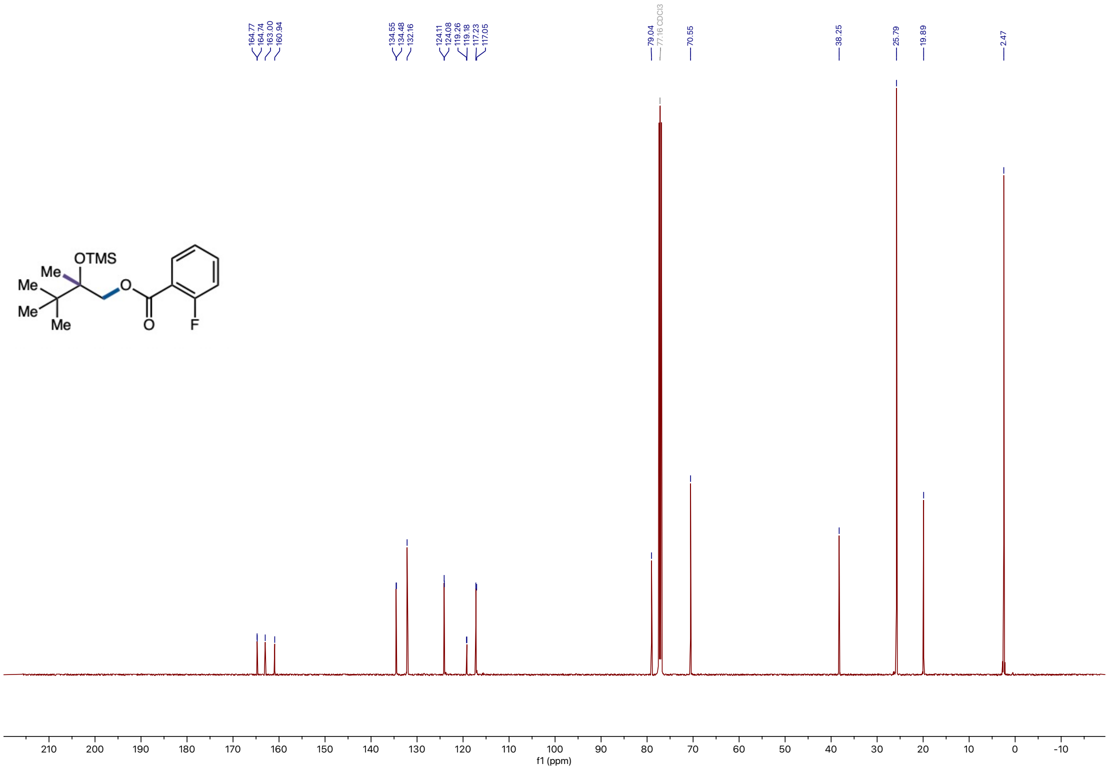

37, ^{19}F NMR, 376 MHz, $CDCl_3$

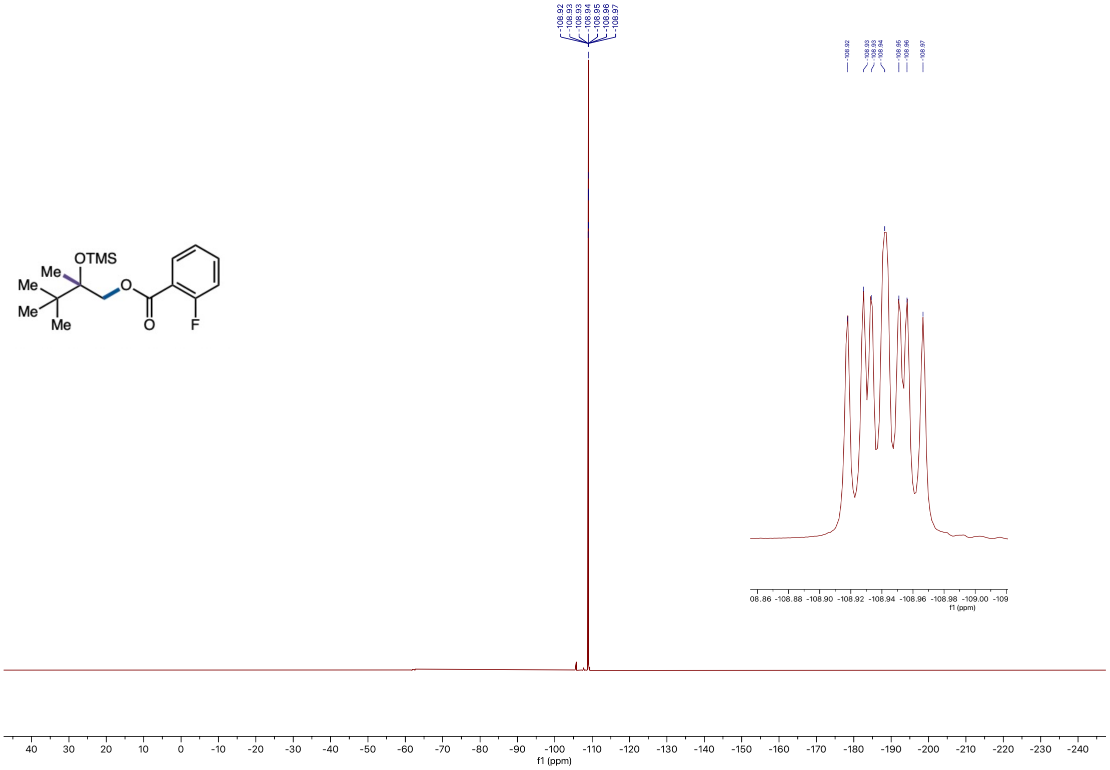

38, ^{1}H NMR, 500 MHz, CDCl$_3$

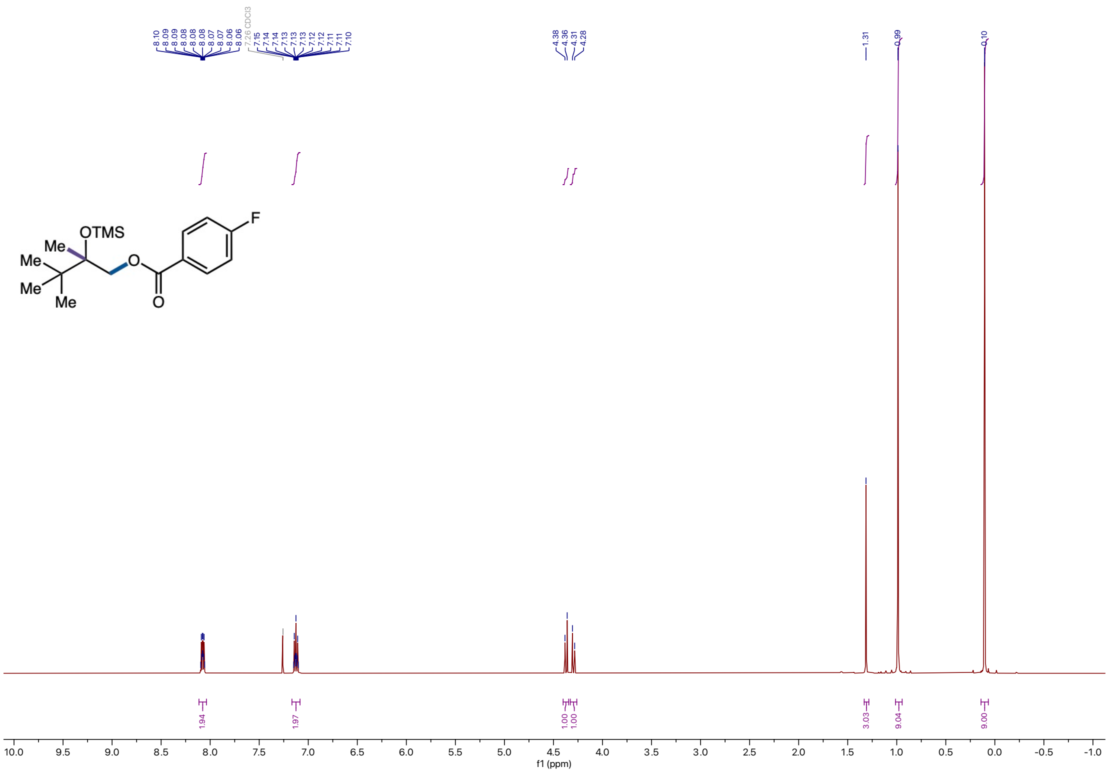

38, ^{13}C NMR, 126 MHz, CDCl$_3$

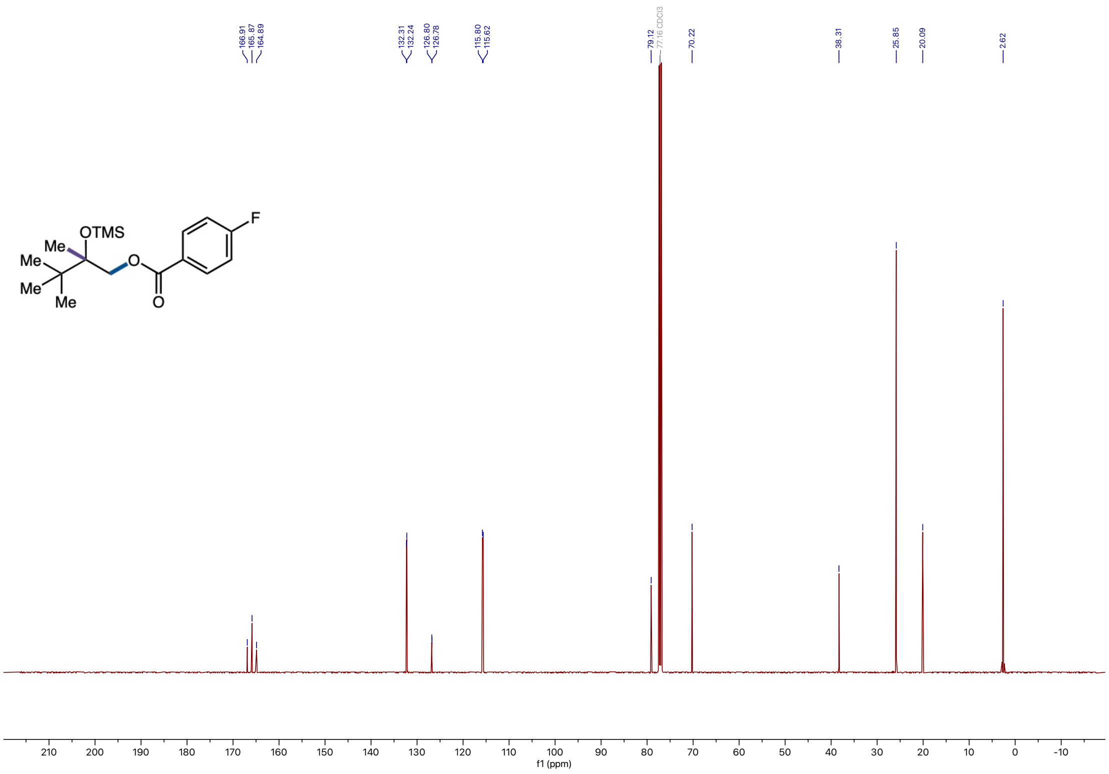

38, ^{19}F NMR, 376 MHz, $CDCl_3$

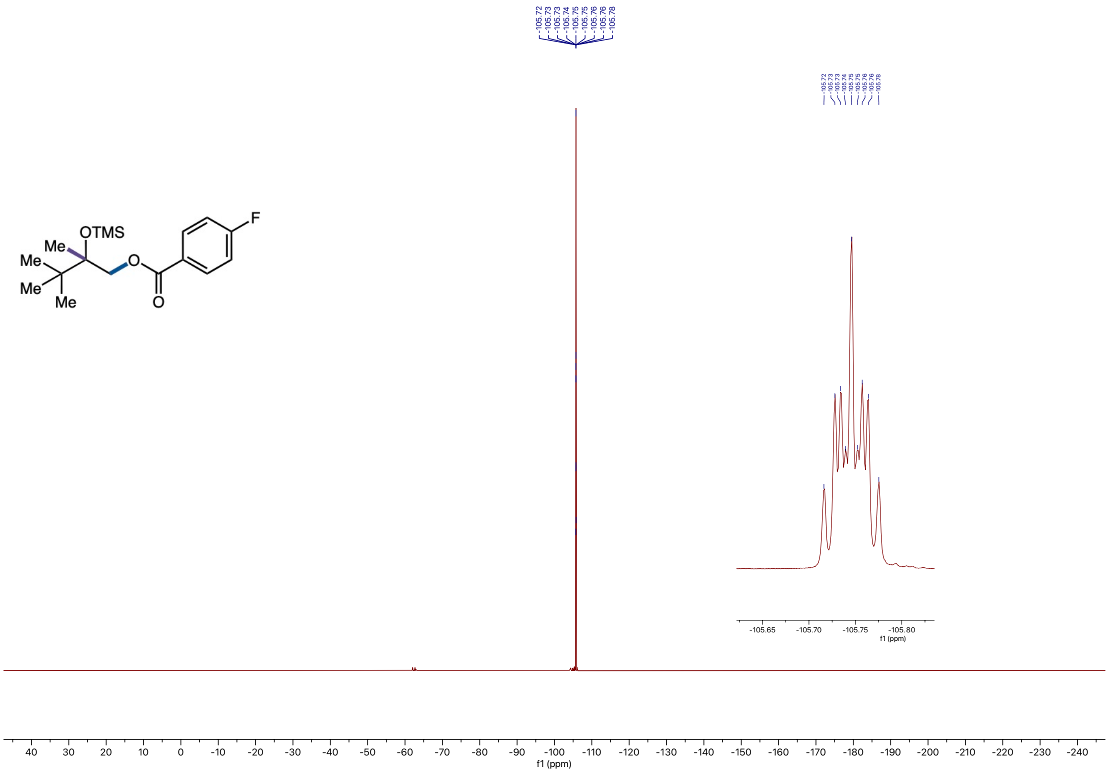

39, ^{1}H NMR, 500 MHz, $CDCl_3$

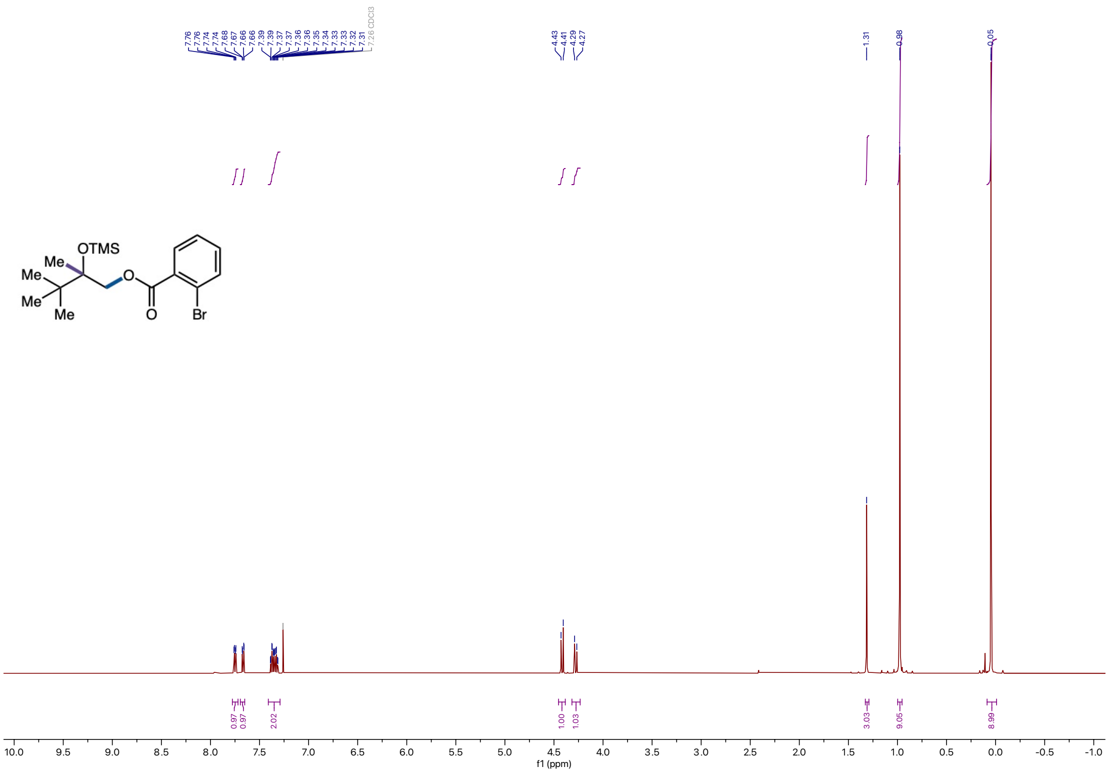

39, ^{13}C NMR, 126 MHz, $CDCl_3$

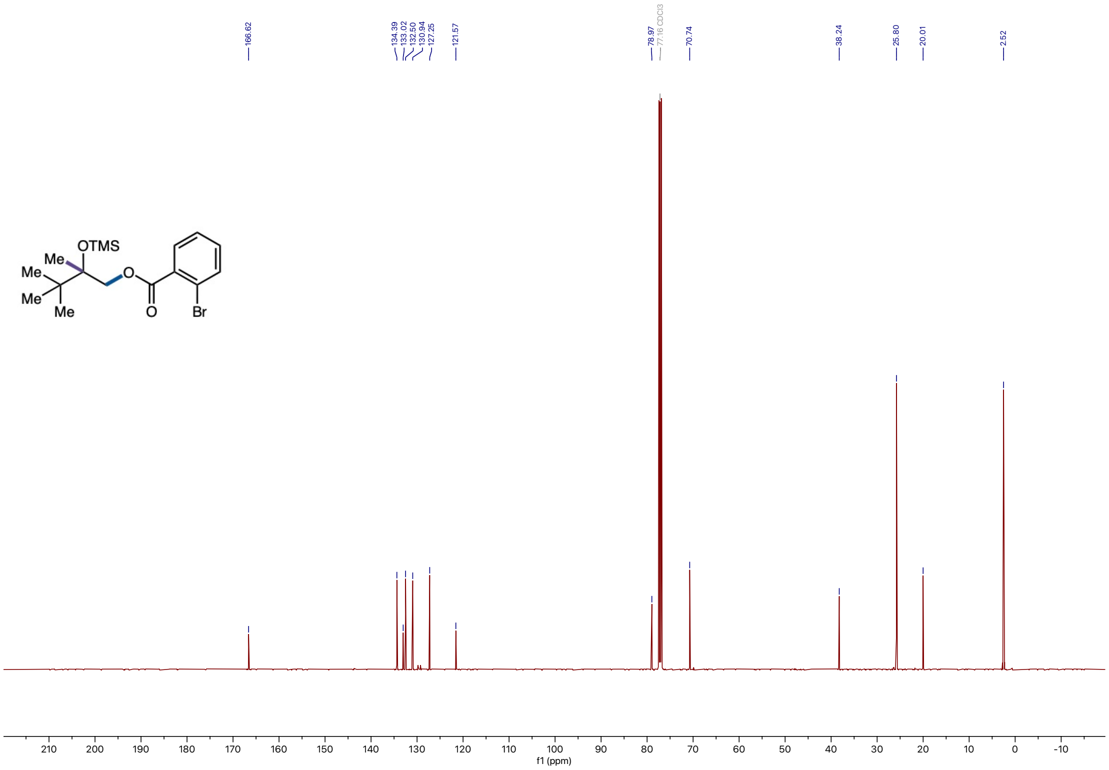

40, ^{1}H NMR, 500 MHz, $CDCl_3$

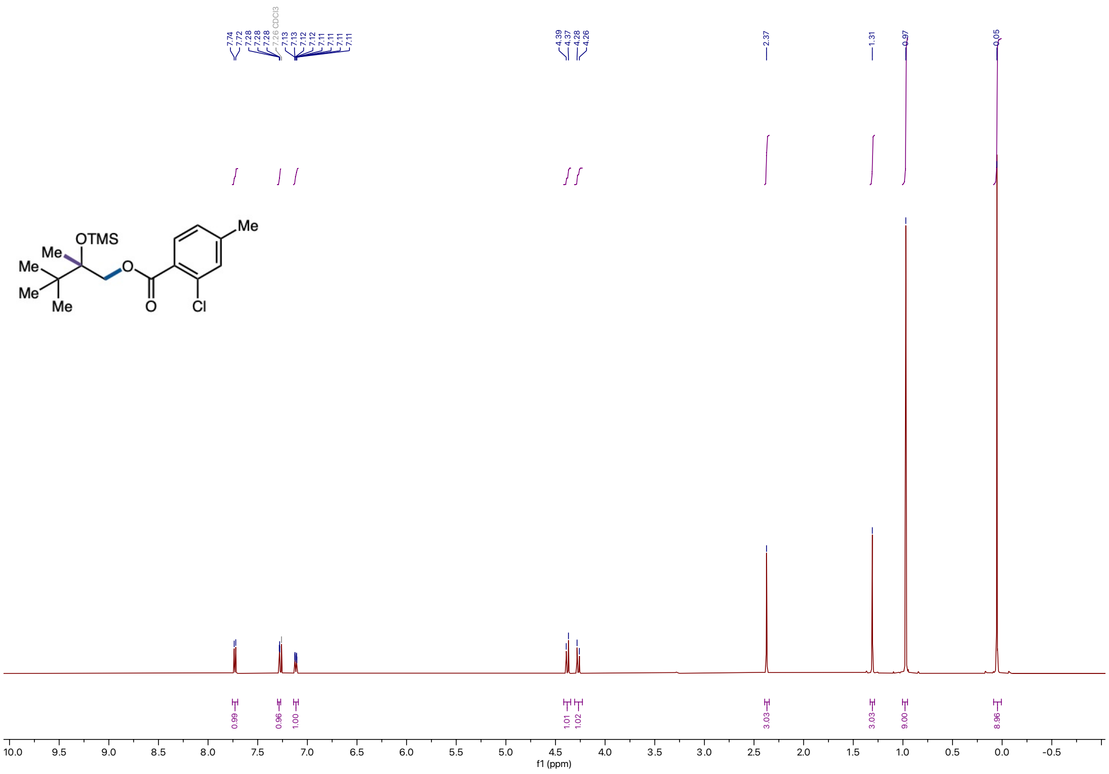

40, ^{13}C NMR, 126 MHz, $CDCl_3$

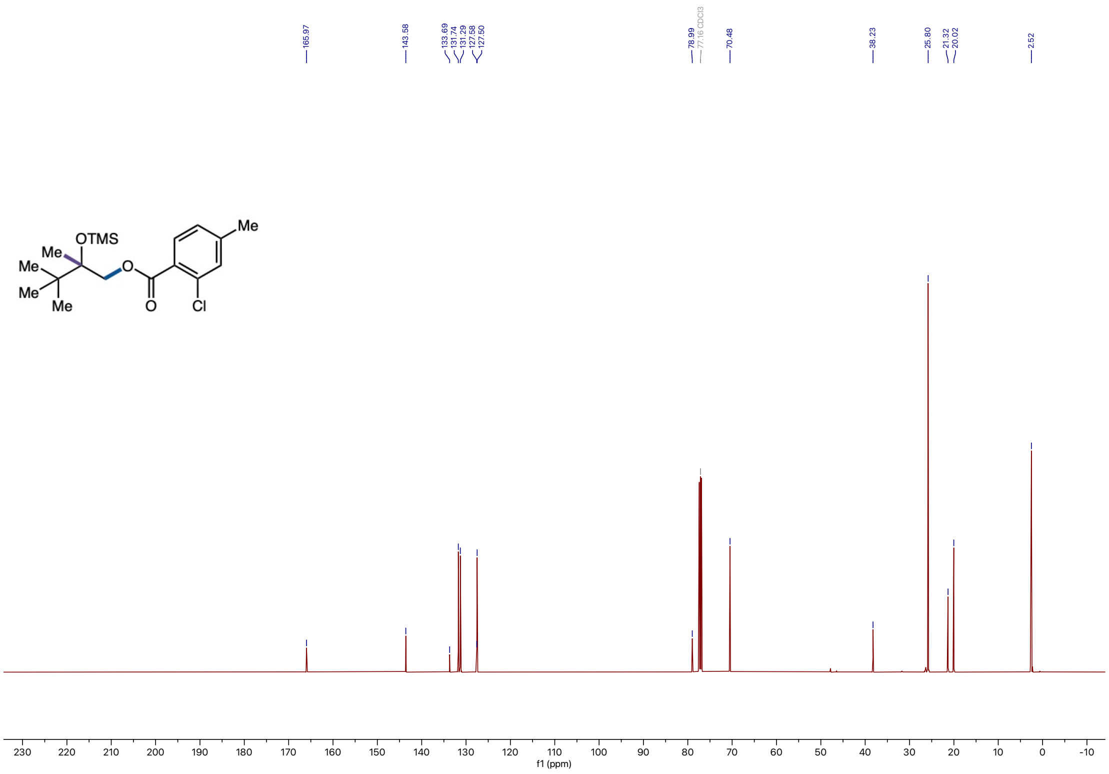

41, ^{1}H NMR, 500 MHz, $CDCl_3$

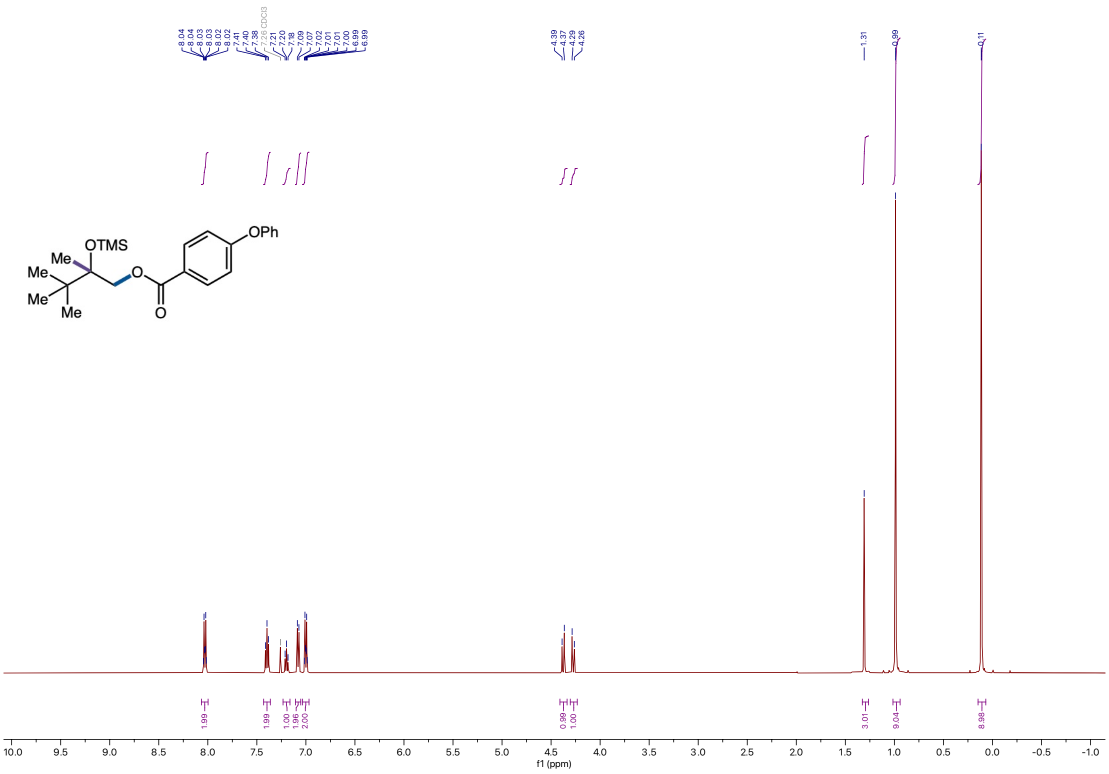

41, ^{13}C NMR, 126 MHz, $CDCl_3$

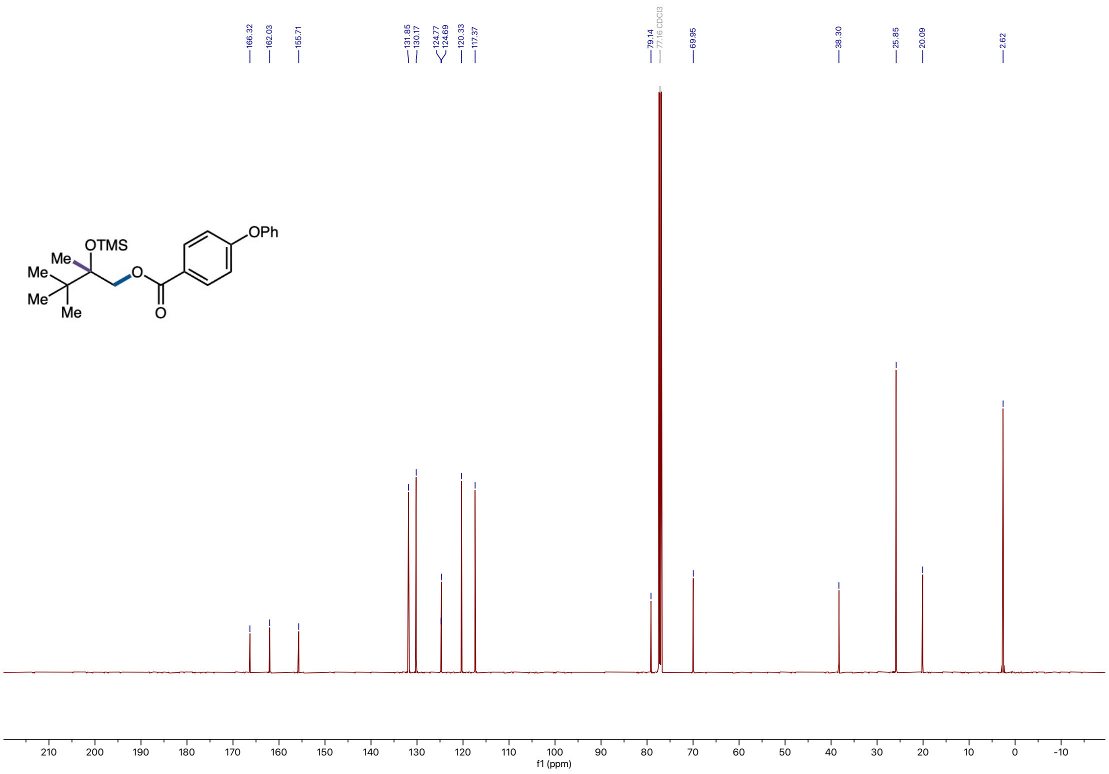

42, ^{1}H NMR, 500 MHz, $CDCl_3$

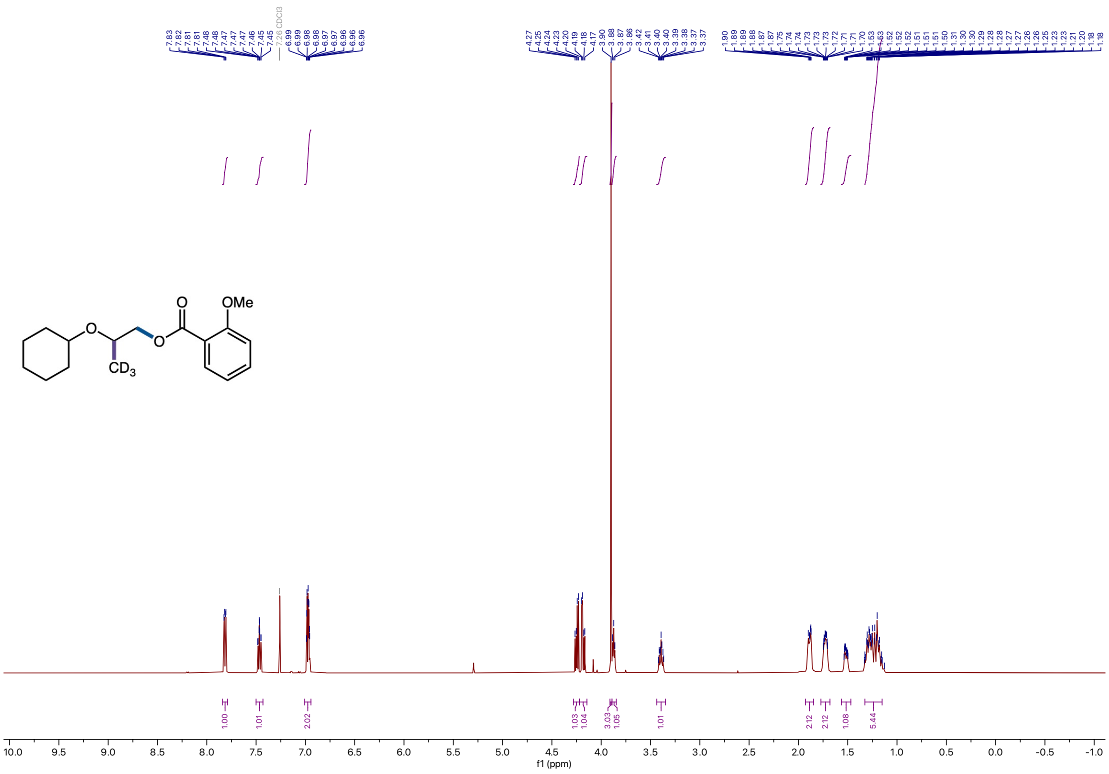

42, ^{13}C NMR, 126 MHz, $CDCl_3$

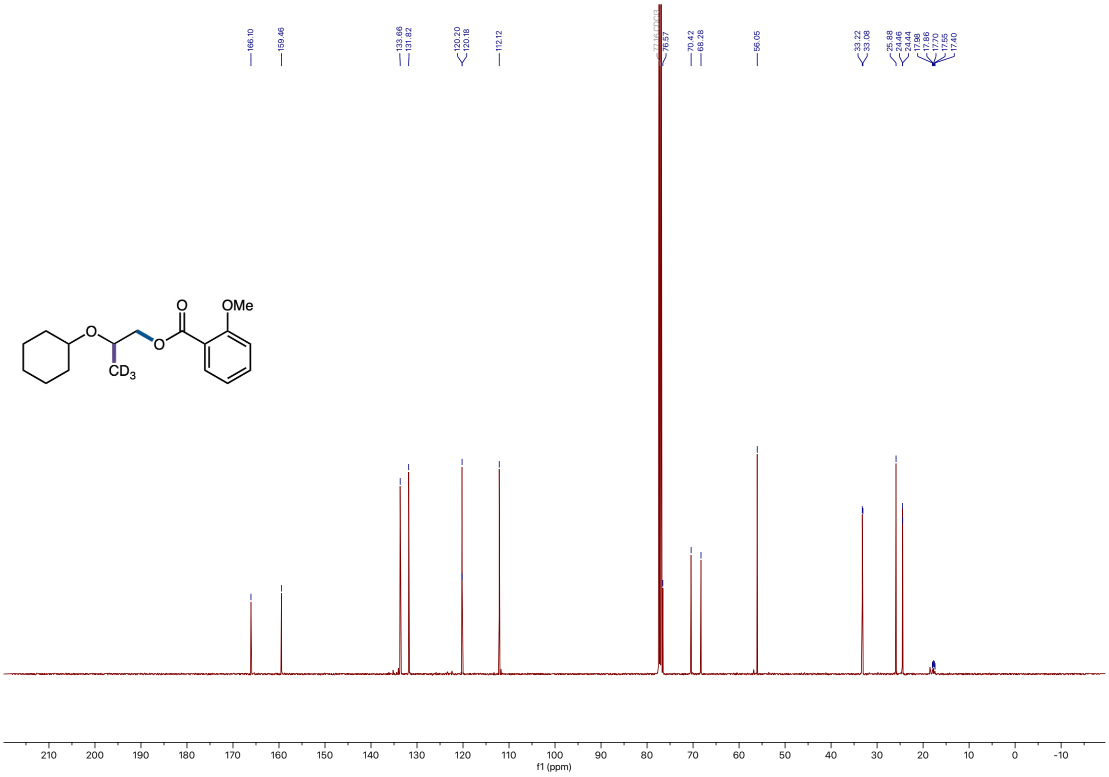

43, ^{1}H NMR, 500 MHz, $CDCl_3$

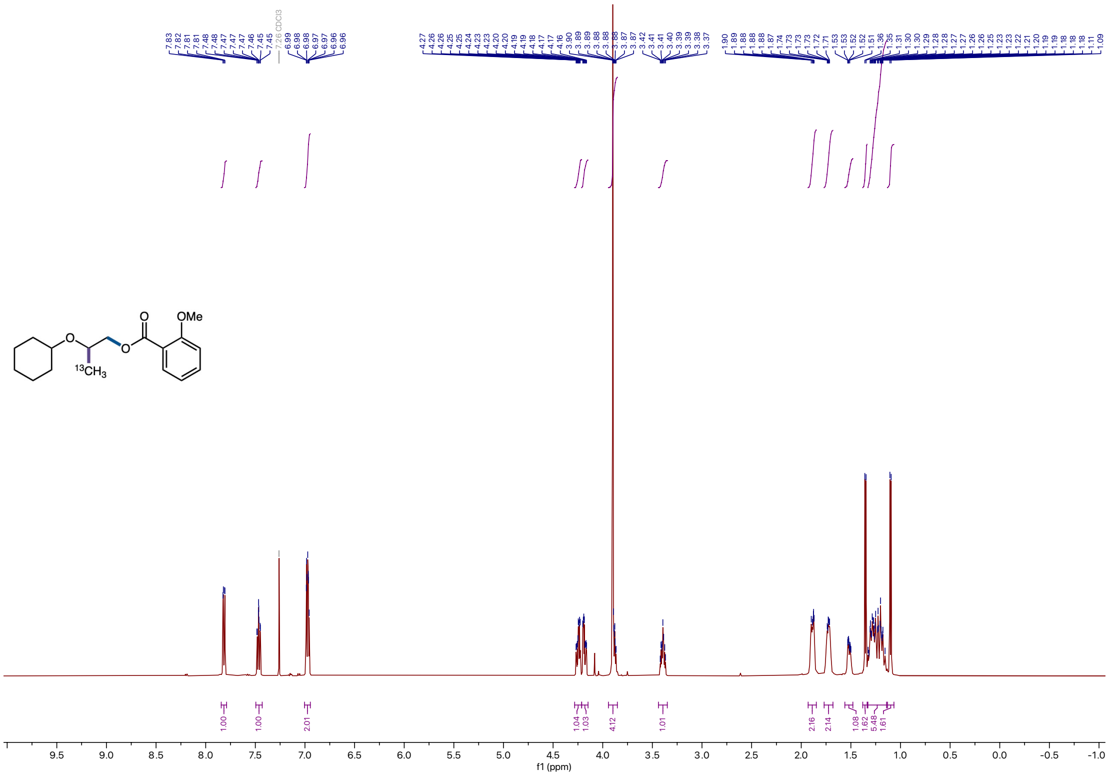

43, ^{13}C NMR, 126 MHz, $CDCl_3$

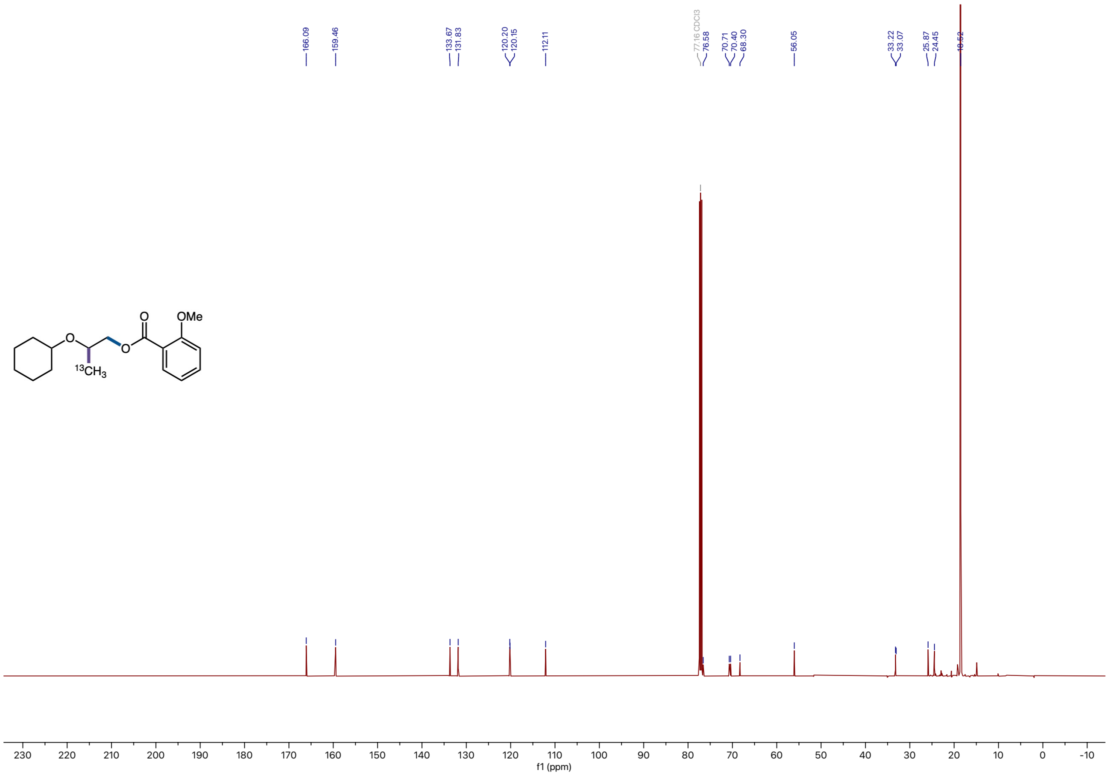

44, ^{1}H NMR, 500 MHz, $CDCl_3$

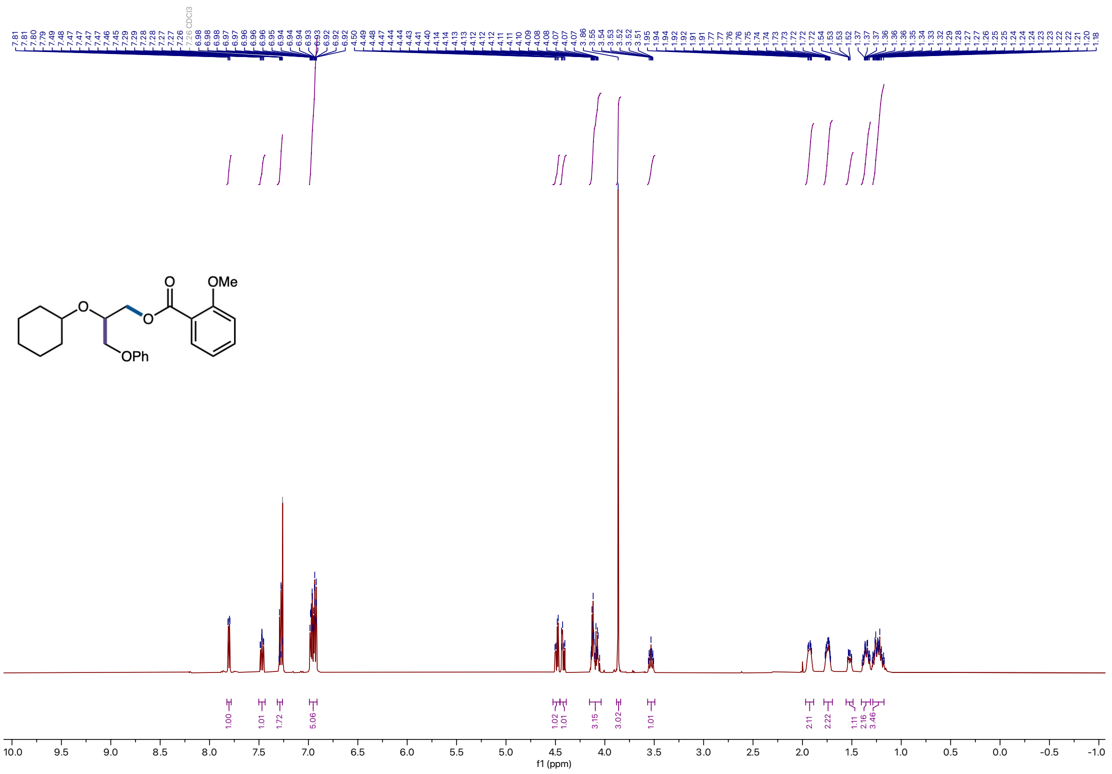

44, ^{13}C NMR, 126 MHz, $CDCl_3$

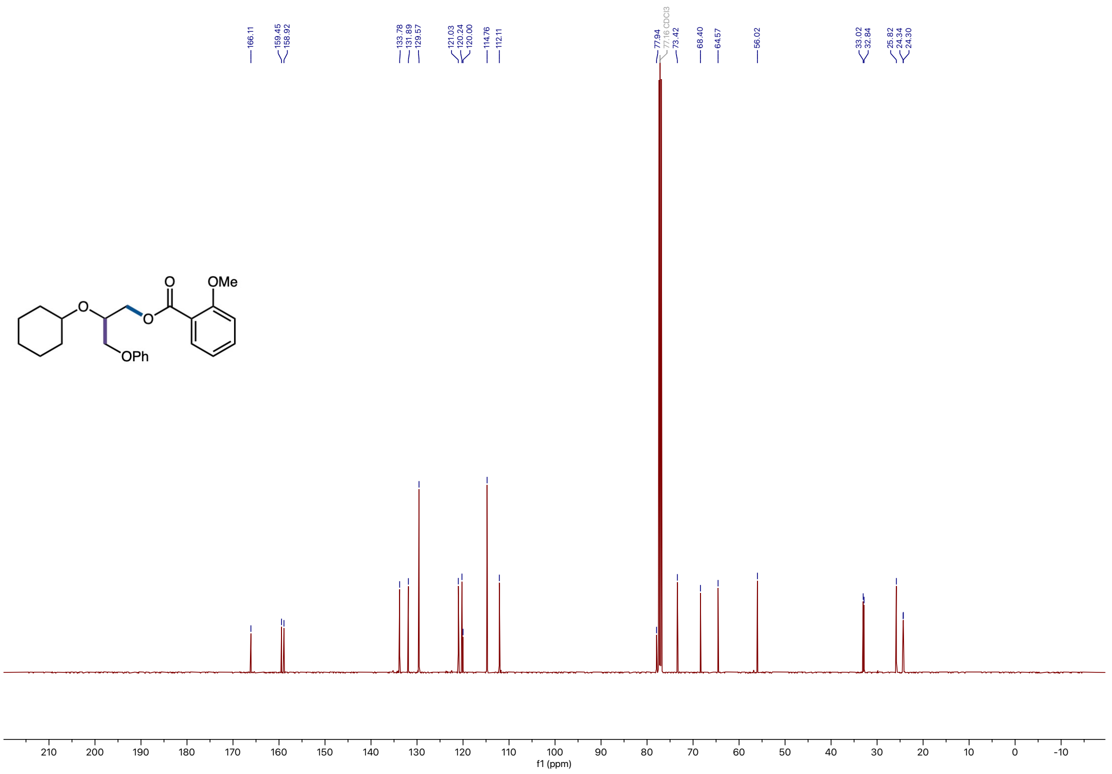

45, ^{1}H NMR, 500 MHz, $CDCl_3$

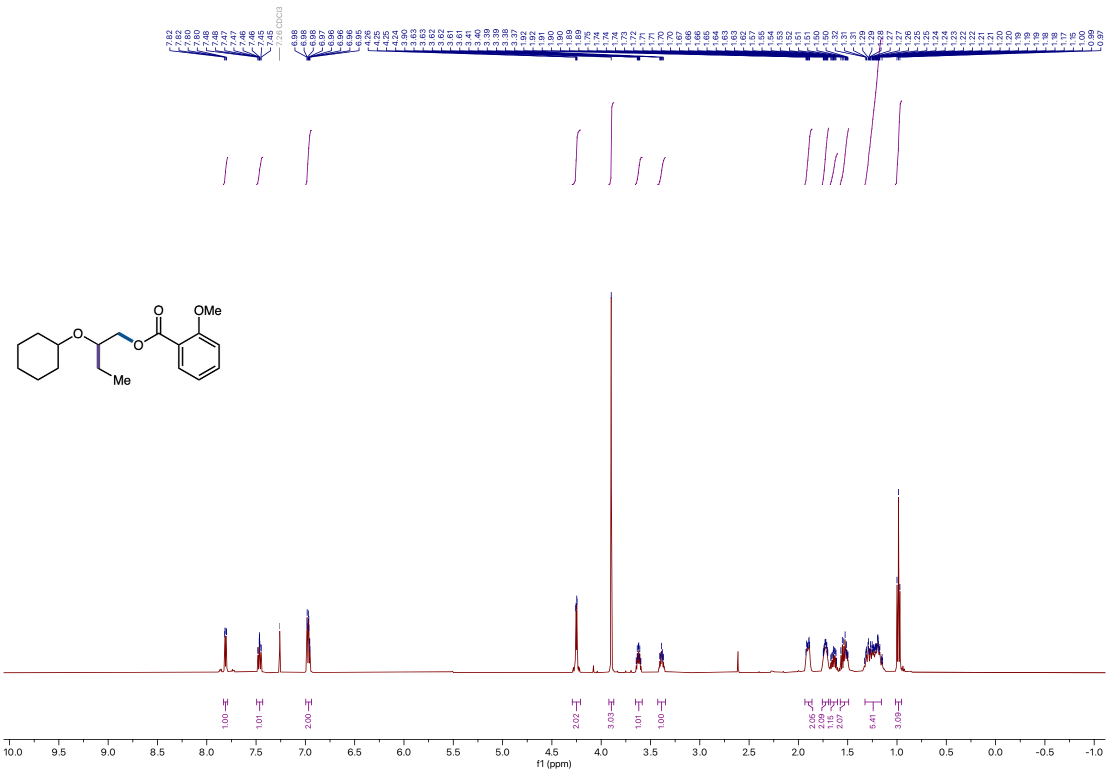

45, ^{13}C NMR, 126 MHz, DMSO-d_6

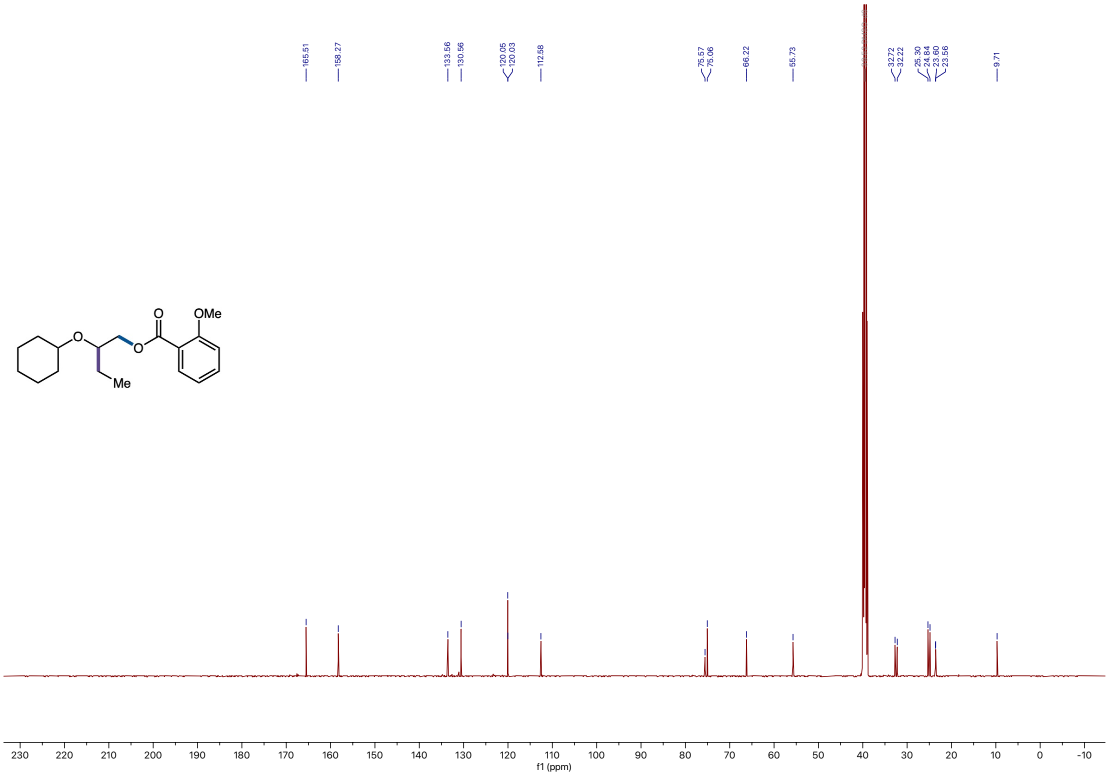

46, ^{1}H NMR, 500 MHz, $CDCl_3$

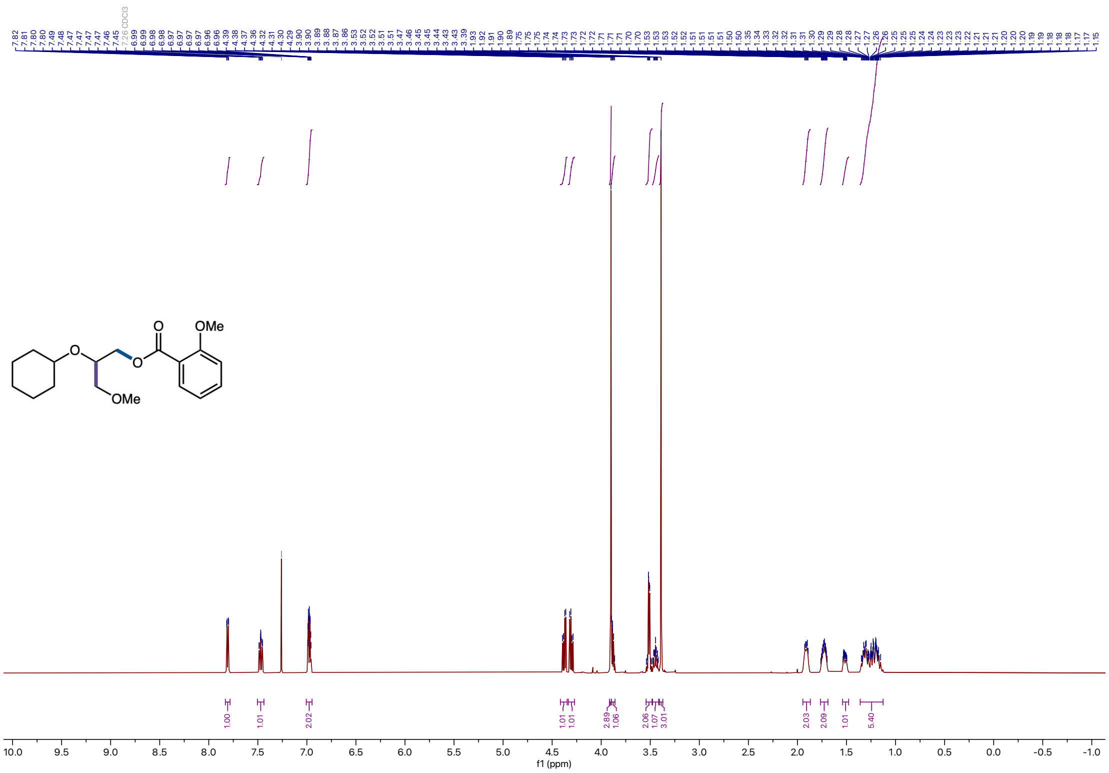

46, ^{13}C NMR, 126 MHz, $CDCl_3$

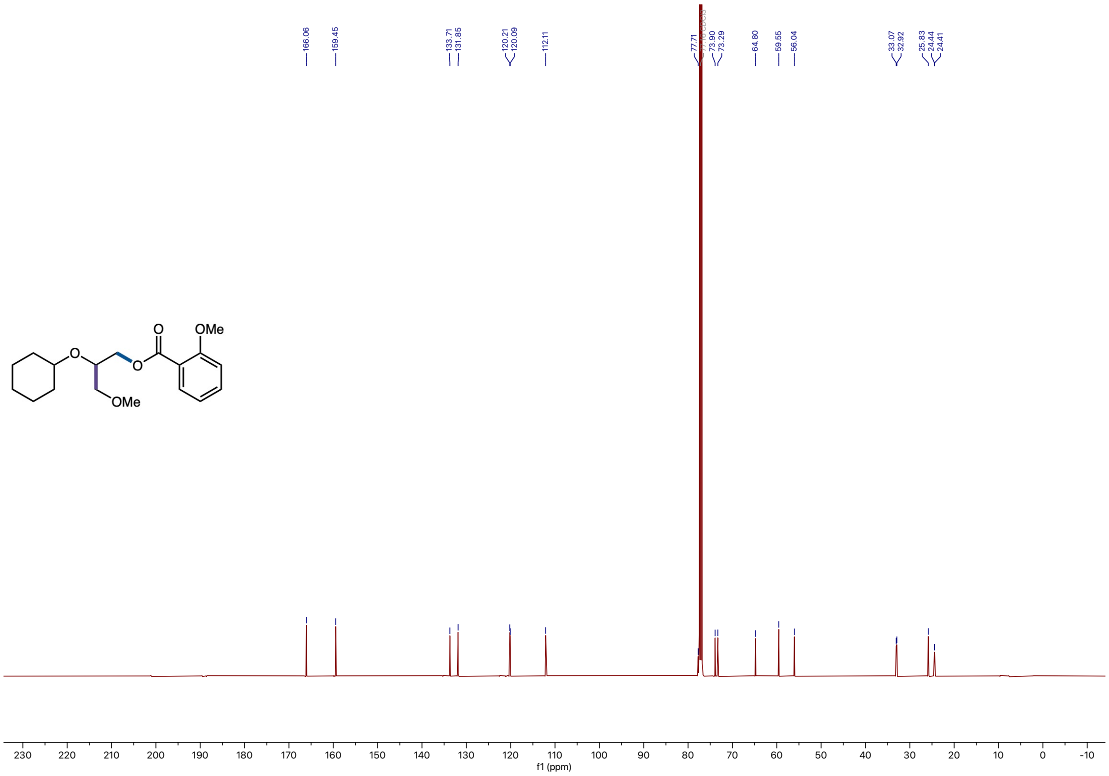

47, ^{1}H NMR, 500 MHz, CDCl$_3$

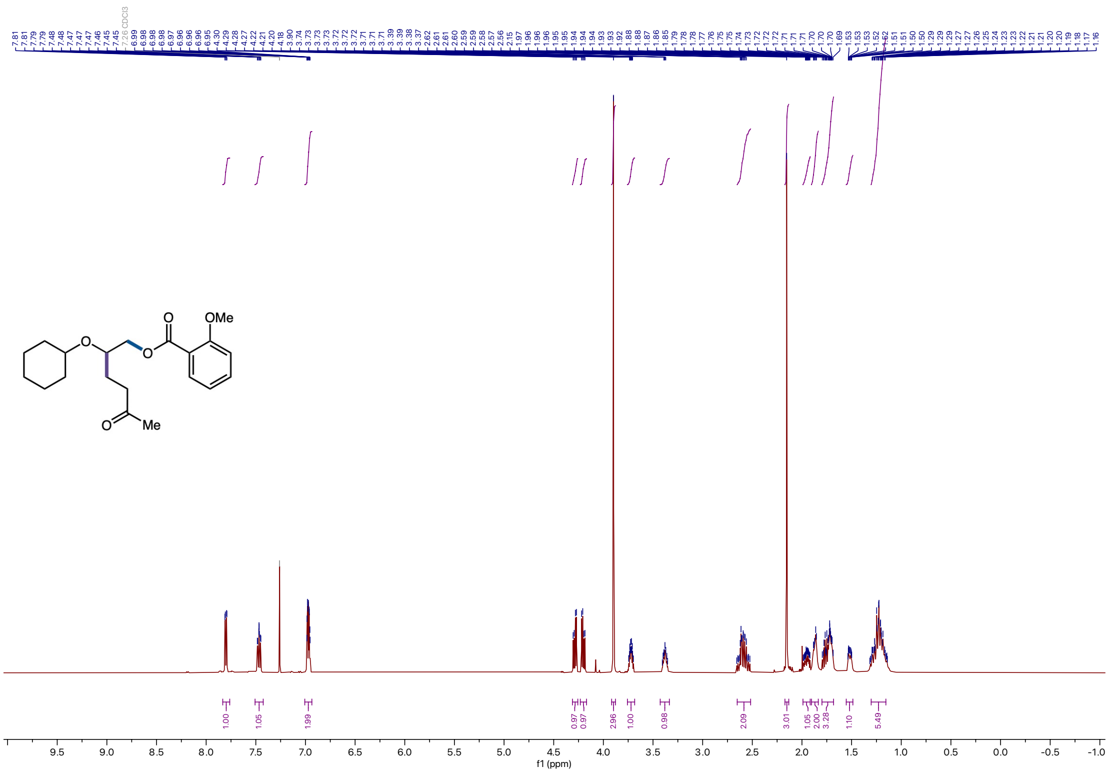

47, ^{13}C NMR, 126 MHz, CDCl$_3$

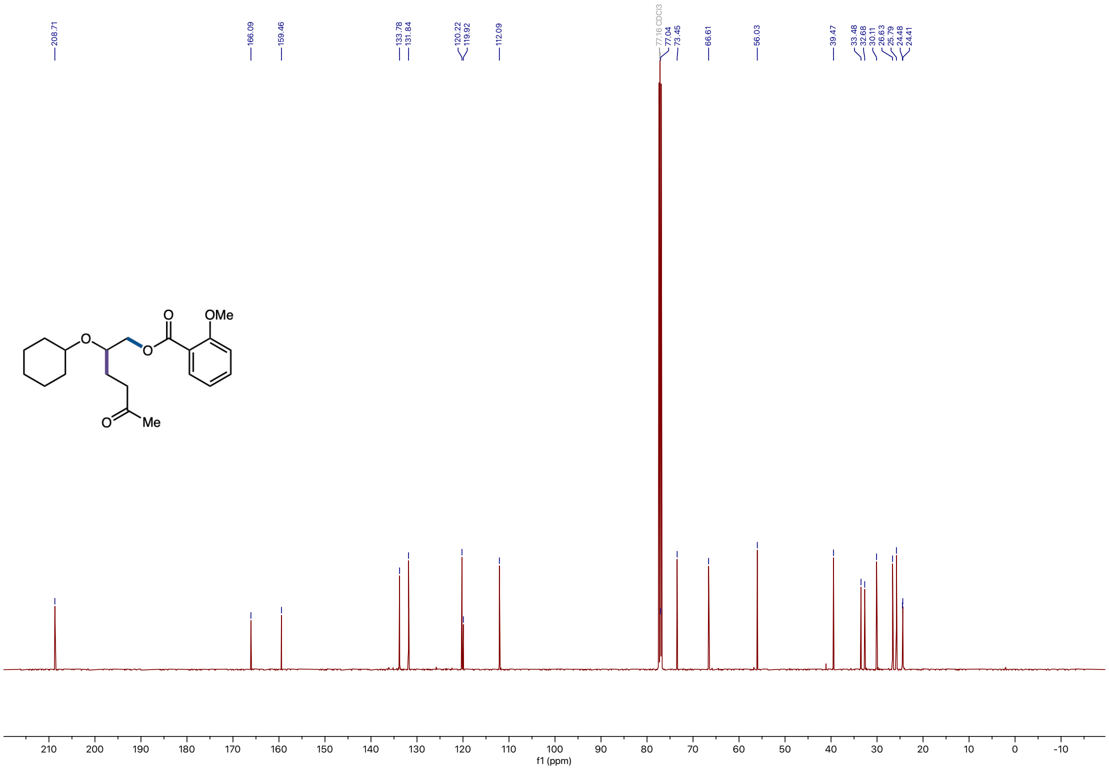

48, ^{1}H NMR, 500 MHz, $CDCl_3$

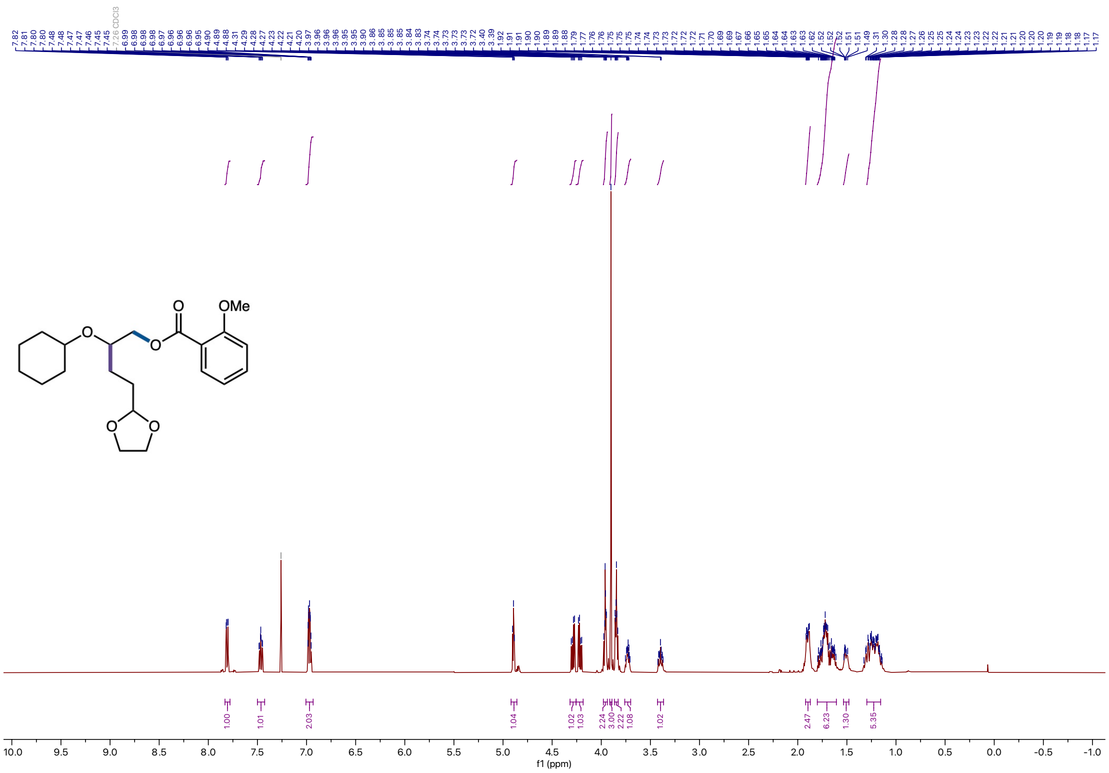

48, ^{13}C NMR, 126 MHz, DMSO-d_6

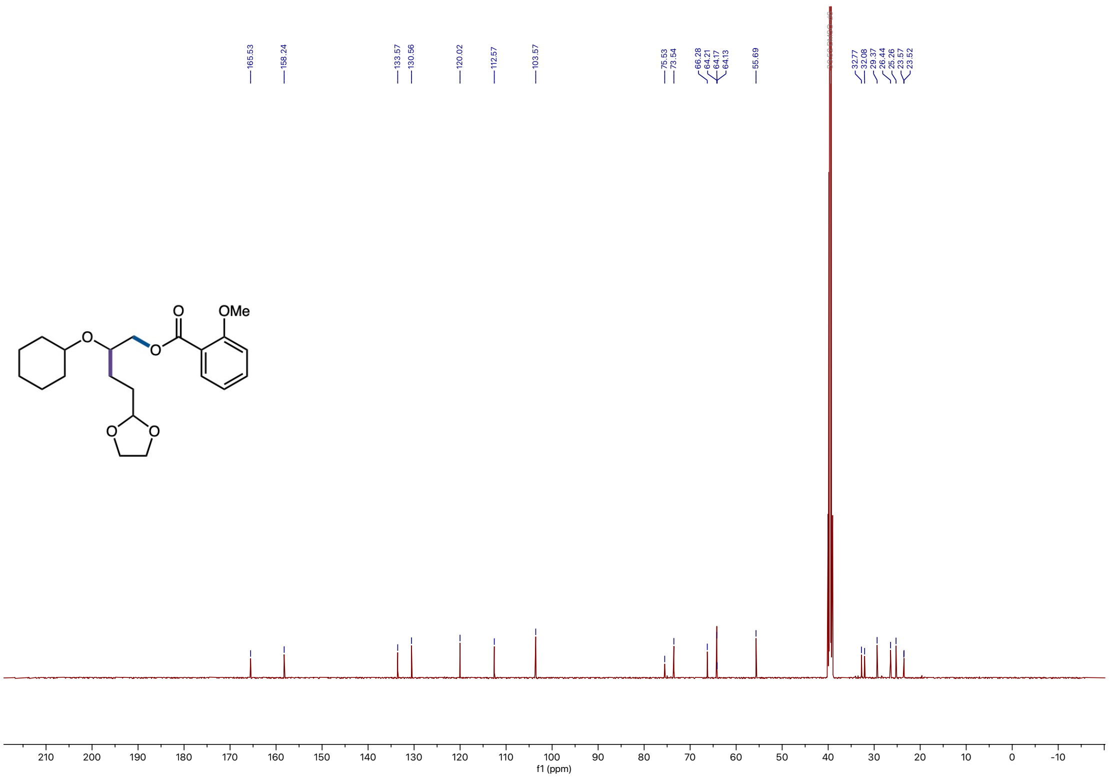

49, ^{1}H NMR, 500 MHz, $CDCl_3$

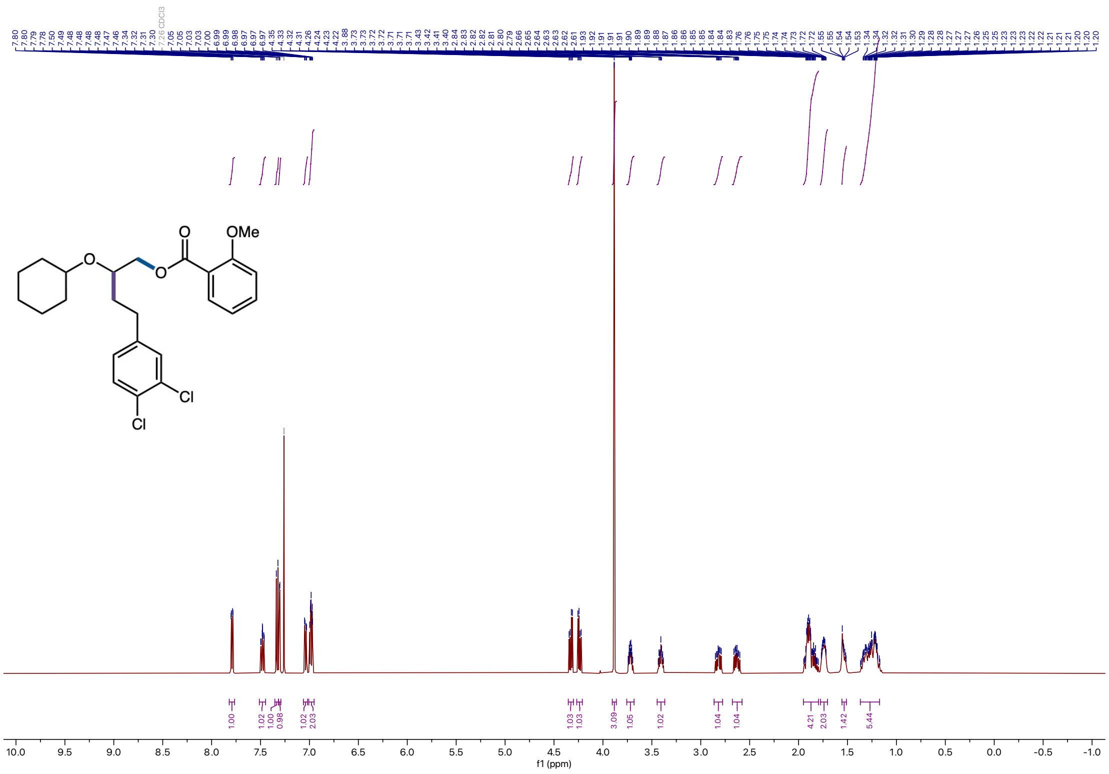

49, ^{13}C NMR, 126 MHz, DMSO-d_6

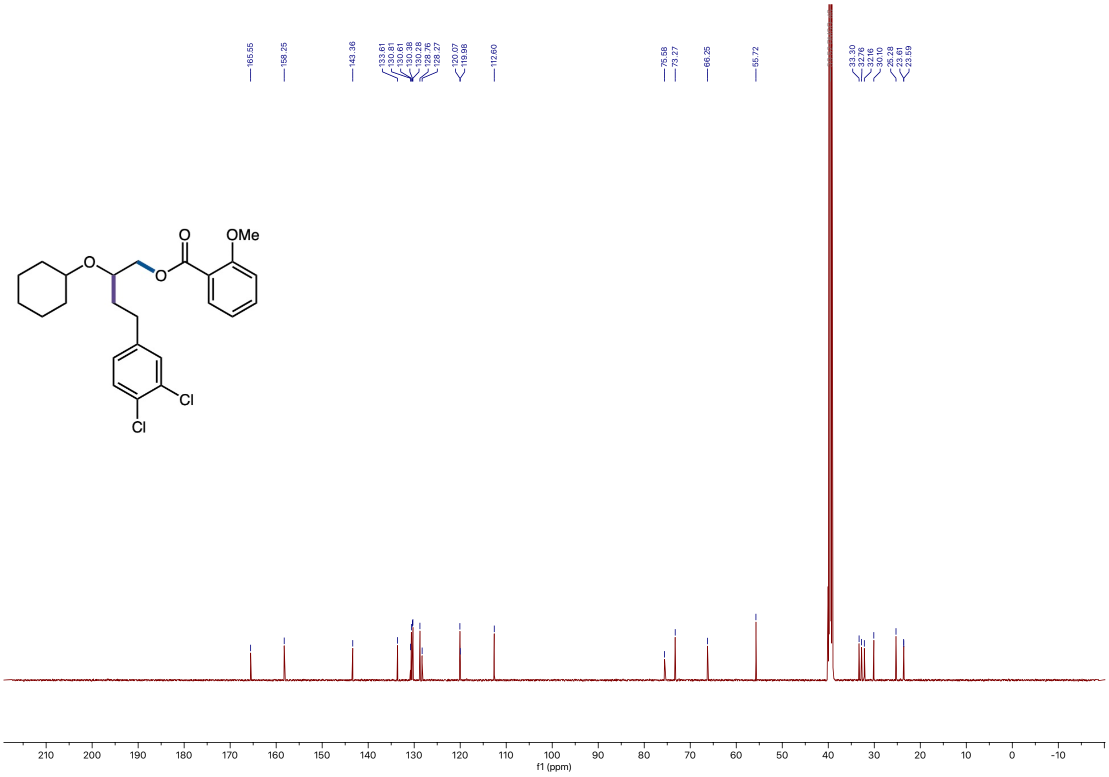

50, ^{1}H NMR, 500 MHz, $CDCl_3$

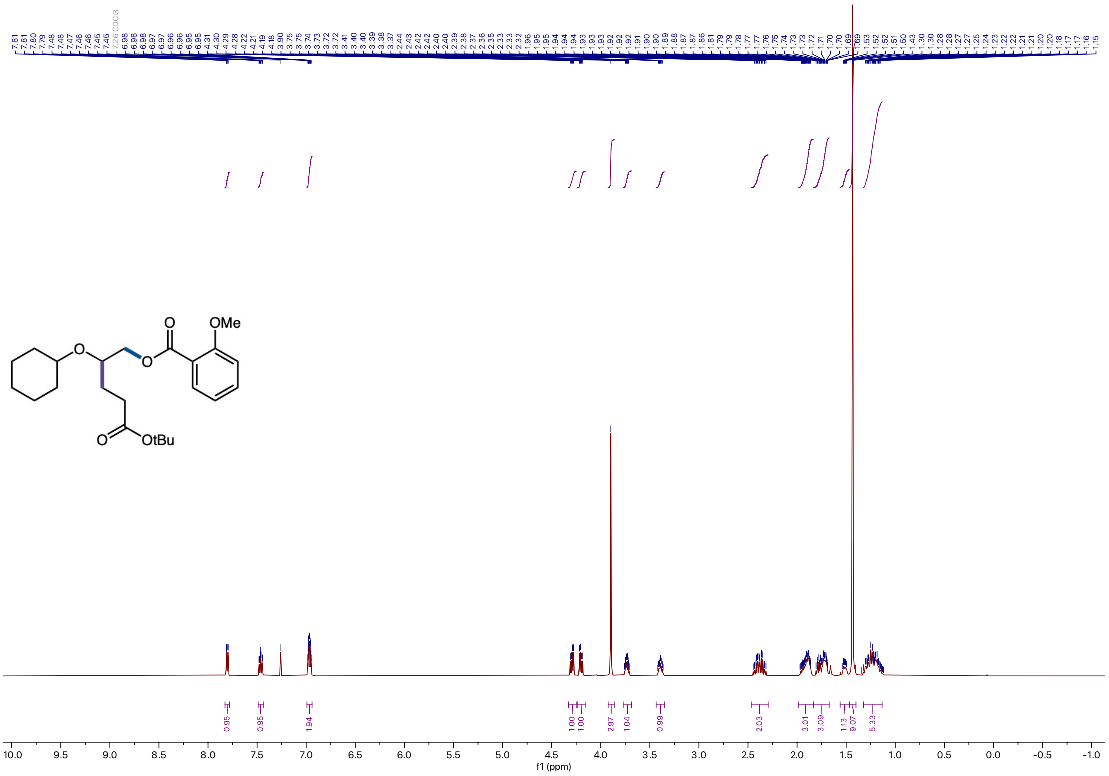

50, ^{13}C NMR, 126 MHz, $(CD_3)_2CO$

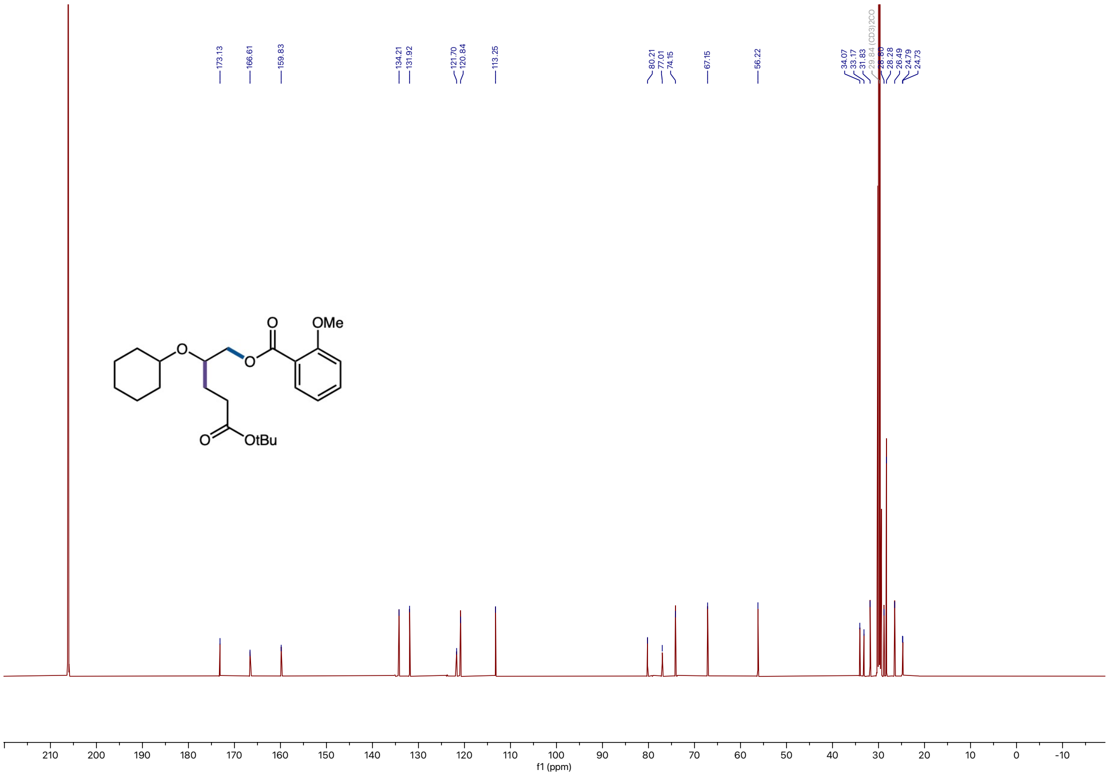

51, ^{1}H NMR, 500 MHz, $CDCl_3$

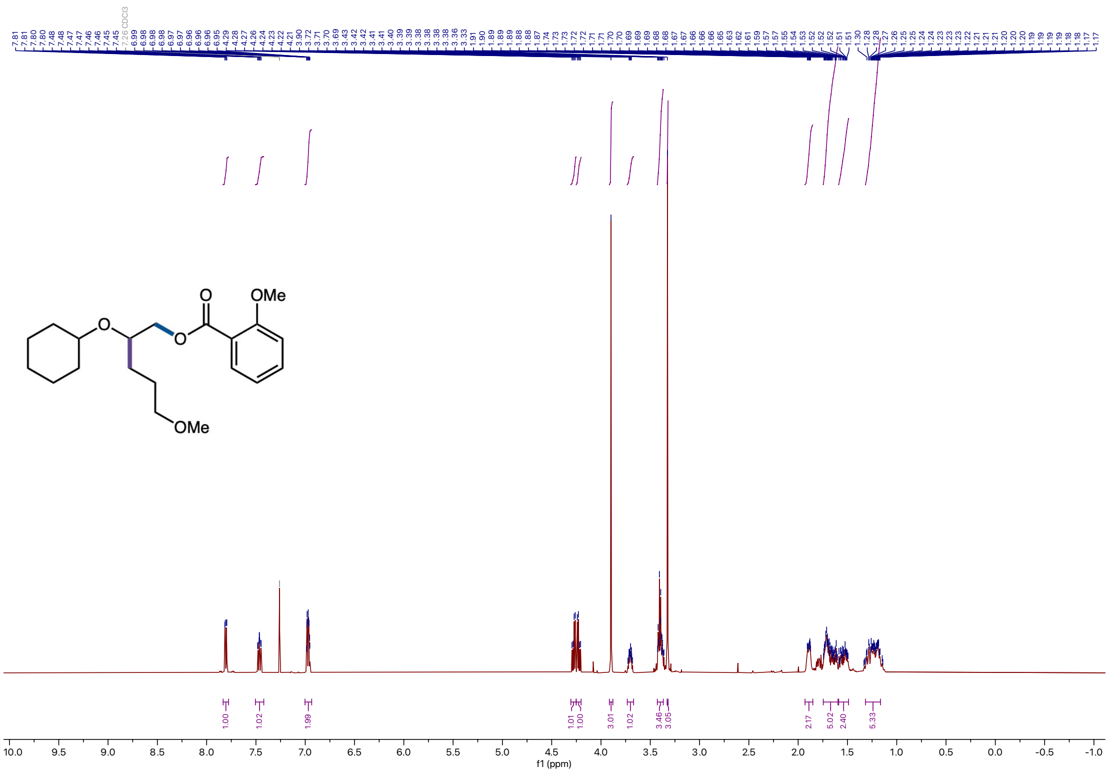

51, ^{13}C NMR, 126 MHz, DMSO-d_6

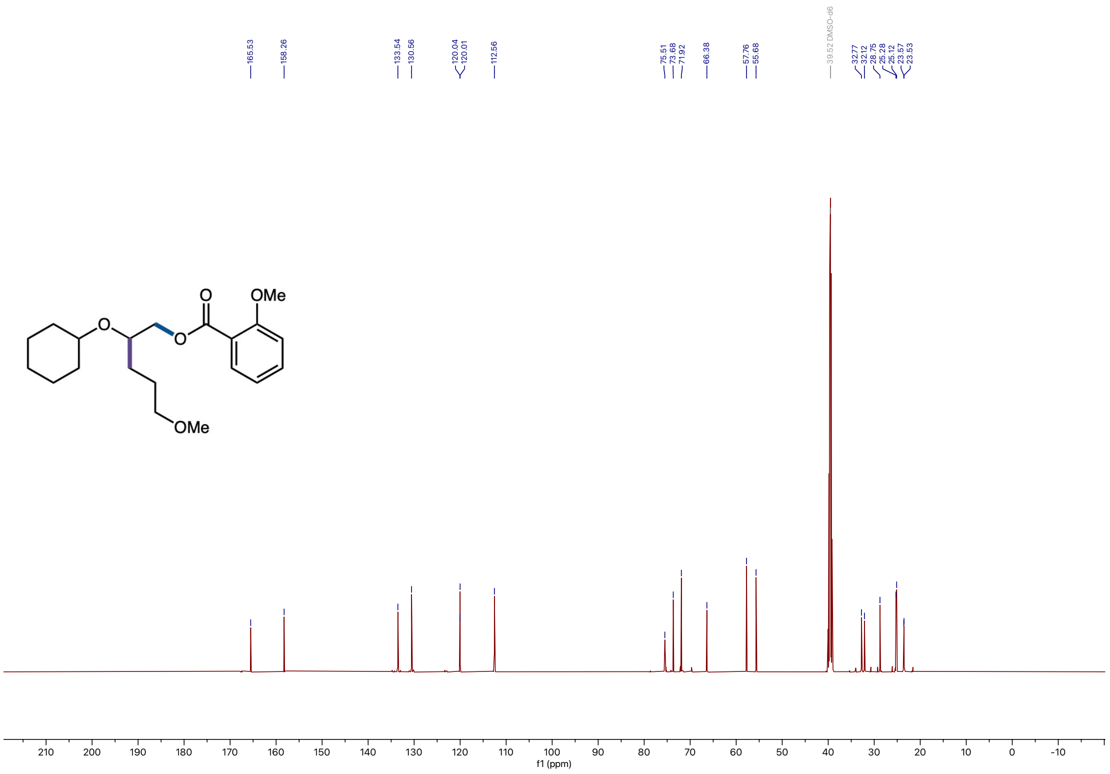

52, ^{1}H NMR, 500 MHz, $CDCl_3$

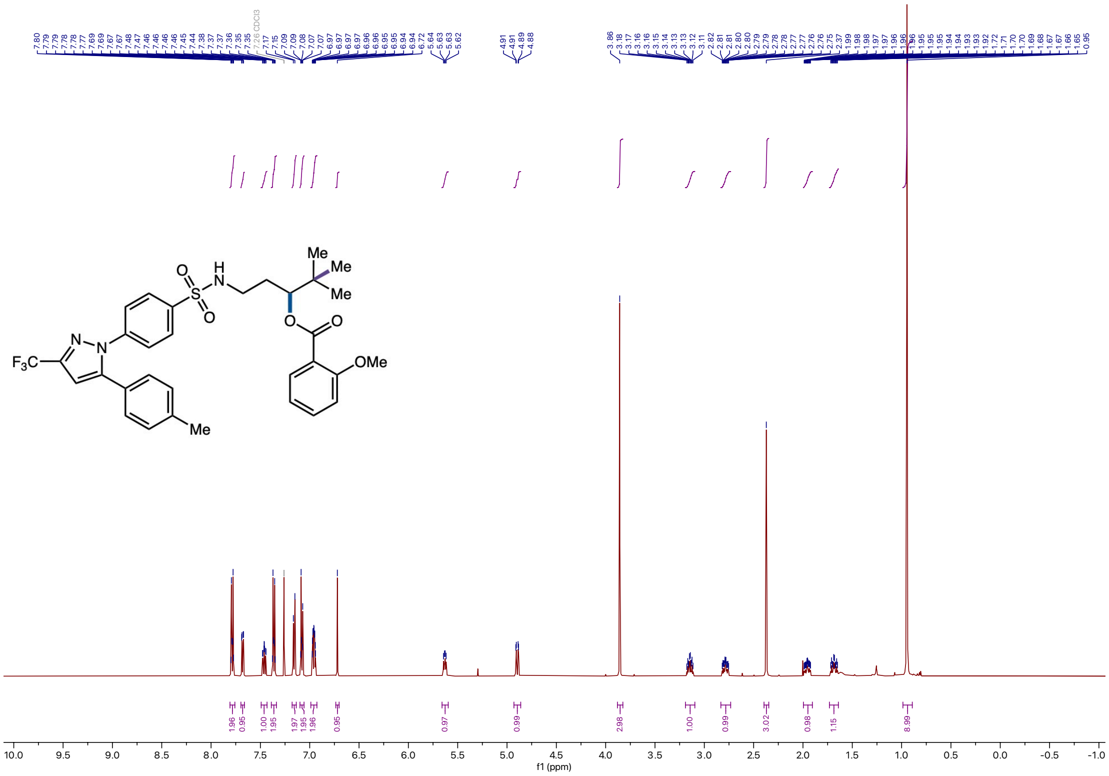

52, ^{13}C NMR, 126 MHz, $CDCl_3$

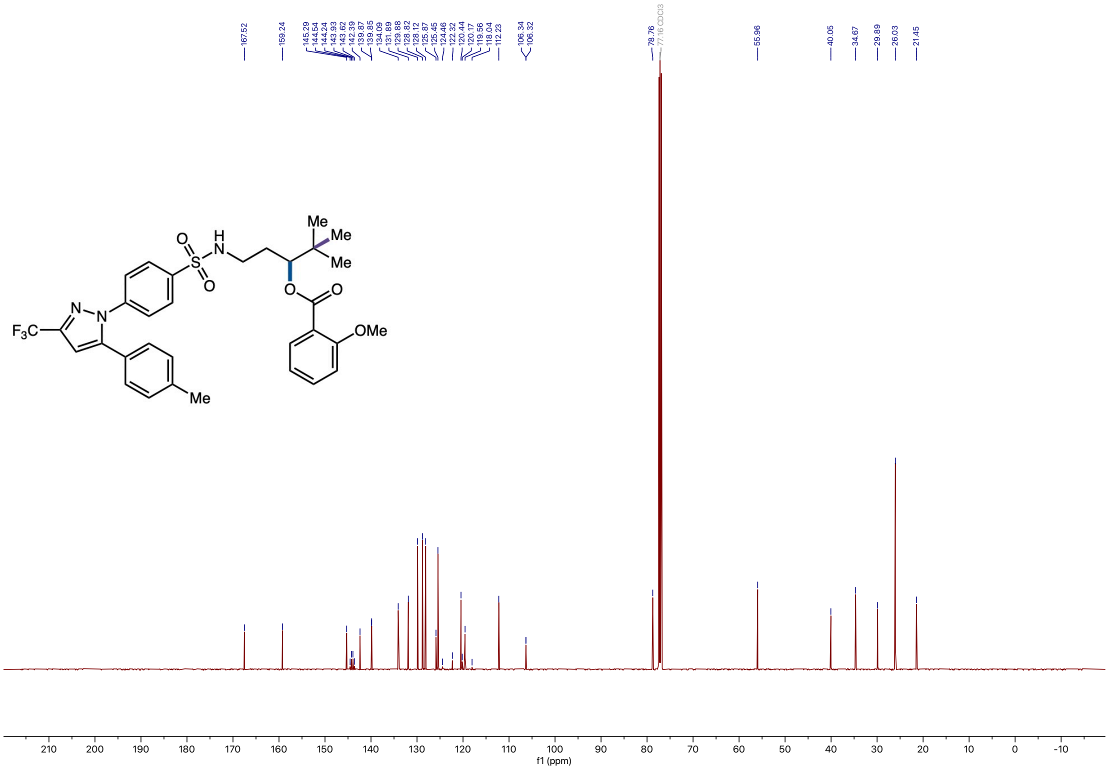

52, ^{19}F NMR, 376 MHz, $CDCl_3$

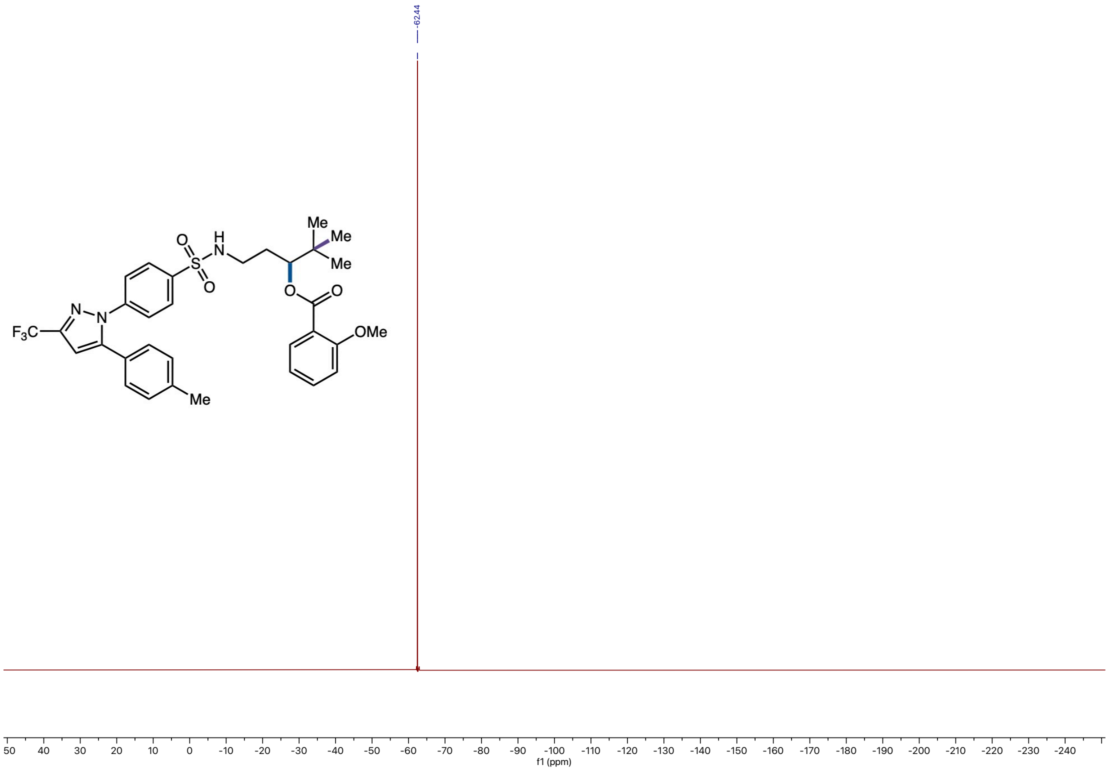

53, ^{1}H NMR, 500 MHz, $CDCl_3$

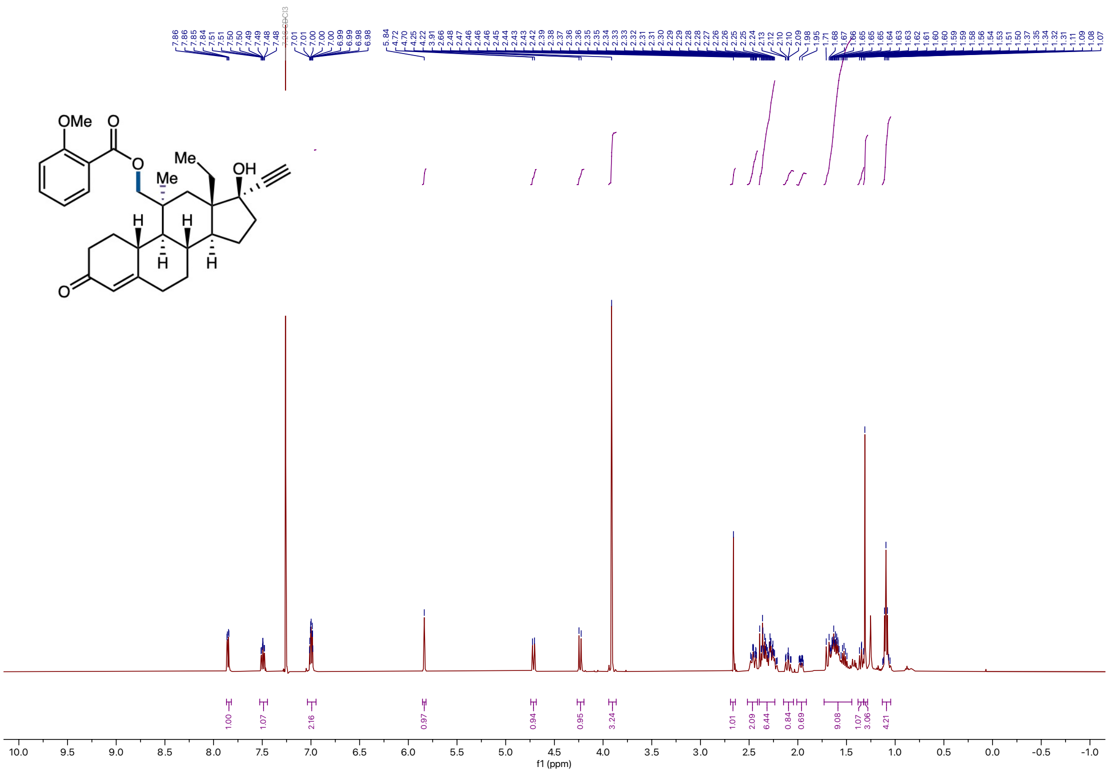

53, ^{13}C NMR, 126 MHz, $CDCl_3$

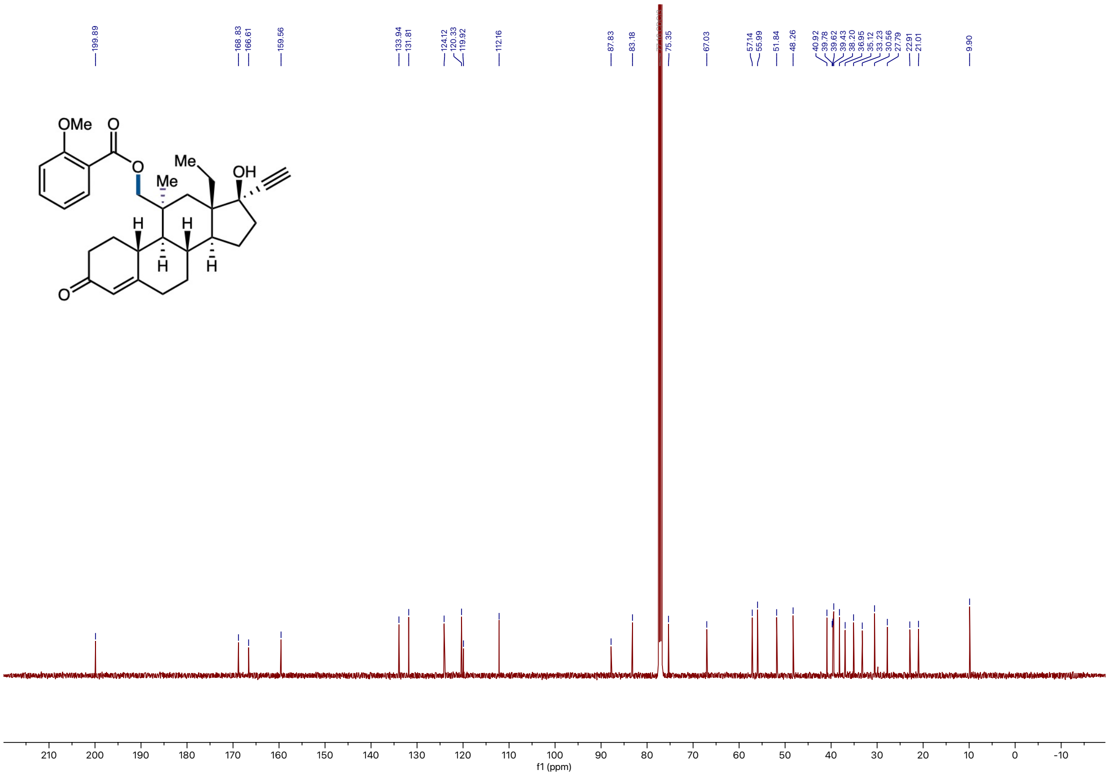

53, HSQC, $CDCl_3$

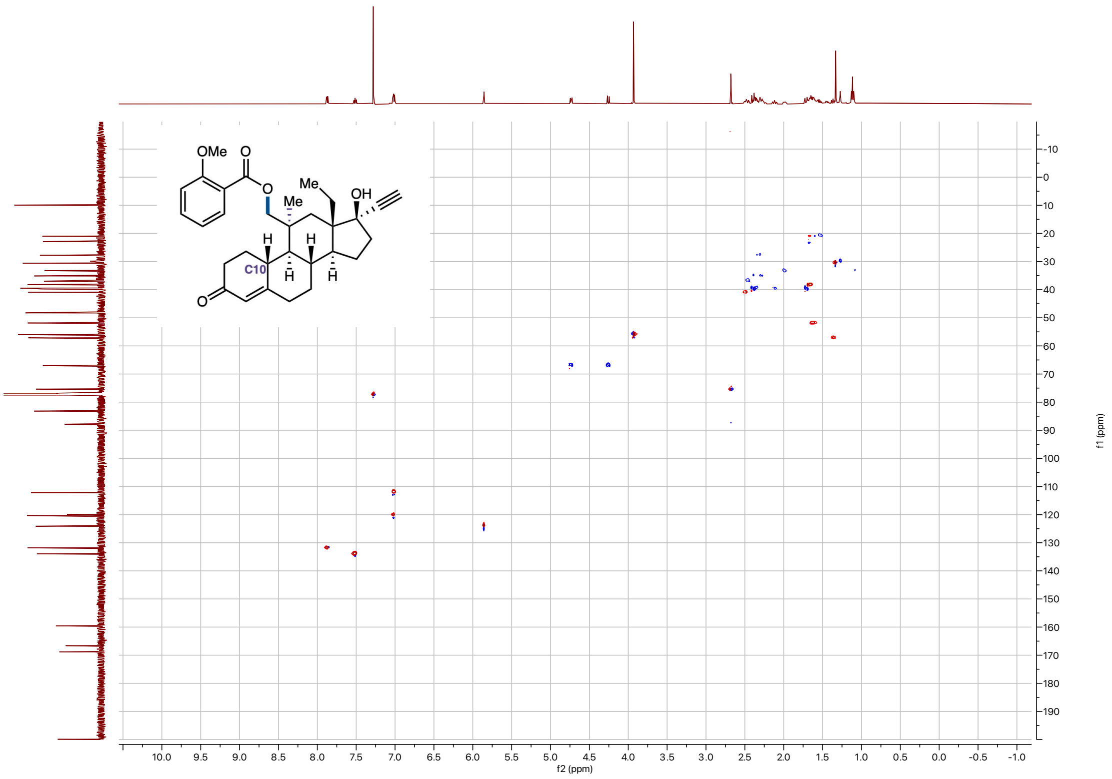

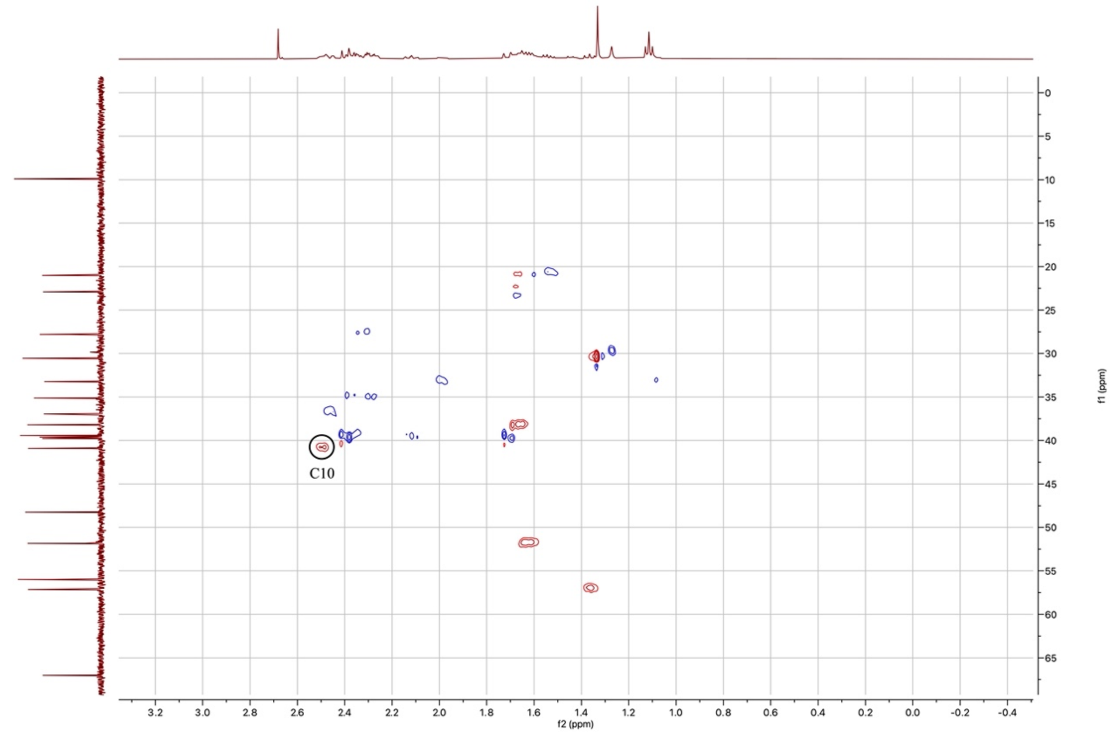

C10 (40.9)/C10-H (2.50) assigned by analogy to etonogestrel starting material.[16]

53, COSY, $CDCl_3$

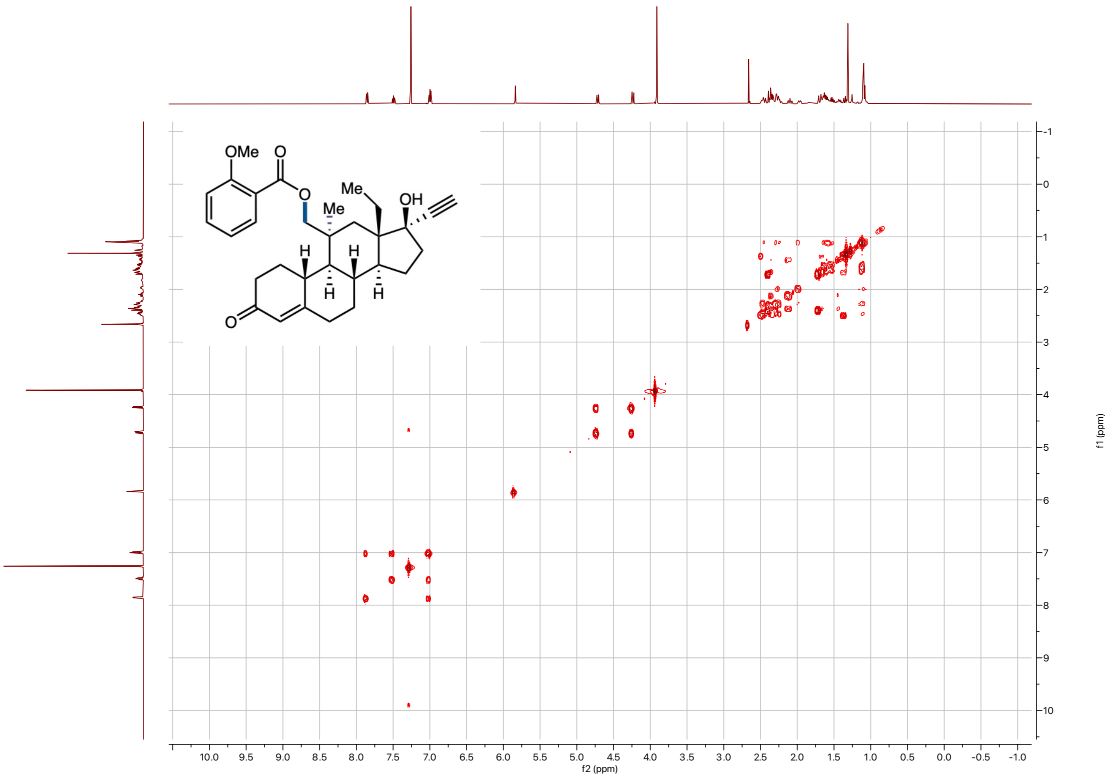

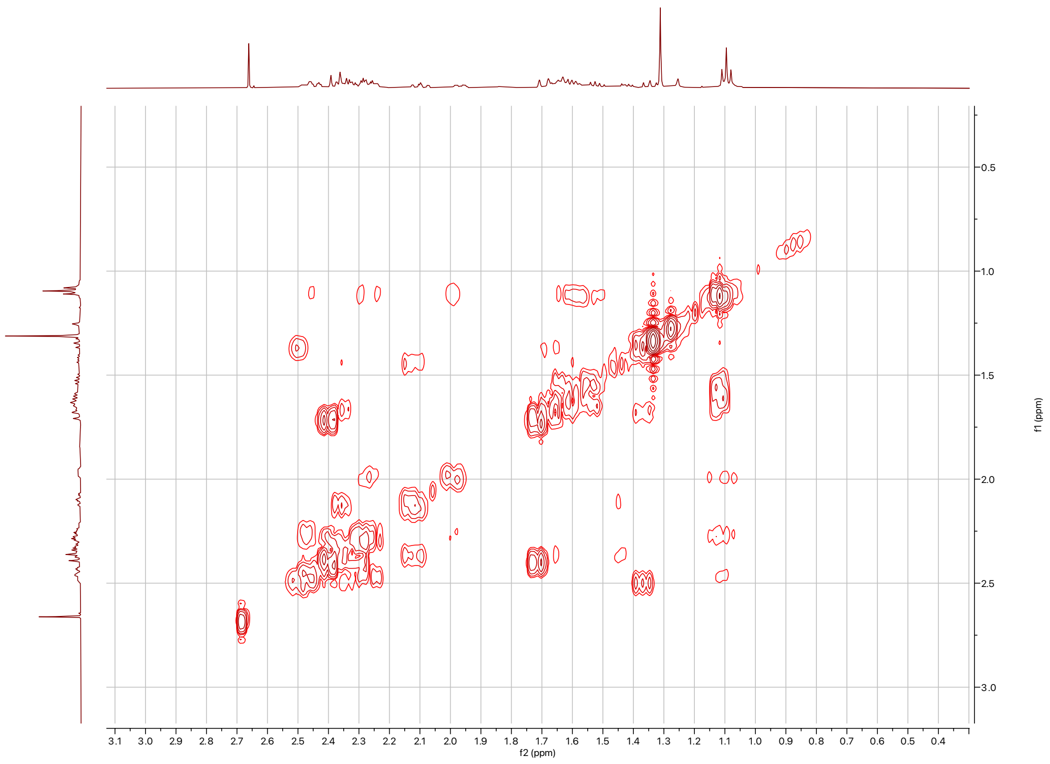

53, NOESY, $CDCl_3$

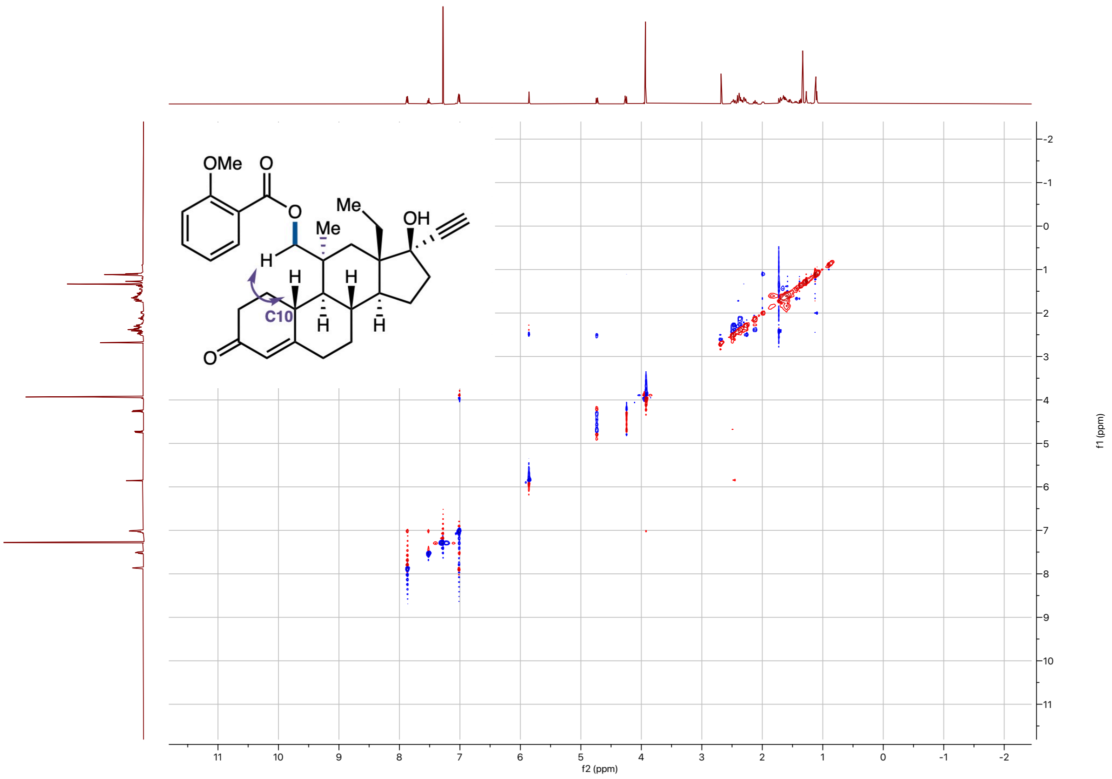

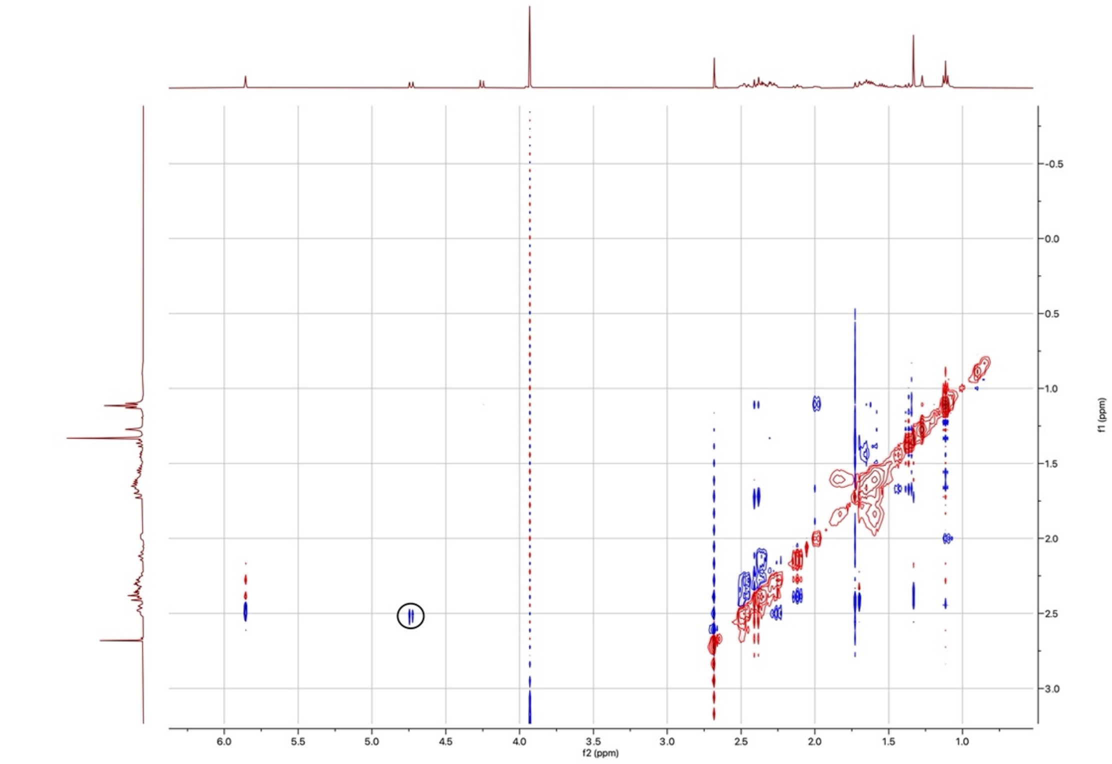

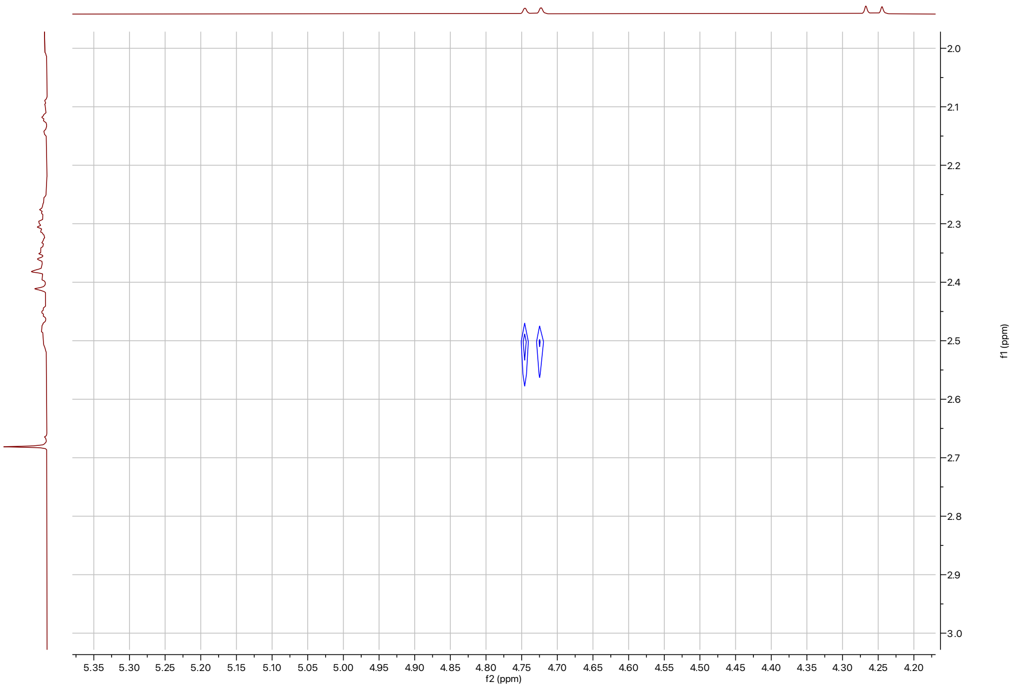

2.0
2.1
2.2
2.3
2.4
2.5
2.6
2.7
2.8
2.9
3.0
f1 (ppm)
5.35 5.30 5.25 5.20 5.15 5.10 5.05 5.00 4.95 4.90 4.85 4.80 4.75 4.70 4.65 4.60 4.55 4.50 4.45 4.40 4.35 4.30 4.25 4.20
f2 (ppm)

54, ^{1}H NMR, 500 MHz, $CDCl_3$

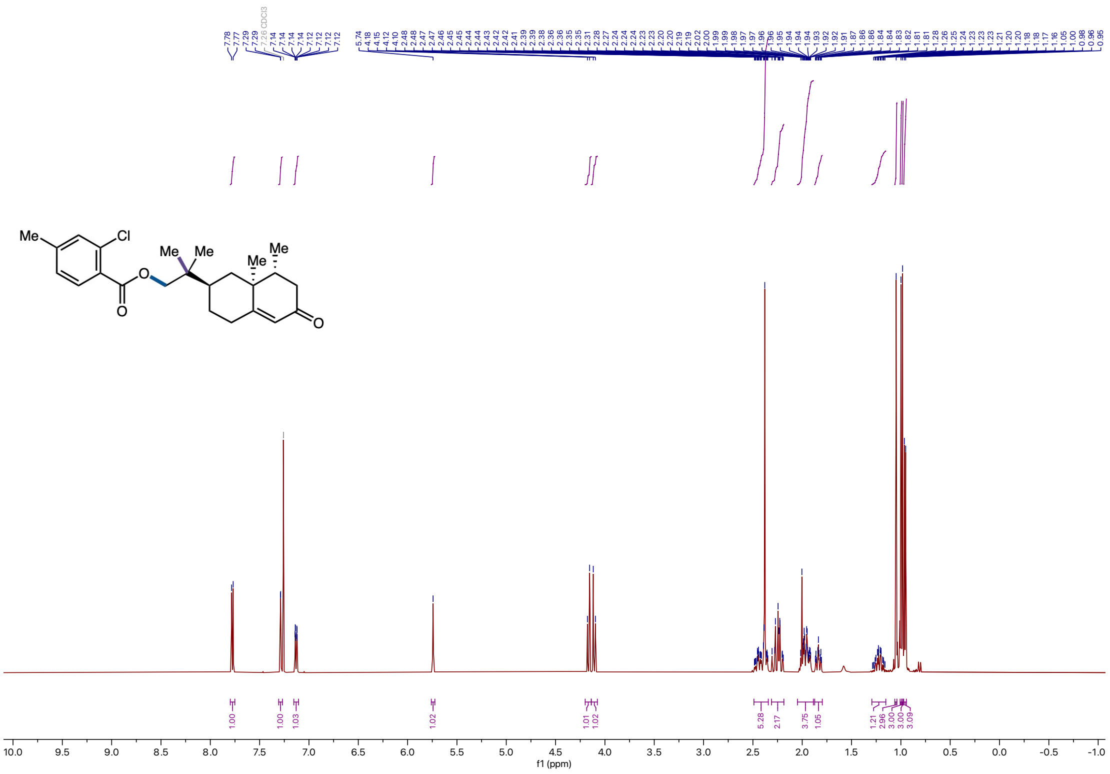

54, ^{13}C NMR, 126 MHz, $CDCl_3$

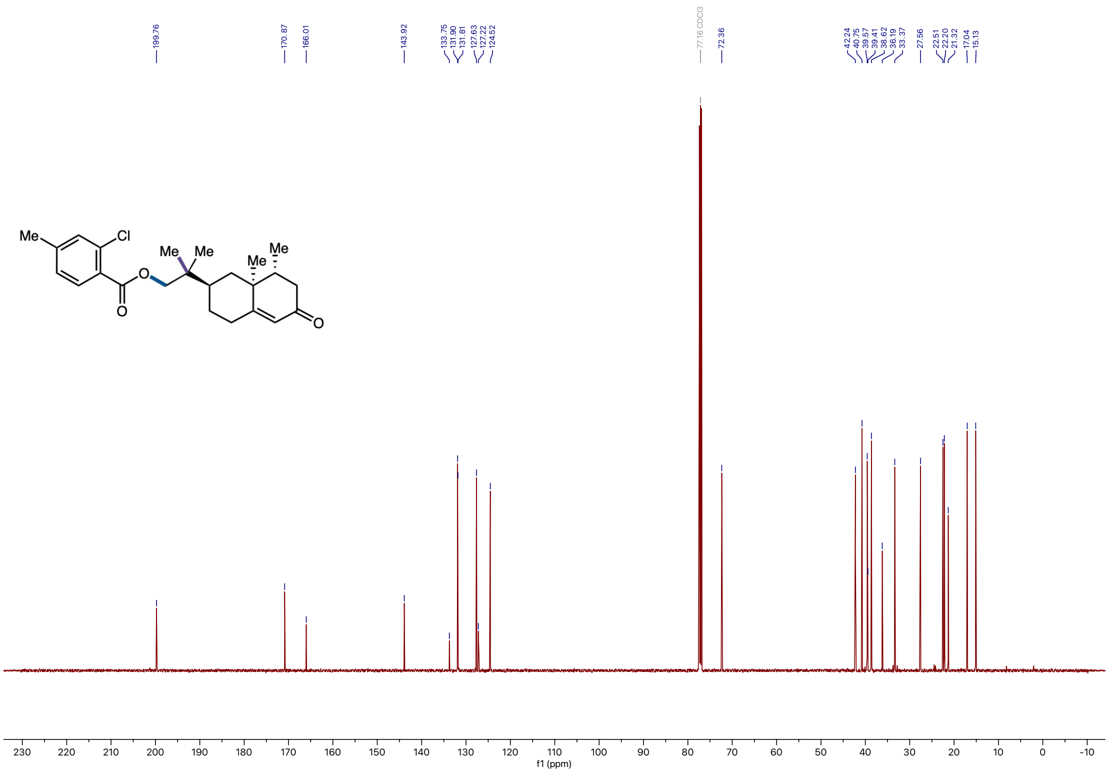

55, ^{1}H NMR, 500 MHz, $CDCl_3$

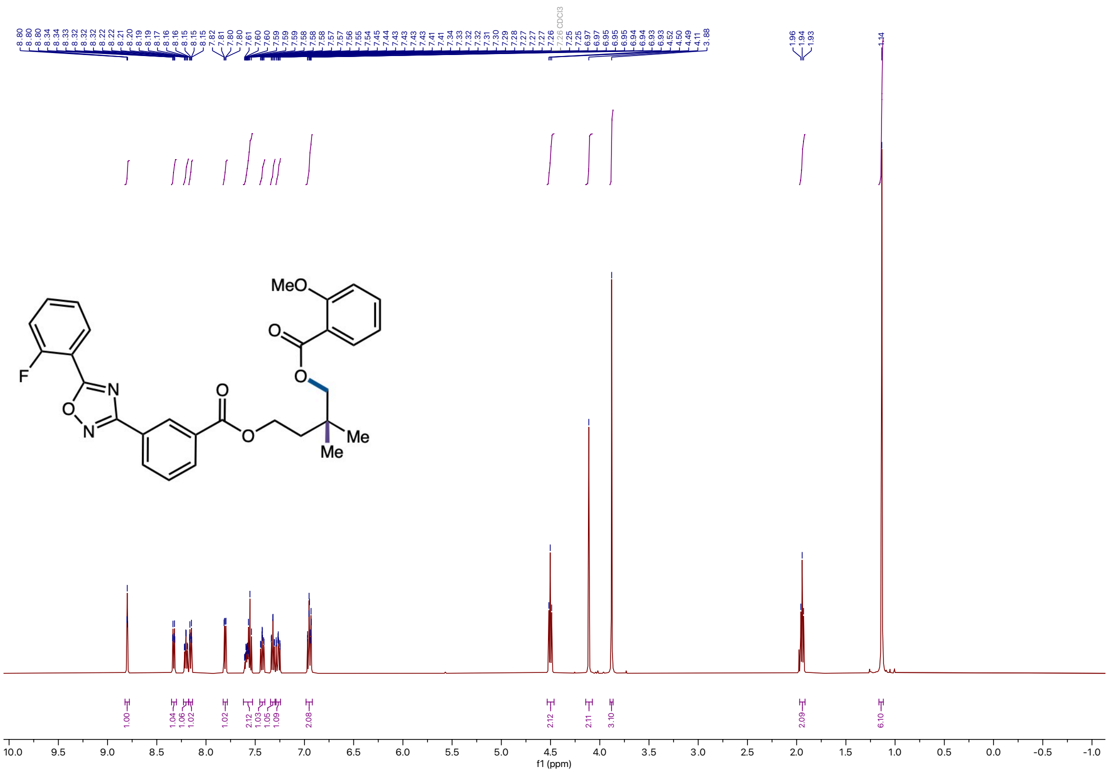

55, ^{13}C NMR, 126 MHz, $CDCl_3$

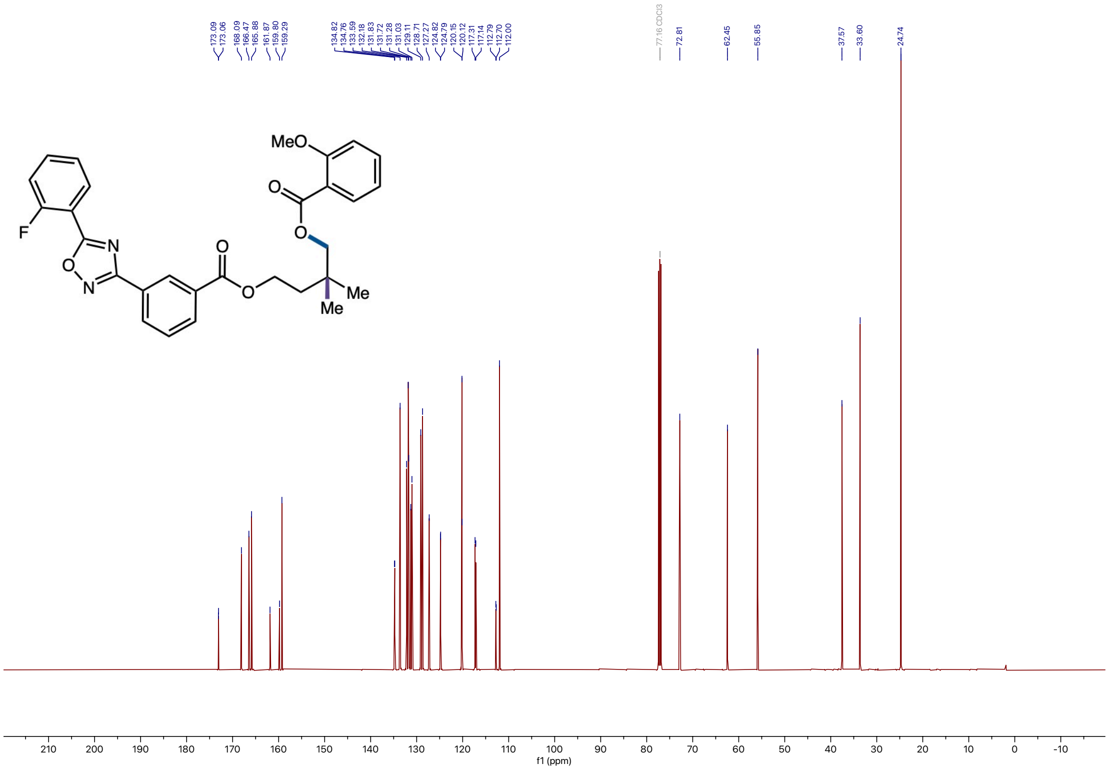

55, ^{19}F NMR, 376 MHz, $CDCl_3$

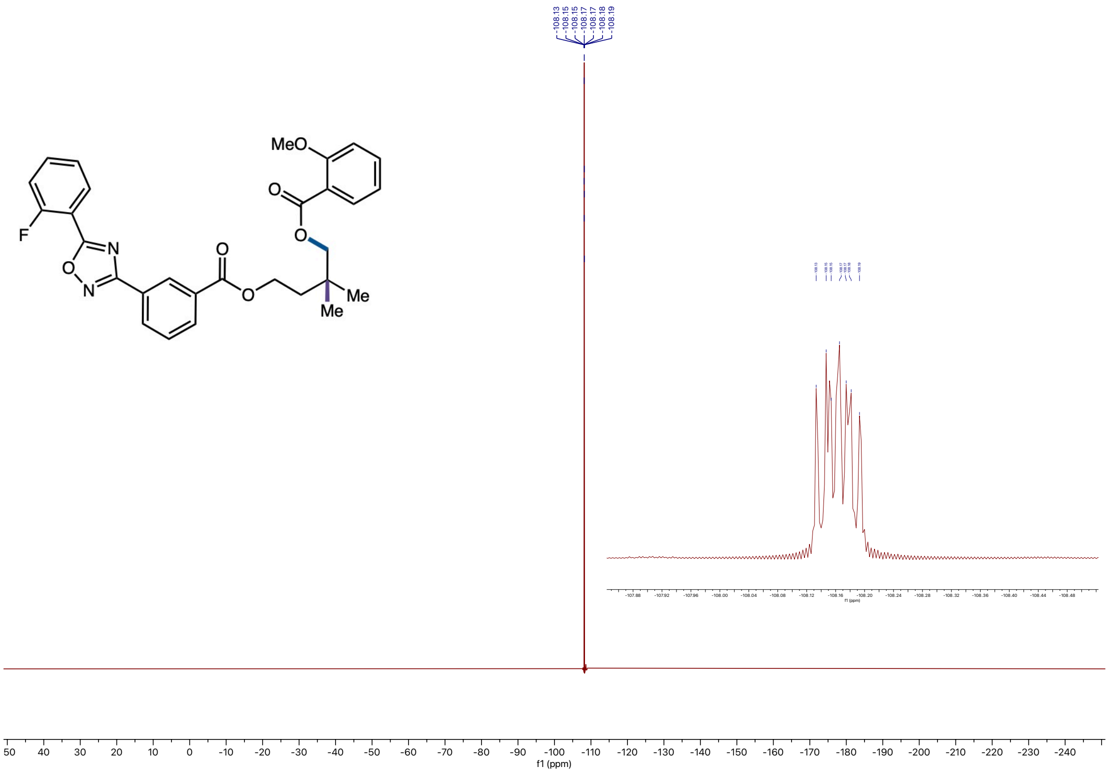

56, ^{1}H NMR, 500 MHz, $CDCl_3$

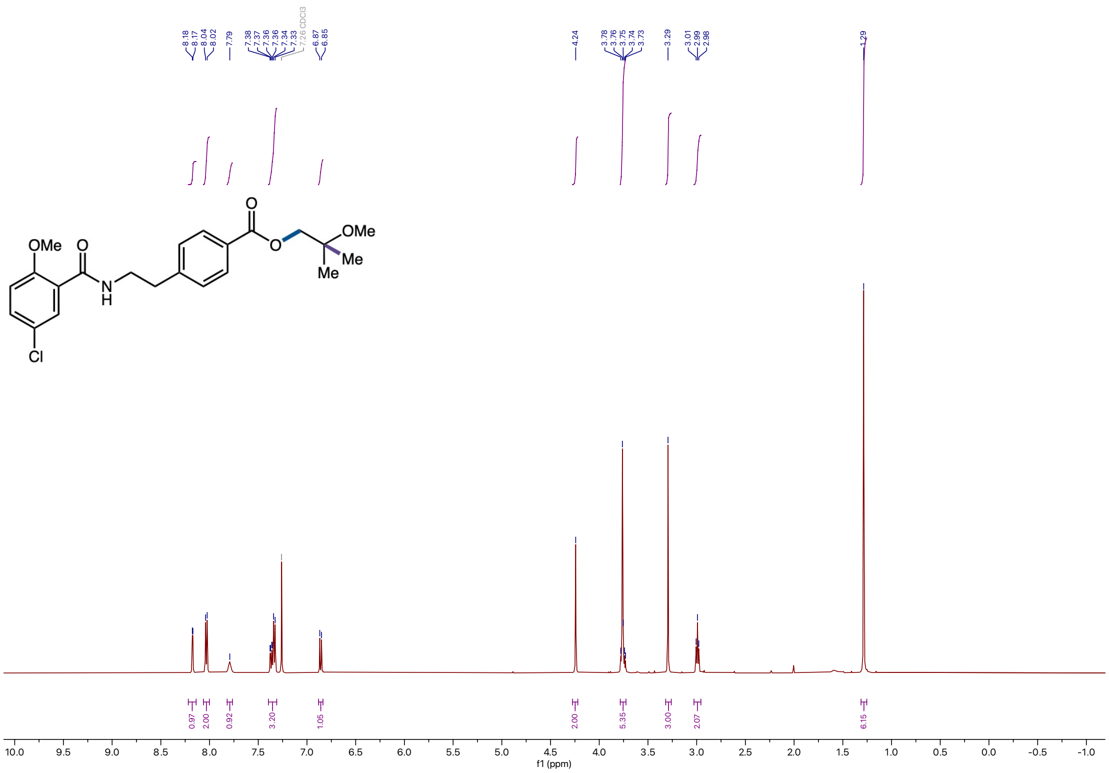

56, ^{13}C NMR, 126 MHz, $CDCl_3$

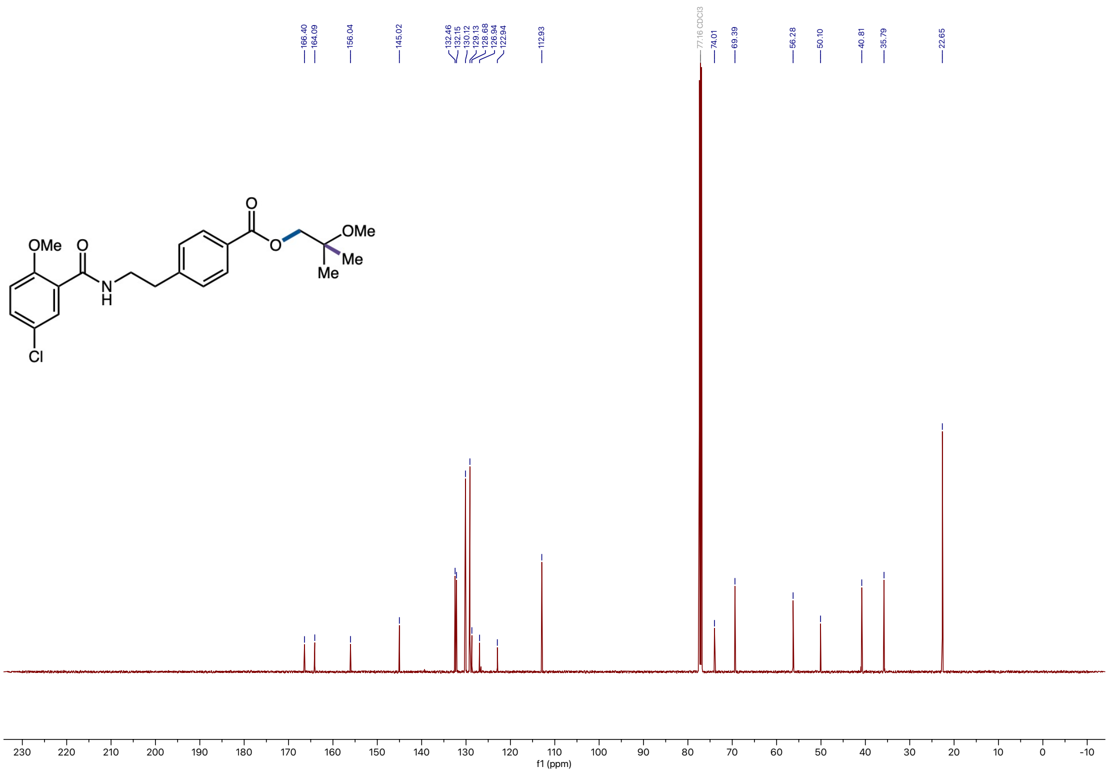

57, ^{1}H NMR, 500 MHz, $CDCl_3$

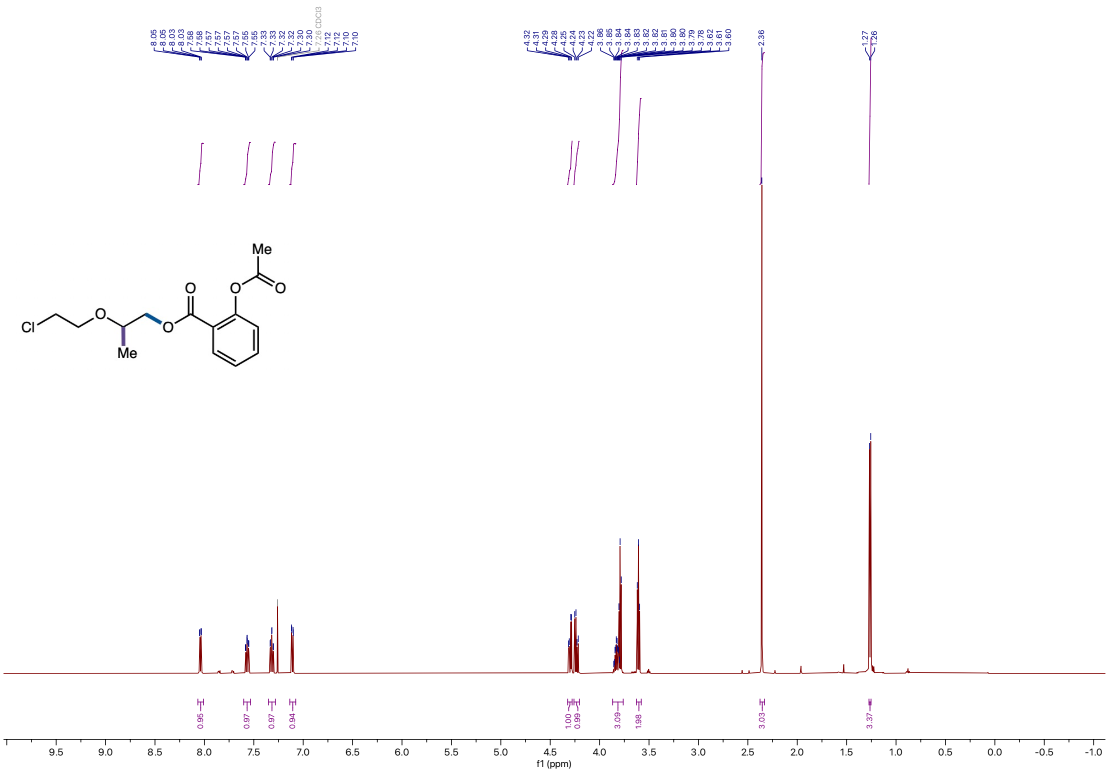

57, ^{13}C NMR, 126 MHz, $CDCl_3$

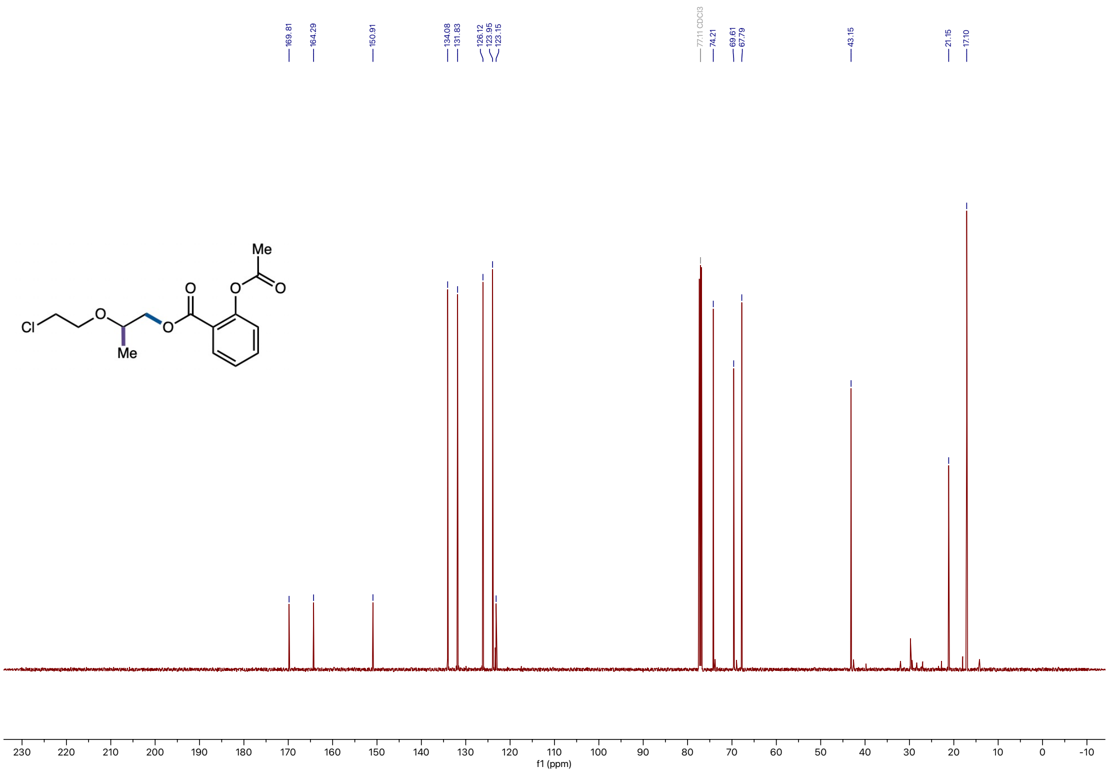

58, ^{1}H NMR, 500 MHz, $CDCl_3$

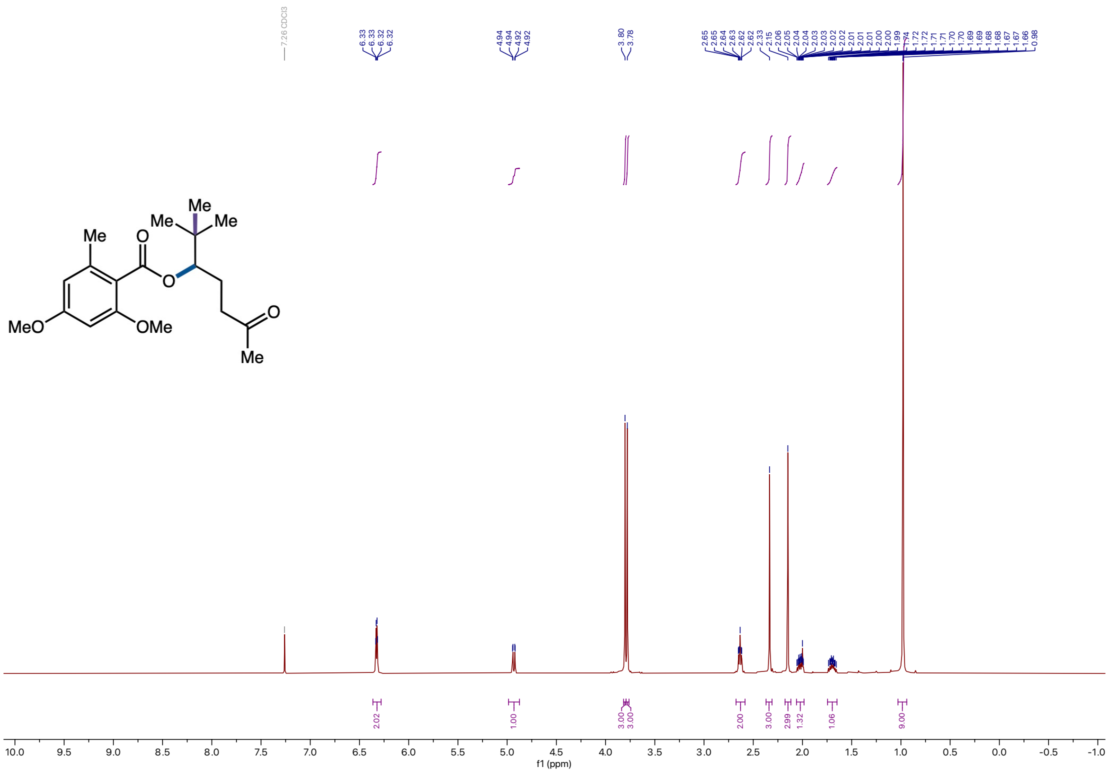

58, ^{13}C NMR, 126 MHz, $CDCl_3$

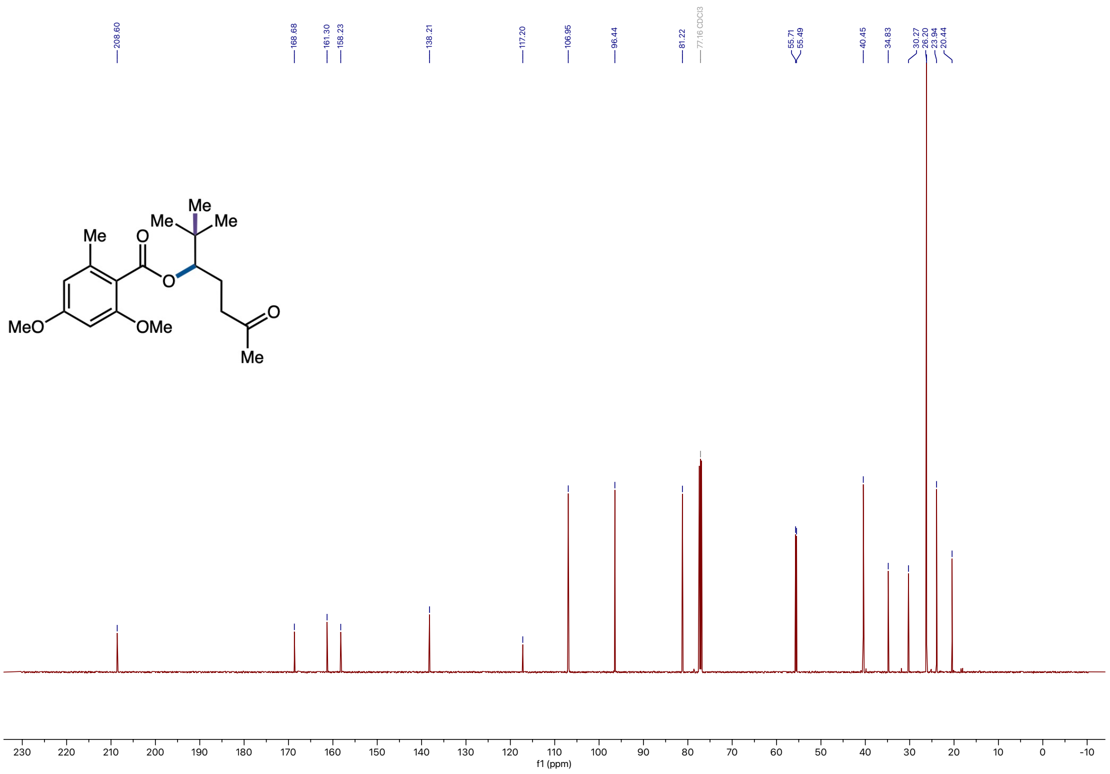

59, ^{1}H NMR, 500 MHz, $CDCl_3$

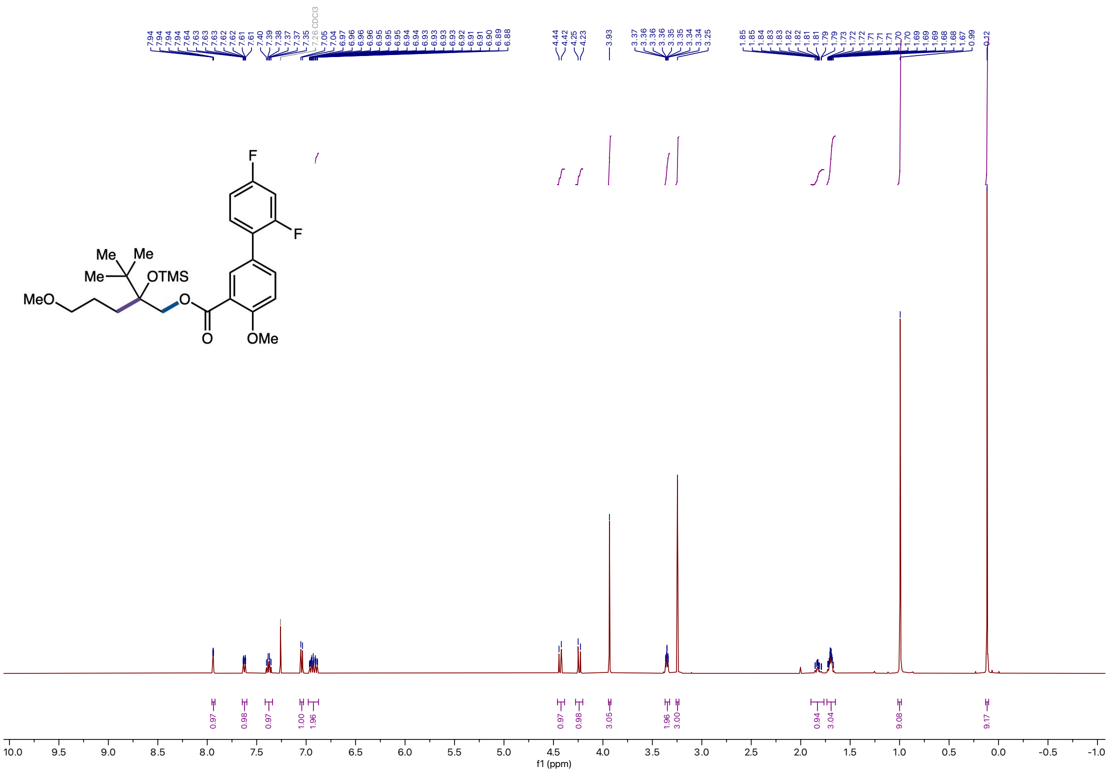

59, ^{13}C NMR, 126 MHz, $CDCl_3$

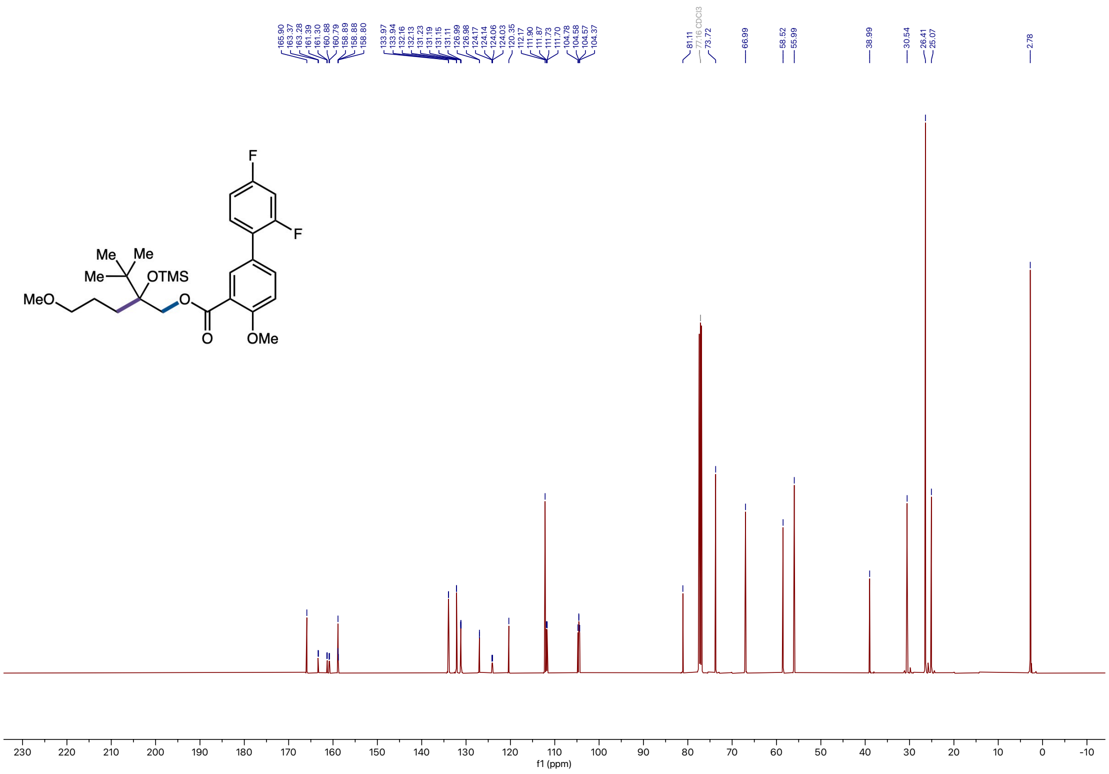

59, ^{19}F NMR, 376 MHz, $CDCl_3$

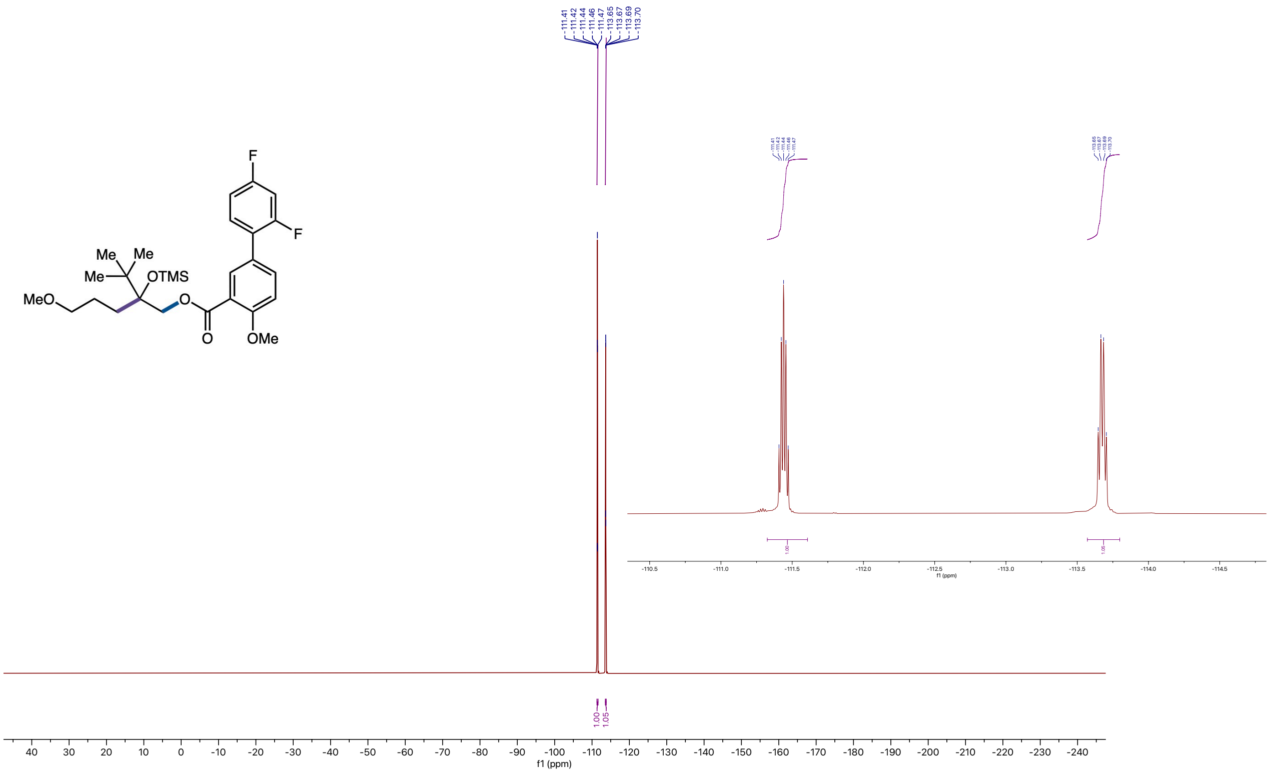

60, ^{1}H NMR, 500 MHz, $CDCl_3$

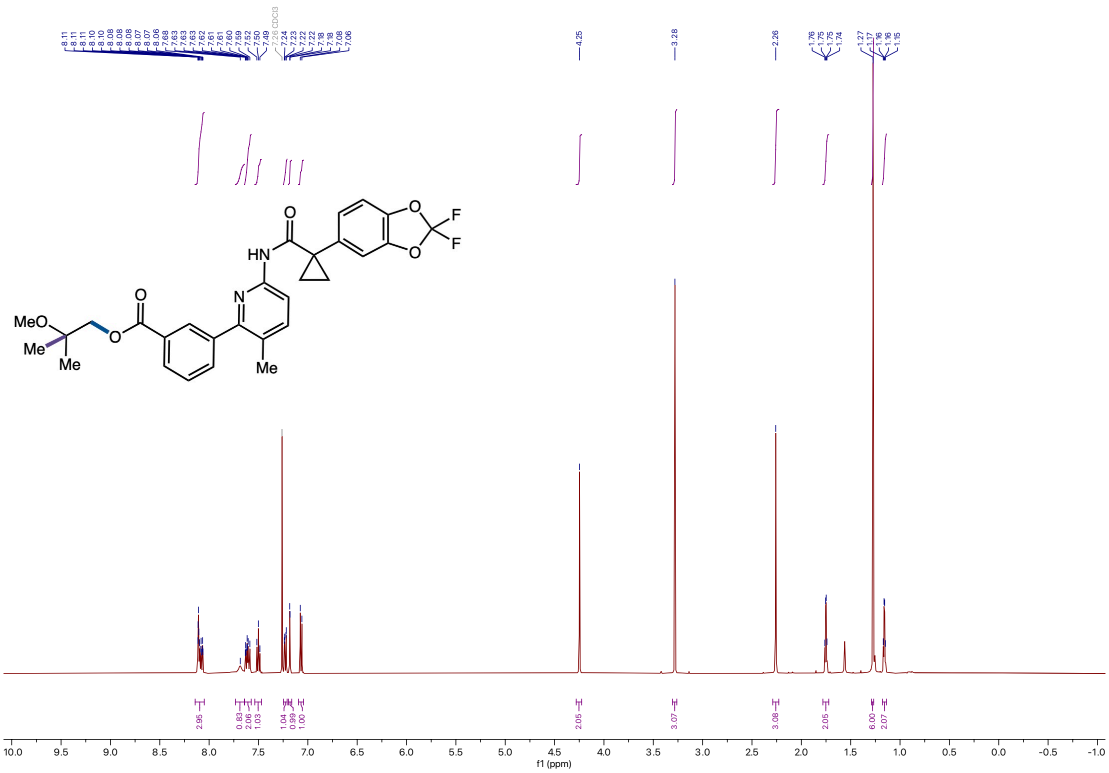

60, ^{13}C NMR, 126 MHz, $CDCl_3$

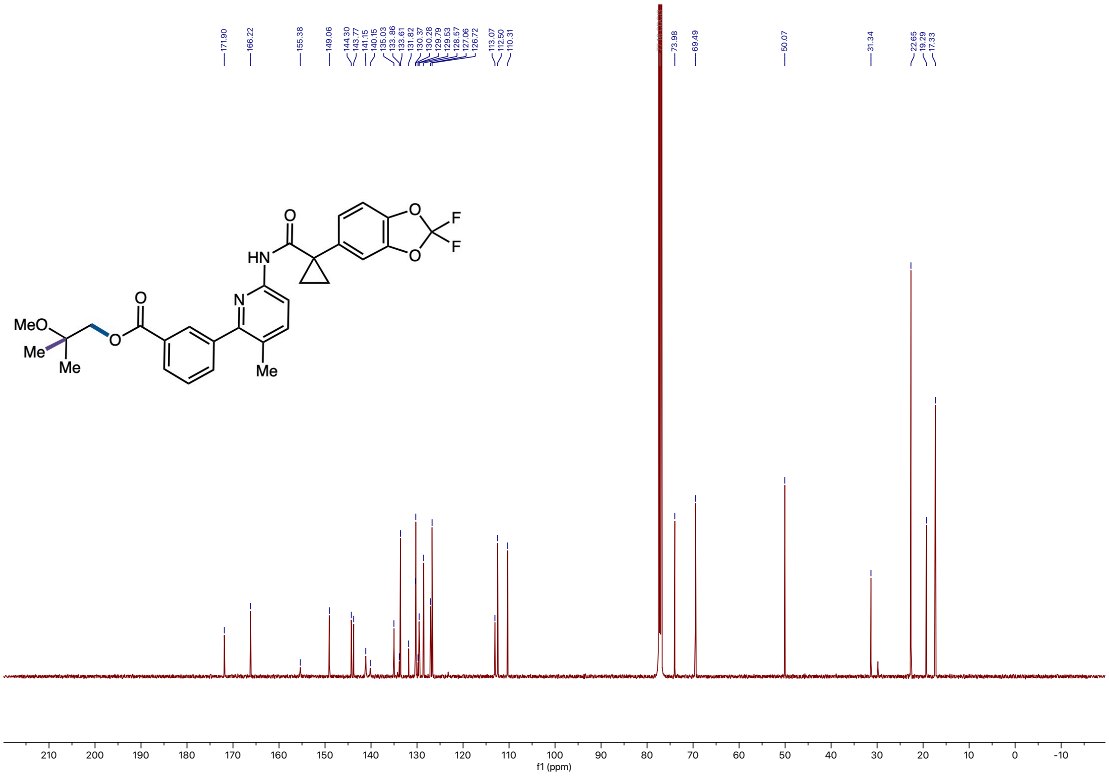

60, ^{19}F NMR, 376 MHz, $CDCl_3$

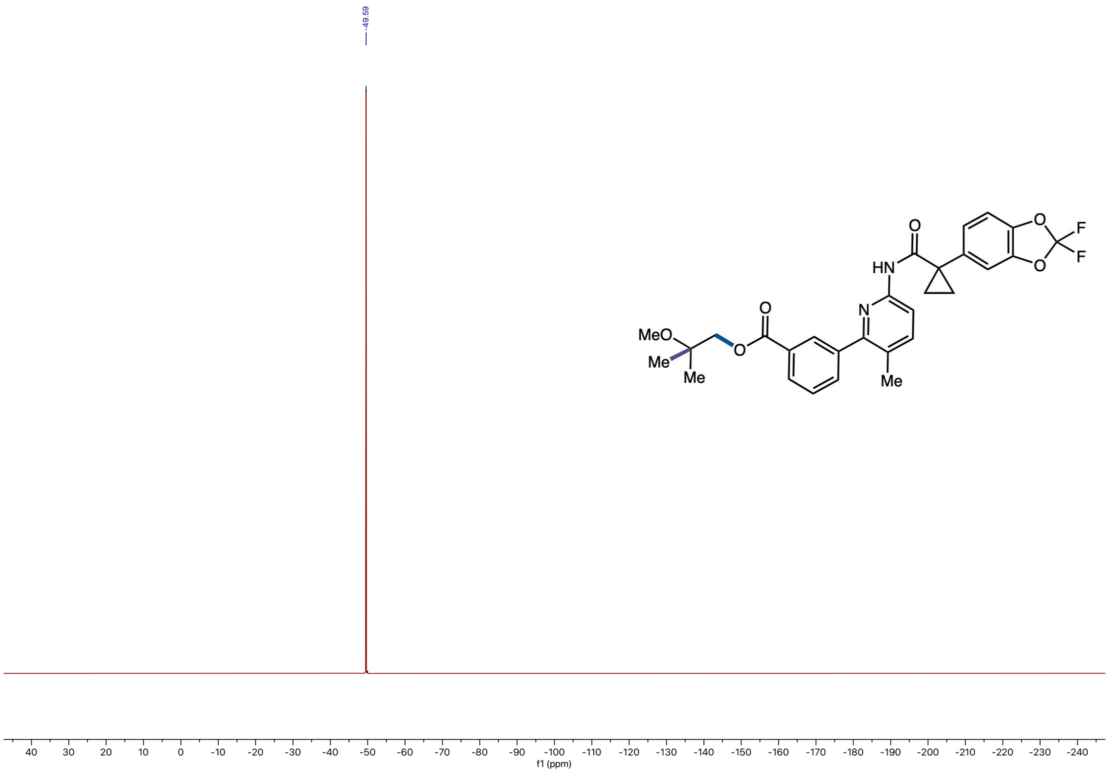

61, ^{1}H NMR, 500 MHz, $CDCl_3$

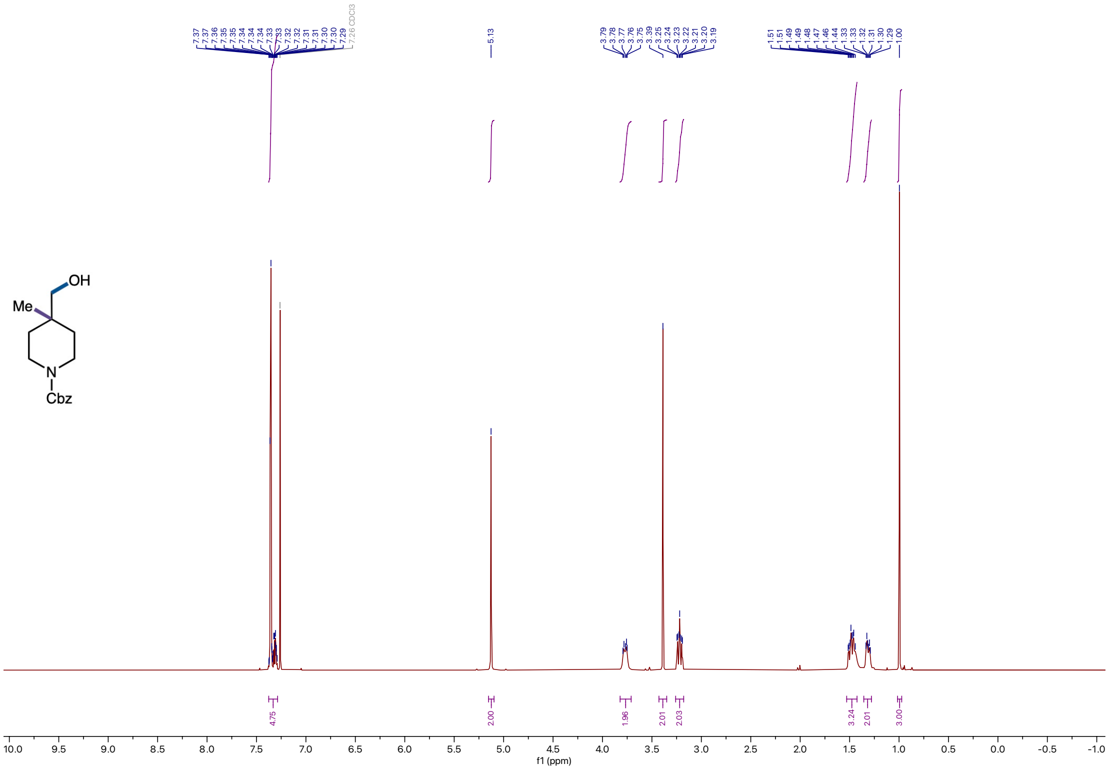

61, ^{13}C NMR, 126 MHz, $CDCl_3$

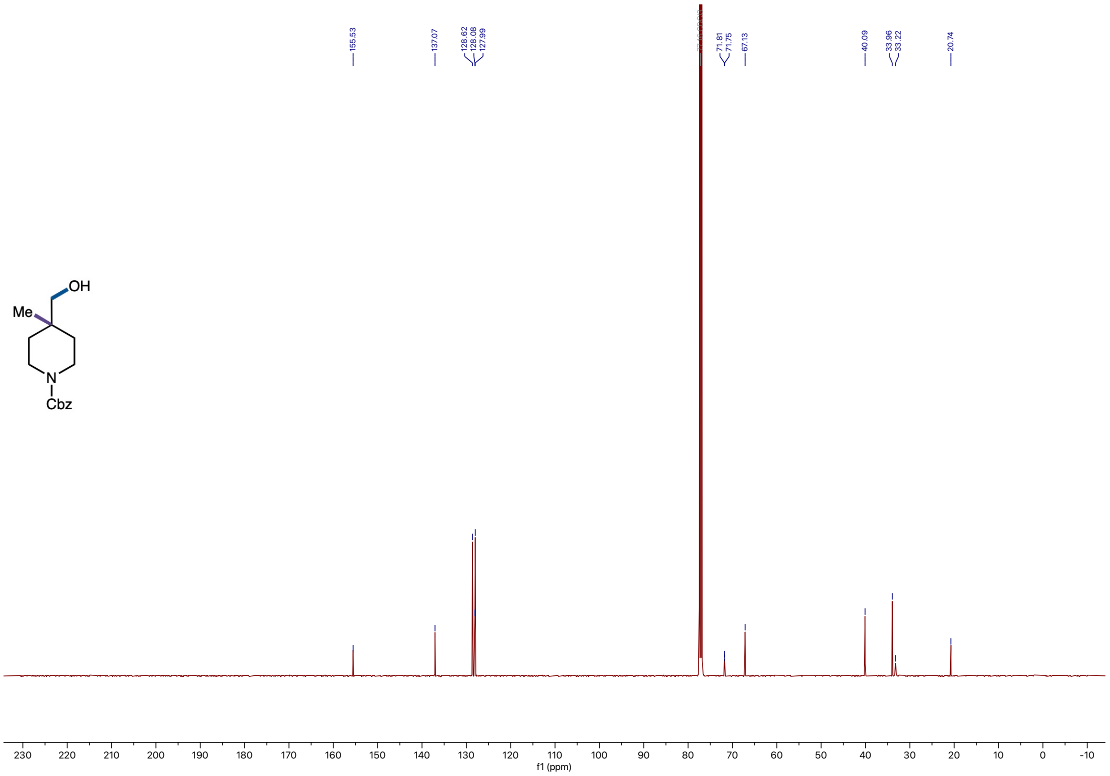

64, ^{1}H NMR, 500 MHz, $CDCl_3$

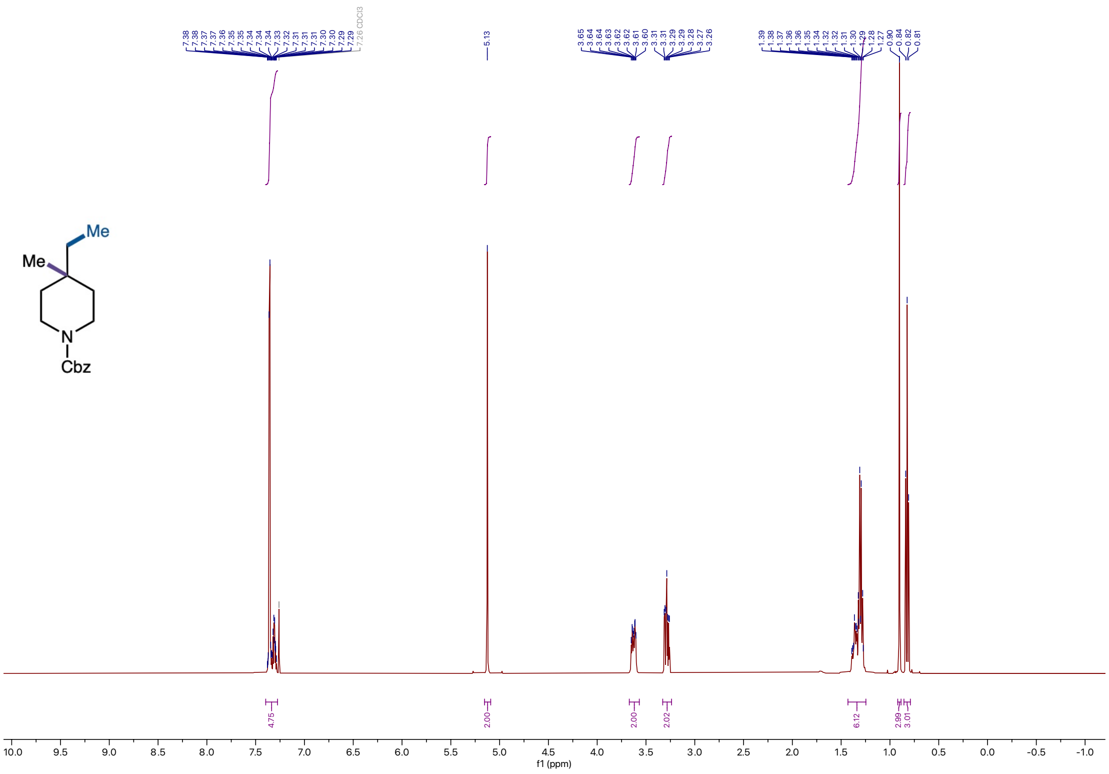

64, ^{13}C NMR, 126 MHz, $CDCl_3$

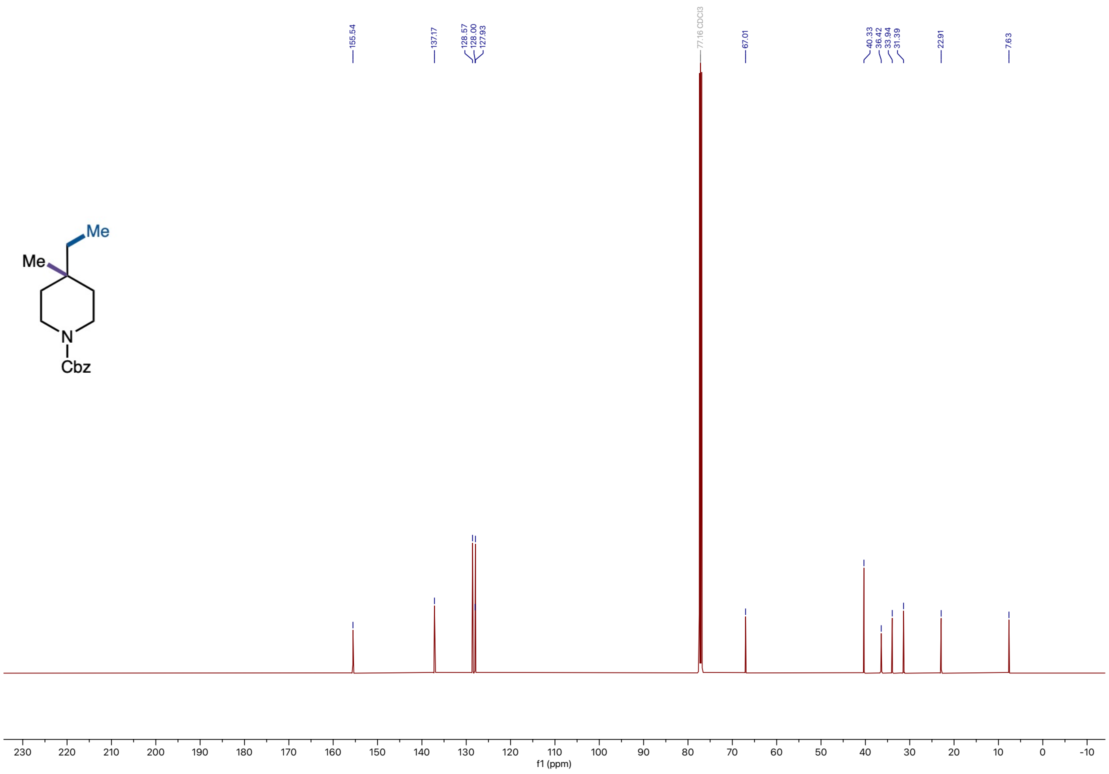

65, ^{1}H NMR, 500 MHz, $(CD_3)_2CO$

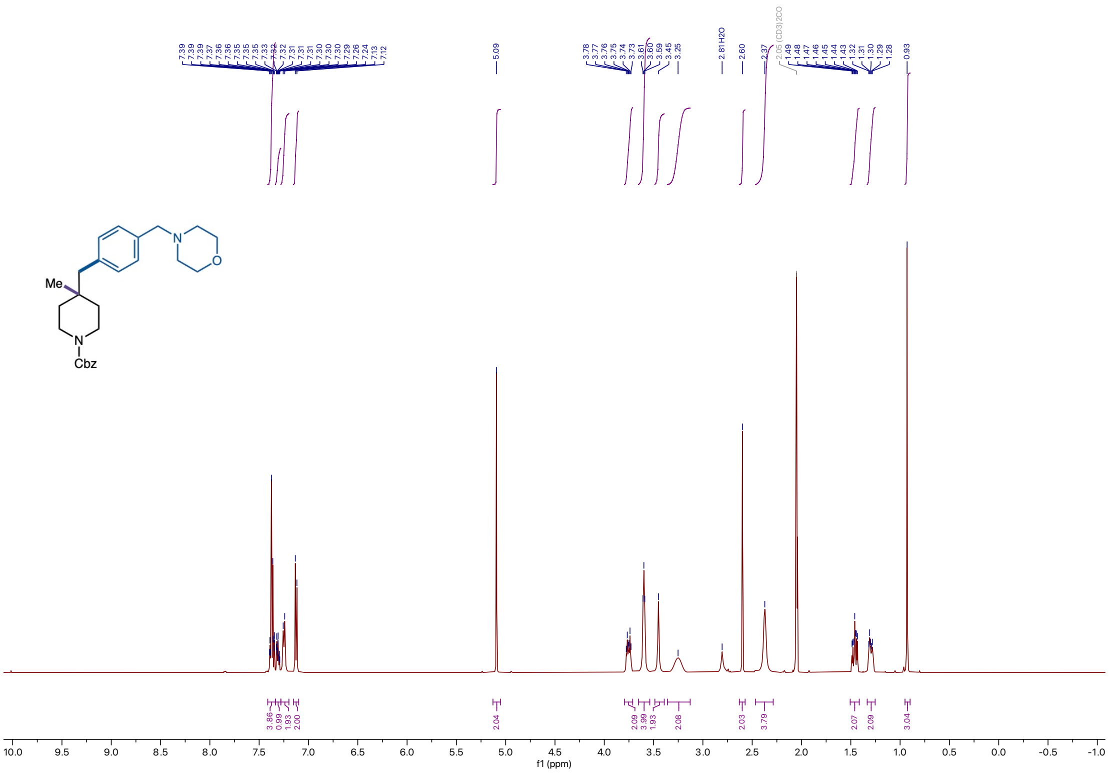

65, ^{13}C NMR, 126 MHz, $(CD_3)_2CO$

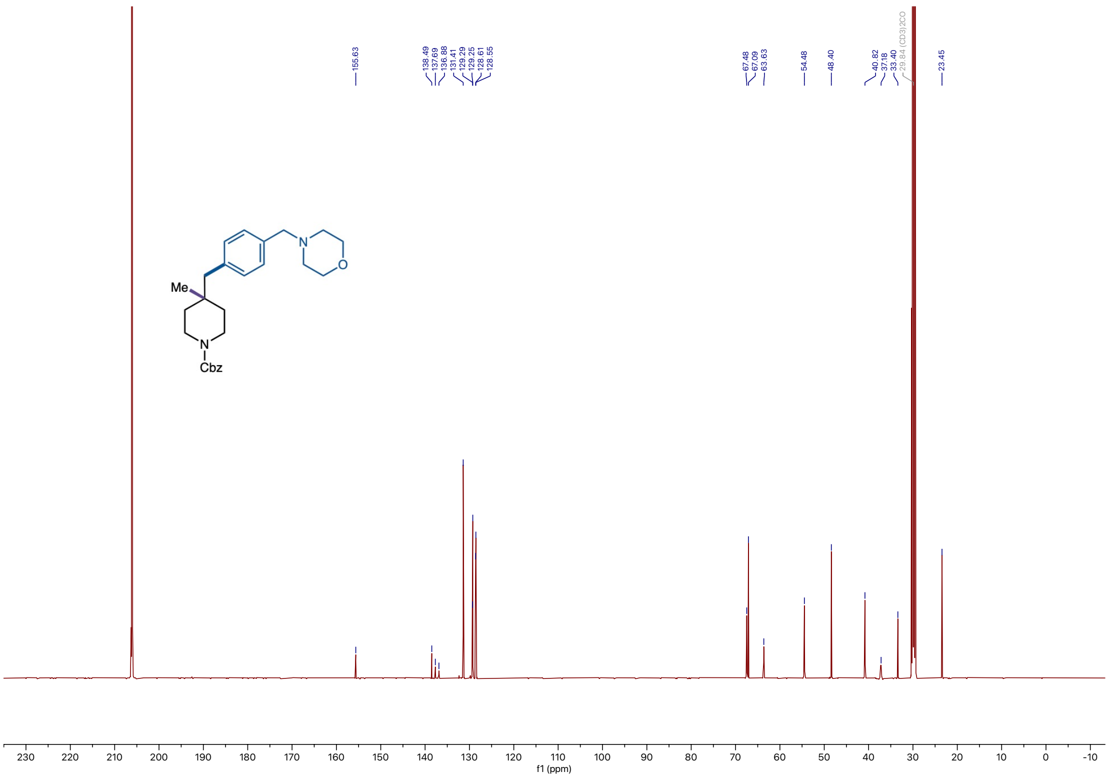

66, ^{1}H NMR, 500 MHz, $CDCl_3$

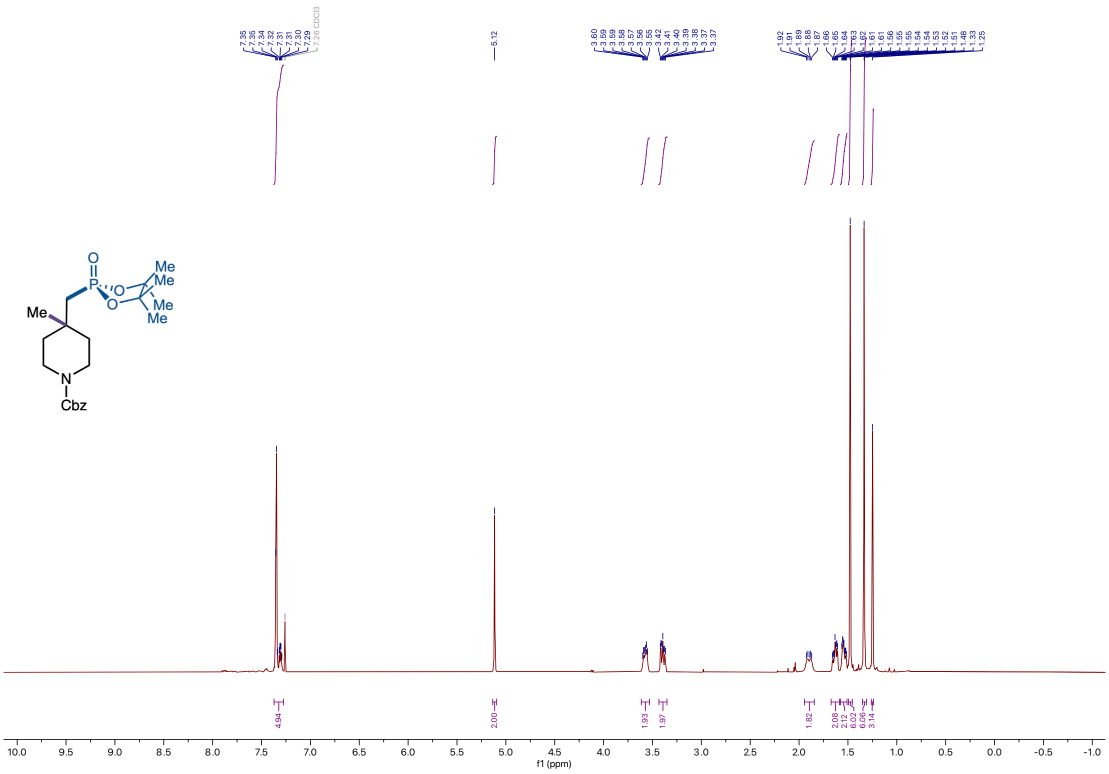

66, ^{13}C NMR, 126 MHz, $CDCl_3$

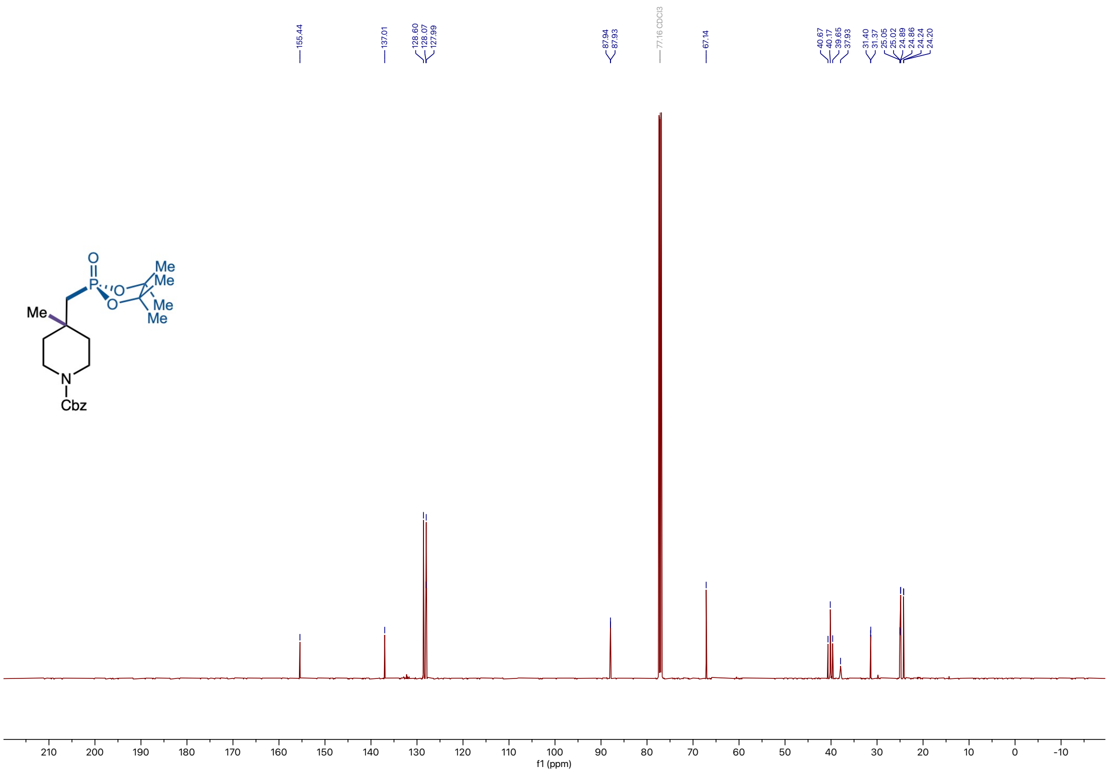

66, ^{31}P NMR, 162 MHz, $CDCl_3$

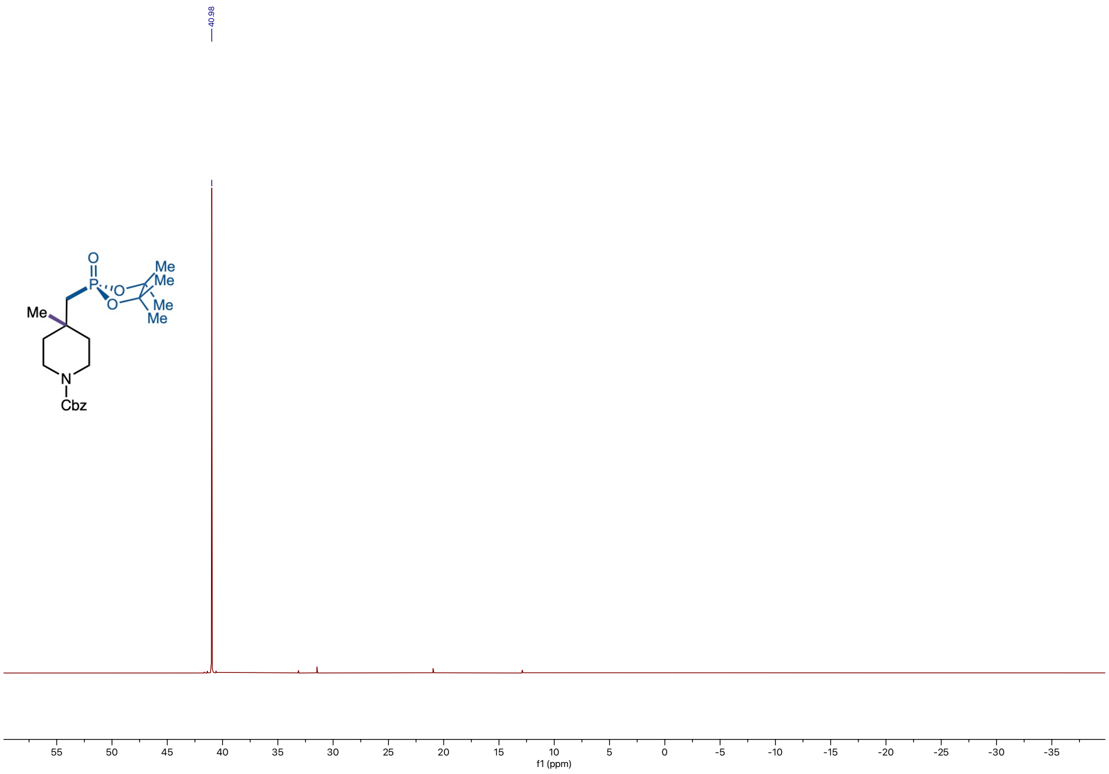

67, ^{1}H NMR, 500 MHz, $CDCl_3$

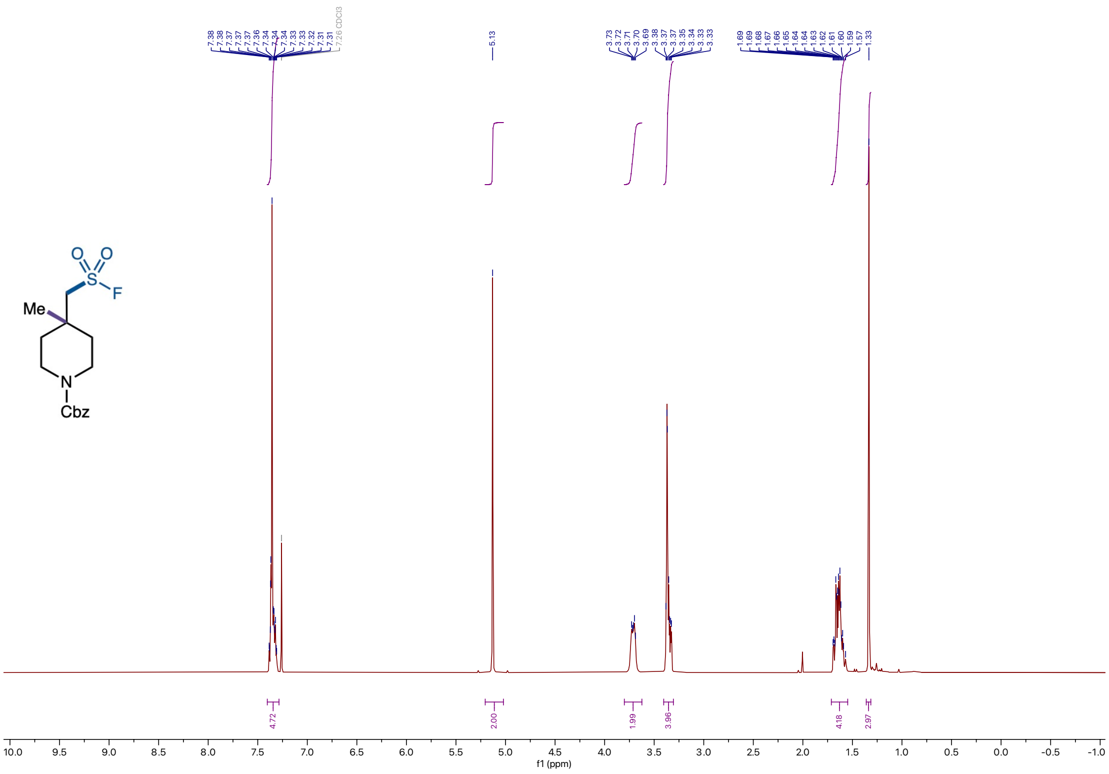

67, ^{13}C NMR, 126 MHz, $CDCl_3$

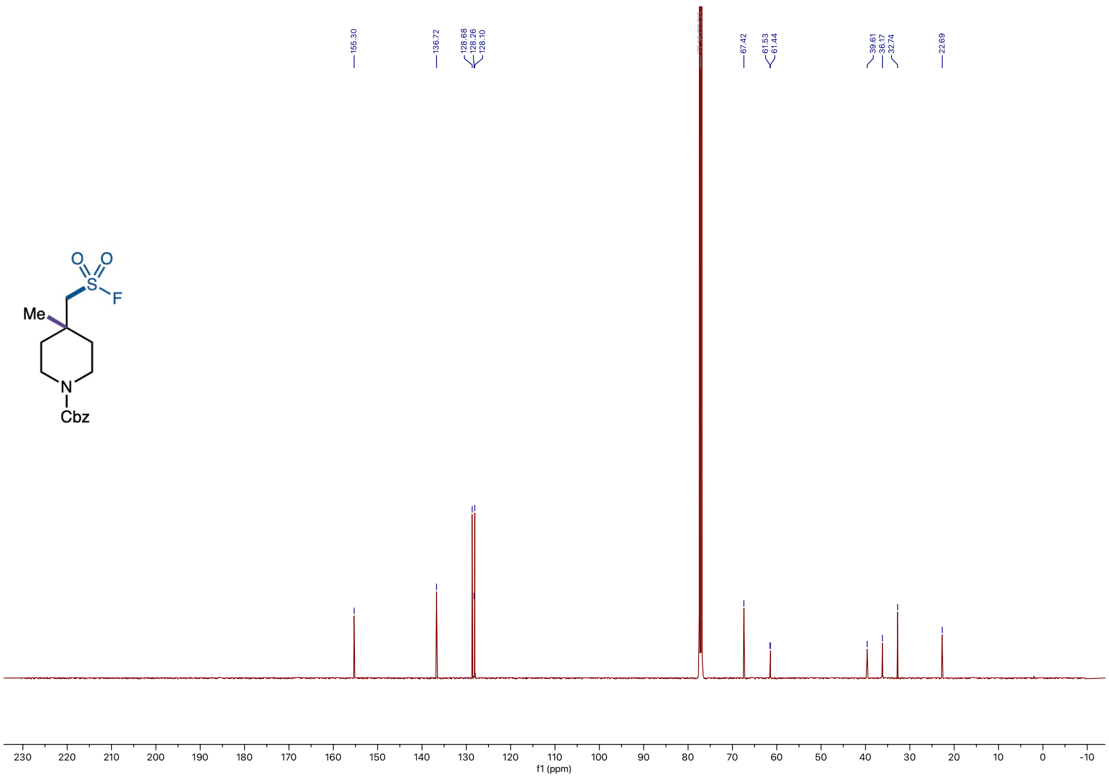

67, ^{19}F NMR, 376 MHz, $CDCl_3$

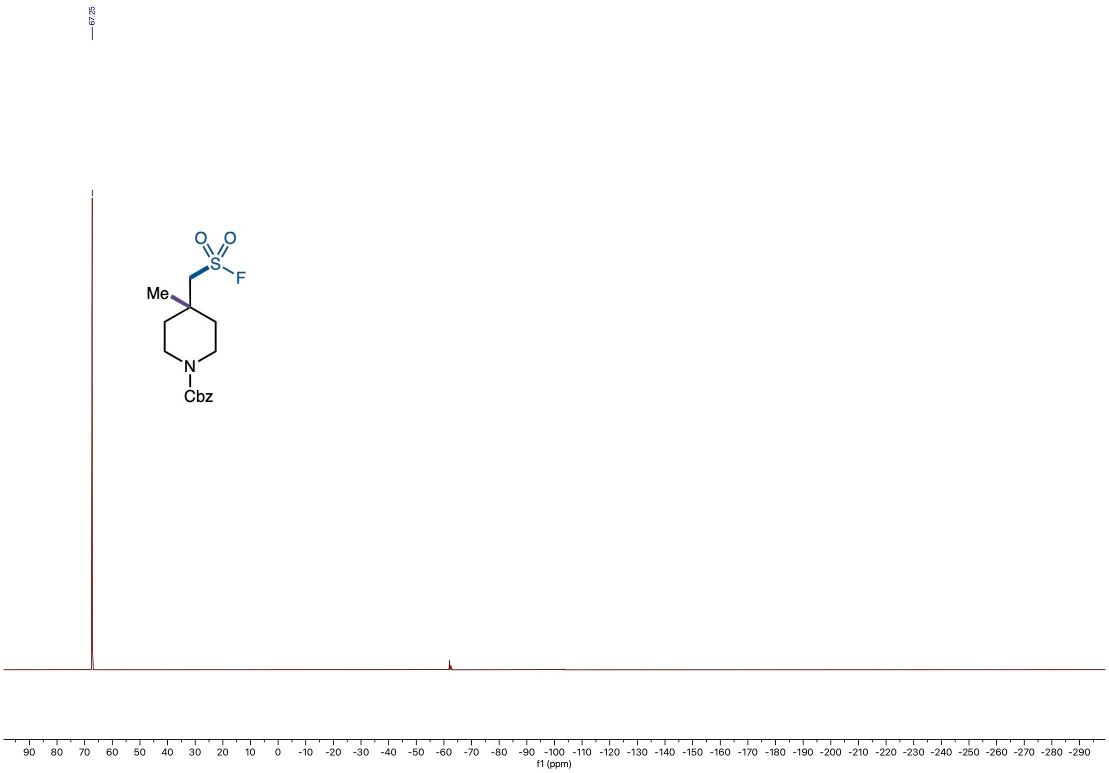

11. References

1. Zhu, Y.-Y.; Lan, G.; Fan, Y.; Veroneau, S.S.; Song, Y.; Micheroni, D.; Lin, W. Merging Photoredox and Organometallic Catalysts in a Metal-Organic Framework Significantly Boosts Photocatalytic Activities. *Angew. Chem. Int. Ed.* **2018**, *57* (43), 14090-14094.
2. Lowry, M. S.; Goldsmith, J. I.; Slinker, J. D.; Rohl, R.; Pascal, R. A.; Malliaras, G. G.; Bernhard, S. Single-Layer Electroluminescent Devices and Photoinduced Hydrogen Production from an Ionic Iridium(III) Complex. *Chem. Mater.* **2005**, *17* (23), 5712–5719.
3. Pangborn, A. B.; Giardello, M. A.; Grubbs, R. H.; Rosen, R. K.; Timmers, F. J. Safe and Convenient Procedure for Solvent Purification. *Organometallics* **1996**, *15* (5), 1518–1520.
4. Lindner, H.; Amberg, W. M.; Carreira, E. M. Iron-Mediated Photochemical Anti-Markovnikov Hydroazidation of Unactivated Olefins. *J. Am. Chem. Soc.* **2023**, *145* (41), 22347-22353.
5. Nagaki, A.; Sasatsuki, K.; Ishiuchi, S.; Miuchi, N.; Takumi, M.; Yoshida, J.-I. Synthesis of Functionalized Ketones from Acid Chlorides and Organolithiums by Extremely Fast Micromixing. *Chem. Eur. J.* **2019**, *25* (19), 4946-4950.
6. Fürstner, A.; Castanet, A.-S.; Radkowski, K.; Lehmann, C. W. Total Synthesis of (*S*)-(+)-Citreofuran by Ring Closing Alkyne Metathesis. *J. Org. Chem.* **2003**, *68* (4), 1521-1528.
7. Joshi, S. M.; Wilson, T. C.; Li, Z.; Preshlock, S.; Gómez-Vallejo, V.; Gouverneur, V.; Llop, J.; Arsequell, G. Synthesis and PET Imaging Biodistribution Studies of Radiolabeled Iododiflunisal, a Transthyretin Tetramer Stabilizer, Candidate Drug for Alzheimer's Disease. *Molecules* **2024**, *29* (2), 488.
8. Gould, C. A.; Pace, A. L.; MacMillan, D. W. C. Rapid and Modular Access to Quaternary Carbons from Tertiary Alcohols via Bimolecular Homolytic Substitution. *J. Am. Chem. Soc.* **2023**, *145* (30), 16330-16336.
9. Kwon, K.; Simons, R. T.; Nandakumar, M.; Roizen, J. L. Strategies to Generate Nitrogen-centered Radicals That May Rely on Photoredox Catalysis: Development in Reaction Methodology and Applications in Organic Synthesis. *Chem. Rev.* **2022**, *122* (2), 2353-2428.
10. Wu, Q.-A.; Chen, F.; Ren, C.-C.; Liu, X.-F.; Chen, H.; Xu, L.-X.; Yu, X.-C.; Luo, S.-P. Donor–acceptor fluorophores as efficient energy transfer photocatalysts for [2 + 2] photodimerization. *Org. Biomol. Chem.* **2020**, *18*, 3707-3716.
11. Ruos, M. E.; Kinney, R. G.; Ring, O. T.; Doyle, A. G. A General Photocatalytic Strategy for Nucleophilic Amination of Primary and Secondary Benzylic C−H Bonds. *J. Am. Chem. Soc.* **2023**, *145* (33)x, 18487-18496.
12. Chen, R.; Intermaggio, N. E.; Xie, J.; Rossi-Ashton, J. A.; Gould, C. A.; Martin, R. T.; Alcázar, J.; MacMillan, D. W. C. Alcohol-alcohol cross-coupling enabled by S_H2 radical sorting. *Science* **2024**, *383* (6689), 1350–1357.
13. Dong, Z.; MacMillan, D. W. C. Metallaphotoredox-enabled deoxygenative arylation of alcohols. *Nature* **2021**, *598*, 451–456.
14. Bissonnette, N. B.; Bisballe, N.; Tran, A. V.; Rossi-Ashton, J. A.; MacMillan, D. W. C. Development of a General Organophosphorus Radical Trap: Deoxyphosphonylation of Alcohols. *J. Am. Chem. Soc.* **2024**, *146* (12), 7942–7949.

15. Carson II, W. P.; Sarver, P. J.; Goudy, N. S.; MacMillan, D. W. C. Photoredox Catalysis-Enabled Sulfination of Alcohols and Bromides. *J. Am. Chem. Soc.* **2023**, *145* (38), 20767–20774.
16. Schwarz, S.; Ring, S.; Weber, G.; Teichmüller, G.; Palme, H.-J.; Pfeiffer, C.; Undeutsch, B.; Erhart, B.; Grawe, D. Synthesis of 13-Ethyl-11-methylene-18,19-dinor-17α-pregn-4-en-20-yn-17-ol (Desogestrel) and its Main Metabolite 3-Oxo Desogestrel. *Tetrahedron* **1994***, 50* (36), 10709-10720.
